# The genome of *Hippophae salicifolia* provides new insights into the sexual differentiation of sea buckthorn

**DOI:** 10.1093/gigascience/giaf046

**Published:** 2025-07-02

**Authors:** Mingyue Chen, Xingyu Yang, Lan Xun, Zhenlin Qu, Shihai Yang, Yunqiang Yang, Yongping Yang

**Affiliations:** CAS Key Laboratory of Tropical Plant Resources and Sustainable Use, Xishuangbanna Tropical Botanical Garden, Chinese Academy of Sciences, Mengla, Yunnan 666303, China; Yunnan International Joint Laboratory for the Conservation and Utilization of Tropical Timber Tree Species, Xishuangbanna Tropical Botanical Garden, Chinese Academy of Sciences, Mengla, Yunnan 666303, China; School of Ecology and Environment, Yunnan University, Kunming 650091, China; CAS Key Laboratory of Tropical Plant Resources and Sustainable Use, Xishuangbanna Tropical Botanical Garden, Chinese Academy of Sciences, Mengla, Yunnan 666303, China; Yunnan International Joint Laboratory for the Conservation and Utilization of Tropical Timber Tree Species, Xishuangbanna Tropical Botanical Garden, Chinese Academy of Sciences, Mengla, Yunnan 666303, China; CAS Key Laboratory of Tropical Plant Resources and Sustainable Use, Xishuangbanna Tropical Botanical Garden, Chinese Academy of Sciences, Mengla, Yunnan 666303, China; College of Agriculture and Biotechnology, Yunnan Agricultural University, Kunming 650500, China; Beijing Jiu Tian International Education, Beijing 100080, China; Xizang Ecological Harmony Seed Industry Co, Ltd, Shigatse, Xizang 857000, China; CAS Key Laboratory of Tropical Plant Resources and Sustainable Use, Xishuangbanna Tropical Botanical Garden, Chinese Academy of Sciences, Mengla, Yunnan 666303, China; Yunnan International Joint Laboratory for the Conservation and Utilization of Tropical Timber Tree Species, Xishuangbanna Tropical Botanical Garden, Chinese Academy of Sciences, Mengla, Yunnan 666303, China; CAS Key Laboratory of Tropical Plant Resources and Sustainable Use, Xishuangbanna Tropical Botanical Garden, Chinese Academy of Sciences, Mengla, Yunnan 666303, China; Yunnan International Joint Laboratory for the Conservation and Utilization of Tropical Timber Tree Species, Xishuangbanna Tropical Botanical Garden, Chinese Academy of Sciences, Mengla, Yunnan 666303, China

**Keywords:** *Hippophae*, chromosomal assembly, sex chromosomes, sex determination

## Abstract

**Background:**

Dioecy, a common reproductive strategy in angiosperms, has evolved independently in various plant lineages, and this has resulted in the evolution of diverse sex chromosome systems and sex determination mechanisms. *Hippophae* is a genus of dioecious plants with an XY sex determination system, but the molecular underpinnings of this process have not yet been clarified. Most previously published sea buckthorn genome data have been derived from females, yet genomic data on males are critically important for clarifying our understanding of sex determination in this genus. Comparative genomic analyses of male and female sea buckthorn plants can shed light on the origins and evolution of sex. These studies can also enhance our understanding of the molecular mechanisms underlying sexual differentiation and provide novel insights and data for future research on sexual reproduction in plants.

**Results:**

We conducted an in-depth analysis of the genomes of 2 sea buckthorn species, including a male *Hippophae gyantsensis*, a female *Hippophae salicifolia*, and 2 haplotypes of male *H. salicifolia*. The genome size of *H. gyantsensis* was 704.35 Mb, and that of the female *H. salicifolia* was 788.28 Mb. The sizes of the 2 haplotype genomes were 1,139.99 Mb and 1,097.34 Mb. The sex-determining region (SDR) of *H. salicifolia* was 29.71 Mb and contained 249 genes. A comparative analysis of the haplotypes of Chr02 of *H. salicifolia* revealed that the Y chromosome was shorter than the X chromosome. Chromosomal evolution analysis indicated that *Hippophae* has experienced significant chromosomal rearrangements following 2 whole-genome duplication events, and the fusion of 2 chromosomes has potentially led to the early formation of sex chromosomes in sea buckthorn. Multiple structural variations between Y and X sex-linked regions might have facilitated the rapid evolution of sex chromosomes in *H. salicifolia*. Comparison of the transcriptome data of male and female flower buds from *H. gyantsensis* and *H. salicifolia* revealed 11 genes specifically expressed in males. Three of these were identified as candidate genes involved in the sex determination of sea buckthorn. These findings will aid future studies of the sex determination mechanisms in sea buckthorn.

**Conclusion:**

A comparative genomic analysis was performed to identify the SDR in *H. salicifolia*. The origins and evolutionary trajectories of sex chromosomes within *Hippophae* were also determined. Three potential candidate genes associated with sea buckthorn sex determination were identified. Overall, our findings will aid future studies aimed at clarifying the mechanisms of sex determination.

## Introduction

Clarifying the genetic mechanisms underlying sexual differentiation, as well as the origins and evolution of sex chromosomes, is a major goal of evolutionary biology. Conventional theories suggest that sex chromosomes evolve from autosomes through a series of genetic and evolutionary changes, which ultimately become determinants of sex. This evolutionary trajectory is often initiated with 2 closely linked mutations on an autosome: a dominant mutation that inhibits female development and a recessive mutation inducing male sterility [1, 2]. Due to the genetic conflict associated with sex differences in reproductive strategies, sexually antagonistic genes accumulate around these mutant genes, which suppresses recombination [3]. As time progresses, the inhibition of recombination can extend along the chromosome, leading to the establishment of sex-linked regions, and eventually may lead to the emergence of heteromorphic sex chromosomes [4, 5]. However, previous studies have shown that the evolution of sex chromosomes does not always result in heteromorphic pairs. For example, the sex chromosomes in scallops have remained highly undifferentiated for over 350 million years. In these species, homomorphic sex chromosomes are notably enriched with numerous bidirectionally reversible sex-biased genes, which might be critically important for maintaining their undifferentiated state [6]. These findings indicate that sex chromosome evolution can follow various patterns. Many aspects of sex chromosomes have not yet been clarified, including the origin of sex and recombination suppression [7]. In contrast to the pronounced differentiation in human and animal sex chromosomes, plant sex chromosomes have arisen and evolved independently across diverse dioecious plant lineages, and they exhibit high diversity and complexity in their evolutionary pathways [8]. Investigation of the sex determination mechanisms in these plants can provide novel insights and data crucial for elucidating the genetic basis of sexual differentiation.

Dioecious plants account for 5% to 6% of angiosperms; sexual differentiation in dioecious plants provides them with unique reproductive advantages [9]. Dioecious plants can optimize the allocation of reproductive resources and enhance breeding efficiency through sexual differentiation [10]. Furthermore, sexual differentiation can affect their ecological niche [11, 12]. Some dioecious plants may reduce self-fertilization and increase gene flow through sexual differentiation, which enhances the genetic diversity of the population. The differentiation of sex chromosomes plays a key role in the molecular mechanism underlying sexual differentiation in dioecious plants. Sex chromosomes, such as the XY and ZW systems, are typically derived from autosomes and evolve into sex-determining chromosomes via a series of genetic and evolutionary processes [2]. The expression patterns of genes on the sex chromosomes, the formation of recombination suppression regions, and the morphological and functional differentiation of sex chromosomes are all key factors affecting these processes [13, 14]. Sex-specific gene expression patterns play a key role in sex determination. In dioecious plants, certain genes may be expressed in only 1 sex, and these differences in gene expression directly affect sex-specific traits [15]. The formation of regions with suppressed recombination is another critical factor affecting sex chromosome differentiation. The recombination frequency in certain regions significantly decreases during sex chromosome evolution, and this phenomenon is known as recombination suppression [14]. Recombination suppression aids the accumulation of sex-specific genes and the stabilization of sex-determining mechanisms. In these regions, gene flow is restricted, which promotes the differentiation of sex-specific genes and the morphological differentiation of sex chromosomes [16].

The identification and functional analysis of sex-determining genes are critically important in studies of sexual differentiation in dioecious plants [8]. Dioecious plants have evolved independently in different lineages and possess distinct sex-determining genes. However, these genes are products of convergent evolution and have similar characteristics that can facilitate their identification. However, the identification of sex-determining genes is extremely challenging because of various factors, such as heterochromatinization and structural variation; the sex-determining genes of only a few species have been successfully characterized to date. Diospyros lotus is the first plant from which sex-determining genes were successfully isolated and identified [17]. Illumina sequencing data from the F1 generation of both female and male plants have been used for the assembly of the male-specific region (MSY) of the Y chromosome. They were then integrated into bud transcriptome data, and 22 differentially expressed genes were identified within the MSY. Phylogenetic analysis revealed that *OGI* is the only gene that predates the speciation event within Diospyros. Evolutionary analyses indicated that *OGI* is a best candidate for a sex-determining gene, and this hypothesis was confirmed in subsequent experiments. Similarly, the MSY region was assembled in kiwifruit using Illumina sequencing data from the F1 generation, and differential expression analysis was performed using transcriptome data from flower buds at various developmental stages. This approach led to the identification of 2 male-specific genes, *SyGI* and *FrBy*, which are conserved across all kiwifruit and lack homologous counterparts in females. Transgenic studies have shown that *SyGI* is a dominant inhibitor of carpel development, and *FrBy* knockout results in pollen inactivation and self-incompatibility [18, 19]. Despite advances in sequencing technology, which has enabled the identification of some plant sex-determining genes, only a few such genes have been isolated and characterized.

Sea buckthorn (*Hippophae*), which belongs to the family Elaeagnaceae, is a shrub or small tree with distinct dioecious characteristics and simple and delicate flowers. The berries of sea buckthorn are renowned for their medicinal properties and nutritional richness; they thus play a key role in the pharmaceutical, cosmetic, and food industries [20, 21]. This species has been used for ecological restoration because of its high stress tolerance and vigorous growth; it also can reproduce prolifically and shows significant nitrogen-fixation activity, which makes it ideal for wind protection, sand stabilization, and reforestation in arid regions [22]. Sea buckthorn has an XY sex chromosome system and shows clear sexual dimorphism; males typically bear cone-like inflorescences that bloom before the females, whereas females have raceme inflorescences that are comparatively smaller [23]. In contrast, the *Elaeagnus* genus, which belongs to the same family, has undergone 2 whole-genome duplication events but remains monoecious. However, sea buckthorn transitioned from monoecy to dioecy after these events [24, 25]. The genomic sequences of *Hippophae tibetana* [26, 27], *Hippophae rhamnoides* subsp. *sinensis* [28], *Hippophae rhamnoides* subsp. *mongolica* [24], and *Hippophae gyantsensis* [29] have all been published. The sex chromosome of *H. tibetana* was identified as the second chromosome through simplified genome-wide association studies [26]; however, detailed research on its sex chromosome has not yet been conducted. Moreover, no studies have compared the genomic differences between sea buckthorn and *Elaeagnus*. Therefore, studies of the sex chromosomes of male sea buckthorn at the genomic level can provide important insights into sexual differentiation in dioecious plants and genetic resources that could aid the breeding of sea buckthorn. These findings will aid future molecular studies of the sex determination mechanisms of sea buckthorn, as well as ongoing efforts to improve varieties and their applications in medicine, ecology, and other fields.

Here, we assembled the genomes of a male *H. gyantsensis* (NCBI:txid193515), a female *Hippophae salicifolia* (NCBI:txid48234), and 2 male *H. salicifolia* with haplotype resolution. Specifically, we conducted a comparative genomic analysis to identify the sex-determining regions (SDRs) in both species and examined their genomic distribution and characteristics, along with the origin and evolution of the sex chromosomes of sea buckthorn. Analysis of transcriptomic data from male and female flowers revealed 3 candidate genes. Our findings provide new insights into sexual differentiation in sea buckthorn and will aid ongoing efforts to enhance sex-related traits and molecular breeding in the future.

## Results

### Genome estimation, sequencing, and assembly

To evaluate the genome size and heterozygosity of the 3 sea buckthorn species, we conducted genomic studies using clean reads obtained from the Illumina next-generation sequencing (NGS) platform: male *H. salicifolia* (57.60 Gb), male *H. gyantsensis* (51.95 Gb), and female *H. salicifolia* (62.31 Gb) (Supplementary Table S1). The *k*-mer (*k* = 21) analysis (Supplementary Fig. S1) indicated that the genome size of male *H. gyantsensis* was approximately 1,031.76 Mb, with a heterozygosity rate of approximately 1.39%; the genome size of male *H. salicifolia* was approximately 1,101.85 Mb, with a heterozygosity rate of approximately 0.744%; and the genome size of female *H. salicifolia* was approximately 1,134.93 Mb, with a heterozygosity rate of approximately 0.728%.

**Figure 1:**
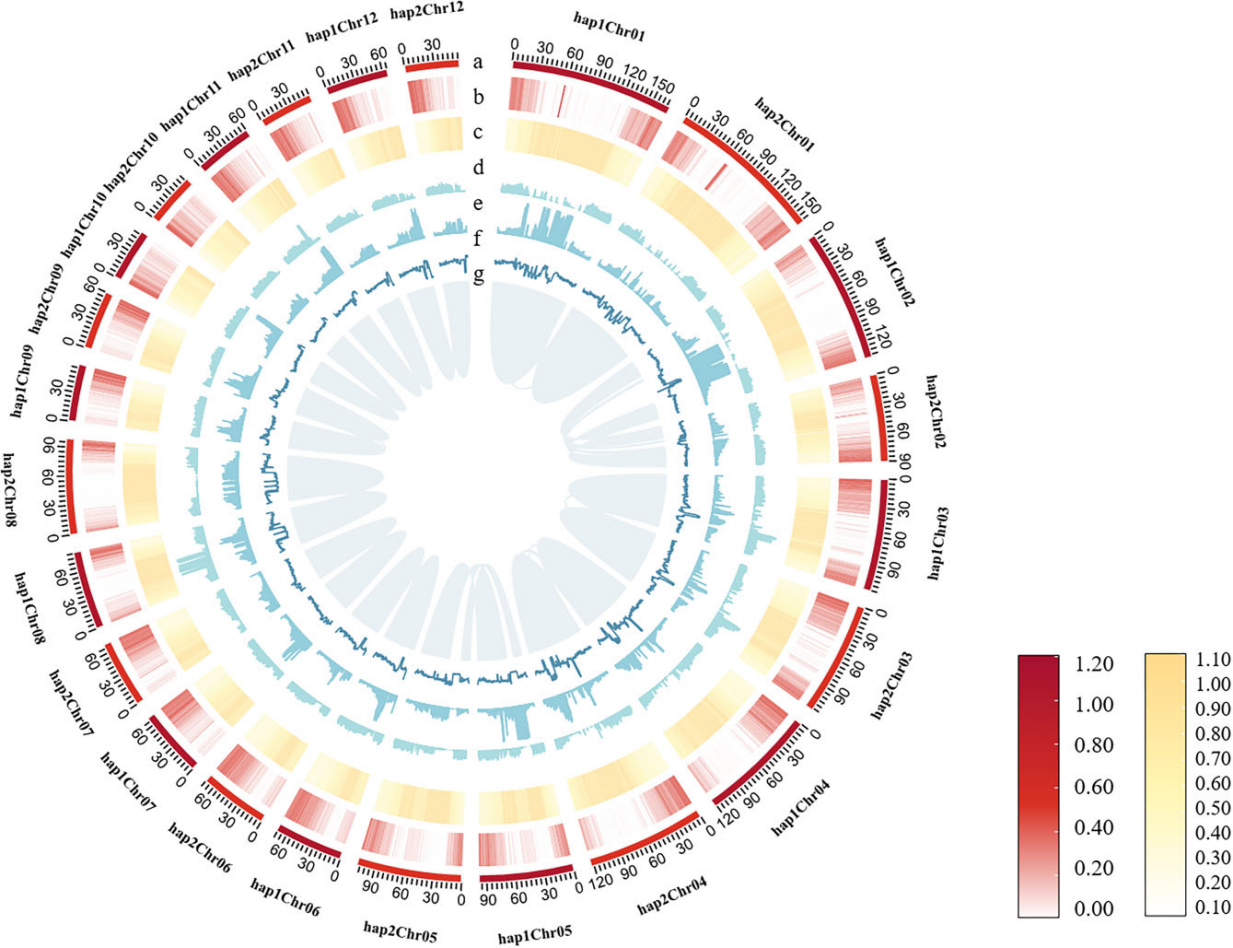
Genomic features of male *H. salicifolia*. (a) Pseudochromosome. (b) Gene density. (c) Repeat sequences density. (d) Ty3 density. (e) Copia density. (f) GC content. (g) Interspecies collinearity. Feature density and GC percentage were calculated with a 5-Mb window size. *H. salicifolia* is diploid, so the haploid genome was used as the reference genome.

We obtained clean reads from Oxford Nanopore Technologies (ONT) sequencing for female *H. salicifolia* (112.31 Gb) and male *H. gyantsensis* (99.03 Gb) to assemble the genomes of the 3 sea buckthorn species; HiFi reads were obtained for male *H. salicifolia* (142.55 Gb) (Supplementary Table S1). Hi-C sequencing generated 79.47 Gb of clean reads for male *H. salicifolia*, 82.52 Gb for female *H. salicifolia*, and 136.13 Gb for male *H. gyantsensis*, which were used for chromosome anchoring (Supplementary Table S2). The contigs of the 3 genomes were anchored to 12 pseudochromosomes (Supplementary Fig. S2). The genome size of male *H. gyantsensis* was 704.35 Mb, with a contig N50 of 18.11 Mb (Supplementary Fig. S3); the genome size of female *H. salicifolia* was 788.28 Mb, with a contig N50 of 30.83 Mb (Supplementary Fig. S4). Male *H. salicifolia* was assembled into 2 haplotypes, Hap1 and Hap2. The genome size of Hap1 was 1,139.99 Mb, with a contig N50 of 53.61 Mb; the genome size of Hap2 was 1,097.34 Mb, with a contig N50 of 64.70 Mb (Supplementary Table S3) (Fig. 1). The genomic BUSCO completeness rates scores were 97.4% for male *H. gyantsensis*, 97.6% for female *H. salicifolia*, 97.6% for male *H. salicifolia* Hap1, and 97.7% for male *H. salicifolia* Hap2 (Supplementary Table S4).

### Genome annotation

The repetitive sequence content of the male *H. gyantsensis* genome was 56.42%, and long terminal repeat (LTR) sequences accounted for 35.06% of the repetitive sequences. The repetitive sequence content of the female *H. salicifolia* genome was 60.41%, and LTRs comprised 37.68% of the repetitive sequences. For Hap1, the repetitive sequence content was 70.88%, of which LTRs comprised 36.96% of the repetitive sequences; for Hap2, the repetitive sequence content was 70.90%, and LTRs comprised 36.14% of the repetitive sequences (Supplementary Table S5).

In the male *H. gyantsensis* genome, 36,482 genes were annotated, and 33,238 were present in at least 1 database: Swissprot, Kyoto Encyclopedia of Genes and Genomes (KEGG), TrEMBL, nonredundant (nr), InterPro, and Gene Ontology (GO). In the female *H. salicifolia* genome, 39,501 genes were annotated, and 34,811 were present in at least 1 of the aforementioned databases. In the Hap1 genome, 45,937 genes were annotated, including 38,287 in at least 1 database. In the Hap2 genome, 39,854 genes were annotated, including 34,460 in at least 1 database (Supplementary Table S6). The BUSCO completeness scores for gene sets in the male *H. gyantsensis*, female *H. salicifolia*, male *H. salicifolia* Hap1, and male *H. salicifolia* Hap2 genomes were 98.9%, 98.8%, 98.7%, and 98.7%, respectively (Supplementary Table S7). These findings confirm the high quality of the 4 sea buckthorn genomes sequenced.

### Comparative genomics analysis

**Figure 2:**
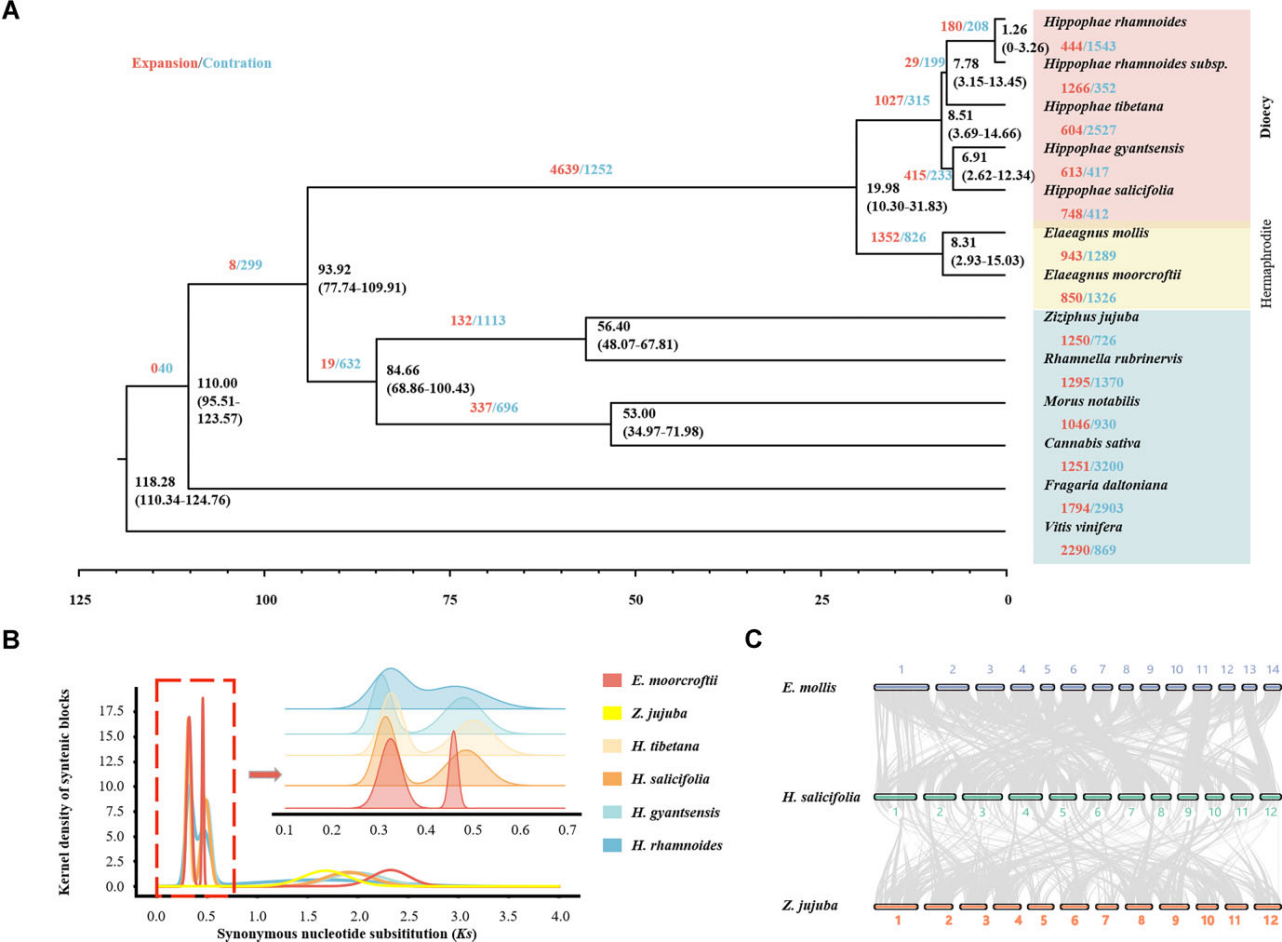
Comparative genomics analysis. (A) The divergence time tree of 13 species. The number of expanded gene families (red) and the number of contracted gene families (blue) are indicated to the right of each species branch. (B) Synonymous substitution rate per site (Ks) distribution for *H. salicifolia, H. gyantsensis, H. rhamnoides, H. tibetana, E. moorcroftii*, and *Z. jujuba*. Two recent WGD events occurred in both *Hippophae* and *Elaeagnus*. (C) Macro-synteny plot of *H. salicifolia, E. mollis*, and *Z. jujuba*. Syntenic comparison between *H. salicifolia* and *Z. jujuba* or between *E. mollis* and *Z. jujuba* revealed a 4:1 ratio that suggests 2 lineage-specific WGDs in Elaeagnaceae.

To investigate the phylogenetic relationships of sea buckthorn, we conducted a gene family analysis of 13 species, including sea buckthorn and other plants from the order Rosales (Supplementary Fig. S5). A phylogenetic tree was constructed using 1,272 single-copy genes from these genomes. Our results (Fig. 2A) indicate that the genera *Hippophae* and *Elaeagnus* diverged approximately 19.89 million years ago (MYA), and there was a notable expansion of gene families preceding their divergence. Within *Hippopha*e, *H. tibetana, H. rhamnoides*, and Fructus Hippophae were grouped in clade 1, whereas *H. salicifolia* and *H. gyantsensis* comprised clade 2. *H. salicifolia* has recently experienced 2 whole-genome duplication (WGD) events (Fig. 2B), which is consistent with the results of previous studies of sea buckthorn [24, 26–29]. Synteny analysis (Fig. 2C) revealed one-to-one homology relationships between chromosomal segments of *Hippophae* and *Elaeagnus* species [24, 25, 30], which indicates that they have a shared history of 2 WGD events. However, the synteny blocks within *Hippophae* were shorter than those in *Elaeagnus* (Supplementary Fig. S6), indicating that the sea buckthorn genome has undergone a higher frequency of chromosomal rearrangements.

### SDR characteristics of *H. gyantsensis* and *H. salicifolia*

**Figure 3:**
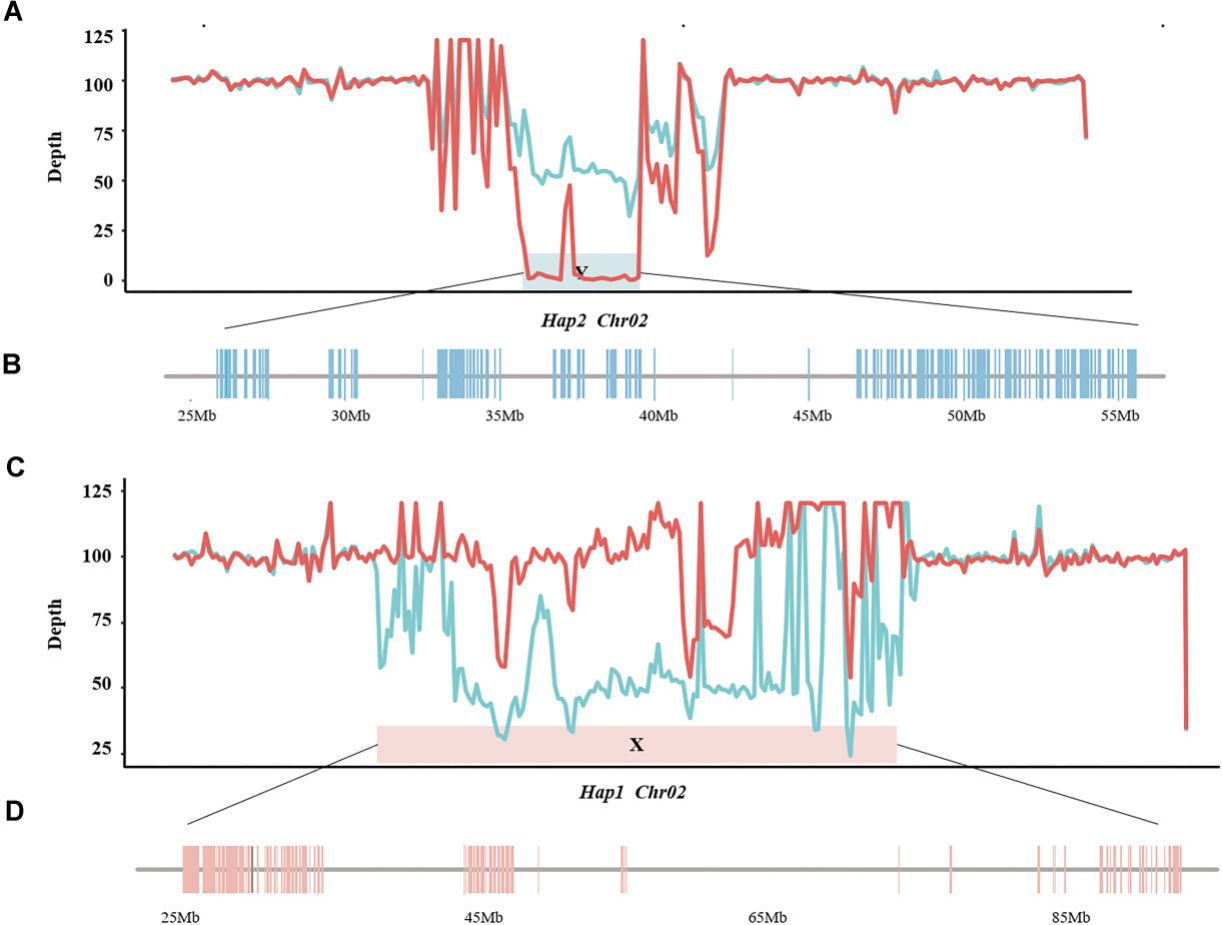
Identification of the *H. salicifolia* 2X- and 2Y-SLRs. (A) The resequencing data of male and female *H. salicifolia* were visualized in the coverage depth of Chr02 of Hap2. The red line represents the depth of female coverage. The blue line represents the depth of male coverage. Y indicates the position of the Y-SLR. (B) Gene distribution in 2Y-SLR. (C) The resequencing data of male and female *H. salicifolia* were visualized in the coverage depth of Chr02 of Hap1. The red line represents the depth of female coverage. The blue line represents the depth of male coverage. X indicates the position of the X-SLR. (D) Gene distribution in 2X-SLR.

To identify sex-linked regions (SLRs) in *H. salicifolia*, we conducted whole-genome resequencing on 2 mixed pools: one consisting of 14 female individuals and the other 14 male individuals. This generated 138.68 Gb and 129.15 Gb of sequencing data for the female and male pools, respectively. Through a comparative analysis of genome coverage depth, we identified a distinct SLR on Chr02 in Hap2 (Fig. 3A, B). Males exhibited a coverage depth of approximately 50% of the genome-wide average, whereas females had negligible coverage in this region; this region was designated as Y-SLR. Using a similar approach for Hap1, we identified an X-SLR (Fig. 3C, D), wherein females displayed average coverage depths, and males showed reduced coverage depths. The Y-SLR in *H. salicifolia* spanned 29.71 Mb and was positioned between 25.85 Mb and 55.55 Mb on Chr02, which comprised 32.88% of the chromosome’s length and encompassed 249 genes. The chromosome was bookended by undifferentiated pseudoautosomal regions (PARs), totaling 60.64 Mb. Conversely, the X-SLR extended 68.02 Mb from 25.83 Mb to 93.86 Mb on Chr02, comprising 52.96% of the chromosome’s length; this region included 334 genes and contained a PAR measuring 60.41 Mb, which was similar to the length of the Y-PAR (Supplementary Fig. S7).

During sex chromosome evolution, genes within SLRs often become degraded and show reduced recombination, which affects the expression and functions of genes [31]. The results of this study provide an in-depth classification and analysis of the genes within the X- and Y-SLRs in the genus *Hippophae* to clarify the evolutionary mechanisms of the sex chromosomes. Genes in the SLRs were classified into 4 categories based on homology and origin: “ancestral” genes, “acquired” genes, duplicated genes, and unique genes. The Y-SLR contained 249 genes, which comprised 99 ancestral, 73 duplicated, 53 acquired, and 24 unique genes; the X-SLR contained 101 ancestral, 82 duplicated, 120 acquired, and 31 unique genes (Supplementary Table S8). The similarity in ancestral gene numbers between the X- and Y-SLRs indicates that there was no significant difference in the degree of degradation of these regions. However, the greater number of genes in X-SLR was attributed to a greater number of insertions of acquired genes. Further analysis indicated that the SLRs of *H. salicifolia* were close to the centromere, which is an area enriched with repetitive sequences. In the Y-SLR, these repetitive sequences comprised 90.58% of the 26.91 Mb region; in the PAR, they comprised 55.32% of this region. The content of repetitive sequences was higher in the X-SLR (94.74%) than in the Y-SLR within the 64.44 Mb region, including 54.86% in the PAR (Supplementary Fig. S8). Although the repetitive sequence content was similar in the PAR regions of both sex chromosomes, the content of repetitive sequences was significantly higher in the X-SLR than in the Y-SLR. These findings indicate that the higher number of acquired genes and repetitive sequences in the X-SLR is likely the main cause of the pronounced morphological divergence between the X and Y chromosomes.

### Evolution of the sex determination region in *H. salicifolia*

**Figure 4:**
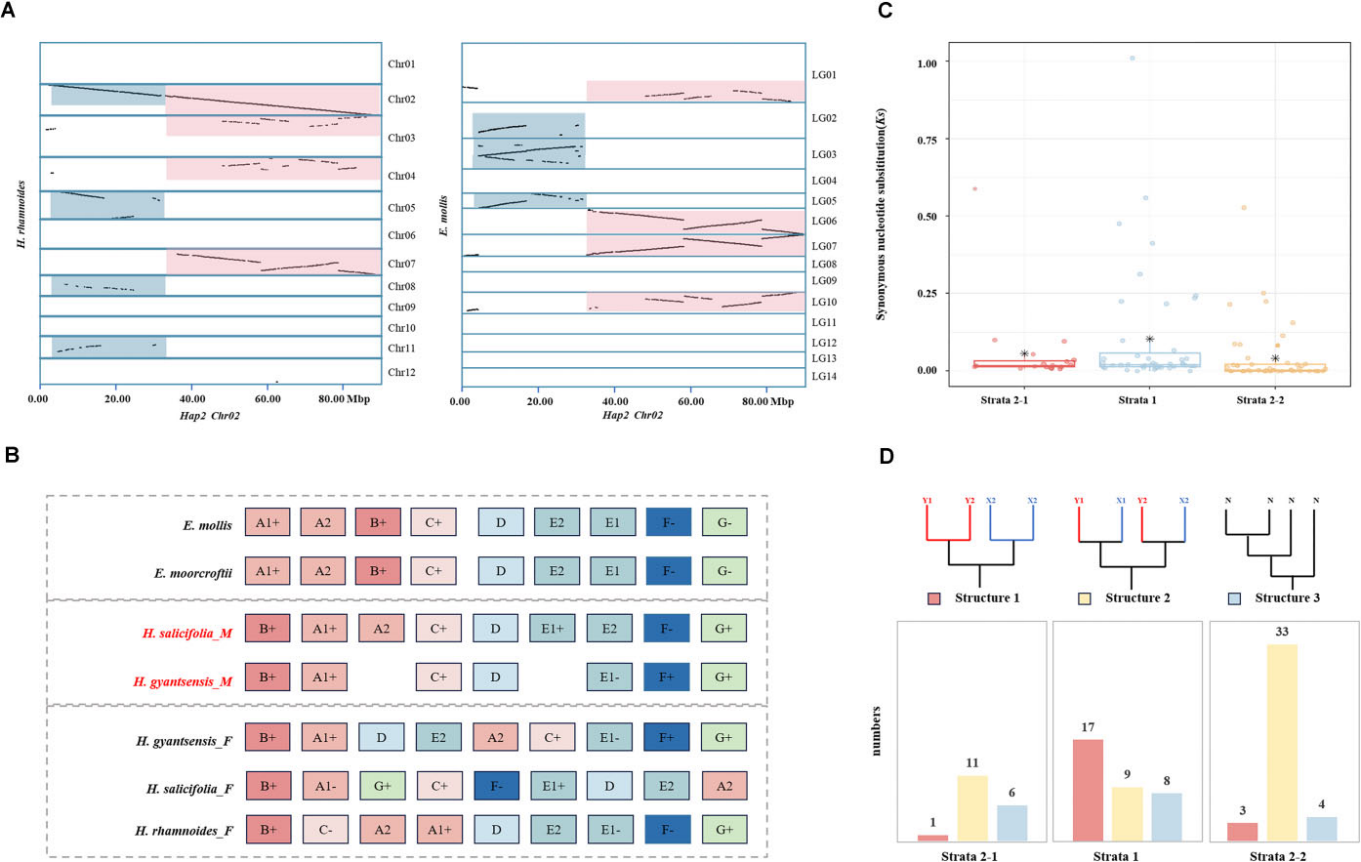
Sex chromosome evolution of *H. salicifolia*. (A) The local syntenic blocks identified between the Hap2 Chr02 and the genomes of *H. rhamnoides* and *E. mollis*. (B) The order and direction of Y-SLR in homologous blocks in the orthologous region of *Hippophae* and *Elaeagnus*. “+” indicates that the internal gene order is consistent, “–” indicates that the internal gene order is opposite, and the absence of a mark suggests that the direction of the internal genes is variable. (C) The distribution of Ks values across different strata, with * indicating the mean value. (D) Phylogenetic structure statistics of single-copy genes within different strata. Structure 1 represents the “ancestral” pattern, structure 2 represents the “recent” pattern, and structure 3 represents the “chaotic” pattern.

To elucidate the origin of the sex chromosomes in the genus *Hippophae*, we compared Chr02 in *H. salicifolia* with the genomes of *H. gyantsensis, H. rhamnoides, Elaeagnus moorcroftii*, and *Elaeagnus mollis* (Fig. 4A) (Supplementary Fig. S9). The synteny analysis identified 2 major collinear blocks adjacent to the SLRs on Chr02 of *H. salicifolia*, which were designated as L and R. Four copies of each of these 2 blocks were present within *Hippophae* and *Elaeagnus*, likely resulting from 2 WGD events shared by both genera. This suggests that these blocks predate the divergence of the 2 genera. On Chr02 of sea buckthorn, the L and R blocks were merged (L–R), a configuration that was not observed on other chromosomes or within the closely related *Elaeagnus*. To determine whether a breakage or fusion event occurred, we used *Ziziphus jujuba* and *Rhamnella rubrinervis* as outgroups for comparison. In both *Z. jujube* and *R. rubrinervis*, the L and R blocks remain separate (Supplementary Fig. S10), indicating that these blocks were likely independent in the ancestral chromosomes of both genera. Integrating these findings with the results of prior synteny analyses (in the comparative genomics section), we infer that following the WGD events, the *Hippophae* genus underwent more frequent chromosomal breakage and fusion events during diploidization. Specifically, a lineage-specific chromosomal fusion event occurred, resulting in the merging of 2 ancestral chromosomes to form Chr02. This fusion might have had a profound effect on the sex determination and reproductive strategies of *Hippophae*.

We performed a microsynteny analysis in which the Y-SLR of *H. salicifolia* was compared with homologous regions in the Y-SLR of other *Hippophae* species and *Elaeagnus* to clarify the evolution of these regions following the formation of sex chromosomes. The collinear regions were categorized into 9 distinct blocks (A–G) through the comparison of chromosomal segment synteny (Supplementary Fig. S11 and Supplementary Table S9). Discrepancies in the positions of blocks A and B between *Elaeagnus* and *Hippophae* were noted (Fig. 4B). However, the ancestral configuration of these blocks remains uncertain because of a lack of systenic information from outgroup comparisons; we hypothesize that *Hippophae* and *Elaeagnus* have structurally diverged. A comparative analysis of the block arrangement and orientation across closely related species has revealed that the Y-SLR in *H. salicifolia* is more structurally conserved than the X-SLR. Specifically, in the X-SLR, blocks A2, G, F, and E1 have been translocated, and block A1 has been inverted. In contrast, only a single translocation involving block E2 was identified in the Y-SLR, while block D is reversed in X-SLR, Y-SLR, or both. We computed the Ks values for homologous gene pairs within these blocks to infer the sequences for the different structural variations (Supplementary Table S10). Block D had the highest Ks value (Ks = 0.1266), followed by block F (Ks = 0.082), block E1 (Ks = 0.0442), block E2 (Ks = 0.0409), block A2 (Ks = 0.03854), block A1 (Ks = 0.01429), and block G (Ks = 0.0037). The elevated Ks value of block D indicates that its structural changes occurred earlier than those of other blocks, and the lower Ks values for A1 and G suggest that their structural changes occurred recently.

Chromosomal rearrangement events are the main drivers of recombination suppression and sex chromosome differentiation [14]. Recombination suppression can promote increased sequence divergence in different regions of the sex chromosomes, forming so-called “evolutionary strata,” which reflect the history and chronological order of structural variations in the evolution of sex chromosomes [32]. The synonymous substitution rate (Ks) is employed as a metric to quantify the degree of sequence divergence between homologous gene pairs.

A higher Ks value generally indicates an earlier divergence event. We analyzed the evolutionary rates of genes within the Y-SLR of *H. salicifolia* by calculating the Ks values of XY homologous gene pairs and mapping these values onto their corresponding evolutionary strata. The distribution of Ks values (Supplementary Table S10) did not exhibit clear clustering of high or low Ks values across specific genomic blocks, a phenomenon potentially attributable to the influence of structural variations on Ks values. Structural variations, such as inversions, may lead to localized increases in Ks values, necessitating a comprehensive consideration of their impact in our analysis. Accordingly, we adjusted the Ks values for certain blocks affected by structural variations. Nevertheless, it is highly probable that block D belongs to an ancient stratum. Furthermore, considering that the sex chromosomes of *Hippophae* originated from the fusion of 2 chromosomes, collinearity alignment results identified the region between blocks C and D as the fusion point. Chromosomal fusion, by juxtaposing previously independent gene regions, promotes the formation of linkage groups associated with sex differentiation, thereby potentially driving the evolution of sex determination.

Thus, we propose that both blocks D and C likely belong to an ancient stratum. The abundance of repetitive sequences between blocks C and D may result from recombination suppression during sex chromosome evolution, and their accumulation likely contributes to the stability of the sex-determining region, a hallmark of sex chromosome evolution. Based on the distribution of Ks values, the influence of structural variations, chromosomal fusion events, and the distribution of repetitive sequences, we divided the Y-SLR into 2 putative evolutionary strata (Supplementary Fig. S12). Stratum 1 (including blocks C and D) was located from 33.13 to 49.72 Mb, and the average Ks value was 0.1041 for the 43 homologous gene pairs in this region. Stratum 2–1 (including blocks A and B) was located from 25.88 to 33.08 Mb and contained 19 homologous gene pairs with an average Ks value of 0.0574. Stratum 2–2 (including blocks E, F, and G) was located from 50.19 to 55.61 Mb and contained 48 homologous gene pairs with an average Ks value of 0.0418 (Fig. 4C).

Genes from different evolutionary strata exhibit distinct phylogenetic patterns. In dioecious genera, where all species are dioecious, sexual differentiation likely predates species divergence. In such cases, genes within the ancient strata of the SDR would have diverged prior to species differentiation. Consequently, homologous genes originating from the same gametophyte tend to cluster together. In contrast, genes from more recent strata may have diverged after species differentiation, leading to a tendency for homologous genes from the same species to cluster together [7, 17]. To further validate the proposed strata, we constructed a phylogenetic tree for single-copy genes within the SLRs and analyzed its topology. In stratum 1, 17 of 34 single-copy genes exhibited an ancient origin, while in strata 2–1 and 2–2, only 1 and 3 genes, respectively, exhibited an ancient origin (Fig. 4D). These results indicate that stratum 1 represents a more ancient partition of the sex chromosomes in *H. salicifolia*, and strata 2–1 and 2–2 are more recent. Integrating the Ks analysis of structural variations, we hypothesize that the inversion event in block D may have facilitated the differentiation of stratum 1 by suppressing recombination, thereby accelerating the functional specialization of the sex chromosomes. Conversely, the inversions in blocks F, E, and A2 are closely associated with the formation of stratum 2. These findings suggest that chromosomal structural variations between X- and Y-SLRs might have played a key role in promoting the spread of recombination suppression and the evolution of sex chromosomes.

### Sea buckthorn sex determination candidate genes

**Figure 5:**
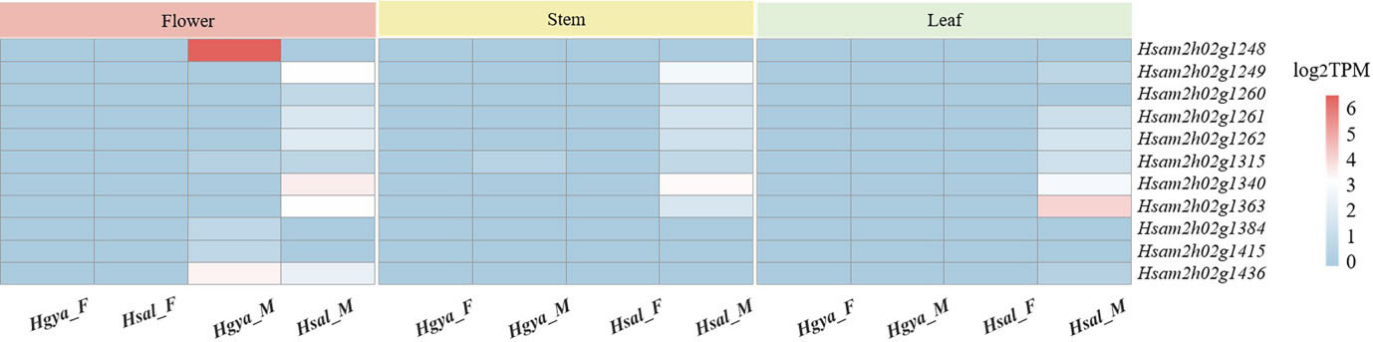
Within the Y-SLR of *H. salicifolia*, there are male-specific expression genes in both *H. salicifolia* and *H. gyantsensis*. “F” represents female, and “M” represents male.

To identify candidate genes involved in sex determination in sea buckthorn, we analyzed the transcriptional profiles of male and female mixed flower buds at various developmental stages of *H. gyantsensis* and *H. salicifolia* (Supplementary Table S11). We utilized transcriptome data, compared these data with the Y-SLR of *H. salicifolia*, and calculated the transcripts per million (TPM) values of the transcripts (Supplementary Table S12). This analysis identified 11 genes that are specifically expressed in males (Fig. 5). We conducted further analysis on the 11 male-specific expressed genes and found that 5 of them lacked homologous counterparts in male *H. gyantsensis*, leading to their preliminary exclusion.

As previously mentioned, if all species within a genus are dioecious, sexual differentiation likely predates species divergence. Consequently, sex-determining genes typically exhibit phylogenetic similarities with genes from ancient evolutionary strata, meaning that homologous genes originating from the same gametophyte tend to cluster together. This is because the divergence of sex-determining genes between males and females occurred before species differentiation [7, 17]. Therefore, we analyzed the phylogenetic tree topology of the remaining 6 genes. The results revealed that 2 of these genes displayed a topology inconsistent with our expectations (Supplementary Fig. S13), showing a clustering pattern where homologous genes from males and females of the same species grouped together. Three genes (*Hsam2h02g1262, Hsam2h02g1315*, and *Hsam2h02g1436*) did not have corresponding homologous genes identified within the X-SLR of *H. salicifolia* and *H. gyantsensis* (Supplementary Fig. S14). However, homologous genes were found on autosomes, suggesting that these genes were likely duplicated from autosomal regions and inserted into the Y-SLR, where they may function as male-specific genes potentially involved in sea buckthorn sex determination. The gene tree structure of *Hsam2h02g1248* largely aligned with our expectations (Supplementary Fig. S15), with homologous genes from the Y-SLR of different sea buckthorn species clustering together and those from the X-SLR forming a separate cluster. Although the ideal phylogenetic tree structure would have shown *Hsam2h02g1248* and *Hgyam02g1367* grouping together rather than exhibiting a sequential divergence pattern, minor variations can easily influence gene tree topology. Thus, *Hsam2h02g1248* remains a strong candidate for a sex-determining gene. Among the 4 selected genes, 3 genes (*Hsam2h02g1248, Hsam2h02g1262*, and *Hsam2h02g1314*) are located within the ancient evolutionary stratum we previously identified (Supplementary Fig. S16). These genes are considered the most promising candidates for sea buckthorn sex determination.

The gene *Hsam2h02g1248*, a homolog of *FAR*, was highly and specifically expressed (TPM > 100) during the male flower bud development phase in *H. gyantsensis*, and it was minimally expressed in stems or leaves. This expression profile resembles that of *AtFAR2* in *Arabidopsis thaliana*, which is thought to be involved in male sterility [33]. Furthermore, the homologous gene of *Hsam2h02g1248* within the X-SLRs has undergone fragmentation (Supplementary Fig. 17). By comparing resequencing data from male and female *H. salicifolia* (Supplementary Fig. 18), we identified an insertion in the X-SLR homologous gene, which likely caused its disruption. Additionally, compared to *Hsam2h02g1248*, the X-SLR homologous gene lacks several sequences, potentially leading to the loss of its original function. The role of this gene and its pattern of expression are intimately linked to the masculinization of sea buckthorn, which makes it a male-stimulating factor (M). *Hsam2h02g1315* and *Hsam2h02g1262* are male-specific and lack X-SLR homologs in sea buckthorn (Supplementary Figs. S19 and S20). *Hsam2h02g1315* is a homologous gene of *Flowering Locus D* (*FD*) , and the homologous gene of *Hsam2h02g1262* in *A. thaliana* is *AtRRP44A*. Both of them are closely related to flower development [34, 35]. One of these 2 genes might be a female suppressor factor in sea buckthorn, yet additional experiments are needed to verify this possibility.

## Discussion

Sexual differentiation is a critically important component of the reproductive strategy of members of the genus *Hippophae*, and studies of sexual differentiation can provide insights into the physiological and molecular mechanisms by which sea buckthorn adapts to different environments, which have implications for ongoing efforts to genetically improve sea buckthorn and regulate the sex determination process. However, the lack of complete genomic information for male sea buckthorn species limits studies of sexual differentiation. The absence of male genomic data has also impeded studies of the molecular mechanisms of sex determination in sea buckthorn, the gene expression regulatory networks during sexual differentiation, and the genetic basis of sex-specific traits. A recent study of *H. tibetana* has indicated that its sex chromosomes are located on Chr02; however, the SDR interval was not determined in this study [26]. We sequenced the genomes of male *H. gyantsensis* and both male and female *H. salicifolia*; we obtained 4 sets of genomic data (including 2 haplotype datasets for male *H. salicifolia*).

*H. salicifolia* and *H. gyantsensis* have recently undergone 2 WGD events, and this finding is consistent with that observed in other species within the family Elaeagnaceae [24, 25]. The recent WGD events are specific to Elaeagnaceae. Chromosomal breakage and fusion events following WGD events have been more common in *Hippophae* than in *Elaeagnus*, which has also experienced 2 WGD events [24, 25]. Previous studies have indicated that chromosomal breakage and fusion play a major role in the formation of sex chromosomes [36]. These events can lead to substantial changes in genome structure, which affects gene expression and function and can sometimes even result in the formation of new species [37]. In our study, the sex chromosomes of both *H. salicifolia* and *H. gyantsensis* were located on chromosome Chr02. Chr02 in *Hippophae* species was derived from the fusion of 2 distinct ancestral chromosomes (A and B). One end of the fusion fragment of chromosome A was located near the centromeric region of the chromosome. When it fused with chromosome B, it introduced a large number of repetitive sequences, which facilitated the subsequent cessation of recombination in this region to form the SLR [38]. Sex chromosomes are known to have independently evolved through similar mechanisms in other lineages, such as the SLRs of papaya and kiwifruit, which have evolved from the pericentromeric regions [39–41]. Throughout the evolutionary process, WGD events, chromosomal rearrangements, and the lack of recombination around the centromeric regions have facilitated the origin of sex chromosomes in *Hippophae* species.

Plant sex chromosomes are derived from a pair of autosomes, and recombination suppression is essential for the evolution of sex chromosomes. Chromosomal rearrangements, heterochromatinization, the accumulation of repetitive sequences, and DNA methylation can all lead to recombination suppression [42, 43], and chromosomal rearrangements are the main cause of recombination suppression [2, 44]. In papaya, 2 large inversions on the Y chromosome have promoted the differentiation of XY, which is the main cause of recombination suppression in the XY chromosomes [40]. In our study, the evolution of the sex chromosomes in *H. salicifolia* appears to be related to the frequent chromosomal rearrangements in the X- and Y-SLRs. Analysis of the arrangement and orientation of homologous blocks within the SLRs revealed that the Y-SLR structure of *H. salicifolia* was more conserved, with multiple blocks in the X-SLR undergoing chromosomal inversions or translocations and 1 block in the Y-SLR undergoing translocation. These structural variations are closely related to the spread of recombination suppression.

Based on the structural variations and the Ks values of the evolutionary strata, we simulated the evolution of the sex chromosomes in *H. salicifolia*: the oldest structural variations occurred over a narrow range near the centromere, which promoted the spread of recombination suppression and the formation of ancient strata. Subsequently, large-scale structural variations occurred around the ancient strata, which promoted the spread of recombination suppression and the formation of new strata. X-linked structural variations do not frequently drive the evolution of sex chromosomes; rearrangements in the Y-SLR have been identified in most sex chromosomes studied to date, such as inversions in papaya and willows in the Y chromosome [45, 46]. However, recent studies of *Silene latifolia* have shown that recombination suppression may stem from an inversion in the X-SLR [47]. Many current theories of sex chromosome evolution are based on the premise of Y-linked inversions [48]; however, the results of our study indicate that X-linked structural variations might be selected and fixed to suppress recombination between XY chromosomes.

Sex-determining genes are the main drivers of reproductive organ differentiation in dioecious plants. However, these genes have been isolated and identified in only a handful of species, including kiwifruit, persimmon, asparagus, and poplar [18, 19, 48, 49]. We were unable to identify homologs of these known sex-determining genes in the SDRs of sea buckthorn, indicating that a unique mechanism promotes maleness and inhibits femaleness in this genus. Typically, sex-determining genes are specifically expressed in male flowers and exhibit a phylogenetic pattern in which homologs from various species cluster together. Within the sea buckthorn’s MSY region, we identified 3 genes that appear consistent with these criteria. Notably, *Hsam2h02g1248* is a candidate male-promoting factor, and its *Arabidopsis* homolog, *FAR2*, is a known male sterility factor [33]. This gene also shares significant evolutionary similarity with the kiwifruit sex-determining gene [19], *FrBy*, which is present during the andromonoecious phase and is differentially maintained in males after sexual differentiation. Our findings are consistent with this pattern, with *Hsam2h02g1248* persisting in males and losing functionality in females after sexual differentiation. Under normal circumstances, the sex determination region has 2 genes that jointly determine sexual differentiation. Based on the characteristics of SLRs, we speculate that sea buckthorn should belong to a species with dual-gene sex determination—that is, in addition to a male-promoting factor, there is also a female-suppressing factor. *Hsam2h02g1315* and *Hsam2h02g1262* are our inferred female-suppressing candidate genes. These 2 genes lack homologs in the X-SLR but have homologs on the autosomes, suggesting they may have originated from autosomal gene duplication. Gene duplication plays a key role in the formation of female-inhibiting factors; for example, the female-inhibiting factor *SyGl* in kiwifruit is derived from an autosomal gene duplication that changes its expression pattern to inhibit carpel development [18]. The sex-determining genes *FERR-R* and *ARR17* in poplar species also arise from duplication [48, 50]. *Hsam2h02g1315* and *Hsam2h02g1262* are both related to flower development. Additional experiments are needed to determine whether they have acquired new functions after duplication.

## Methods

### Sample collection

All samples required for this study of *H. gyantsensis* were obtained from Jiangzi County, Shigatse City, Tibet Autonomous Region, China. All samples of *H. salicifolia* were collected from Gyirong County, Tibet Autonomous Region, China. For genomic sequencing, we collected leaf samples from 1 male *H. gyantsensis*, 1 female *H. gyantsensis*, and 1 male *H. salicifolia*. Additionally, to assist with genome annotation, we collected leaf and stem samples from 3 male and 3 female individuals of *H. salicifolia* and 2 male and 2 female individuals of *H. gyantsensis* for transcriptome sequencing, with 2 replicates for each sample. To identify the SLRs in *H. gyantsensis* and *H. salicifolia*, we performed whole-genome resequencing on 2 mixed pools: 1 of 14 female *H. salicifolia* individuals and 1 of 14 male *H. salicifolia* individuals. Additionally, to elucidate the differential gene expression patterns between male and female individuals, we collected mixed floral bud samples from both *H. gyantsensis* and *H. salicifolia* for transcriptome sequencing analysis.

### Genome sequencing

Genomic DNA samples for short-read sequencing were extracted from the leaves using the CTAB method [51]. Libraries were constructed using the MGIEasy Universal DNA Library Prep Kit V1.0 (CAT#1000005250, MGI) per the standard protocol. The qualified libraries were sequenced on the DNBSEQ-T7RS platform. Total RNA was extracted by grinding tissue using the CTAB-LiCl method (Plant) on dry ice and processed per the manufacturer’s protocol. After meeting the quality control standards, an appropriate amount of total RNA was used to construct a library for sequencing using the DNBSEQ-T7RS platform (RRID:SCR_017981).

ONT regular DNA was extracted using the Grandomics Genomic DNA Kit per the manufacturer’s protocol. DNA samples were accurately quantified using a Qubit 3.0 Fluorometer (Invitrogen). Long, size-selected DNA fragments were then extracted using the PippinHT system (Sage Science). DNA was repaired and adapters were attached to the ends using an SQK-LSK110 kit. The concentrations of library fragments were quantified using a Qubit 3.0 Fluorometer. The DNA library was loaded into the primed Nanopore PromethION sequencer (Oxford Nanopore Technologies) flow cell. After DNA extraction, the SMRTbell target size library was constructed using the 15-kb preparation solution according to PacBio’s standard protocol (Pacific Biosciences) for HiFi sequencing. Sequencing was conducted on the Grandomics PacBio Sequel II instrument. Genomic DNA was extracted for the Hi-C library from leaves. Next, we constructed the Hi-C library and obtained sequencing data using the DNBSEQ-T7RS platform.

### Genome size assessment and genome assembly

The *k*-mer (*k* = 21) frequency table of clean NGS reads was generated using the Jellyfish (RRID:SCR_005491) v2.3.0 [52] program, and the genome size and heterozygosity of the 3 sea buckthorn species were assessed using GenomeScope (RRID:SCR_017014) v2.0 [53]. NextDenovo (RRID:SCR_025033) v2.4.0 was used to generate the preliminary assembly from the ONT clean sequencing data for male *H. gyantsensis* and female *H. salicifolia*. NextPolish (RRID:SCR_025232) v1.3.1 [54] was used to perform 3 rounds of ONT and NGS iterative error correction on the initially assembled genomes. For male *H. salicifolia* sequenced using HiFi sequencing, raw BAM files obtained from sequencing were converted into gz format using SAMTOOLS (RRID:SCR_002105) v1.18 [55], and Hifiasm (RRID:SCR_021069) v0.19.9-r616 [56] was used to assemble contigs by integrating HiFi and HiC reads. After obtaining the preliminary assemblies of the 3 sea buckthorn genomes, Minimap2 (RRID:SCR_018550) v2.28-r1209 [57] was used to compare the third-generation sequencing data with the genome, and the read coverage depth and coverage breadth for each contig were calculated. Redundant sequences and small genome sequences were identified and removed based on read coverage using Purge_Dups (RRID:SCR_021173) v1.2.5 [58] software. Cleaned Hi-C reads were input into Juicer (RRID:SCR_017226) v1.6 software [59] for ALLHiC (RRID:SCR_022750) v210623 [60] chromosome construction. Manual adjustments for inversions and shifts in the assembly were made using Juicebox (RRID:SCR_021172) [61] to obtain 3 chromosome-level sea buckthorn genomes. The final Hi-C contact map was visualized using HiCExplorer (RRID:SCR_022111) v3.7.3 [62]. To improve the quality of the chromosome assembly, the male *H. salicifolia* genome was corrected using NextPolish2 [63], using clean HiFi and NGS data as input. The completeness of the genome assembly was evaluated using BUSCO (RRID:SCR_015008) v5.7.0 [64] and the embryophyta-odb10 dataset.

### Genome annotation

Repetitive sequences of the 3 sea buckthorn genomes were annotated using the Extensive de novo TE Annotator (EDTA (RRID:SCR_022063) v2.1.2 [65]). After obtaining the EDTA-annotated transposable element (TE) library, TEsorter v1.4.6 [66] was used to reclassify the “LTR-unknown,” followed by deepTE [67] classification for the “TEsorter-unknown.” Finally, the 3 obtained TE databases were merged, and the repetitive sequences were masked using RepeatMasker (RRID:SCR_012954) v4.1.2-p1 [68].

Homology-based protein alignment, *de novo* prediction, and transcriptome prediction were used for gene structure annotation. HISAT2 (RRID:SCR_015530) v2.2.1 [69] was used to align clean reads from the transcriptome to the genome, and the resulting alignment files were used as input for Braker3 [70, 71]. GeneMark-ET [72] was used for gene structure identification, and Augustus was used for model training to obtain high-confidence gene structures based on the transcript and *de novo* prediction results. *H. rhamnoides* protein sequences were input into Braker3, and GeneMark-EP [73] combined with ProtHint was used for sequence alignment. Augustus was used for model training to obtain high-confidence gene structures based on the known proteins and *de novo* prediction results. The results obtained via Braker3 were then integrated using TSEBRA [74] (default parameters); this was followed by quality filtering and normalization using scripts from the MAKER (RRID:SCR_005309) package v3.01.04 [75], which ultimately generated a gff annotation file that describes genetic structural information. TBtools (RRID:SCR_023018) [76] was used to extract the longest transcript, gffread (RRID:SCR_018965) v0.12.8 [77] was used to extract the corresponding cds sequence, and then SeqKit (RRID:SCR_018926) [78] was used to translate it into a protein sequence. BUSCO (RRID:SCR_015008) v5.7.0 [64] was used to assess the integrated annotation proteins.

InterProScan (RRID:SCR_005829) v5 [79] was used to conduct a search of the annotated genes with the parameters set to “-appl Pfam, CDD.” The predicted genes were functionally annotated by scanning the NCBI nr, TrEMBL, and Swiss-Prot [80] databases using DIAMOND (RRID:SCR_009457) v2.0.15 [81] with default parameters. eggNOG-mapper (RRID:SCR_021165) [82] with default parameters was used to obtain KEGG (RRID:SCR_012773) [83] and GO (RRID:SCR_017505) [84] annotations.

### Comparative genomics analysis

Protein sequences of *H. rhamnoides, H. salicifolia, H. gyantsensis*, Fructus Hippophae, *H. tibetana, E. moorcroftii, E. mollis, Ziziphus jujuba, Fragaria daltoniana, Vitis vinifera, Morus notabilis, Cannabis sativa*, and *Rhamnella rubrinervis* were selected for gene family analysis. OrthoFinder (RRID:SCR_017118) v2.5.5 [85] was used to identify orthologous groups with the parameters set as “-M msa,” and single-copy genes were used to construct a phylogenetic tree. The SeqKit (RRID:SCR_018926) [78] tool was used to extract the coding sequences of single-copy orthologous genes, and they were aligned using MUSCLE (RRID:SCR_011812) v3.8.31 [86]. The SeqKit (RRID:SCR_018926) [78] tool was used to concatenate the sequences into supergenes, which were then trimmed using trimAl (RRID:SCR_017334) v1.4 [87] (-gt 0.6 -cons 60). The trimmed sequences were used to construct a phylogenetic tree using the maximum likelihood method in RAxML (RRID:SCR_006086) v1.1.0 software [88]. Three fossil calibration time points were obtained from the TimeTree (RRID:SCR_021162) database [89]: *V. vinifera* and *Z. jujuba* (109.8 to 122.4 MYA), *Z. jujuba* and *C. sativa* (68.5 to 85.2 MYA), and *C. sativa* and *M. notabilis* (48.9 to 70.9 MYA). The MCMCtree program in the PAML (RRID:SCR_014932) v.4.10.0 software package [90] was used to estimate the divergence times of each node in the phylogenetic tree obtained in the previous step. The phylogenetic tree with divergence times and the sorted gene family results (i.e., gene families with significant differences in copy number were removed) were used to construct a phylogenetic tree containing information on gene family expansions and contractions using the CAFE (RRID:SCR_005983) V5 [91] program.

We used WGDI v0.6.5 [92] to detect whole-genome duplication events. DIAMOND (RRID:SCR_009457) v2.0.15 [81] was used to identify homologous genes with an e-value that did not exceed 1e-5. WGDI was used to identify collinear genes with the parameter “-icl.” Next, the “ks” parameter in WGDI was modified to calculate Ks values, and the “bi” and “c” parameters were modified to filter the collinear results. Finally, the “kp” parameter was modified to calculate the Ks peak, and the “kf” parameter was used to fit and visualize the results. JCVI (RRID:SCR_021641) [93] (MCscan Python version) was used for whole-genome collinearity analysis between *Hippophae* and *Elaeagnus* plants.

### Sex determination interval and characteristics

The *H. salicifolia* resequencing data were compared with the Hap1 and Hap2 reference genomes using BWA-MEM2 (RRID:SCR_022192) v2.2.1 [94] software. Based on the comparison results, a window was established within an interval of 50 kb, with a sliding window of 5 kb for stepwise progression. BEDTools (RRID:SCR_006646) v2.30.0 [95] was used to calculate the coverage depth of each window, and the R package ggplot2 (RRID:SCR_014601) [96] was used to visualize the results. The chromosomes and intervals of the SDR were located assuming that male-specific regions have no female read coverage, and male read coverage was half that of other regions. After obtaining the initial SLRs, Integrative Genomics Viewer (IGV) (RRID:SCR_011793) software [97] was used to view the precise SLR range by comparing different regions.

We mainly used JCVI (RRID:SCR_021641) [93] and BLASTP (RRID:SCR_001010) v2.14.0+ [98] to analyze the origin of genes within the X- and Y-SLRs of *H. salicifolia*. To begin, we conducted a collinearity analysis by comparing the X-SLR and Y-SLR regions with each other and with 2 species of the genus *Elaeagnus*. Given that both *Hippophae* and *Elaeagnus* belong to the family Elaeagnaceae and diverged after experiencing 2 WGD events, we designated the copies of Chr02 in *Elaeagnus* and *Hippophae* as the putative ancestral chromosome. Genes within the SLRs that exhibited homology with those in the putative ancestral chromosomes of *Hippophae* and *Elaeagnus* were classified as “ancestral” genes. For the remaining genes, we performed a BLASTP (RRID:SCR_001010) v2.14.0+ [98] search to identify homologous sequences. Genes with homologous sequences on autosomes (nonputative ancestral chromosomes) were categorized as “acquired” genes, which may have been gained through duplication and insertion events of autosomal genes. Genes without homologous sequences were classified as “specific” genes, with those exclusively present in the X-SLR termed X-SLR-specific genes and those exclusively present in the Y-SLR termed Y-SLR-specific genes. Finally, genes with duplicated copies within the SLRs were categorized as “duplicated” genes. Specifically, if 2 duplicated copies were identified among the “ancestral” genes: one was assigned to the “duplicated” gene category, and the same classification method was applied to other gene categories.

### Sex chromosome evolution of *H. salicifolia* and *H. gyantsensis*

For evolutionary analysis, we first used JCVI (RRID:SCR_021641) [93] to analyze the collinearity Chr02 of *H. salicifolia* with *H. gyantsensis, H. rhamnoides, E. moorcroftii, E. mollis, R. rubrinervis*, and *Z. jujuba*. JCVI (RRID:SCR_021641) [93] was also used to analyze the microsynteny of the SLR regions in *H. salicifolia* with *H. gyantsensis, H. rhamnoides*, Fructus Hippophae, *E. moorcroftii*, and *E. mollis*. Based on the identified homologous blocks, we analyzed block rearrangements in *Hippophae* and *Elaeagnus* plants. To analyze the substitution rates of X- and Y-linked genes, we created pairwise alignments including X-linked and Y-linked homologous genes and then used ParaAT.pl v2.0 [99] and KaKs_Caculater v3.0 [100] to calculate synonymous and nonsynonymous substitution rates. Then, based on the block coordinate, we calculated the average Ks value of homologous genes within each block. Different phylogenetic patterns were observed for genes in the “ancient” and “recent” regions of the sex chromosomes. Therefore, OrthoFinder (RRID:SCR_017118) v2.5.5 [85] was used to identify single-copy orthologous genes within the sea buckthorn SLRs, and MAFFT (RRID:SCR_011811) v7.520 [101] was used to align the sequences in each set of single-copy orthologs. We also conducted searches of the corresponding chromosomes of *E. moorcroftii* and *E. mollis* using BLASTP [98] to identify corresponding homologous genes. Finally, we used IQ-TREE2 v2.2.0 [102] to generate the gene trees with 1,000 bootstrap replicates.

### Screening of sex-determining candidate genes

To identify candidate genes involved in sex determination, we first conducted an analysis of differentially expressed genes between males and females. Fastp (RRID:SCR_016962) [103] software was used to filter the transcriptome data of male and female flower buds of *H. gyantsensis* and *H. salicifolia*, and STAR (RRID:SCR_004463) [104] was used to align them with the male *H. salicifolia* Hap2 genome; RSEM (RRID:SCR_000262) [105] software was used to calculate expression levels. Finally, we utilized the R package ggplot2 [96] to generate a heatmap by plotting the logarithm-transformed TPM values. Using the same method, we also calculated the expression levels of the stem and leaf transcriptome data from both female and male individuals of *H. gyantsensis* and *H. salicifolia* (the transcriptome data used were the same as those used for assisting genome annotation, including the published data for the stem and leaf transcriptome of the female *H. gyantsensis* [PRJNA997223]) [28].

After identifying genes specifically expressed in males, we further screened them based on their phylogenetic positions relative to those of homologous genes. First, we extracted the protein sequences of genes specifically expressed in males in the SDR region of *H. salicifolia* as queries and used data from *H. rhamnoides, H. tibetana*, male *H. gyantsensis*, female *H. gyantsensis*, male *H. salicifolia*, female *H. salicifolia*, and Fructus Hippophae as protein databases; *E. moorcroftii* and *E. mollis* were used as outgroups in homologous gene searches. Based on the obtained BLASTP (RRID:SCR_001010) v2.14.0+ [98] alignment results, we retained sequences with identity greater than 70 and performed sequence alignment using MAFFT (RRID:SCR_011811) v7.520 [101] software. Gene trees were constructed using IQ-TREE2 v2.2.0 [102], and Figtree was used to visualize the trees.

## Supplementary Material

giaf046_Supplemental_Files

giaf046_Authors_Response_To_Reviewer_Comments_original_submission

giaf046_GIGA-D-24-00421_original_submission

giaf046_GIGA-D-24-00421_Revision_1

giaf046_Reviewer_1_Report_original_submission Zhiqiang Wu -- 10/28/2024

giaf046_Reviewer_1_Report_Revision_1 Zhiqiang Wu -- 2/26/2025

giaf046_Reviewer_2_Report_original_submission Alan E. Yocca -- 11/26/2024

## Data Availability

The sequencing data that support the findings of this study are openly available in the NCBI Sequence Read Archive (SRA) under BioProject accession number PRJNA1166656. The resequencing data for female and male *Hippophae salicifolia* can be found in the National Genomics Data Center (NGDC) under project accession number PRJCA036349. The genome assembly and annotation data of the 3 sea buckthorns have been deposited in FigShare [106]. All supporting data and materials are available in the *GigaScience* database, GigaDB [107], with separate datasets for male *H. gyantsensis* [108] and female *H. salicifolia* [109].

## Additional Files

**Supplementary Fig. S1**. Distribution of *k*-mer (*k* = 21) frequency in sequencing reads of the 3 sea buckthorn plants. (a) Distribution of *k*-mer frequency for male *H. salicifolia* from Illumina sequencing. (b) Distribution of *k*-mer frequency for female *H. salicifolia* from Illumina sequencing. (c) Distribution of *k*-mer frequency for male *H. gyantsensis* from Illumina sequencing.

**Supplementary Fig. S2**. Hi-C heatmap of chromosomes for male *H. gyantsensis* (a), female *H. salicifolia* (b), Hap1 (c), and Hap2 (d) of male *H. salicifolia*.

**Supplementary Fig. S3**. Circos plot of the genomic landscape of male *H. gyantsensis*. (a) Pseudochromosome. (b) Gene density. (c) Repeat sequences density. (d) Ty3 density. (e) Copia density. (f) GC content.

**Supplementary Fig. S4**. Circos plot of the genomic landscape of female *H. salifolia*. (a) Pseudochromosome. (b) Gene density. (c) Repeat sequences density. (d) Ty3 density. (e) Copia density. (f) GC content.

**Supplementary Fig. S5**. Statistics of orthogroups in different plants defined by OrthoFinder.

**Supplementary Fig. S6**. The synteny dot plot within the genome of *E. mollis* and *H. salicifolia*. (a) *E. mollis*. (b) *H. salicifolia*.

**Supplementary Fig. S7**. Hi-C schematic diagram of the sex chromosomes in *H. salicifolia*. (a) Hap1 Chr02. (b) Hap2 Chr02. (c) The schematic of Chr02 in Hap1, with the yellow region highlighting the X-SLR. (d) The schematic of the sex chromosome in Hap2, where the yellow region denotes the Y-SLR.

**Supplementary Fig. S8**. Density plot of repetitive sequences on Chr02 for Hap1 (a) and Hap2 (b). Pink dots represent the density of pseudoautosomal repeats, and blue dots represent the density of repeats in sex-linked regions.

**Supplementary Fig. S9**. Synteny relationship between Chr02 of Hap2 and its closely related species. (a) Syntenic point map of Hap2 Chr02 and whole genome of *H. gyantsensis*. (b) Syntenic point map of Hap2 Chr02 and whole genome of *E. moorcroftii*.

**Supplementary Fig. S10**. Synteny relationship between chromosome 2 of Hap2 and its closely related species. (a) Synteny relationship between Chr02 of Hap2 and *Z. jujuba*. (b) Synteny relationship between Chr02 of Hap2 and *R. rubrinervis*.

**Supplementary Fig. S11**. Microsynteny of homologous blocks between the Y-SLR of Hap2 and its closely related species. (a) *E. mollis*. (b) *E. moorcroftii*. (c) *H. rhamnoides*. (d) Fructus Hippophae. (e) Female *H. gyantsensis*. (f) Male *H. gyantsensis*. (g) Hap1. The yellow collinearity blocks correspond to the pseudoautosomal regions (PARs) of the chromosomes.

**Supplementary Fig. S12**. The Ks values of XY homologous gene pairs within the Y-SLR are mapped to the positional coordinates of the Y chromosome. Blue represents gene pairs within stratum 1, red represents gene pairs within stratum 2–1, and yellow represents gene pairs within stratum 2–2.

**Supplementary Fig. S13**. Among the 11 male specifically expressed genes, the phylogenetic trees of 2 genes are not consistent with the expected phylogenetic structure. 2Y-SLR represents the genes within the Y-SLR, and 2X-SLR represents the genes within the X-SLR. Furthermore, the genes within the Y-SLR are marked in red. (a) *Hsam2h02g1340*. (b) *Hsam2h02g1384*.

**Supplementary Fig. S14**. Among the 11 male specifically expressed genes, 3 are male-specific genes that lack corresponding homologous genes within the X-SLR. The genes within the Y-SLR are marked in red, and the genes within the X-SLR are marked in blue. (a) *Hsam2h02g1262*. (b) *Hsam2h02g1315*. (c) *Hsam2h02g1436*.

**Supplementary Fig. S15**. Gene tree constructed by *Hsam2h02g1248* and its homologous genes. The genes within the Y-SLR are marked in red, and the genes within the X-SLR are marked in blue.

**Supplementary Fig. S16**. The distribution of the 4 candidate genes within the SLR; 3 genes are located in the presumed ancient strata, and 1 gene (*Hsam02g1436*) is close to the PAR region.

**Supplementary Fig. S17**. The sequence alignment of *Hsam2h02g1248* with its homologous genes in the female- and male-specific linkage regions of *H. salicifolia* and *H. gyantsensis* reveals that gene fragmentation has occurred in the homologous genes of females.

**Supplementary Fig. S18**. Alignments of the resequencing reads from male and female individuals to the genes. (a) *Hsam2h02g1248*. (b) The homologous genes of *Hsam2h02g1248* in X-SLR were *Hsam1h02g1337, Hsam1h02g1338*, and *Hsam1h02g1339*.

**Supplementary Fig. S19**. Alignments of the resequencing reads from male and female individuals to the gene *Hsam2h02g1262*.

**Supplementary Fig. S20**. Alignments of the resequencing reads from male and female individuals to the gene *Hsam2h02g1315*.

**Supplementary Table S1**. Sequencing statistics of male *H. gyantsensis*, female *H. salicifolia*, and male *H. salicifolia*.

**Supplementary Table S2**. Statistics of Hi-C read mapping for male *H. gyantsensis*, female *H. salicifolia*, and male *H. salicifolia*.

**Supplementary Table S3**. Summary of male and female sea buckthorn genome assemblies.

**Supplementary Table S4**. Quality assessment of the completeness of female and male genome assemblies using the BUSCO tool.

**Supplementary Table S5**. Repeat sequence annotation statistics.

**Supplementary Table S6**. Functional annotation of predicted protein-coding genes of the male and female sea buckthorn genomes.

**Supplementary Table S7**. Quality assessment of the completeness of female and male gene sets with the BUSCO tool.

**Supplementary Table S8**. The classification of gene origins within the X- and Y-SLR.

**Supplementary Table S9**. The collinearity relationship between the X-SLR of *H. salicifolia* and the homologous regions of closely related species.

**Supplementary Table S10**. The Ks values of homologous genes in different blocks within the SLRs of *H. salicifolia*.

**Supplementary Table S11**. Statistics of transcriptome sequencing of female and male mixed flower buds in *H. gyantsensis* and *H. salicifolia*.

**Supplementary Table S12**. TPM values of genes in the SLR of *H. salicifolia* across different tissues in males and females.

## Abbreviations

bp: base pairs; BUSCO: Benchmarking Universal Single-Copy Orthologs; CTAB: cetyl trimethyl ammonium bromide; Gb: gigabase pairs; HiC: high-throughput/resolution chromosome conformation capture; Ks: synonymous substitutions per synonymous site; Mb: megabase pairs; MSY: male-specific region; Mya: million years ago; NGS: next-generation sequencing; ONT: Oxford Nanopore Technologies; Pacbio: Pacific Biosciences; PAR: pseudoautosomal region; SDR: sex-determining region; SLR: sex-link region; TPM: transcripts per million.
