# Supplementary material for: The genome of Hippophae salicifolia provides new insights into the sexual differentiation of sea buckthorn
Source: Gigascience. 2025 Jul 2;14:giaf046. doi: 10.1093/gigascience/giaf046 (PMC12218201; doi:10.1093/gigascience/giaf046)
Supplement: giaf046_Supplemental_Files [file giaf046_supplemental_files.zip › Fig2.docx]

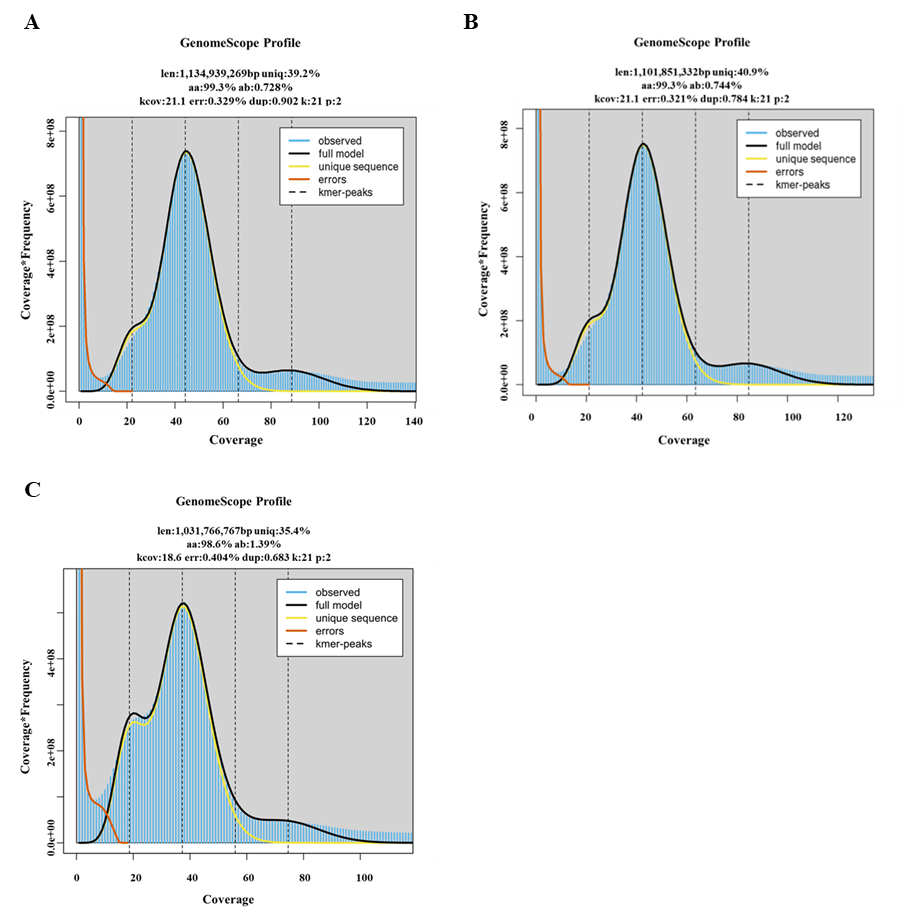


Fig. S1 Distribution of K-mer (K = 21) frequency in sequencing reads of the three seabuckthorn plants. (a) Distribution of K-mer frequency for female *H. salicifolia* from Illumina sequencing. (b) Distribution of K-mer frequency for male *H. salicifolia* from Illumina sequencing. (c) Distribution of K-mer frequency for male *H. gyantsensis* from Illumina sequencing.


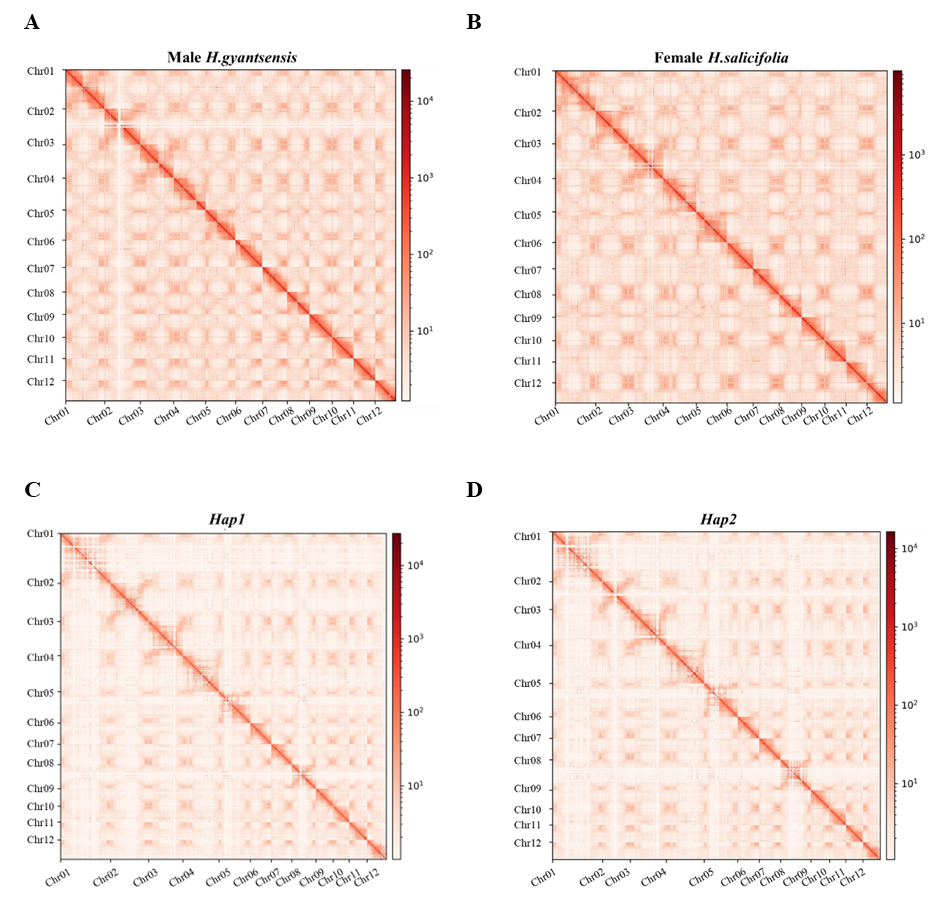


Fig. S2 Hi-C heatmap of chromosomes for male *H. gyantsensis* (a), female *H. salicifolia* (b), Hap1 (c), and Hap2 (d) of male *H. salicifolia*.


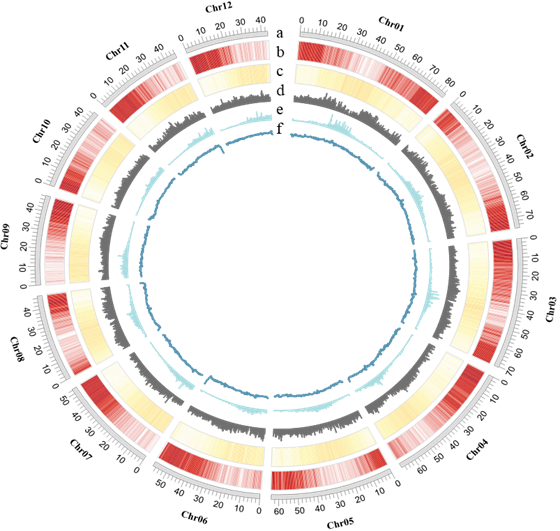


Fig. S3 Circos plot of the genomic landscape of male *H. gyantsensis*. (a) Pseudochromosome. (b) Gene density. (c) Repeat sequences density. (d) Gypsy density. (e) Copia density. (f) GC content.


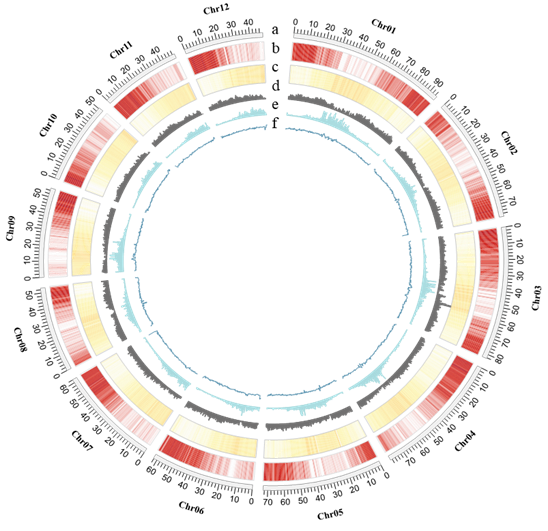


Fig. S4 Circos plot of the genomic landscape of female *H. salifolia*. (a) Pseudochromosome. (b) Gene density. (c) Repeat sequences density. (d) Gypsy density. (e) Copia density. (f) GC content.


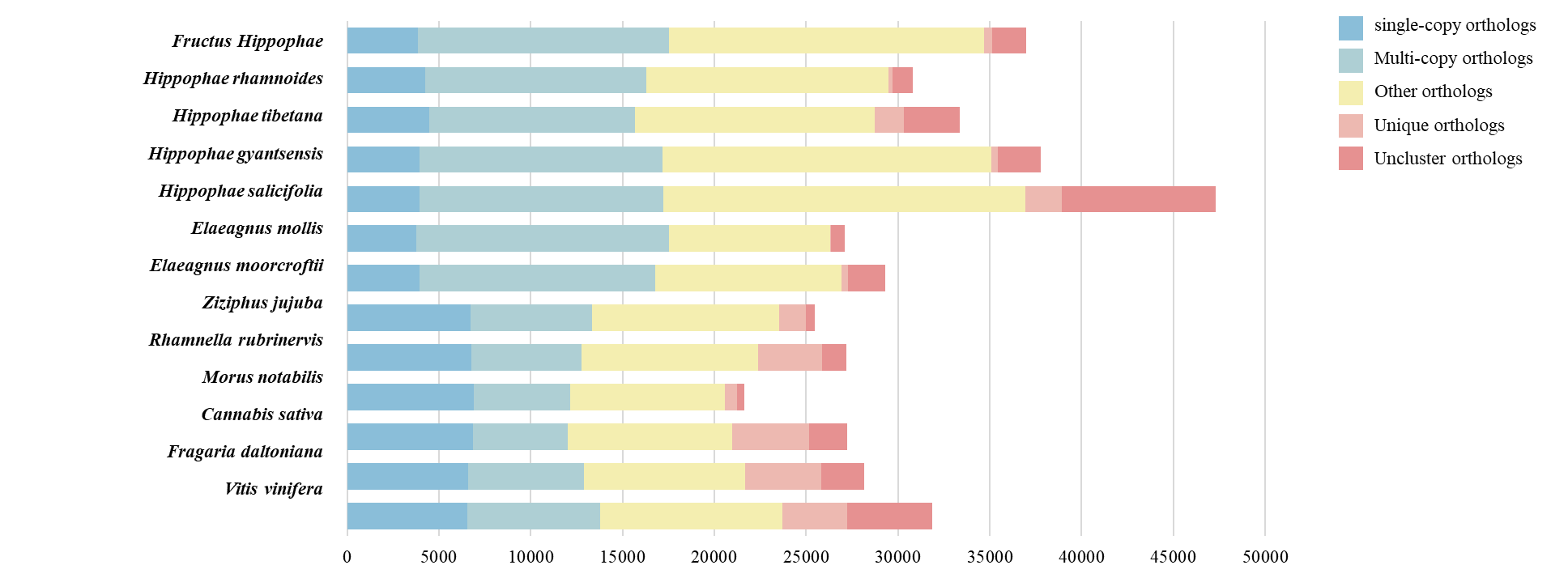


Fig. S5 Statistics of orthogroups in different plants defined by OrthoFinder.


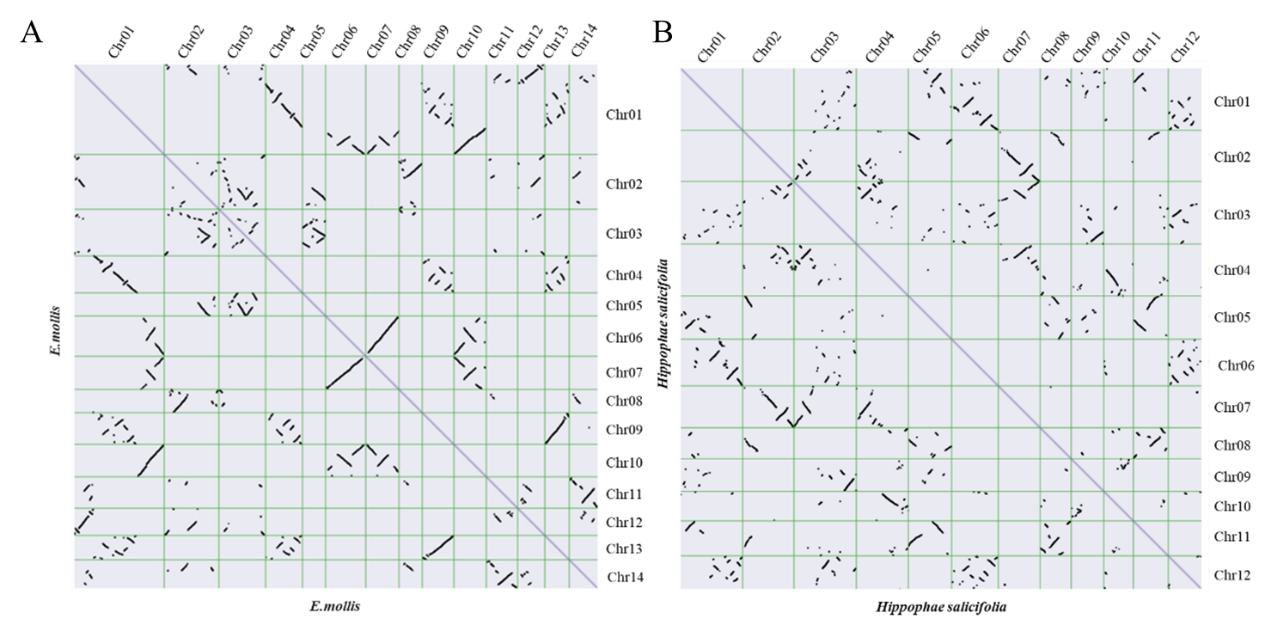


Fig. S6 The collinearity relationship dot plot within the *Elaeagnus mollis* genome and within the *H. salicifolia* genome. (a) *E. mollis* (b) *H. salicifolia*


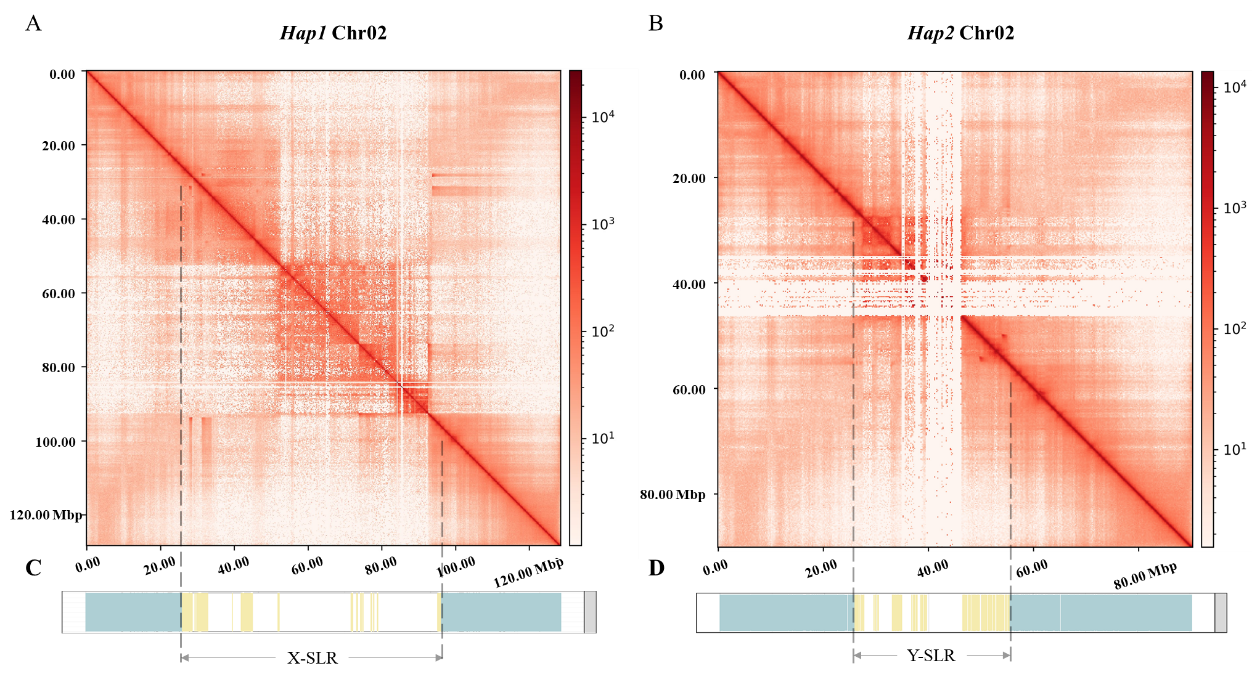


Fig. S7 Hi-C Schematic Diagram of the Sex Chromosomes in *H. salicifolia*. (a) Hap1 Chr02. (b) Hap2 Chr02. (c) the schematic of Chr02 in Hap1, with the yellow region highlighting the X-SLR. (d) the schematic of the sex chromosome in Hap2, where the yellow region denotes the Y-SLR.


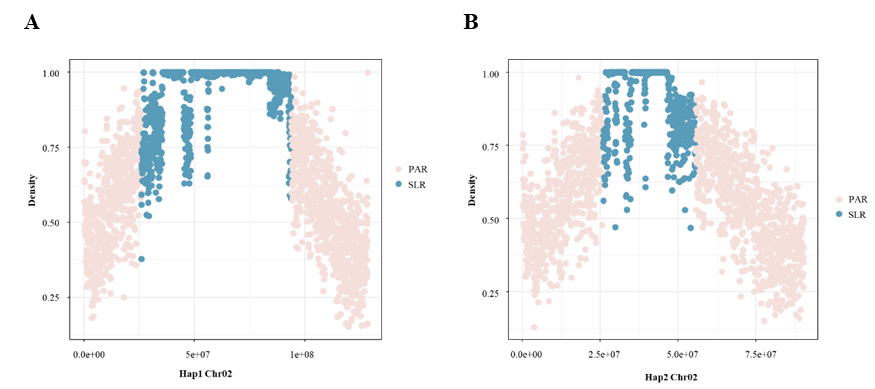


Fig. S8 Density plot of repetitive sequences on Chr02 for Hap1 (a) and Hap2(b). Pink dots represent the density of pseudoautosomal repeats, and blue dots represent the density of repeats in sex-linked regions.


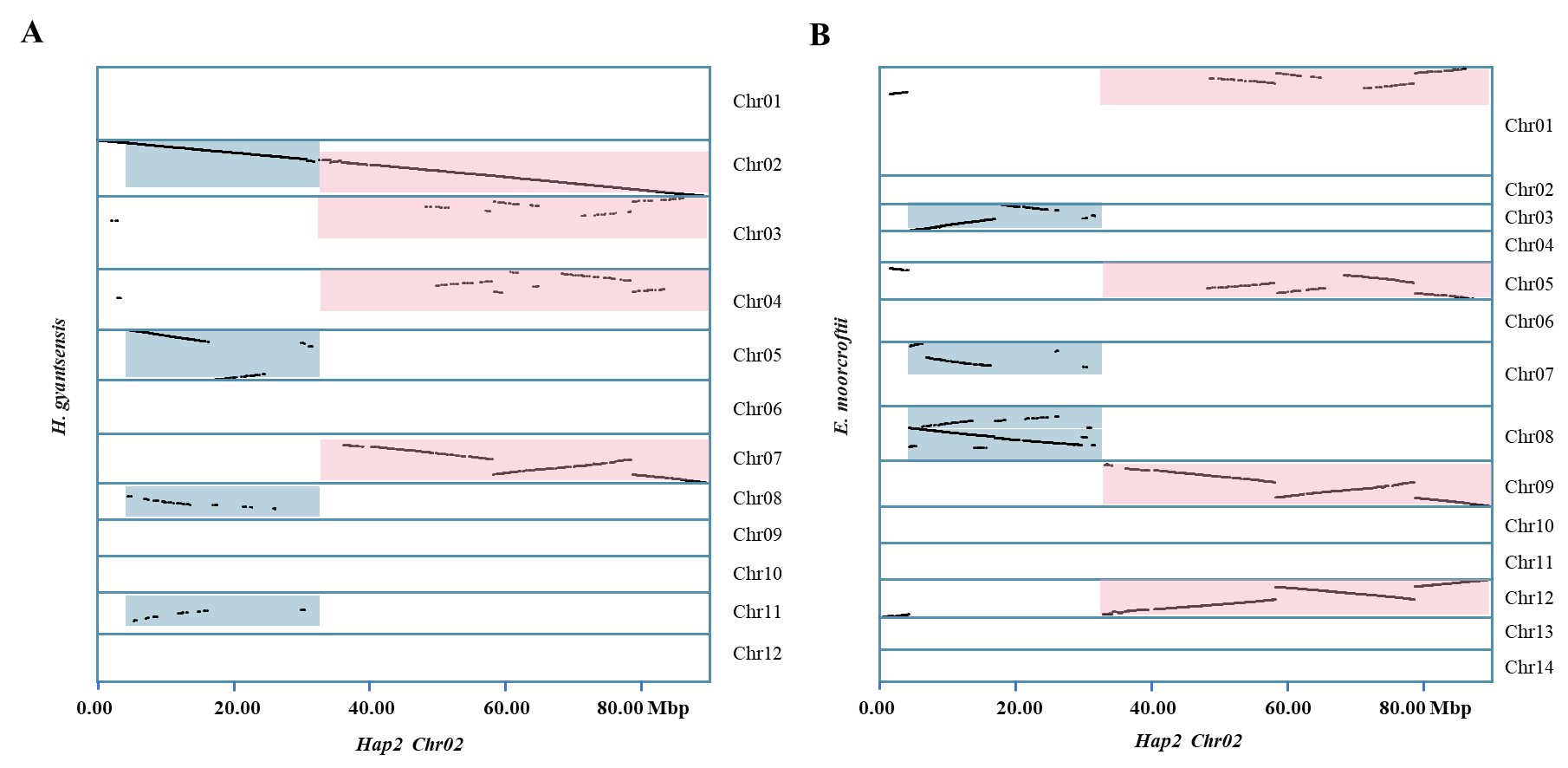


Fig. S9 Synteny relationship between Chr02 of Hap2 and its closely related species: (a) Syntenic point map of Hap2 Chr02 and whole genome of *H. gyantsensis* (b) Syntenic point map of Hap2 Chr02 and whole genome of *E. moorcroftii*.


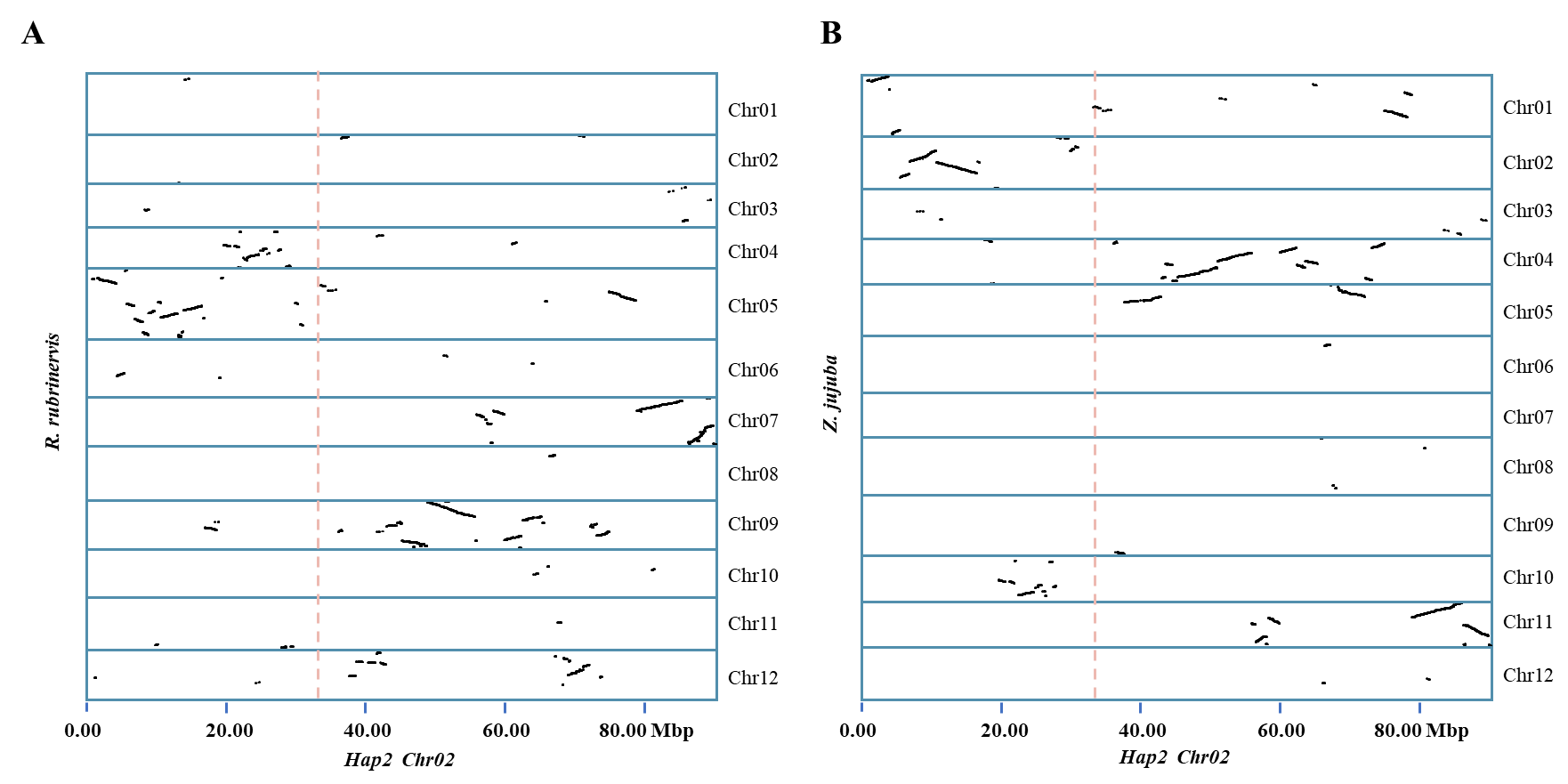


Fig. S10 Synteny relationship between chromosome 2 of Hap2 and its closely related species: (a) Synteny relationship between Chr02 of Hap2 and *Z. jujuba*. (b) Synteny relationship between Chr02 of Hap2 and *R. rubrinervis.*


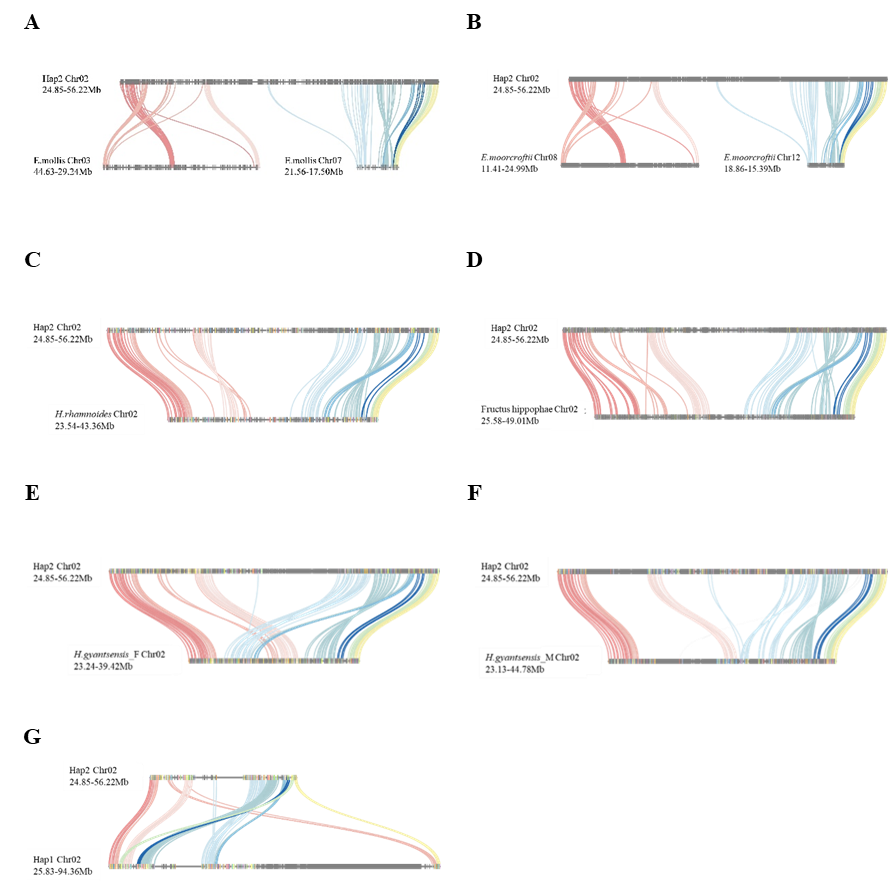


Fig. S11 M Microsynteny of homologous blocks between the Y-SLR of Hap2 and its closely related species. (a) *E. mollis* (b) *E. moorcroftii* (c) *H. rhamnoides* (d) Fructus Hippophae (e) female *H. gyantsensis* (f) male *H. gyantsensis* (g) Hap1.


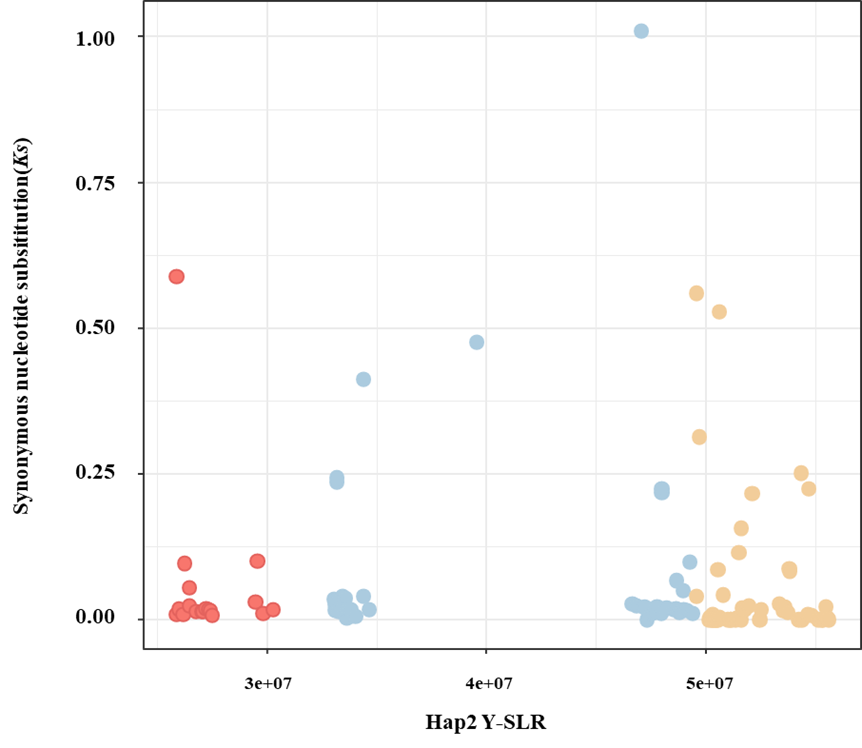


Fig. S12 The Ks values of XY homologous gene pairs within the Y-SLR are mapped to the positional coordinates of the Y chromosome. Blue represents gene pairs within stratum 1, red represents gene pairs within stratum 2-1, and yellow represents gene pairs within stratum 2-2.


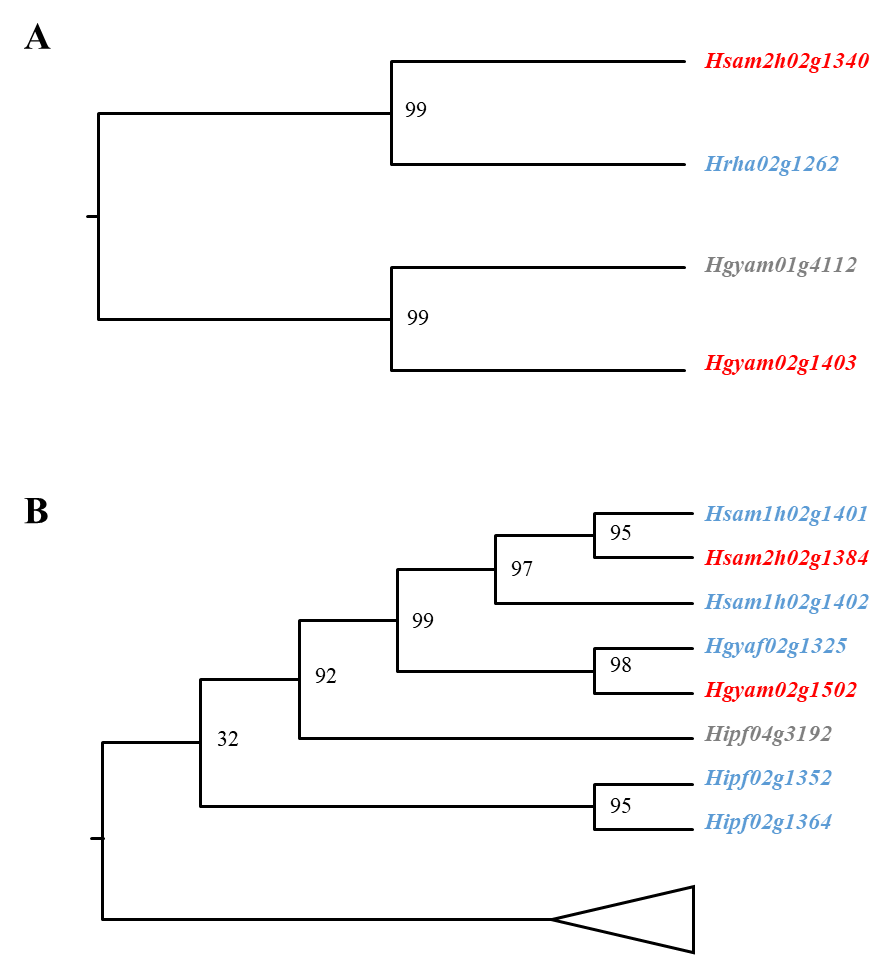


Fig. S13 Among the 11 male-specifically expressed genes, the phylogenetic trees of two genes are not consistent with the expected phylogenetic structure. The genes within the Y-SLR are marked in red, the genes within the X-SLR are marked in blue (a) *Hsam2h02g1340* (b) *Hsam2h02g1384*.


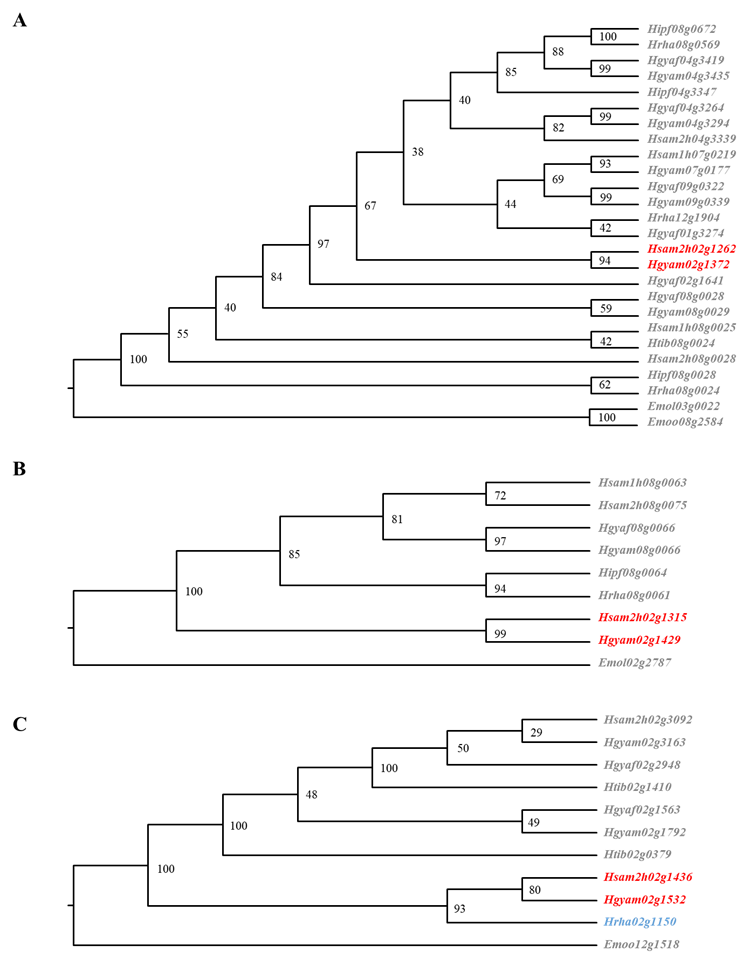


Fig. S14 Among the 11 male-specifically expressed genes, three are male-specific genes that lack corresponding homologous genes within the X-SLR. The genes within the Y-SLR are marked in red, the genes within the X-SLR are marked in blue. (a) *Hsam2h02g1262* (b) *Hsam2h02g1315* (c) *Hsam2h02g1436*.


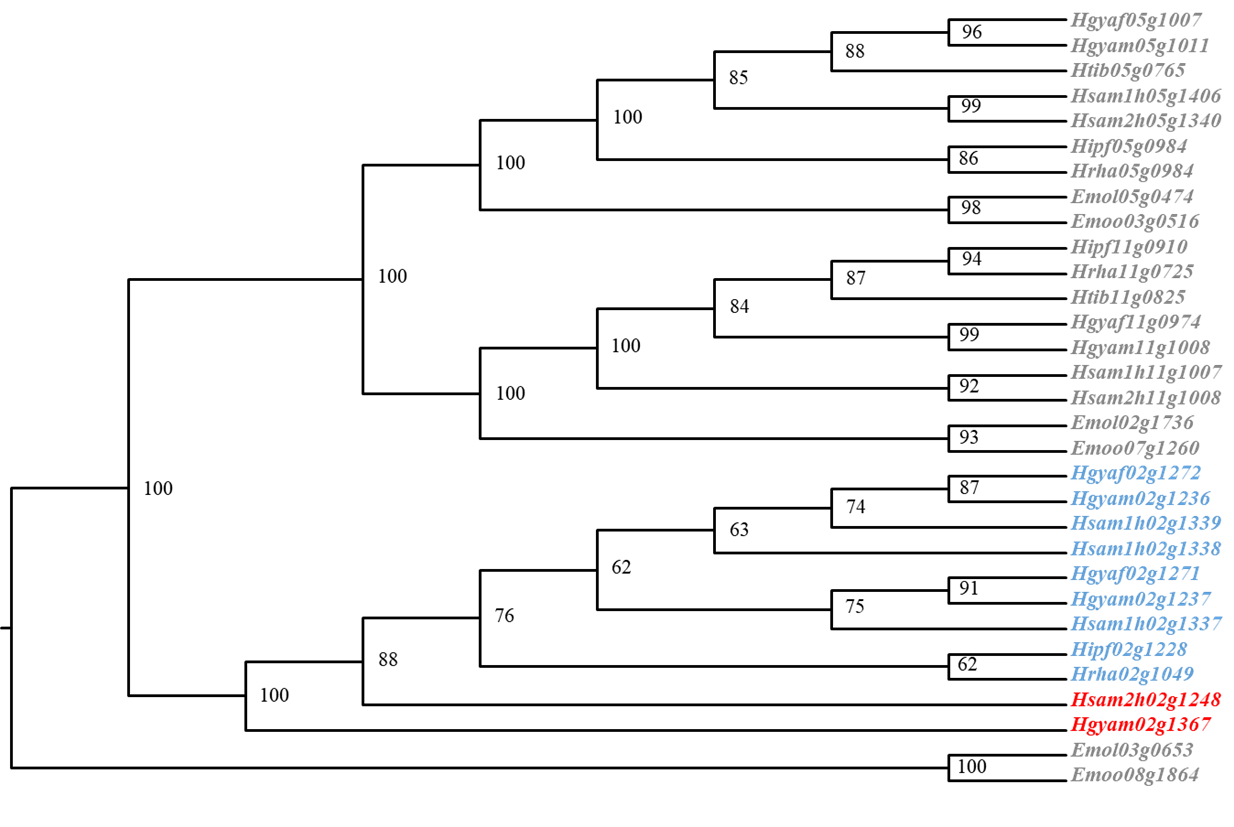


Fig. S15 Gene tree constructed by *Hsam2h02g1248* and its homologous genes. The genes within the Y-SLR are marked in red, the genes within the X-SLR are marked in blue.


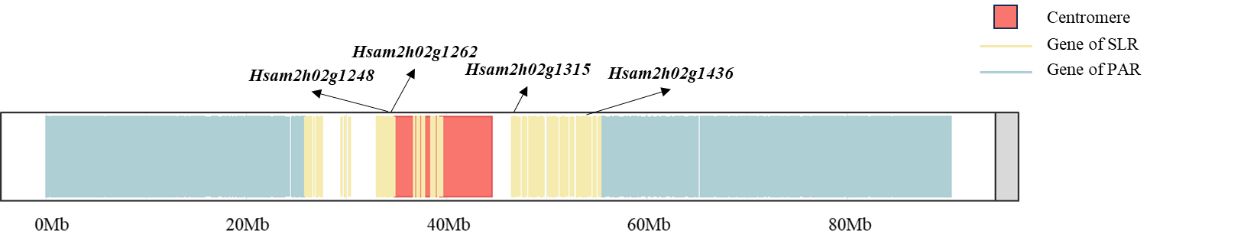


Fig. S16 The distribution of the four candidate genes within the SLR; three genes are located in the presumed ancient strata, one gene (*Hsam02g1436*) is close to the PAR region.


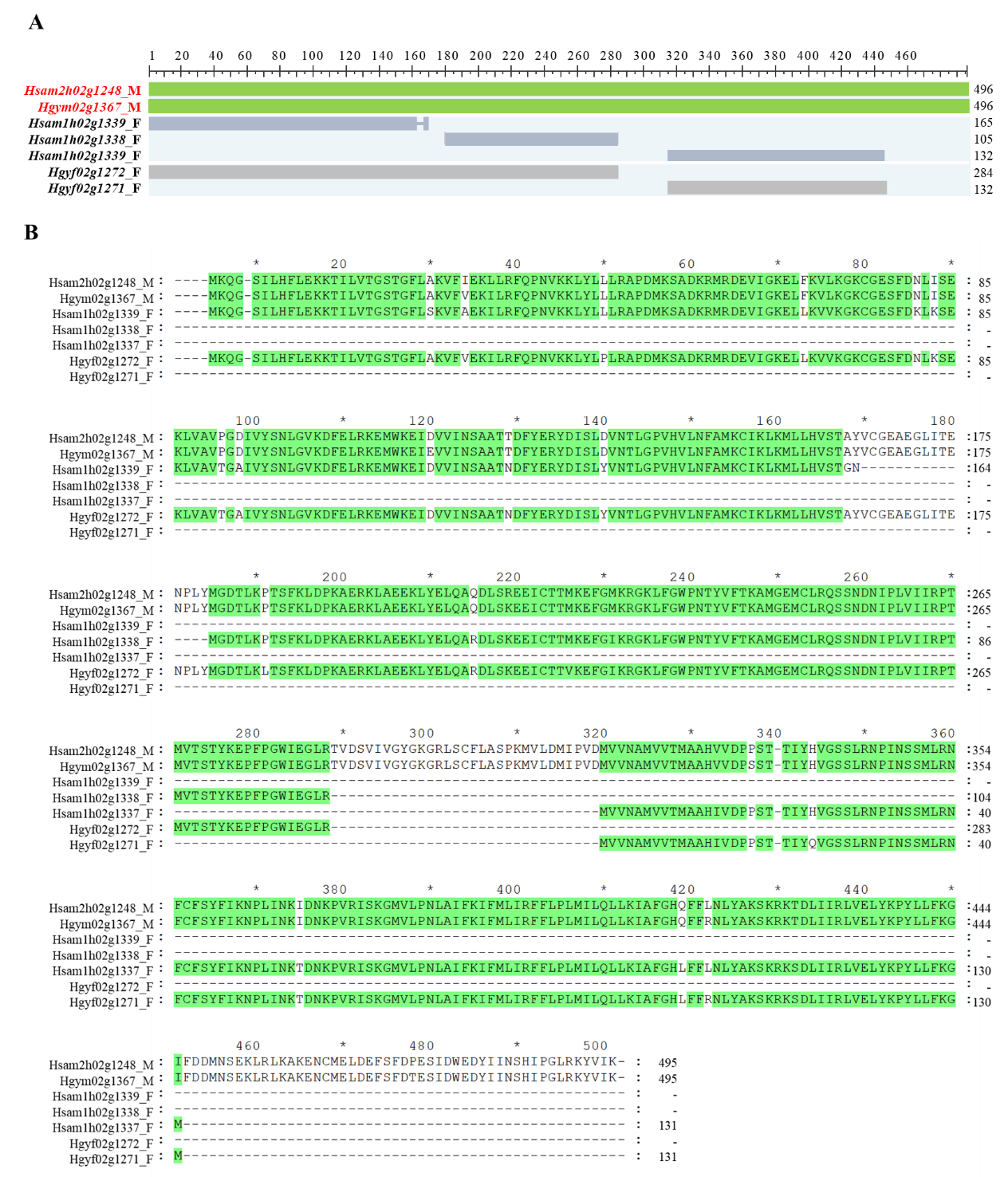


Fig. S17 The sequence alignment of *Hsam2h02g1248* with its homologous genes in the female- and male-specific linkage regions of *H. salicifolia* and *H. gyantsensis* reveals that gene fragmentation has occurred in the homologous genes of females.


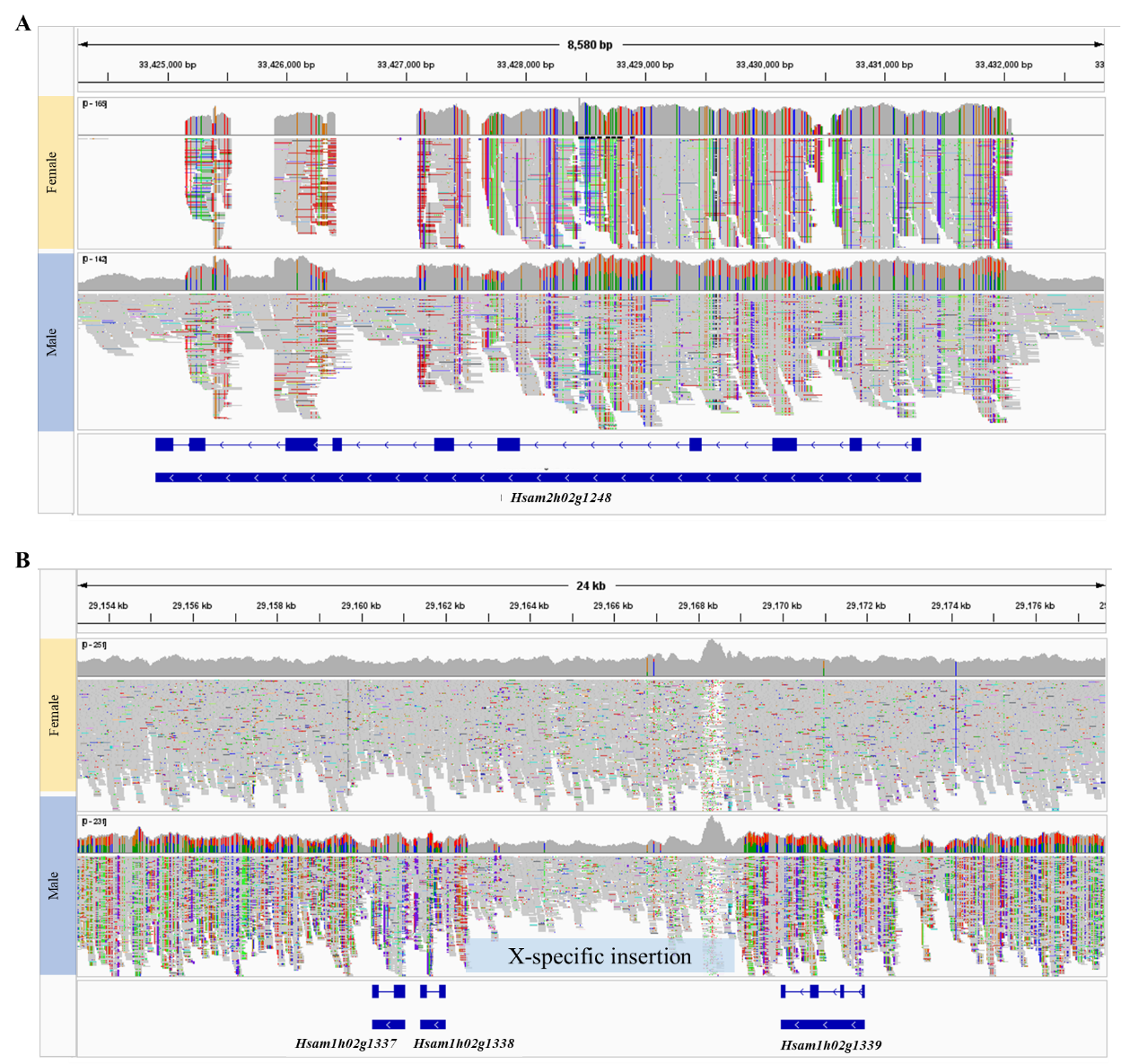


Fig. S18 Alignments of the resequencing reads from male and female individuals to the genes. (a) *Hsam2h02g1248*. (b) The homologous genes of *Hsam2h02g1248* in X-SLR were *Hsam1h02g1337*, *Hsam1h02g1338* and *Hsam1h02g1339*.


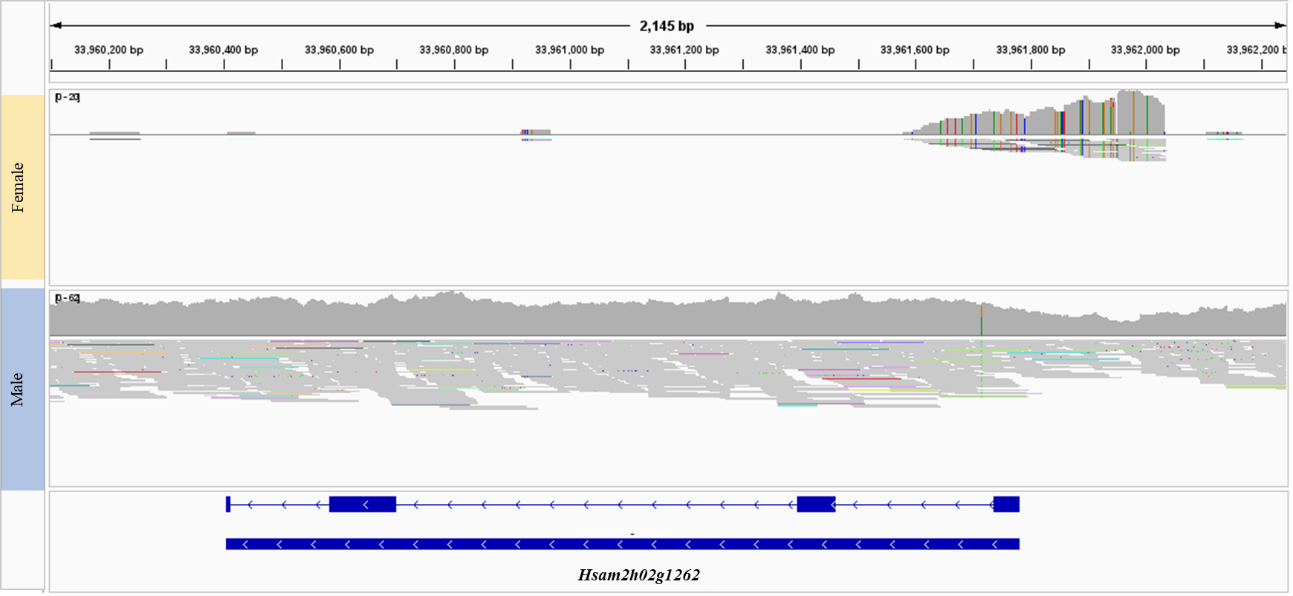


Fig. S19 Alignments of the resequencing reads from male and female individuals to the gene *Hsam2h02g1262*.


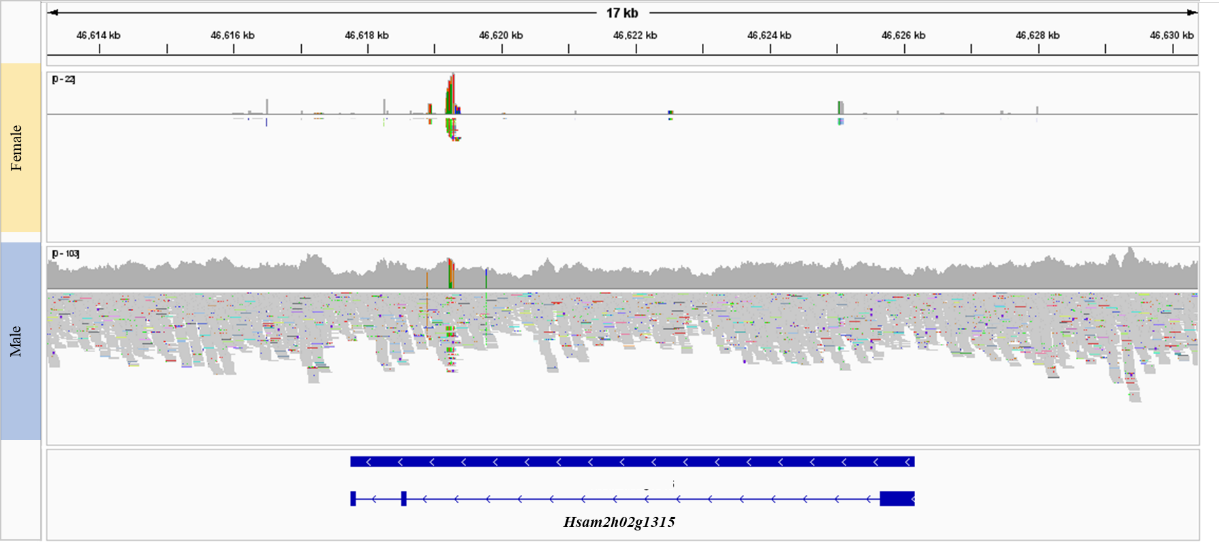


Fig. S20 Alignments of the resequencing reads from male and female individuals to the gene *Hsam2h02g1315.*
