# Supplementary material for: The genome of Hippophae salicifolia provides new insights into the sexual differentiation of sea buckthorn
Source: Gigascience. 2025 Jul 2;14:giaf046. doi: 10.1093/gigascience/giaf046 (PMC12218201; doi:10.1093/gigascience/giaf046)
Supplement: giaf046_GIGA-D-24-00421_original_submission [file giaf046_giga-d-24-00421_original_submission.pdf]

GigaScience

The genome of Hippophae salicifolia provides new insights into the sexual differentiation of seabuckthorn

--Manuscript Draft--

**Manuscript Number:**	GIGA-D-24-00421
**Full Title:**	The genome of Hippophae salicifolia provides new insights into the sexual differentiation of seabuckthorn
**Article Type:**	Research
**Funding Information:**	the Second Tibetan Plateau Scientific Expedition and Research (STEP) program (2019QZKK0502) — Dr Yongping Yang<br>Science and Technology Plan Projects of Tibet Autonomous Region (XZ202401ZY0006) — Dr Yongping Yang<br>Science and Technology Program Projects of Shigatse City (RKZ2023ZY-03) — Dr Shihai Yang<br>Yunling Scholar Project to Yang Yongping — Dr Yongping Yang
**Abstract:**	Background: Dioecy, a common reproductive strategy in angiosperms, has evolved independently in various plant lineages, and this has resulted in the evolution of diverse sex chromosome systems and sex determination mechanisms. Hippophae is a genus of dioecious plants with an XY sex determination system, but the molecular underpinnings of this process have not yet been clarified. Most previously published sea buckthorn genome data have been derived from females, yet genomic data on males are critically important for clarifying our understanding of sex determination in this genus. Comparative genomic analyses of male and female seabuckthorn plants can shed light on the origins and evolution of sex. These studies can also enhance our understanding of the molecular mechanisms underlying sexual differentiation and provide novel insights and data for future research on sexual reproduction in plants.<br>Results: We conducted an in-depth analysis of the genomes of two seabuckthorn species, including a male Hippophaegyantsensis, a female Hippophaesalicifolia, and two haplotypes of male H. salicifolia. The genome size of H. gyantsensis was 704.35 Mb, and that of the female H. salicifolia was 788.28 Mb. The sizes of the two haplotype genomes were 1,139.98 Mb and 1,097.34 Mb. The sex-determining regions (SDRs) of the two male sea buckthorn genomes were mapped. The SDR of H. salicifolia was 29.71 Mb and contained 249 genes. The SDR of H. gyantsensis was 12.31 Mb and contained 235 genes. A comparative analysis of the haplotypes of Chr02 of H. salicifolia revealed that the Y chromosome was shorter than the X chromosome. Chromosomal evolution analysis indicated that Hippophae has experienced significant chromosomal rearrangements following two whole-genome duplication events, and the fusion of two chromosomes has potentially led to the early formation of sex chromosomes in seabuckthorn. Multiple structural variations between Y and X sex-linked regions might have facilitated the rapid evolution of sex chromosomes in H. salicifolia. Comparison of the transcriptome data of male and female flower buds from H. gyantsensis and H. salicifolia revealed 11 genes specifically expressed in males. Three of these were identified as candidate genes involved in the sex determination of seabuckthorn. These findings will aid future studies of the sex determination mechanisms in seabuckthorn.<br>Conclusion: A comparative genomic analysis was performed to identify the sex-determination regions (SDRs) in H. gyantsensis and H. salicifolia. The origins and evolutionary trajectories of sex chromosomes within Hippophae were also determined. Three potential candidate genes associated with seabuckthorn sex determination were identified. Overall, our findings will aid future studies aimed at clarifying the mechanisms of sex determination.
**Corresponding Author:**	mingyue chen<br>XTBG: Xishuangbanna Tropical Botanical Garden<br>yunnan, 待投 CHINA
**Corresponding Author Secondary**	

Powered by Editorial Manager® and ProduXion Manager® from Aries Systems Corporation

**Information:**	
**Corresponding Author's Institution:**	XTBG: Xishuangbanna Tropical Botanical Garden
**Corresponding Author's Secondary Institution:**	
**First Author:**	Mingyue chen
**First Author Secondary Information:**	
**Order of Authors:**	Mingyue chen
	Xingyu Yang
	Lan Xun
	Zhenlin Qu
	Shihai Yang
	Yunqiang Yang
	Yongping Yang
**Order of Authors Secondary Information:**	
**Additional Information:**	
**Question**	**Response**
Are you submitting this manuscript to a special series or article collection?	No
**Experimental design and statistics**<br><br>Full details of the experimental design and statistical methods used should be given in the Methods section, as detailed in our Minimum Standards Reporting Checklist. Information essential to interpreting the data presented should be made available in the figure legends.<br><br>Have you included all the information requested in your manuscript?	Yes
**Resources**<br><br>A description of all resources used, including antibodies, cell lines, animals and software tools, with enough information to allow them to be uniquely identified, should be included in the Methods section. Authors are strongly encouraged to cite Research Resource Identifiers (RRIDs) for antibodies, model organisms and tools, where possible.	Yes

Powered by Editorial Manager® and ProduXion Manager® from Aries Systems Corporation

Have you included the information requested as detailed in our Minimum Standards Reporting Checklist?	
**Availability of data and materials**<br><br>All datasets and code on which the conclusions of the paper rely must be either included in your submission or deposited in publicly available repositories (where available and ethically appropriate), referencing such data using a unique identifier in the references and in the "Availability of Data and Materials" section of your manuscript.<br><br>Have you have met the above requirement as detailed in our Minimum Standards Reporting Checklist?	Yes

Powered by Editorial Manager® and ProduXion Manager® from Aries Systems Corporation

Manuscript Click here to access/download;Manuscript;salicifolia.docx

The genome of *Hippophae salicifolia* provides new insights into the sexual differentiation of seabuckthorn

Mingyue Chen [a,b,c], Xingyu Yang [a,b], Xun Lan [a,f], Zhenlin Qu [d], Shihai Yang[e,*], Yunqiang Yang[a,b*], Yongping Yang[a,b*]

[a] Xishuangbanna Tropical Botanical Garden, Chinese Academy of Sciences, Mengla, Yunnan 666303, China.

[b] Yunnan International Joint Laboratory for the Conservation and Utilization of Tropical Timber Tree Species, Xishuangbanna Tropical Botanical Garden, Chinese Academy of Sciences, Mengla, Yunnan 666303, China.

[c] School of Ecology and Environment, Yunnan University, Kunming 650091, China

[d] Beijing Jiu Tian International Education, Beijing 100080, China.

[e] Xizang Ecological Harmony Seed Industry Co, Ltd, Shigatse, Xizang 857000, China.

[f] College of Agriculture and Biotechnology, Yunnan agricultural university, Kunming 650500, China.

* corresponding author(s): Yunqiang Yang (yangyunqiang@xtbg.ac.cn) or Yongping Yang (yangyp@xtbg.ac.cn)

Abstract

Background: Dioecy, a common reproductive strategy in angiosperms, has evolved independently in various plant lineages, and this has resulted in the evolution of diverse sex chromosome systems and sex determination mechanisms. *Hippophae* is a genus of dioecious plants with an XY sex determination system, but the molecular underpinnings of this process have not yet been clarified. Most previously published sea buckthorn genome data have been derived from females, yet genomic data on males are critically important for clarifying our understanding of sex determination in this genus. Comparative genomic analyses of male and female seabuckthorn plants can shed light on the origins and evolution of sex. These studies can also enhance our understanding of the molecular mechanisms underlying sexual differentiation and provide novel insights and data for future research on sexual reproduction in plants.

Results: We conducted an in-depth analysis of the genomes of two seabuckthorn species, including a male *Hippophae gyantsensis*, a female *Hippophae salicifolia*, and two haplotypes of male *H. salicifolia*. The genome size of *H. gyantsensis* was 704.35 Mb, and that of the female *H. salicifolia* was 788.28 Mb. The sizes of the two haplotype genomes were 1,139.98 Mb and 1,097.34 Mb. The sex-determining regions (SDRs) of the two male sea buckthorn genomes were mapped. The SDR of *H. salicifolia* was 29.71 Mb and contained 249 genes. The SDR of *H. gyantsensis* was 12.31 Mb and contained 235 genes. A comparative analysis of the haplotypes of Chr02 of *H. salicifolia* revealed that the Y chromosome was shorter than the X chromosome. Chromosomal evolution analysis indicated that *Hippophae* has experienced significant chromosomal rearrangements following two whole-genome duplication events, and

the fusion of two chromosomes has potentially led to the early formation of sex chromosomes in seabuckthorn. Multiple structural variations between Y and X sex-linked regions might have facilitated the rapid evolution of sex chromosomes in *H. salicifolia*. Comparison of the transcriptome data of male and female flower buds from *H. gyantsensis* and *H. salicifolia* revealed 11 genes specifically expressed in males. Three of these were identified as candidate genes involved in the sex determination of seabuckthorn. These findings will aid future studies of the sex determination mechanisms in seabuckthorn.

Conclusion: A comparative genomic analysis was performed to identify the sex-determination regions (SDRs) in *H. gyantsensis* and *H. salicifolia*. The origins and evolutionary trajectories of sex chromosomes within *Hippophae* were also determined. Three potential candidate genes associated with seabuckthorn sex determination were identified. Overall, our findings will aid future studies aimed at clarifying the mechanisms of sex determination.

Keywords: *Hippophae*, chromosomal assembly, sex chromosomes, sex determination

Introduction

Clarifying the genetic mechanisms underlying sexual differentiation, as well as the origins and evolution of sex chromosomes, is a major goal of evolutionary biology. Conventional theories suggest that sex chromosomes evolve from autosomes through a series of genetic and evolutionary changes, which ultimately become determinants of sex. This evolutionary trajectory is often initiated with two closely linked mutations on an autosome: a dominant mutation that inhibits female development and a recessive mutation inducing male sterility [1,2]. Due to the genetic conflict associated with sex differences in reproductive strategies, sexually

antagonistic genes accumulate around these mutant genes, which suppresses recombination [3]. As time progresses, the inhibition of recombination can extend along the chromosome, leading to the establishment of sex-linked regions, and eventually may lead to the emergence of heteromorphic sex chromosomes [4,5]. However, previous studies have shown that the evolution of sex chromosomes does not always result in heteromorphic pairs. For example, the sex chromosomes in scallops have remained highly undifferentiated for over 350 million years. In these species, homomorphic sex chromosomes are notably enriched with numerous bidirectionally reversible sex-biased genes, which might be critically important for maintaining their undifferentiated state [6]. These findings indicate that sex chromosome evolution can follow various patterns. Many aspects of sex chromosomes have not yet been clarified, including the origin of sex and recombination suppression [7]. In contrast to the pronounced differentiation in human and animal sex chromosomes, plant sex chromosomes have arisen and evolved independently across diverse dioecious plant lineages, and they exhibit high diversity and complexity in their evolutionary pathways [8]. Investigation of the sex determination mechanisms in these plants can provide novel insights and data crucial for elucidating the genetic basis of sexual differentiation.

Dioecious plants account for 5% to 6% of angiosperms; sexual differentiation in dioecious plants provides them with unique reproductive advantages [9]. Dioecious plants can optimize the allocation of reproductive resources and enhance breeding efficiency through sexual differentiation [10]. Furthermore, sexual differentiation can affect their ecological niche [11,12]. Some dioecious plants may reduce self-fertilization and increase gene flow through sexual differentiation, which enhances the genetic diversity of the population. The differentiation of

sex chromosomes plays a key role in the molecular mechanism underlying sexual differentiation in dioecious plants. Sex chromosomes, such as the XY and ZW systems, are typically derived from autosomes and evolve into sex-determining chromosomes via a series of genetic and evolutionary processes [2]. The expression patterns of genes on the sex chromosomes, the formation of recombination suppression regions, and the morphological and functional differentiation of sex chromosomes are all key factors affecting these processes [13,14]. Sex-specific gene expression patterns play a key role in sex determination. In dioecious plants, certain genes may be expressed in only one sex, and these differences in gene expression directly affect sex-specific traits [15]. The formation of regions with suppressed recombination is another critical factor affecting sex chromosome differentiation. The recombination frequency in certain regions significantly decreases during sex chromosome evolution, and this phenomenon is known as recombination suppression [14]. Recombination suppression aids the accumulation of sex-specific genes and the stabilization of sex-determining mechanisms. In these regions, gene flow is restricted, which promotes the differentiation of sex-specific genes and the morphological differentiation of sex chromosomes [16].

The identification and functional analysis of sex-determining genes are critically important in studies of sexual differentiation in dioecious plants [8]. Dioecious plants have evolved independently in different lineages and possess distinct sex-determining genes. However, these genes are products of convergent evolution and have similar characteristics that can facilitate their identification. However, the identification of sex-determining genes is extremely challenging because of various factors, such as heterochromatinization and structural variation; the sex-determining genes of only a few species have been successfully characterized to date.

Diospyros lotus is the first plant from which sex-determining genes were successfully isolated and identified [17]. Illumina sequencing data from the F1 generation of both female and male plants have been used for the assembly of the male-specific region (MSY) of the Y chromosome. They were then integrated into bud transcriptome data, and 22 differentially expressed genes were identified within the MSY. Phylogenetic analysis revealed that *OGI* is the only gene that predates the speciation event within Diospyros. Evolutionary analyses indicated that *OGI* is a best candidate for a sex-determining gene, and this hypothesis was confirmed in subsequent experiments. Similarly, the MSY region was assembled in kiwifruit using Illumina sequencing data from the F1 generation, and differential expression analysis was performed using transcriptome data from flower buds at various developmental stages. This approach led to the identification of two male-specific genes, *SyGI* and *FrBy*, which are conserved across all kiwifruit and lack homologous counterparts in females. Transgenic studies have shown that *SyGI* is a dominant inhibitor of carpel development, and *FrBy* knockout results in pollen inactivation and self-incompatibility [18,19]. Despite advances in sequencing technology, which has enabled the identification of some plant sex-determining genes, only a few such genes have been isolated and characterized.

Seabuckthorn (*Hippophae*), which belongs to the family Elaeagnaceae, is a shrub or small tree with distinct dioecious characteristics and simple and delicate flowers. The berries of seabuckthorn are renowned for their medicinal properties and nutritional richness; they thus play a key role in the pharmaceutical, cosmetic, and food industries [20,21]. This species has been used for ecological restoration because of its high stress tolerance and vigorous growth; it also can reproduce prolifically and shows significant nitrogen-fixation activity, which makes it

ideal for wind protection, sand stabilization, and reforestation in arid regions [22]. Seabuckthorn has an XY sex chromosome system and shows clear sexual dimorphism; males typically bear cone-like inflorescences that bloom before the females, whereas females have raceme inflorescences that are comparatively smaller [23]. The genomic sequences of *Hippophae tibetana* [24], *Hippophae rhamnoides* subsp. sinensis [25], *Hippophae rhamnoides* subsp. mongolica [26], and *H. gyantsensis* [27] have all been published. The sex chromosome of *H. tibetana* was identified as the second chromosome through simplified genome-wide association studies [24]; however, detailed research on its sex chromosomes has not yet been conducted. Therefore, studies of the sex chromosomes of male seabuckthorn at the genomic level can provide important insights into sexual differentiation in dioecious plants and genetic resources that could aid the breeding of seabuckthorn. These findings will aid future molecular studies of the sex determination mechanisms of seabuckthorn, as well as ongoing efforts to improve varieties and their applications in medicine, ecology, and other fields.

The genetic basis of sexual differentiation in seabuckthorn has been a major focus of studies of plant reproductive biology and sexual evolution. Here, we assembled the genomes of a male *H. gyantsensis*, a female *H. salicifolia*, and two male *H. salicifolia* with haplotype resolution. Specifically, we conducted a comparative genomic analysis to identify the SDRs in both species and examined their genomic distribution and characteristics, along with the origin and evolution of the sex chromosomes of seabuckthorn. Analysis of transcriptomic data from male and female flowers revealed three candidate genes. Our findings provide new insights into sexual differentiation in seabuckthorn and will aid ongoing efforts to enhance sex-related traits and molecular breeding in the future.

Results

Genome estimation, sequencing, and assembly

To evaluate the genome size and heterozygosity of the three seabuckthorn species, we conducted genomic studies using clean reads obtained from the Illumina next-generation sequencing (NGS) platform: male *H. salicifolia* (57.60Gb), male *H. gyantsensis* (51.95Gb), and female *H. salicifolia* (62.31Gb) (Supplementary Table S1). K-mer (k=21) analysis (Supplementary Fig. S1) indicated that the genome size of male *H. gyantsensis* was approximately 1,031.76 Mb, with a heterozygosity rate of approximately 1.39%; the genome size of male *H. salicifolia* was approximately 1,018.51 Mb, with a heterozygosity rate of approximately 0.744%; and the genome size of female *H. salicifolia* was approximately 1,134.93 Mb, with a heterozygosity rate of approximately 0.728%.

We obtained clean reads from Oxford Nanopore (ONT) sequencing for female *H. salicifolia* (112.31 Gb) and male *H. gyantsensis* (99.03 Gb) to assemble the genomes of the three seabuckthorn species; HiFi reads were obtained for male *H. salicifolia* (142.55 Gb) (Supplementary Table S1). Hi-C sequencing generated 79.47 Gb of clean reads for male *H. salicifolia*, 82.52 Gb for female *H. salicifolia*, and 136.13 Gb for male *H. gyantsensis*, which were used for chromosome anchoring (Supplementary Table S2). The contigs of the three genomes were anchored to 12 pseudochromosomes (Supplementary Fig. S2). The genome size of male *H. gyantsensis* was 704.35 Mb; the contig N50 was 18.11 Mb(Supplementary Fig. S3); the genome size of female *H. salicifolia* was 788.28 Mb, with a contig N50 of 30.83 Mb (Supplementary Fig. S4). Male *H. salicifolia* was assembled into two haplotypes, Hap1 and

Hap2. The genome size of Hap1 was 1,139.98 Mb, with a contig N50 of 53.61 Mb; the genome size of Hap2 was 1,097.34 Mb, with a contig N50 of 64.70 Mb (Supplementary Table S3) (Fig. 1). The genomic BUSCO completeness rates scores were 97.4% for male *H. gyantsensis*, 97.6% for female *H. salicifolia*, 97.6% for male *H. salicifolia* Hap1, and 97.7% for male *H. salicifolia* Hap2 (Supplementary Table S4).

Genome annotation

The repetitive sequence content of the male *H. gyantsensis* genome was 56.42%, and long terminal repeat sequences (LTRs) accounted for 35.06% of the repetitive sequences. The repetitive sequence content of the female *H. salicifolia* genome was 60.41%, and LTRs comprised 37.68% of the repetitive sequences. For Hap1, the repetitive sequence content was 70.88%, of which LTRs comprised 43.41% of the repetitive sequences; for Hap2, the repetitive sequence content was 70.90%, and LTRs comprised 36.14% of the repetitive sequences (Supplementary Table S5).

In the male *H. gyantsensis* genome, 36,482 genes were annotated, and 33,238 were present in at least one database: Swissprot, KEGG, TrEMBL, nr, InterPro, and GO. In the female *H. salicifolia* genome, 39,501 genes were annotated, and 34,811 were present in at least one of the aforementioned databases. In the Hap1 genome, 45,937 genes were annotated, including 38,287 in at least one database. In the Hap2 genome, 39,854 genes were annotated, including 34,460 in at least one database (Supplementary Table S6). The BUSCO completeness scores for gene sets in the male *H. gyantsensis*, female *H. salicifolia*, male *H. salicifolia* Hap1, and male *H. salicifolia* Hap2 genomes were 98.9%, 98.8%, 98.9%, and 98.7%, respectively (Supplementary

Table S7). These findings confirm the high quality of the four seabuckthorn genomes sequenced.

Comparative genomics analysis

To investigate the phylogenetic relationships of seabuckthorn, we conducted a gene family analysis of 13 species, including seabuckthorn and other plants in the family Elaeagnaceae (Supplementary Fig. S5). A phylogenetic tree was constructed using 1,272 single-copy genes from these genomes. Our results indicate that the genus *Hippophae* and *Elaeagnus* diverged approximately 19.89 million years ago (mya), and there was a notable expansion of gene families preceding their divergence. Within *Hippopha*e, *H. tibetana*, *H. rhamnoides*, and Fructus hippophae were grouped in Clade 1, whereas *H. salicifolia* and *H. gyantsensis* comprised Clade 2. *H. salicifolia* has recently experienced two whole-genome duplication (WGD) events, which is consistent with the results of previous studies of seabuckthorn [25,27]. Synteny analysis revealed one-to-one homology relationships between chromosomal segments of *Hippophae* and *Elaeagnus* species, which indicates that they have a shared history of two WGD events. However, the synteny blocks within *Hippophae* were shorter than those in *Elaeagnus*, indicating that the seabuckthorn genome has undergone a higher frequency of chromosomal rearrangements.

SDR characteristics of *H. gyantsensis* and *H. salicifolia*

To identify the sex-linked regions (SLRs) in *H. salicifolia*, we compared the next-generation sequencing (NGS) coverage of male and female genomes. We identified a distinct SLR on Chr02 in Hap2 (Fig. 3a&3b). Males exhibited a coverage depth of approximately 50%

of the genome-wide average, whereas females had negligible coverage in this region; this region was designated as Y-SLR. Using a similar approach for Hap1, we identified an X-SLR (Figure 3c&3d), wherein females displayed average coverage depths, and males showed reduced coverage depths. The Y-SLR in *H. salicifolia* spanned 29.71 Mb and was positioned between 25.85 Mb and 55.55 Mb on Chr02, which comprised 32.88% of the chromosome's length and encompassed 249 genes. The chromosome was bookended by undifferentiated pseudoautosomal regions (PARs), totaling 60.64 Mb. Conversely, the X-SLR extended 68.02 Mb from 25.83 Mb to 93.86 Mb on Chr02, comprising 52.96% of the chromosome's length; this region included 334 genes and contained a PAR measuring 60.41 Mb, which was similar to the length of the Y-PAR. Applying the same methodology to *H. gyantsensis*, we identified a preliminary Y-SLR1, 5.6 Mb in length, that was located between 33.86 Mb and 39.47 Mb on Chr02(Supplementary Fig. S6). Given that the *H. gyantsensis* genome data were unphased, we used the phasing information from *H. salicifolia* for synteny analysis to refine the SLR regions. The refined Y-SLR in *H. gyantsensis* was 12.31 Mb, which spanned two segments from 14.59 Mb to 15.97 Mb and 33.86 Mb to 44.78 Mb and contained 235 genes.

During sex chromosome evolution, genes within SLRs often become degraded and show reduced recombination, which affects the expression and functions of genes [30]. The results of this study provide an in-depth classification and analysis of the genes within the X- and Y-SLRs in the genus *Hippophae* to clarify the evolutionary mechanisms of the sex chromosomes. Genes in the SLRs were classified into four categories based on homology and origin: "ancestral" genes, "acquired" genes, duplicated genes, and unique genes. The Y-SLR contained 249 genes, which comprised 99 ancestral, 73 duplicated, 53 acquired, and 24 unique genes; the

X-SLR contained 100 ancestral, 82 duplicated, 120 acquired, and 31 unique genes (Supplementary Table S8). The similarity in ancestral gene numbers between the X- and Y-SLRs indicates that there was no significant difference in the degree of degradation of these regions. However, the greater number of genes in X-SLR was attributed to a greater number of insertions of acquired genes. Further analysis indicated that the SLRs of *H. salicifolia* were close to the centromere, which is an area enriched with repetitive sequences. In the Y-SLR, these repetitive sequences comprised 90.58% of the 26.91 Mb region; in the PAR, they comprised 55.32% of this region. The content of repetitive sequences was higher in the X-SLR (94.74%) than in the Y-SLR within the 64.44 Mb region, including 54.86% in the PAR (Supplementary Fig. S7). Although the repetitive sequence content was similar in the PAR regions of both sex chromosomes, the content of repetitive sequences was significantly higher in the X-SLR than in the Y-SLR. These findings indicate that the higher number of acquired genes and repetitive sequences in the X-SLR is likely the main cause of the pronounced morphological divergence between the X and Y chromosomes.

Evolution of the Sex Determination Region in *H. salicifolia*

To elucidate the origin of the sex chromosomes in the genus *Hippophae*, we compared Chr02 in *H. salicifolia* with the genomes of *H. gyantsensis*, *H. rhamnoides*, *Elaeagnus moorcroftii*, and *Elaeagnus mollis* (Fig. 4a) (Supplementary Fig. S8). The synteny analysis identified two major collinear blocks adjacent to the SLRs on Chr02 of *H. salicifolia*, which were designated as L and R. Four copies of each of these two blocks were present within *Hippophae* and *Elaeagnus*, indicating that they might have been present prior to their

divergence. On Chr02 of seabuckthorn, the L and R blocks were merged (L-R), a configuration that was not observed on other chromosomes or within the closely related *Elaeagnus*. The L and R blocks were also separated in outgroup taxa such as *Ziziphus jujuba* and *Rhamnella rubrinervis* (Supplementary Fig. S9). These observations indicate a lineage-specific chromosomal fusion event in seabuckthorn, and Chr02 was possibly derived from the merging of two ancestral chromosomes. This fusion might have had a profound effect on the sex determination and reproductive strategies of *Hippophae*.

We performed a microsynteny analysis in which the Y-SLR of *H. salicifolia* was compared with homologous regions in the Y-SLR of other *Hippophae* species and *Elaeagnus* to clarify the evolution of these regions following the formation of sex chromosomes. The collinear regions were categorized into nine distinct blocks through comparison of chromosomal segment synteny (Supplementary Fig. S10 and Table S9). Discrepancies in the positions of blocks A and B between *Elaeagnus* and *Hippophae* were noted (Fig. 4b). However, the ancestral configuration of these blocks remains uncertain because of a lack of systenic information from outgroup comparisons; we hypothesize that *Hippophae* and *Elaeagnus* have structurally diverged. A comparative analysis of the block arrangement and orientation across closely related species has revealed that the Y-SLR in *H. salicifolia* is more structurally conserved than the X-SLR. Specifically, in the X-SLR, blocks A2, G, F, and E1 have been translocated, and blocks A1 have been inverted. In contrast, only a single translocation involving block E2 was identified in the Y-SLR, block D is reversed in X-SLR or Y-SLR, or both. We computed the Ks values for homologous gene pairs within these blocks to infer the sequences for the different structural variations (Supplementary Table S10). Block D had the highest Ks value (Ks=0.1266),

followed by block F (Ks=0.082), block E1 (Ks=0.0442), block E2 (Ks=0.0409), block A2 (Ks=0.03854), block A1 (Ks=0.01429), and block G (Ks=0.0037). The elevated Ks value of block D indicates that its structural changes occurred earlier than those of other blocks, and the lower Ks values for A1 and G suggest that their structural changes occurred recently.

Chromosomal rearrangement events are the main drivers of recombination suppression and sex chromosome differentiation [14]. Recombination suppression can promote increased sequence divergence in different regions of the sex chromosomes, forming so-called "evolutionary strata", which reflect the history and chronological order of structural variations in the evolution of sex chromosomes [31]. We analyzed the evolutionary rate of genes within the Y-SLR of *H. salicifolia* by calculating the Ks values of XY homologous gene pairs and mapping these Ks values onto the corresponding evolutionary strata (Supplementary Fig. S11). Based on the distribution of Ks values, we divided the Y-SLR into two presumed evolutionary strata. Stratum 1 (including blocks C and D) was located from 33.13 Mb to 49.72 Mb, and the average Ks value was 0.1041 for the 43 homologous gene pairs in this region. Stratum 2-1 (including blocks A and B) was located from 25.88 Mb to 33.08 Mb and contained 19 homologous gene pairs with an average Ks value of 0.0574. Stratum 2-2 (including blocks E, F, and G) was located from 50.19 Mb to 55.61 Mb and contained 38 homologous gene pairs with an average Ks value of 0.0418 (Fig 4c). Distinct phylogenetic patterns were observed for genes from different evolutionary strata. In the ancient strata, genes from the same gametophyte tended to cluster together, whereas in the newer strata, homologous genes from the same species tended to cluster together. We constructed a phylogenetic tree for the single-copy genes within the SLRs and analyzed the topology of the tree. In Stratum 1, an ancient origin was observed

for 16 out of 34 single-copy genes; in presumed Strata 2-1 and 2-2, the numbers of genes with an ancient origin were 1 and 3, respectively (Fig 4d). These results indicate that Stratum 1 represents a more ancient part of the sex chromosomes in *H. salicifolia*, and Strata 2-1 and 2-2 are newer. This, combined with the results of the Ks analysis of structural variations, indicated that the inversion of block D preceded the formation of Stratum 1, and the inversions of blocks F, E, and A2 were closely related to the formation of Stratum 2. These findings suggest that chromosomal structural variations between X- and Y-SLRs might have played a key role in promoting the spread of recombination suppression and the evolution of sex chromosomes.

Seabuckthorn sex determination candidate genes

To identify candidate genes involved in sex determination in seabuckthorn, we analyzed the transcriptional profiles of male and female mixed flower buds at various developmental stages of *H. gyantsensis* and *H. salicifolia* (Supplementary Table S11). We utilized transcriptome data, compared these data with the Y-SLR of *H. salicifolia*, and calculated the transcripts per million (TPM) values of the transcripts (Supplementary Table S15). This analysis identified 11 genes specifically expressed in males (Figure 5). Sex-determining genes often share phylogenetic similarities with ancient strata genes, and homologous genes from the same gametophyte tended to cluster together. We characterized the topology of the phylogenetic tree of the candidate genes. Our findings indicated that five of the 11 candidate genes lacked detectable homologs in male *H. gyantsensis*, and the topological structure of the phylogenetic tree of two genes did not match expectations (Supplementary Fig. S12), while the topological structures of the remaining four genes were consistent with our hypothesis (Supplementary Fig.

S13). Among these four genes, three are located in the ancient strata that we previously proposed (Supplementary Fig. S14) and are candidate genes for sex determination in seabuckthorn.

The gene *Hsam2h02g1248*, a homolog of *FAR*, was highly and specifically expressed (TPM > 100) during the male flower bud development phase in *H. gyantsensis*, and it was minimally expressed in stems or leaves. This expression profile resembles that of *AtFAR2* in *Arabidopsis thaliana*, which is thought to be involved in male sterility [32]. Additionally, the pseudogenization of *Hsam2h02g1248* was observed in the female counterparts of both *H. gyantsensis* and *H. salicifolia*, which resulted in the loss of its original function (Supplementary Fig. S15). The role of this gene and its pattern of expression are intimately linked to the masculinization of seabuckthorn, which makes it a male-stimulating factor (M). *Hsam2h02g1315* and *Hsam2h02g1262* are male-specific and lack X-SLR homologs in seabuckthorn (Supplementary Fig. S16&S17). Autosomal homologs are present, indicating that they are possibly derived from autosomal duplications in the Y chromosome. *Hsam2h02g1315* is a homologous gene of FD, and the homologous gene of *Hsam2h02g1262* in *A. thaliana* is *AtRRP44A*. Both of them are closely related to flower development [33,34]. One of these two genes might be a female suppressor factor in seabuckthorn, yet additional experiments are needed to verify this possibility.

Discussion

Sexual differentiation is a critically important component of the reproductive strategy of

members of the genus *Hippophae*, and studies of sexual differentiation can provide insights into the physiological and molecular mechanisms by which seabuckthorn adapts to different environments, which have implications for ongoing efforts to genetically improve seabuckthorn and regulate the sex determination process. However, the lack of complete genomic information for male seabuckthorn species limits studies of sexual differentiation. The absence of male genomic data has also impeded studies of the molecular mechanisms of sex determination in seabuckthorn, the gene expression regulatory networks during sexual differentiation, and the genetic basis of sex-specific traits. A recent study of *H. tibetana* has indicated that its sex chromosomes are located on Chr02; however, the SDR interval was not determined in this study [24]. We sequenced the genomes of male *H. gyantsensis* and both male and female *H. salicifolia*; we obtained four sets of genomic data (including two haplotype datasets for male *H. salicifolia*).

H. salicifolia and *H. gyantsensis* have recently undergone two WGD events, and this finding is consistent with that observed in other species within the family Elaeagnaceae. The recent WGD event is specific to Elaeagnaceae. Chromosomal breakage and fusion events following WGD events have been more common in *Hippophae* than in *Elaeagnus*, which has also experienced two WGD events [28,29]. Previous studies have indicated that chromosomal breakage and fusion play a major role in the formation of sex chromosomes [35]. These events can lead to substantial changes in genome structure, which affects gene expression and function and can sometimes even result in the formation of new species [36]. In our study, the sex chromosomes of both *H. salicifolia* and *H. gyantsensis* were located on chromosome Chr02. Chr02 in *Hippophae* species was derived from the fusion of two distinct ancestral chromosomes (A and B). One end of the fusion fragment of chromosome A was located near the centromeric

region of the chromosome. When it fused with chromosome B, it introduced a large number of repetitive sequences, which faciltated the subsequent cessation of recombination in this region to form the SLR [37]. The SLRs of papaya and kiwifruit have evolved from the pericentromeric regions [38–40]. Throughout the evolutionary process, WGD events, chromosomal rearrangements, and the lack of recombination around the centromeric regions have facilitated the origin of sex chromosomes in *Hippophae* species.

Plant sex chromosomes are derived from a pair of autosomes, and recombination suppression is essential for the evolution of sex chromosomes. Chromosomal rearrangements, heterochromatinization, the accumulation of repetitive sequences, and DNA methylation can all lead to recombination suppression [41,42], chromosomal rearrangements are the main cause of recombination suppression [2,43]. In papaya, two large inversions on the Y chromosome have promoted the differentiation of XY, which is the main cause of recombination suppression in the XY chromosomes [39]. In our study, the evolution of the sex chromosomes in *H. salicifolia* appears to be related to the frequent chromosomal rearrangements in the X- and Y-SLRs. Analysis of the arrangement and orientation of homologous blocks within the SLRs revealed that the Y-SLR structure of *H. salicifolia* was more conserved, with multiple blocks in the X-SLR undergoing chromosomal inversions or translocations and one block in the Y-SLR undergoing translocation. These structural variations are closely related to the spread of recombination suppression. Based on the structural variations and the Ks values of the evolutionary strata, we simulated the evolution of the sex chromosomes in *H. salicifolia*: the oldest structural variations occurred over a narrow range near the centromere, which promoted the spread of recombination suppression and the formation of ancient strata. Subsequently,

large-scale structural variations occurred around the ancient strata, which promoted the spread of recombination suppression and the formation of new strata. X-linked structural variations do not frequently drive the evolution of sex chromosomes; rearrangements in the Y-SLR have been identified in most sex chromosomes studied to date, such as inversions in papaya and willows in the Y chromosome [44,45]. However, recent studies of Silene latifolia have shown that recombination suppression may stem from an inversion in the X-SLR [46]. Many current theories of sex chromosome evolution are based on the premise of Y-linked inversions [46]; however, the results of our study indicate that X-linked structural variations might be selected and fixed to suppress recombination between XY chromosomes.

Sex-determining genes are the main drivers of reproductive organ differentiation in dioecious plants. However, these genes have been isolated and identified in only a handful of species, including kiwifruit, persimmon, asparagus, and poplar [18,19,47,48]. We were unable to identify homologs of these known sex-determining genes in the SDRs of seabuckthorn, indicating that a unique mechanism promotes maleness and inhibits femaleness in this genus. Typically, sex-determining genes are specifically expressed in male flowers and exhibit a phylogenetic pattern in which homologs from various species cluster together. Within the sea buckthorn's MSY region, we identified three genes that appear consistent with these criteria. Notably, *Hsam2h02g1248* is a candidate male-promoting factor, and its Arabidopsis homolog, *FAR2*, is a known male sterility factor [32]. This gene also shares significant evolutionary similarity with the kiwifruit sex-determining gene [19], *FrBy*, which is present during the andromonoecious phase and is differentially maintained in males after sexual differentiation. Our findings are consistent with this pattern, with *Hsam2h02g1248* persisting in males and

losing functionality in females after sexual differentiation. Under normal circumstances, the sex determination region has two genes that jointly determine sexual differentiation. Based on the characteristics of SLRs, we speculate that seabuckthorn should belong to a species with dual-gene sex determination, that is, in addition to a male-promoting factor, there is also a female-suppressing factor. *Hsam2h02g1315* and *Hsam2h02g1262* are our inferred female-suppressing candidate genes. These two genes lack homologs in the X-SLR but have homologs on the autosomes, suggesting they may have originated from autosomal gene duplication. Gene duplication plays a key role in the formation of female-inhibiting factors; for example, the female-inhibiting factor *SyGl* in kiwifruit is derived from an autosomal gene duplication that changes its expression pattern to inhibit carpel development [18]. The sex-determining genes *FERR-R* and *ARR17* in poplar species also arise from duplication [47,49]. *Hsam2h02g1315* and *Hsam2h02g1262* are both related to flower development. Additional experiments are needed to determine whether they have acquired new functions after duplication.

Material and Methods

Sample collection

All genomic and transcriptomic samples required for this study of *H. gyantsensis* were obtained from Jiangzi County, Shigatse City, Tibet Autonomous Region, China. The genomic and transcriptomic samples of *H. salicifolia* were collected from Gyirong County, Tibet Autonomous Region, China. For genomic sequencing, we collected leaf samples from one male *H. gyantsensis*, one female, and one male *H. salicifolia*. To analyze the differences in gene expression between males and females, we collected mixed female and male buds from both *H.*

gyantsensis and *H. salicifolia*.

Genome sequencing

Genomic DNA samples for short-read sequencing were extracted from the leaves of two-week-old seedlings using the CTAB method [50]. Libraries were constructed using the MGIEasy Universal DNA Library Prep Kit V1.0 (CAT#1000005250, MGI) per the standard protocol. The qualified libraries were sequenced on the DNBSEQ-T7RS platform. Total RNA was extracted by grinding tissue using the CTAB-LiCl method (Plant) on dry ice and processed per the manufacturer's protocol. After meeting the quality control standards, an appropriate amount of total RNA was used to construct a library for sequencing using the DNBSEQ-T7RS platform.

ONT regular DNA was extracted using the Grandomics Genomic DNA Kit per the manufacturer's protocol. DNA samples were accurately quantified using a Qubit® 3.0 Fluorometer (Invitrogen, USA). Long, size-selected DNA fragments were then extracted using the PippinHT system (Sage Science, USA). DNA was repaired and adapters were attached to the ends using an SQK-LSK110 kit. The concentrations of library fragments were quantified using a Qubit® 3.0 Fluorometer. The DNA library was loaded into the primed Nanopore PromethION sequencer (Oxford Nanopore Technologies, UK) flow cell. After DNA extraction, the SMRTbell target size library was constructed using the 15 kb preparation solution according to PacBio's standard protocol (Pacific Biosciences, CA, USA) for HiFi sequencing. Sequencing was conducted on the Grandomics PacBio Sequel II instrument. Genomic DNA was extracted for the Hi-C library from leaves. Next, we constructed the Hi-C library and obtained sequencing

data using the Illumina MGI-2000 platform.

Genome Size Assessment and Genome Assembly

The K-mer (k=21) frequency table of clean NGS reads was generated using the Jellyfish (RRID:SCR_005491) v2.3.0 [51] program, and the genome size and heterozygosity of the three seabuckthorn species were assessed using GenomeScope (RRID:SCR_017014) v2.0 [52]. NextDenovo (RRID:SCR_025033) v2.4.0 was used to generate the preliminary assembly from the ONT clean sequencing data for male *H. gyantsensis* and female *H. salicifolia*. NextPolish (RRID:SCR_025232) v1.3.1 [53] was used to perform three rounds of ONT and NGS iterative error correction on the initially assembled genomes. For male *H. salicifolia* sequenced using HiFi sequencing, raw BAM files obtained from sequencing were converted into gz format using SAMTOOLS (RRID:SCR_002105) v1.18 [54], and Hifiasm (RRID:SCR_021069) v0.19.9-r616 [55] was used to assemble contigs by integrating HiFi and HiC reads. After obtaining the preliminary assemblies of the three seabuckthorn genomes, Minimap2 (RRID:SCR_018550) v2.28-r1209 [56] was used to compare the third-generation sequencing data with the genome, and the read coverage depth and coverage breadth for each contig were calculated. Redundant sequences and small genome sequences were identified and removed based on read coverage using Purge_Dups (RRID:SCR_021173) v1.2.5 [57] software. Cleaned Hi-C reads were input into Juicer (RRID:SCR_017226) v1.6 software [58] for ALLHiC (RRID:SCR_022750) v210623 [59] chromosome construction. Manual adjustments for inversions and shifts in the assembly were made using Juicebox (RRID:SCR_021172) [60] to obtain three chromosome-level seabuckthorn genomes. The final Hi-C contact map was visualized using HiCExplorer

(RRID:SCR_022111) v3.7.3 [61]. To improve the quality of the chromosome assembly, the male *H. salicifolia* genome was corrected using NextPolish2 [62], using clean HiFi and NGS data as input. The completeness of the genome assembly was evaluated using BUSCO (RRID:SCR_015008) v5.7.0 [63] and the embryophyta-odb10 dataset.

Genome annotation

Repetitive sequences of the three seabuckthorn genomes were annotated using the Extensive de novo TE Annotator (EDTA (RRID:SCR_022063) v2.1.2 [64]). After obtaining the EDTA-annotated transposable element (TE) library, TEsorter v1.4.6 [65] was used to reclassify the "LTR-unknown", followed by deepTE [66] classification for the "TEsorter-unknown". Finally, the three obtained TE databases were merged, and the repetitive sequences were masked using RepeatMasker (RRID:SCR_012954) v4.1.2-p1 [67].

Homology-based protein alignment, *de novo* prediction, and transcriptome prediction were used for gene structure annotation. HISAT2 (RRID:SCR_015530) v2.2.1 [68] was used to align clean reads from the transcriptome to the genome, and the resulting alignment files were used as input for Braker3 [69,70]. GeneMark-ET [71] was used for gene structure identification, and Augustus was used for model training to obtain high-confidence gene structures based on the transcript and *de novo* prediction results. *H. rhamnoides* protein sequences were input into Braker3, and GeneMark-EP [72] combined with ProtHint was used for sequence alignment. Augustus was used for model training to obtain high-confidence gene structures based on the known proteins and *de novo* prediction results. The results obtained via Braker3 were then integrated using TSEBRA [73] (default parameters); this was followed by quality filtering and

normalization using scripts from the MAKER (RRID:SCR_005309) package v3.01.04 [74], which ultimately generated a gff annotation file that describes genetic structural information. TBtools (RRID:SCR_023018) [75] was used to extract the longest transcript, gffread (RRID:SCR_018965) v0.12.8 [76] was used to extract the corresponding cds sequence, and then SeqKit (RRID:SCR_018926) [77] was used to translate it into a protein sequence. BUSCO (RRID:SCR_015008) v5.7.0 [63] was used to assess the integrated annotation proteins.

InterProScan (RRID:SCR_005829) v5 [78] was used to conduct a search of the annotated genes with the parameters set to '-appl Pfam, CDD'. The predicted genes were functionally annotated by scanning the NCBI non-redundant protein (nr), TrEMBL, and Swiss-Prot [79] databases using DIAMOND (RRID:SCR_009457) v2.0.15 [80] with default parameters. eggNOG-mapper (RRID:SCR_021165) [81] with default parameters was used to obtain Kyoto Encyclopedia of Genes and Genomes (KEGG (RRID:SCR_012773) [82]) and Gene Ontology (GO (RRID:SCR_017505) [83]) annotations.

Comparative genomics analysis

Protein sequences of *H. rhamnoides*, *H. salicifolia*, *H. gyantsensis*, Fructus Hippophae, *H. tibetana*, *Elaeagnus moorcroftii*, *Elaeagnus mollis*, *Ziziphus jujuba*, *Fragaria daltoniana*, *Vitis vinifera*, *Morus notabilis*, *Cannabis sativa*, and *Rhamnella rubrinervis* were selected for gene family analysis. OrthoFinder (RRID:SCR_017118) v2.5.5 [84] was used to identify orthologous groups with the parameters set as '-M msa', and single-copy genes were used to construct a phylogenetic tree. The SeqKit (RRID:SCR_018926) [77] tool was used to extract the coding sequences of single-copy orthologous genes, and they were aligned using MUSCLE

(RRID:SCR_011812) v3.8.31 [85]. The SeqKit (RRID:SCR_018926) [77] tool was used to concatenate the sequences into supergenes, which were then trimmed using trimAl (RRID:SCR_017334) v1.4 [86] (-gt 0.6 -cons 60). The trimmed sequences were used to construct a phylogenetic tree using the maximum likelihood method in RAxML (RRID:SCR_006086) v1.1.0 software [87]. Three fossil calibration time points were obtained from the TimeTree (RRID:SCR_021162) database [88]: *V. vinifera* and *Z. jujuba* [109.8 to 122.4 million years ago (mya)], *Z. jujuba* and *C. sativa* (68.5 to 85.2 mya), and *C. sativa* and *M. notabilis* (48.9 to 70.9 mya). The MCMCtree program in the PAML (RRID:SCR_014932) v.4.10.0 software package [89] was used to estimate the divergence times of each node in the phylogenetic tree obtained in the previous step. The phylogenetic tree with divergence times and the sorted gene family results (i.e., gene families with significant differences in copy number were removed) were used to construct a phylogenetic tree containing information on gene family expansions and contractions using the CAFE (RRID:SCR_005983) V5 [90] program.

We used WGDI v0.6.5 [91] to detect whole-genome duplication events. DIAMOND (RRID:SCR_009457) v2.0.15 [80] was used to identify homologous genes with an e-value that did not exceed 1e-5. WGDI was used to identify collinear genes with the parameter '-icl'. Next, the 'ks' parameter in WGDI was modified to calculate Ks values, and the 'bi' and 'c' parameters were modified to filter the collinear results. Finally, the 'kp' parameter was modified to calculate the Ks peak, and the 'kf' parameter was used to fit and visualize the results. JCVI (RRID:SCR_021641) [92] (MCscan Python version) was used for whole-genome collinearity analysis between *Hippophae* and *Elaeagnus* plants.

Sex determination interval and characteristics

H. gyantsensis resequencing data were compared with the female and male *H. gyantsensis* reference genomes using BWA-MEM2 (RRID:SCR_022192) v2.2.1 [93] software. Similarly, second-generation sequencing data of female and male *H. salicifolia* were also compared with the Hap1 and Hap2 reference genomes of *H. salicifolia*. Based on the comparison results, a window was established within an interval of 50 kb, with a sliding window of 5 kb for stepwise progression. BEDTools (RRID:SCR_006646) v2.30.0 [94] was used to calculate the coverage depth of each window, and the R package ggplot2 (RRID:SCR_014601) [95] was used to visualize the results. The chromosomes and intervals of the SDR were located assuming that male-specific regions have no female read coverage and male read coverage was half that of other regions. After obtaining the initial SLRs, Integrative Genomics Viewer(IGV) (RRID:SCR_011793) software [96] was used to view the precise SLR range by comparing different regions. The microsynteny analysis of the Y-SLR interval genes of *H. salicifolia* and the Y chromosome of *H. gyantsensis* was performed using JCVI (RRID:SCR_021641)[92] to determine the sex-linked genes of *H. gyantsensis*.

We mainly used JCVI (RRID:SCR_021641) [92] and BLASTP (RRID:SCR_001010) v2.14.0+ [97] to analyze the origin of genes within the X- and Y-SLRs of *H. salicifolia*. First, the X- and Y-SLRs were compared with themselves and two *Elaeagnus* species for collinearity analysis. Genes identified in the homologous regions were classified as “ancestral” genes. A search of the remaining genes was conducted using BLASTP (RRID:SCR_001010) v2.14.0+

[97] to identify homologous genes. Genes with homologous genes on the autosomes were referred to as “acquired” genes, and genes without homologous genes were referred to as “specific” genes. Finally, genes that were the same in both the original and "foreign" genes were classified as “duplicate” genes.

Sex chromosome evolution of H. salicifolia and H. gyantsensis

For evolutionary analysis, we first used JCVI (RRID:SCR_021641) [92] to analyze the collinearity Chr02 of *H. salicifolia* with *H. gyantsensis*, *H. rhamnoides*, *E. moorcroftii*, *E. mollis*, *R. rubrinervis*, and *Z. jujuba*. JCVI (RRID:SCR_021641) [92] was also used to analyze the microsynteny of the SLR regions in *H. salicifolia* with *H. gyantsensis*, *H. rhamnoides*, Fructus Hippophae, *E. moorcroftii*, and *E. mollis*. Based on the identified homologous blocks, we analyzed block rearrangements in *Hippophae* and *Elaeagnus* plants. To analyze the substitution rates of X- and Y-linked genes, we created pairwise alignments including X-linked and Y-linked homologous genes, and then used ParaAT.pl v2.0 [98] and KaKs_Caculater v3.0 [99] to calculate synonymous and non-synonymous substitution rates. Different phylogenetic patterns were observed for genes in the "ancient" and "recent" regions of the sex chromosomes. Therefore, OrthoFinder (RRID:SCR_017118) v2.5.5 [84] was used to identify single-copy orthologous genes within the seabuckthorn SLRs, and MAFFT (RRID:SCR_011811) v7.520 [100] was used to align the sequences in each set of single-copy orthologs. We also conducted searches of the corresponding chromosomes of *E. moorcroftii* and *E. mollis* using BLASTP [97] to identify corresponding homologous genes. Finally, we used IQ-TREE2 v2.2.0 [101] to

generate the gene trees with 1,000 bootstrap replicates.

Screening of sex-determining candidate genes

To identify candidate genes involved in sex determination, we first conducted an analysis of differentially expressed genes between males and females. Fastp (RRID:SCR_016962) [102] software was used to filter the transcriptome data of male and female flower buds of *H. gyantsensis* and *H. salicifolia*, and STAR (RRID:SCR_004463) [103] was used to align them with the male *H. salicifolia* Hap2 genome; RSEM (RRID:SCR_000262) [104] software was used to calculate expression levels. After identifying genes specifically expressed in males, we further screened them based on their phylogenetic positions relative to those of homologous genes. First, we extracted the protein sequences of genes specifically expressed in males in the SDR region of *H. salicifolia* as queries and used data from *H. rhamnoides*, *H. tibetana*, male *H. gyantsensis*, female *H. gyantsensis*, male *H. salicifolia*, female *H. salicifolia*, and Fructus Hippophae as protein databases; *E. moorcroftii*, *E. mollis*, and *A. thaliana* were used as outgroups in homologous gene searches. Based on the obtained BLASTP (RRID:SCR_001010) v2.14.0+ [97] alignment results, we retained the best-aligned sequences from one species and used MAFFT (RRID:SCR_011811) v7.520 [100] software for sequence comparison. Gene trees were constructed using IQ-TREE2 v2.2.0 [101], and Figtree was used to visualize the trees.

Acknowledgements

This research was supported by the Second Tibetan Plateau Scientific Expedition and Research (STEP) program (2019QZKK0502), Science and Technology Plan Project of Tibet Autonomous Region (XZ202401ZY0006), Science and Technology Program Projects of Shigatse City (RKZ2023ZY-03), Yunling Scholar Project to Yang Yongping.

Additional Files

Supplementary Fig. S1. Distribution of K-mer (K = 21) frequency in sequencing reads of the three seabuckthorn plants. (a) Distribution of K-mer frequency for male *H. salicifolia* from Illumina sequencing. (b) Distribution of K-mer frequency for female *H. salicifolia* from Illumina sequencing. (c) Distribution of K-mer frequency for male *H. gyantsensis* from Illumina sequencing.

Supplementary Fig. S2. Hi-C heatmap of chromosomes for male *H. gyantsensis* (a), female *H. salicifolia* (b), Hap1 (c), and Hap2 (d) of male *H. salicifolia*.

Supplementary Fig. S3. Circos plot of the genomic landscape of male *H. gyantsensis*. (a) Pseudochromosome. (b) Gene density. (c) Repeat sequences density. (d) Gypsy density. (e) Copia density. (f) GC content.

Supplementary Fig. S4. Circos plot of the genomic landscape of female *H. salifolia*. (a) Pseudochromosome. (b) Gene density. (c) Repeat sequences density. (d) Gypsy density. (e) Copia density. (f) GC content.

Supplementary Fig. S5. Statistics of orthogroups in different plants defined by OrthoFinder.

Supplementary Fig. S6. Identification of the 2Y-SLR of *H. gyantsensis*. The next-generation sequencing data of male and female *H. gyantsensis* were visualized in the coverage depth of Chr02. The red line represents the depth of female coverage, and the blue line represents the depth of male coverage.

Supplementary Fig. S7. Density plot of repetitive sequences on Chr02 for Hap1 (a) and Hap2(b). Pink dots represent the density of pseudoautosomal repeats, and blue dots represent the density of repeats in sex-linked regions.

Supplementary Fig. S8. Synteny relationship between Chr02 of Hap2 and its closely related species: (a) Syntenic point map of Hap2 Chr02 and whole genome of *H. gyantsensis* (b) Syntenic point map of Hap2 Chr02 and whole genome of *E. moorcroftii*.

Supplementary Fig. S9. Synteny relationship between chromosome 2 of Hap2 and its closely related species: (a) Synteny relationship between Chr02 of Hap2 and *Z. jujuba*. (b) Synteny relationship between Chr02 of Hap2 and *R. rubrinervis*.

Supplementary Fig. S10. Microsynteny of homologous blocks between the Y-SLR of Hap2 and its closely related species. (a) *E. mollis* (b) *E. moorcroftii* (c) *H. rhamnoides* (d) Fructus Hippophae (e) female *H. gyantsensis* (f) male *H. gyantsensis* (g) Hap1.

Supplementary Fig. S11. The Ks values of XY homologous gene pairs within the Y-SLR are mapped to the positional coordinates of the Y chromosome. Blue represents gene pairs within stratum 1, red represents gene pairs within stratum 2-1, and yellow represents gene pairs within stratum 2-2.

Supplementary Fig. S12. Among the 11 male-specifically expressed genes, the phylogenetic trees of two genes are not consistent with the expected phylogenetic structure. 2Y-SLR

represents the genes within the Y-SLR, and 2X-SLR represents the genes within the X-SLR. Furthermore, the genes within the Y-SLR are marked in red. (a) *Hsam2h02g1340* (b) *Hsam2h02g1384*.

Supplementary Fig. S13. Among the 11 male-specifically expressed genes, the phylogenetic trees of four genes are consistent with the expected phylogenetic structure. 2Y-SLR represents the genes within the Y-SLR, and 2X-SLR represents the genes within the X-SLR. Furthermore, the genes within the Y-SLR are marked in red. (a) *Hsam2h02g1248* (b) *Hsam2h02g1262* (c) *Hsam2h02g1315* (d) *Hsam2h02g1436*.

Supplementary Fig. S14. The distribution of the four candidate genes within the SLR; three genes are located in the presumed ancient strata, one gene (*Hsam02g1436*) is close to the PAR region.

Supplementary Fig. S15. The sequence alignment of *Hsam2h02g1248* with its homologous genes in the female- and male-specific linkage region of *H. salicifolia* and *H. gyantsensis* indicates that pseudogenization has occurred in the homologous genes of females.

Supplementary Fig. S16. Alignments of the Illumina reads from male and female individuals to the gene *Hsam2h02g1315*.

Supplementary Fig. S17. Alignments of the Illumina reads from male and female individuals to the gene *Hsam2h02g1262*.

Supplementary Table S1. Sequencing statistics of male *H. gyantsensis,* female *H. salicifolia* and male *H. salicifolia.*

Supplementary Table S2. *Statistics of Hi-C reads mapping for male H. gyantsensis, female H. salicifolia and male H. salicifolia.*

Supplementary Table S3. Summary of male and female seabuckthorn genome assemblies.

Supplementary Table S4. Quality assessment of the completeness of female and male genome assemblies using the BUSCO tool.

Supplementary Table S5. Repeat sequence annotation statistics.

Supplementary Table S6. Functional annotation of predicted protein-coding genes of the male and female seabuckthorn genomes.

Supplementary Table S7. Quality assessment of the completeness of female and male gene sets with the BUSCO tool.

Supplementary Table S8. The classification of gene origins within the X- and Y-SLR.

Supplementary Table S9. The collinearity relationship between the X-SLR of *H. salicifolia* and the homologous regions of closely related species.

Supplementary Table S10. The Ks values of homologous genes in different blocks within the SLRs of *H. salicifolia*.

Supplementary Table S11. Statistics of transcriptome sequencing of female and male mixed flower buds in *H. gyantsensis* and *H. salicifolia*.

Supplementary Table S12. TPM values of genes in the SLR of *H. salicifolia* across different tissues in males and females.

Abbreviations

bp: base pairs; BUSCO: Benchmarking Universal Single-Copy Orthologs; CTAB: cetyl trimethyl ammonium bromide; Gb: gigabase pairs; HiC: high-throughput/resolution

chromosome conformation capture; Mb: megabase pairs; Mya: million years ago; ONT: Oxford Nanopore Technologies; Pacbio: Pacific Biosciences; SLR: sex-link region; SDR: sex-determining region; MSY: male-specific region; Ks: synonymous substitutions per synonymous site; TPM: transcripts per million; NGS: next-generation sequencing; PAR: pseudoautosomal region;

Author Contributions

YPY, YQY and SHY designed the research. MYC and XYY performed the research. MYC, XYY, XL and ZLQ analyzed the data. MYC and YQY wrote the paper.

Competing Interests

The authors declare that they have no competing interests.

Ethics Statement

All plant molecular materials and specimens were collected with permission.

Data Availability

The sequencing data that support the findings of this study are openly available in the NCBI Sequence Read Archive (SRA) under BioProject accession number PRJNA1166656. The genome assembly and annotation data of the three seabuckthorns (https://doi.org/10.6084/m9.figshare.27093856.v1) have been deposited in FigShare.

References

1. Charlesworth D, Charlesworth B, Marais G. Steps in the evolution of heteromorphic sex chromosomes. *Heredity*. 2005;95(2):118-128. doi:10.1038/sj.hdy.6800697.

2. Charlesworth B. The evolution of sex chromosomes. *Science*. 1991;251(4997):1030-1033. doi:10.1126/science.1998119.

3. Charlesworth D. Plant sex determination and sex chromosomes. *Heredity*. 2002;88(2):94-101. doi:10.1038/sj.hdy.6800016.

4. Aryal R, Ming R. Sex determination in flowering plants: papaya as a model system. *Plant Sci.* 2014;217-218:56-62. doi:10.1016/j.plantsci.2013.10.018.

5. Ming R, Bendahmane A, Renner SS. Sex chromosomes in land plants. *Annu Rev Plant Biol.* 2011;62:485-514. doi:10.1146/annurev-arplant-042110-103914.

6. Han W, Liu L, Wang J, et al. Ancient homomorphy of molluscan sex chromosomes sustained by reversible sex-biased genes and sex determiner translocation. *Nat Ecol Evol.* 2022;6(12):1891-1906. doi:10.1038/s41559-022-01898-6.

7. Wright AE, Dean R, Zimmer F, Mank JE. How to make a sex chromosome. *Nat Commun.* 2016;7:12087. doi:10.1038/ncomms12087.

8. Westergaard M. The mechanism of sex determination in dioecious flowering plants. *Adv Genet.* 1958;9:217-281. doi:10.1016/s0065-2660(08)60163-7.

9. Renner SS. The relative and absolute frequencies of angiosperm sexual systems: dioecy, monoecy, gynodioecy, and an updated online database. *Am J Bot*. 2014;101(10):1588-1596. doi:10.3732/ajb.1400196.

10. Yu Q, Barrett SCH, Wang X-J, et al. Sexual dimorphism, temporal niche differentiation, and evidence for the Jack Sprat effect in an annual dioecious plant. *Journal of Systematics and Evolution*. 2022;60:1078-1091. doi: 10.1111/jse.12753.

11. Onyekwelu SS, Harper JL. Sex ratio and niche differentiation in spinach (*Spinacia oleracea* L.). *Nature*. 1979;282:609-611. doi: 10.1038/282609a0.

12. Sánchez-Vilas J, Pannell JR. Differential niche modification by males and females of a dioecious herb: extending the Jack Sprat effect. *J Evol Biol.* 2010;23(10):2262-2266. doi:10.1111/j.1420-9101.2010.02089.x.

13. Ohta K, Shibata T, Nicolas A. Changes in chromatin structure at recombination initiation sites during yeast meiosis. *EMBO J.* 1994;13(23):5754-5763. doi:10.1002/j.1460-2075.1994.tb06913.x.

14. Rifkin JL, Beaudry FEG, Humphries Z, et al. Widespread Recombination Suppression Facilitates Plant Sex Chromosome Evolution. *Mol Biol Evol.* 2021;38(3):1018-1030. doi:10.1093/molbev/msaa271

15. Ellegren H, Parsch J. The evolution of sex-biased genes and sex-biased gene expression. *Nat Rev Genet*. 2007;8(9):689-698. doi:10.1038/nrg2167.

16. Berner D, Roesti M. Genomics of adaptive divergence with chromosome-scale heterogeneity in crossover rate. *Mol Ecol.* 2017;26(22):6351-6369. doi:10.1111/mec.14373.

17. Akagi T, Henry IM, Tao R, Comai L. Plant genetics. A Y-chromosome-encoded small RNA acts as a sex determinant in persimmons. *Science.* 2014;346(6209):646-650. doi:10.1126/science.1257225.

18. Akagi T, Henry IM, Ohtani H, et al. A Y-Encoded Suppressor of Feminization Arose via Lineage-Specific Duplication of a Cytokinin Response Regulator in Kiwifruit. *Plant Cell.* 2018;30(4):780-795. doi:10.1105/tpc.17.00787.

19. Akagi T, Pilkington SM, Varkonyi-Gasic E, et al. Two Y-chromosome-encoded genes determine sex in kiwifruit. *Nat Plants.* 2019;5(8):801-809. doi:10.1038/s41477-019-0489-6.

20. Yu W, Du Y, Li S, et al.. Sea buckthorn-nutritional composition, bioactivity, safety, and applications: A review. *Journal of Food Composition and Analysis*. 2024; doi: 10.1016/j.jfca.2024.106371.

21. Ling N, Tian H, Wang Q, et al. Advance in *Hippophae rhamnoides* polysaccharides: Extraction, structural characteristics, pharmacological activity, structure-activity relationship and application. *Int J Biol Macromol.* 2024;270(Pt 2):132420. doi:10.1016/j.ijbiomac.2024.132420.

22. Dolkar P, Dolkar D, Kant A, Chaurasia OP, Stobdan T. Gender differences in phenotypic plasticity and adaptive response of Seabuckthorn (*Hippophae rhamnoides* L.) along an altitudinal gradient in trans-Himalaya. *JBR*. 2018, 9 (1): 1-10. doi: 10.3233/JBR-180294.

23. Puterova J, Razumova O, Martinek T, et al. Satellite DNA and Transposable Elements in Seabuckthorn *(Hippophae rhamnoides*), a Dioecious Plant with Small Y and Large X Chromosomes. *Genome Biol Evol*. 2017;9(1):197-212. doi:10.1093/gbe/evw303.

24. Wang R, Wu B, Jian J, et al. How to survive in the world's third poplar: Insights from the genome of the highest altitude woody plant, *Hippophae tibetana* (Elaeagnaceae). *Front Plant Sci.* 2022;13:1051587. doi:10.3389/fpls.2022.1051587.

25. Wu Z, Chen H, Pan Y, et al. Genome of *Hippophae rhamnoides* provides insights into a conserved molecular mechanism in actinorhizal and rhizobial symbioses. *New Phytol.* 2022;235(1):276-291. doi:10.1111/nph.18017.

26. Yu L, Diao S, Zhang G, et al. Genome sequence and population genomics provide insights into chromosomal evolution and phytochemical innovation of *Hippophae rhamnoides*. *Plant Biotechnol J*. 2022;20(7):1257-1273. doi:10.1111/pbi.13802.

27. Chen M, Yang D, Yang S, et al. Chromosome-level genome assembly of *Hippophae gyantsensis*. Sci Data. 2024;11(1):126. doi:10.1038/s41597-024-02909-w.

28. Fu X, Wu J, Ma X, Li K, Zhang H, Wu S, et al. The Chromosome-Level Genome of *Elaeagnus moorcroftii* Wall., an Economically and Ecologically Important Tree Species in Drylands. *Diversity*. 2022, 14 (6): 468-468. doi: 10.3390/d14060468.

29. Wu J, Zhang L, Ma X, et al. The evolutionary significance of whole genome duplications in oil biosynthesis of oil crops. *Hortic Res*. 2024;11(7):uhae156. doi:10.1093/hr/uhae156.

30. ojas MG, Pozzi E, Ramasamy R. Unlocking the mystery of the human Y chromosome. *Nat Rev Urol*. 2024;21(2):65-66. doi:10.1038/s41585-023-00826-y.

31. Bergero R, Charlesworth D. The evolution of restricted recombination in sex chromosomes. *Trends Ecol Evol.* 2009;24(2):94-102. doi:10.1016/j.tree.2008.09.010.

32. Doan TT, Carlsson AS, Stymne S, Hofvander P. Biochemical characteristics of *AtFAR2*, a fatty acid reductase from *Arabidopsis thaliana* that reduces fatty acyl-CoA and -ACP substrates into fatty alcohols. Acta Biochim Pol. 2016;63(3):565-570. doi:10.18388/abp.2016_1245.

33. Abe M, Kobayashi Y, Yamamoto S, et al. *FD*, a bZIP protein mediating signals from the

floral pathway integrator *FT* at the shoot apex. *Science.* 2005;309(5737):1052-1056. doi:10.1126/science.1115983.

34. Kitagawa M, Wu P, Balkunde R, Cunniff P, Jackson D. An RNA exosome subunit mediates cell-to-cell trafficking of a homeobox mRNA via plasmodesmata. *Science*. 2022;375(6577):177-182. doi:10.1126/science.abm0840.

35. Huang Z, De O Furo I, Liu J, et al. Recurrent chromosome reshuffling and the evolution of neo-sex chromosomes in parrots. *Nat Commun.* 2022;13(1):944. doi:10.1038/s41467-022-28585-1.

36. Damas J, Corbo M, Lewin HA. Vertebrate Chromosome Evolution. *Annu Rev Anim Biosci*. 2021;9:1-27. doi:10.1146/annurev-animal-020518-114924.

37. Haenel Q, Laurentino TG, Roesti M, Berner D. Meta-analysis of chromosome-scale crossover rate variation in eukaryotes and its significance to evolutionary genomics. *Mol Ecol.* 2018;27(11):2477-2497. doi:10.1111/mec.14699.

38. Zhou R, Macaya-Sanz D, Rodgers-Melnick E, et al. Characterization of a large sex determination region in *Salix purpurea* L. (Salicaceae). *Mol Genet Genomics*. 2018;293(6):1437-1452. doi:10.1007/s00438-018-1473-y.

39. Wu M, Moore RC. The Evolutionary Tempo of Sex Chromosome Degradation in *Carica papaya*. *J Mol Evol*. 2015;80(5-6):265-277. doi:10.1007/s00239-015-9680-1.

40. Pilkington SM, Tahir J, Hilario E, et al. Genetic and cytological analyses reveal the recombination landscape of a partially differentiated plant sex chromosome in kiwifruit. *BMC Plant Biol*. 2019;19(1):172. doi:10.1186/s12870-019-1766-2.

41. Mariotti B, Manzano S, Kejnovský E, Vyskot B, Jamilena M. Accumulation of Y-specific

satellite DNAs during the evolution of *Rumex acetosa* sex chromosomes. *Mol Genet Genomics*. 2009;281(3):249-259. doi:10.1007/s00438-008-0405-7.

42. Lahn BT, Page DC. Four evolutionary strata on the human X chromosome. *Science*. 1999;286(5441):964-967. doi:10.1126/science.286.5441.964.

43. Natri HM, Merilä J, Shikano T. The evolution of sex determination associated with a chromosomal inversion. *Nat Commun*. 2019;10(1):145. doi:10.1038/s41467-018-08014-y.

44. Yin T, Difazio SP, Gunter LE, et al. Genome structure and emerging evidence of an incipient sex chromosome in Populus. *Genome Res.* 2008;18(3):422-430. doi:10.1101/gr.7076308.

45. Wang J, Na JK, Yu Q, et al. Sequencing papaya X and Yh chromosomes reveals molecular basis of incipient sex chromosome evolution. *Proc Natl Acad Sci*. 2012;109(34):13710-13715. doi:10.1073/pnas.1207833109.

46. Yue J, Krasovec M, Kazama Y, et al. The origin and evolution of sex chromosomes, revealed by sequencing of the *Silene latifolia* female genome. *Curr Biol*. 2023;33(12):2504-2514.e3. doi:10.1016/j.cub.2023.05.046.

47. Xue L, Wu H, Chen Y, et al. Evidences for a role of two Y-specific genes in sex determination in *Populus deltoides*. *Nat Commun*. 2020;11(1):5893. doi:10.1038/s41467-020-19559-2.

48. Harkess A, Huang K, van der Hulst R, et al. Sex Determination by Two Y-Linked Genes in Garden Asparagus. *Plant Cell.* 2020;32(6):1790-1796. doi:10.1105/tpc.19.00859.

49. Müller NA, Kersten B, Leite Montalvão AP, et al. A single gene underlies the dynamic

evolution of poplar sex determination. *Nat Plants.* 2020;6(6):630-637. doi:10.1038/s41477-020-0672-9.

50. Pahlich E, Gerlitz C. A rapid DNA isolation procedure for small quantities of fresh leaf tissue. *Phytochemistry*. 1980;19:11-13 doi: 10.1016/0031-9422(80)85004-7.

51. Marçais G, Kingsford C. A fast, lock-free approach for efficient parallel counting of occurrences of k-mers. *Bioinformatics*. 2011;27(6):764-770. doi:10.1093/bioinformatics/btr011.

52. Vurture GW, Sedlazeck FJ, Nattestad M, et al. GenomeScope: fast reference-free genome profiling from short reads. *Bioinformatics.* 2017;33(14):2202-2204. doi:10.1093/bioinformatics/btx153.

53. Hu J, Fan J, Sun Z, Liu S. NextPolish: a fast and efficient genome polishing tool for long-read assembly. *Bioinformatics*. 2020;36(7):2253-2255. doi:10.1093/bioinformatics/btz891.

54. Danecek P, Bonfield JK, Liddle J, et al. Twelve years of SAMtools and BCFtools. *Gigascience*. 2021;10(2):giab008. doi:10.1093/gigascience/giab008.

55. Cheng H, Jarvis ED, Fedrigo O, et al. Haplotype-resolved assembly of diploid genomes without parental data. *Nat Biotechnol*. 2022;40(9):1332-1335. doi:10.1038/s41587-022-01261-x.

56. Li H. Minimap2: pairwise alignment for nucleotide sequences. *Bioinformatics*. 2018;34(18):3094-3100. doi:10.1093/bioinformatics/bty191

57. Guan D, McCarthy SA, Wood J, et al. Identifying and removing haplotypic duplication in primary genome assemblies. *Bioinformatics*. 2020;36(9):2896-2898.

doi:10.1093/bioinformatics/btaa025.

58. Durand NC, Shamim MS, Machol I, et al. Juicer Provides a One-Click System for Analyzing Loop-Resolution Hi-C Experiments. *Cell Syst*. 2016;3(1):95-98. doi:10.1016/j.cels.2016.07.002

59. Zhang X, Zhang S, Zhao Q, Ming R, Tang H. Assembly of allele-aware, chromosomal-scale autopolyploid genomes based on Hi-C data. *Nat Plants*. 2019;5(8):833-845. doi:10.1038/s41477-019-0487-8.

60. Durand NC, Robinson JT, Shamim MS, et al. Juicebox Provides a Visualization System for Hi-C Contact Maps with Unlimited Zoom. *Cell Syst*. 2016;3(1):99-101. doi:10.1016/j.cels.2015.07.012.

61. Ramírez F, Bhardwaj V, Arrigoni L, et al. High-resolution TADs reveal DNA sequences underlying genome organization in flies. *Nat Commun.* 2018;9(1):189. doi:10.1038/s41467-017-02525-w.

62. Hu J, Wang Z, Liang F, et al. NextPolish2: A Repeat-aware Polishing Tool for Genomes Assembled Using HiFi Long Reads. *Genomics Proteomics Bioinformatics*. 2024;22(1):qzad009. doi:10.1093/gpbjnl/qzad009.

63. Manni M, Berkeley MR, Seppey M, Simão FA, Zdobnov EM. BUSCO Update: Novel and Streamlined Workflows along with Broader and Deeper Phylogenetic Coverage for Scoring of Eukaryotic, Prokaryotic, and Viral Genomes. *Mol Biol Evol*. 2021;38(10):4647-4654. doi:10.1093/molbev/msab199.

64. Ou S, Su W, Liao Y, et al. Benchmarking transposable element annotation methods for creation of a streamlined, comprehensive pipeline. *Genome Biol*. 2019;20(1):275.

doi:10.1186/s13059-019-1905-y.

65. Zhang RG, Li GY, Wang XL, et al. TEsorter: an accurate and fast method to classify LTR-retrotransposons in plant genomes. *Hortic Res*. 2022; doi:10.1093/hr/uhac017.

66. Yan H, Bombarely A, Li S. DeepTE: a computational method for de novo classification of transposons with convolutional neural network. *Bioinformatics*. 2020;36(15):4269-4275. doi:10.1093/bioinformatics/btaa519.

67. Chen N. Using RepeatMasker to identify repetitive elements in genomic sequences. *Curr Protoc Bioinformatics*. 2004;Chapter 4. doi:10.1002/0471250953.bi0410s05.

68. Kim D, Langmead B, Salzberg SL. HISAT: a fast spliced aligner with low memory requirements. *Nat Methods*. 2015;12(4):357-360. doi:10.1038/nmeth.3317.

69. Stanke M, Diekhans M, Baertsch R, Haussler D. Using native and syntenically mapped cDNA alignments to improve de novo gene finding. *Bioinformatics*. 2008;24(5):637-644. doi:10.1093/bioinformatics/btn013.

70. Stanke M, Schöffmann O, Morgenstern B, Waack S. Gene prediction in eukaryotes with a generalized hidden Markov model that uses hints from external sources. *BMC Bioinformatics*. 2006;7:62. doi:10.1186/1471-2105-7-62.

71. Brůna T, Lomsadze A, Borodovsky M. GeneMark-EP+: eukaryotic gene prediction with self-training in the space of genes and proteins. *NAR Genom Bioinform*. 2020;2(2):lqaa026. doi:10.1093/nargab/lqaa026.

72. Gabriel L, Brůna T, Hoff KJ, et al. BRAKER3: Fully automated genome annotation using RNA-seq and protein evidence with GeneMark-ETP, AUGUSTUS and TSEBRA. Preprint. bioRxiv. 2024;2023.06.10.544449. doi:10.1101/2023.06.10.544449.

73. Gabriel L, Hoff KJ, Brůna T, Borodovsky M, Stanke M. TSEBRA: transcript selector for BRAKER. *BMC Bioinformatics*. 2021;22(1):566. doi:10.1186/s12859-021-04482-0.

74. Campbell MS, Holt C, Moore B, Yandell M. Genome Annotation and Curation Using MAKER and MAKER-P. *Curr Protoc Bioinformatics*. 2014;48:4.11.1-4.11.39. doi:10.1002/0471250953.bi0411s48.

75. Chen C, Chen H, Zhang Y, et al. TBtools: An Integrative Toolkit Developed for Interactive Analyses of Big Biological Data. *Mol Plant*. 2020;13(8):1194-1202. doi:10.1016/j.molp.2020.06.009.

76. Pertea G, Pertea M. GFF Utilities: GffRead and GffCompare. F1000Res. 2020;9:ISCB Comm J-304. doi:10.12688/f1000research.23297.2.

77. Shen W, Le S, Li Y, Hu F. SeqKit: A Cross-Platform and Ultrafast Toolkit for FASTA/Q File Manipulation. *PLoS One*. 2016;11(10):e0163962. doi:10.1371/journal.pone.0163962.

78. Jones P, Binns D, Chang HY, et al. InterProScan 5: genome-scale protein function classification. *Bioinformatics*. 2014;30(9):1236-1240. doi:10.1093/bioinformatics/btu031.

79. Boeckmann B, Bairoch A, Apweiler R, et al. The SWISS-PROT protein knowledgebase and its supplement TrEMBL in 2003. *Nucleic Acids Res*. 2003;31(1):365-370. doi:10.1093/nar/gkg095.

80. Buchfink B, Reuter K, Drost HG. Sensitive protein alignments at tree-of-life scale using DIAMOND. *Nat Methods*. 2021;18(4):366-368. doi:10.1038/s41592-021-01101-x.

81. Cantalapiedra CP, Hernández-Plaza A, Letunic I, et al. eggNOG-mapper v2: Functional

Annotation, Orthology Assignments, and Domain Prediction at the Metagenomic Scale. *Mol Biol Evol*. 2021;38(12):5825-5829. doi:10.1093/molbev/msab293.

82. Ogata H, Goto S, Sato K, Fujibuchi W, Bono H, Kanehisa M. KEGG: Kyoto Encyclopedia of Genes and Genomes. *Nucleic Acids Res*. 1999;27(1):29-34. doi:10.1093/nar/27.1.29.

83. Gene Ontology Consortium, Aleksander SA, Balhoff J, et al. The Gene Ontology knowledgebase in 2023. *Genetics*. 2023;224(1):iyad031. doi:10.1093/genetics/iyad031.

84. Emms DM, Kelly S. OrthoFinder: solving fundamental biases in whole genome comparisons dramatically improves orthogroup inference accuracy. *Genome Biol*. 2015;16(1):157. doi:10.1186/s13059-015-0721-2.

85. Edgar RC. MUSCLE: multiple sequence alignment with high accuracy and high throughput. *Nucleic Acids Res*. 2004;32(5):1792-1797. doi:10.1093/nar/gkh340.

86. Capella-Gutiérrez S, Silla-Martínez JM, Gabaldón T. trimAl: a tool for automated alignment trimming in large-scale phylogenetic analyses. *Bioinformatics.* 2009;25(15):1972-1973. doi:10.1093/bioinformatics/btp348.

87. Stamatakis A, Hoover P, Rougemont J. A rapid bootstrap algorithm for the RAxML Web servers. *Syst Biol*. 2008;57(5):758-771. doi:10.1080/10635150802429642.

88. Kumar S, Stecher G, Suleski M, Hedges SB. TimeTree: A Resource for Timelines, Timetrees, and Divergence Times. *Mol Biol Evol*. 2017;34(7):1812-1819. doi:10.1093/molbev/msx116.

89. Yang Z. PAML 4: phylogenetic analysis by maximum likelihood. *Mol Biol Evol*. 2007;24(8):1586-1591. doi:10.1093/molbev/msm088.

90. Mendes FK, Vanderpool D, Fulton B, Hahn MW. CAFE 5 models variation in evolutionary

rates among gene families. *Bioinformatics*. 2021;36(22-23):5516-5518. doi:10.1093/bioinformatics/btaa1022.

91. Sun P, Jiao B, Yang Y, et al. WGDI: A user-friendly toolkit for evolutionary analyses of whole-genome duplications and ancestral karyotypes. *Mol Plant*. 2022;15(12):1841-1851. doi:10.1016/j.molp.2022.10.018.
92. Tang H, Krishnakumar V, Zeng X, et al. JCVI: A versatile toolkit for comparative genomics analysis. *Imeta*. 2024;3(4):e211. doi:10.1002/imt2.211.
93. Li H, Durbin R. Fast and accurate long-read alignment with Burrows-Wheeler transform. *Bioinformatics*. 2010;26(5):589–95. 10.1093/bioinformatics/btp698.
94. Quinlan AR, Hall IM. BEDTools: a flexible suite of utilities for comparing genomic features. *Bioinformatics*. 2010;26(6):841-842. doi:10.1093/bioinformatics/btq033.
95. Ginestet C. ggplot2: Elegant Graphics for Data Analysis. *Journal of the Royal Statistical Society Series A: Statistics in Society*. 2011; doi: 10.1111/j.1467-985X.2010.00676_9.x.
96. Robinson JT, Thorvaldsdottir H, Turner D, Mesirov JP. igv.js: an embeddable JavaScript implementation of the Integrative Genomics Viewer (IGV). *Bioinformatics*. 2023;39(1):btac830. doi:10.1093/bioinformatics/btac830.
97. Camacho C, Coulouris G, Avagyan V, et al. BLAST+: architecture and applications. *BMC Bioinformatics*. 2009;10:421. doi:10.1186/1471-2105-10-421.
98. Zhang Z, Xiao J, Wu J, et al. ParaAT: a parallel tool for constructing multiple protein-coding DNA alignments. *Biochem Biophys Res Commun*. 2012;419(4):779-781. doi:10.1016/j.bbrc.2012.02.101.
99. Wang D, Zhang Y, Zhang Z, Zhu J, Yu J. KaKs_Calculator 2.0: a toolkit incorporating

gamma-series methods and sliding window strategies. *Genomics Proteomics Bioinformatics*. 2010;8(1):77-80. doi:10.1016/S1672-0229(10)60008-3.

100. Nakamura T, Yamada KD, Tomii K, Katoh K. Parallelization of MAFFT for large-scale multiple sequence alignments. *Bioinformatics*. 2018;34(14):2490-2492. doi:10.1093/bioinformatics/bty121.

101. Minh BQ, Schmidt HA, Chernomor O, et al. IQ-TREE 2: New Models and Efficient Methods for Phylogenetic Inference in the Genomic Era. *Mol Biol Evol*. 2020;37(5):1530-1534. doi:10.1093/molbev/msaa015.

102. Chen S, Zhou Y, Chen Y, Gu J. fastp: an ultra-fast all-in-one FASTQ preprocessor. *Bioinformatics*. 2018;34(17):i884-i890. doi:10.1093/bioinformatics/bty560.

103. Dobin A, Davis CA, Schlesinger F, et al. STAR: ultrafast universal RNA-seq aligner. *Bioinformatics*. 2013;29(1):15-21. doi:10.1093/bioinformatics/bts635.

104. Li B, Dewey CN. RSEM: accurate transcript quantification from RNA-Seq data with or without a reference genome. *BMC Bioinformatics.* 2011;12:323. doi:10.1186/1471-2105-12-323.

Tables and Figures

Figure 1. Genomic features of male *H. salicifolia*. (a) Pseudochromosome. (b) Gene density. (c) Repeat sequences density. (d) Gypsy density. (e) Copia density. (f) GC content. (g) Interspecies collinearity. Feature density and GC percentage were calculated with a 5Mb window size. *H. salicifolia* is diploid, so the haploid genome was used as the reference genome.

Figure 2. Comparative genomics analysis. (a) The divergence time tree of 13 species. The number of expanded gene families (red) and the number of contracted gene families (blue) are indicated to the right of each species branch. (b) Synonymous substitution rate per site (Ks) distribution for *H. salicifolia*, *H. gyantsensis*, *H. rhamnoides*, *H. tibetana*, *E. moorcroftii* and *Z. jujuba*. Two recent whole genome duplication(WGD) events occurred in both *Hippophae* and *Elaeagnus*. (c) Macro-synteny plot of *H. salicifolia*, *E. mollis* and *Z. jujuba*. Syntenic comparison between *H. salicifolia* and *Z. jujuba* or between *E. mollis* and *Z. jujuba* revealed a 4:1 ratio that suggests two lineage-specific WGDs in Elaeagnaceae.

Figure 3. Identification of the *H. salicifolia* 2X- and 2Y-SLRs. (a) The next-generation sequencing data of male and female *H. salicifolia* were visualized in the coverage depth of Chr02 of Hap2. The red line represents the depth of female coverage. The blue line represents

the depth of male coverage. Y indicates the position of the Y-SLR. (b) Genes distribution in 2Y-SLR (c) The next-generation sequencing data of male and female *H. salicifolia* were visualized in the coverage depth of Chr02 of Hap1. The red line represents the depth of female coverage. The blue line represents the depth of male coverage. X indicates the position of the X-SLR. (d) Genes distribution in 2X-SLR.

Figure 4. Sex chromosome evolution of *H. salicifolia.* (A) The local syntenic blocks identified between the Hap2 Chr02 and the genomes of *H. rhamnoides* and *E. mollis*. (B) The order and direction of Y-SLR in homologous blocks in the orthologous region of *Hippophae* and *Elaeagnus*. '+' indicates that the internal gene order is consistent, '-' indicates that the internal gene order is opposite, and the absence of a mark suggests that the direction of the internal genes is variable. (C) The distribution of Ks values across different strata, with * indicating the mean value. (D) Phylogenetic structure statistics of single-copy genes within different strata. Structure 1 represents the "ancestral" pattern, Structure 2 represents the "recent" pattern, and Structure 3 represents the "chaotic" pattern.

Figure 5. Within the Y-SLR of *H. salicifolia*, there are male-specific expression genes in both *H. salicifolia* and *H. gyantsensis*. 'F' represents female, and 'M' represents male.

Figure1

Click here to access/download;Figure;Fig1.tif

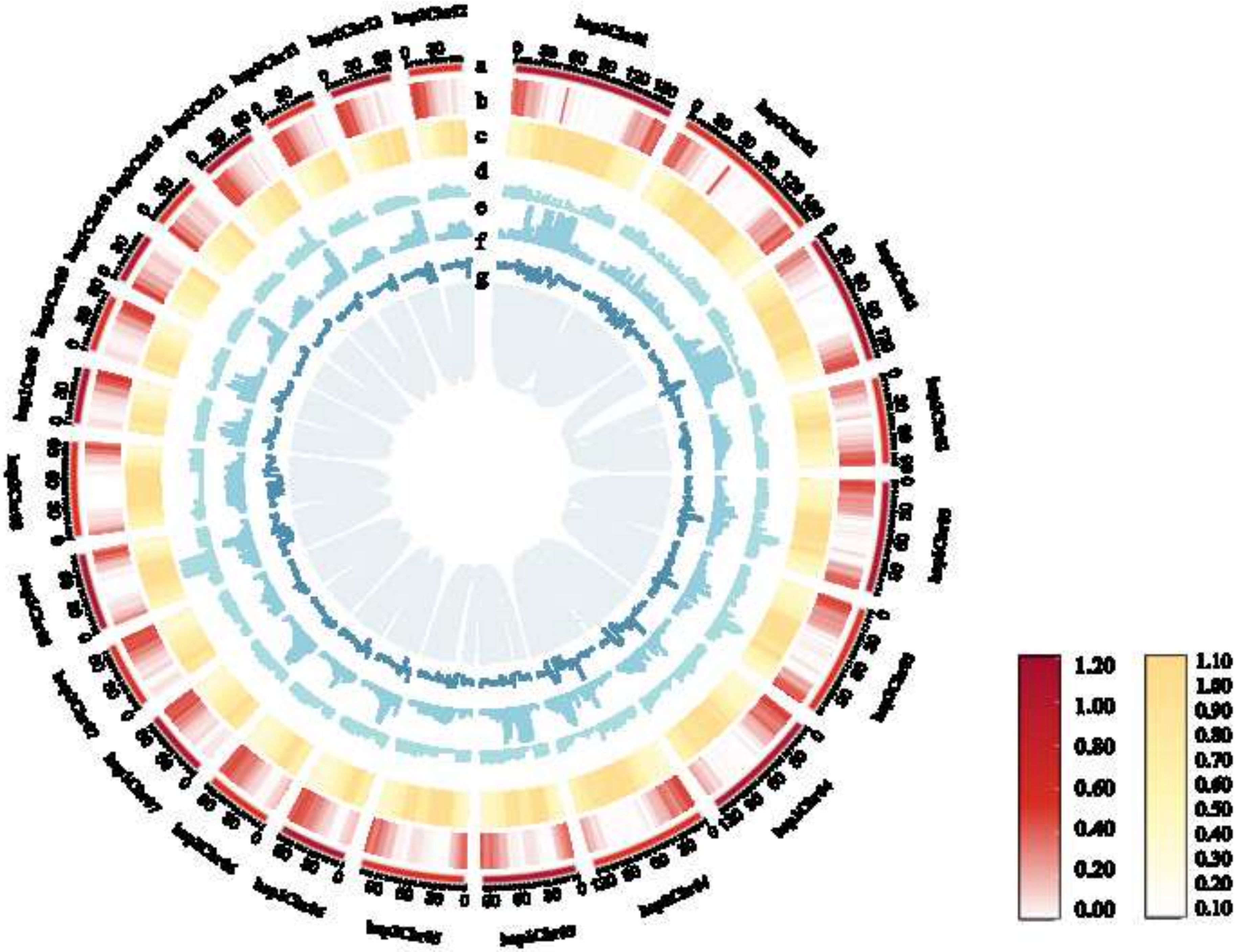

Figure2

Click here to access/download;Figure;Fig2.tif

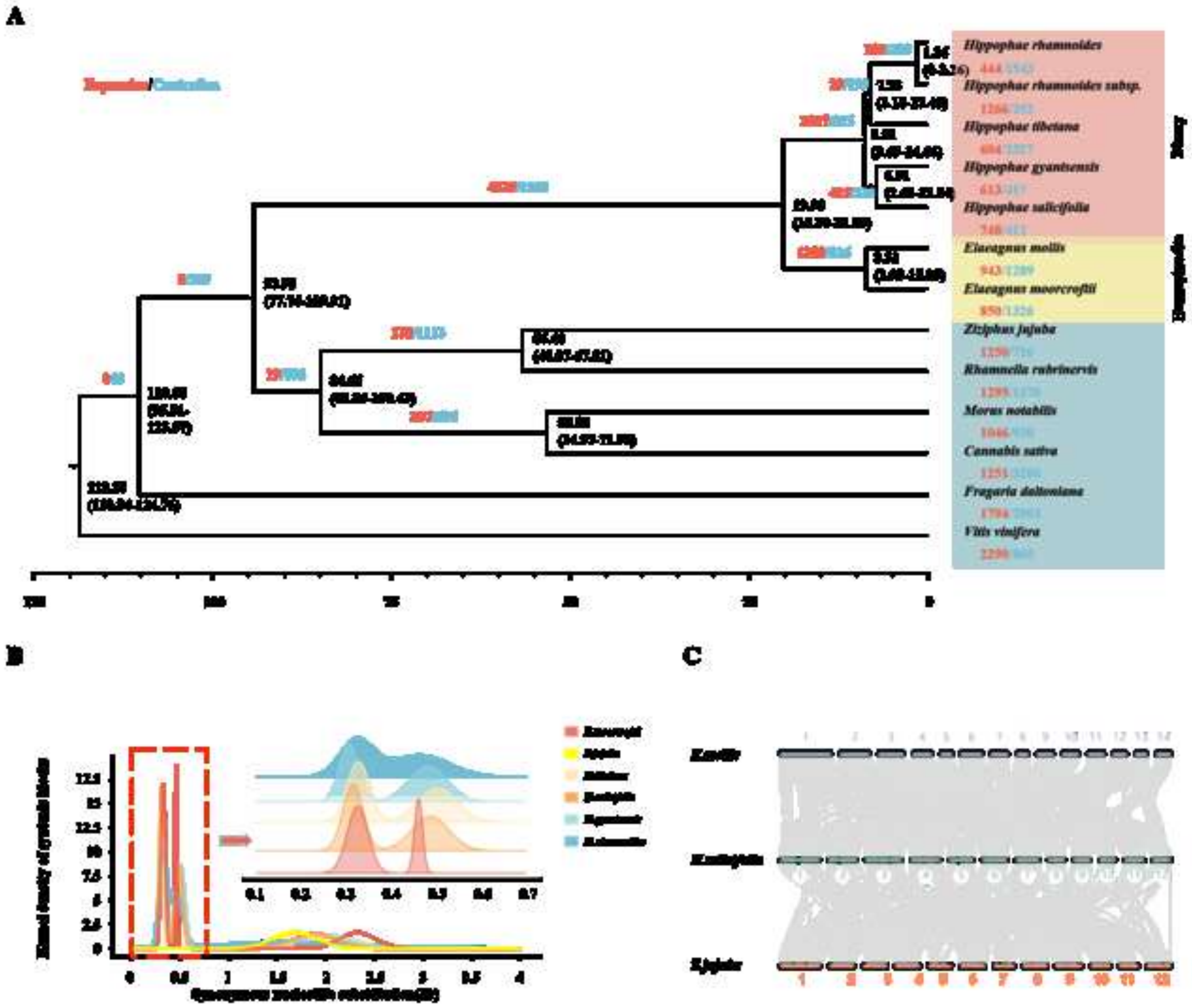

Figure3
Click here to access/download;Figure;Fig3.tif

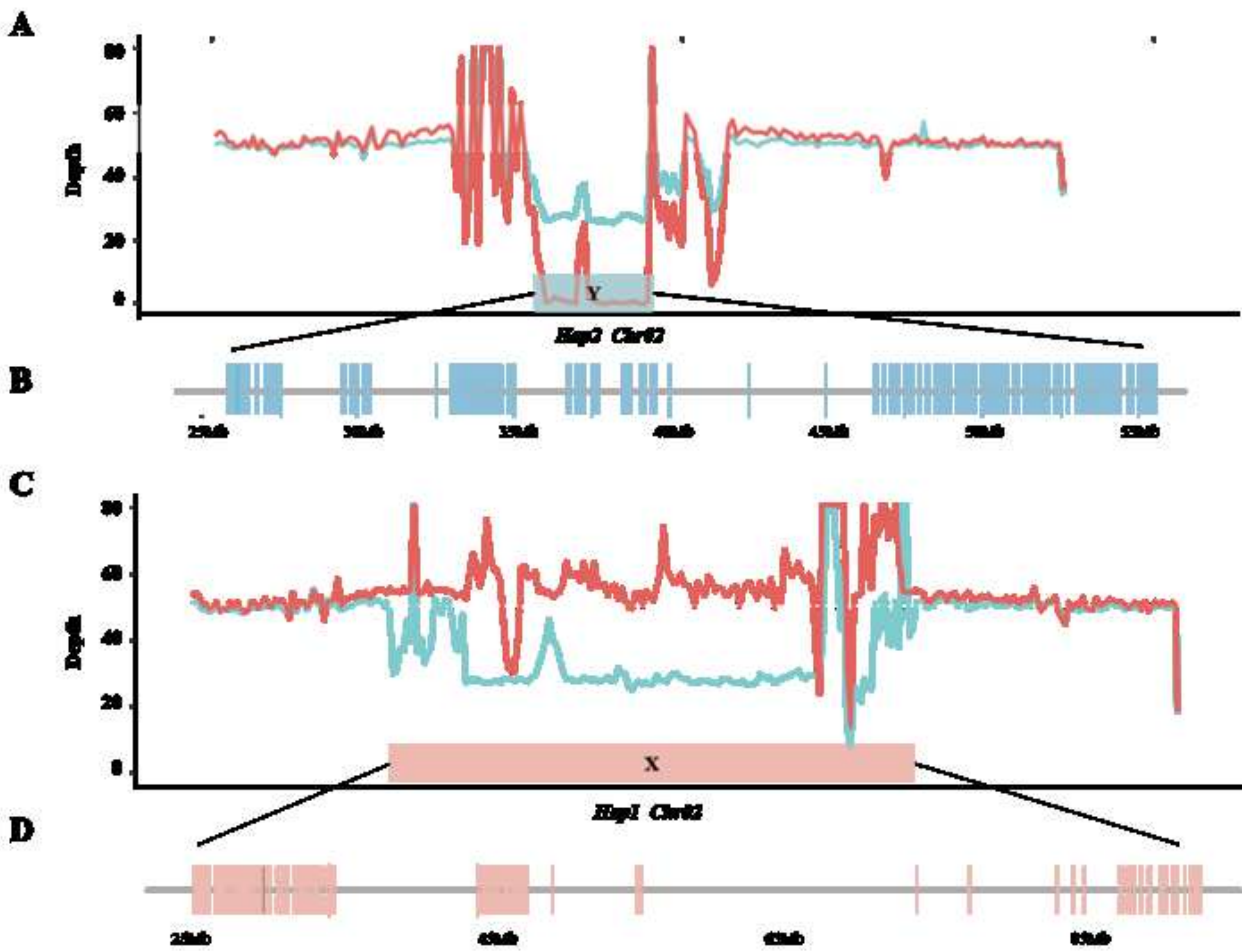

Figure4

Click here to access/download;Figure;Fig4.tif

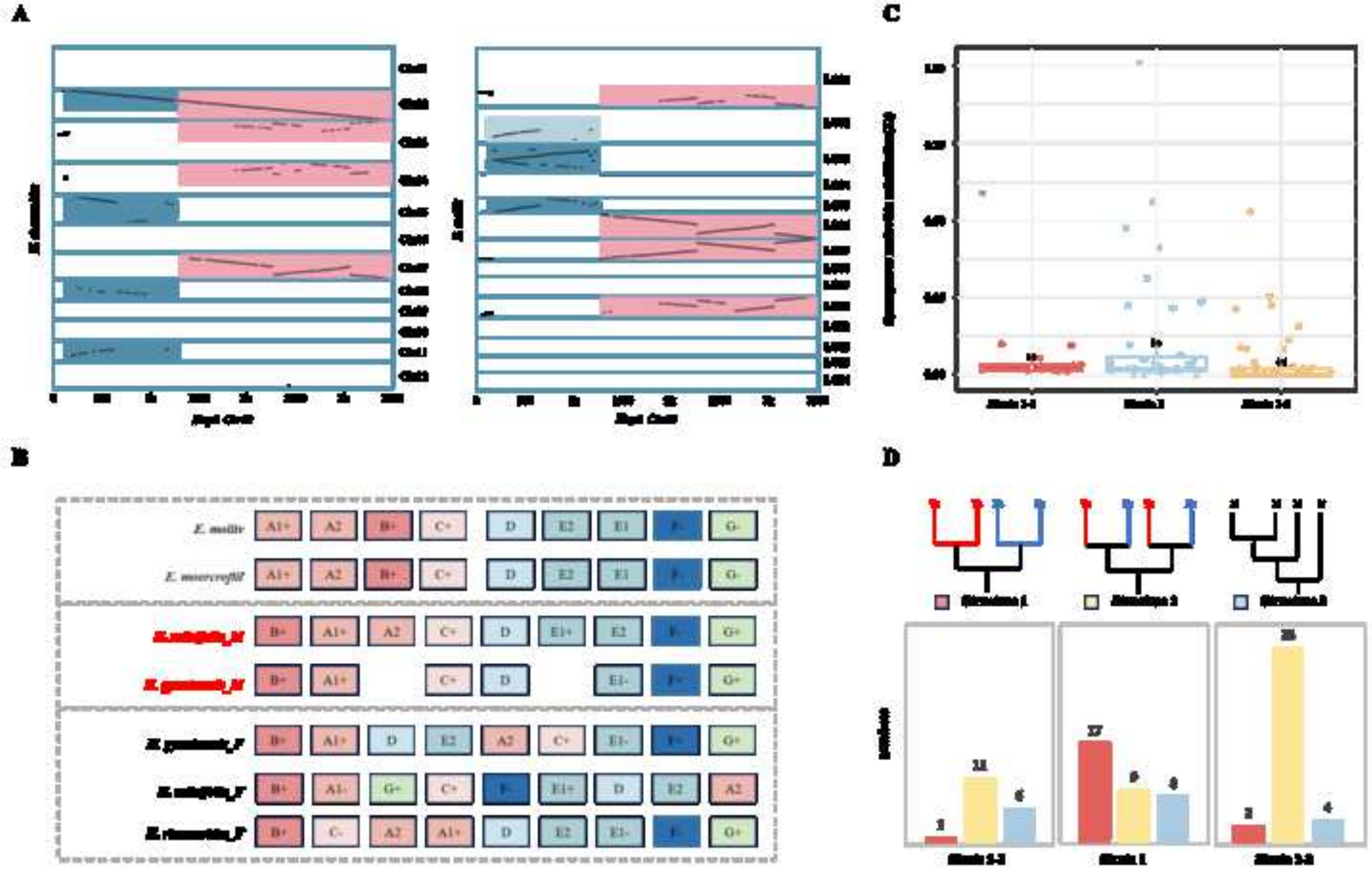

Figure5

Click here to access/download;Figure;Fig5.tif

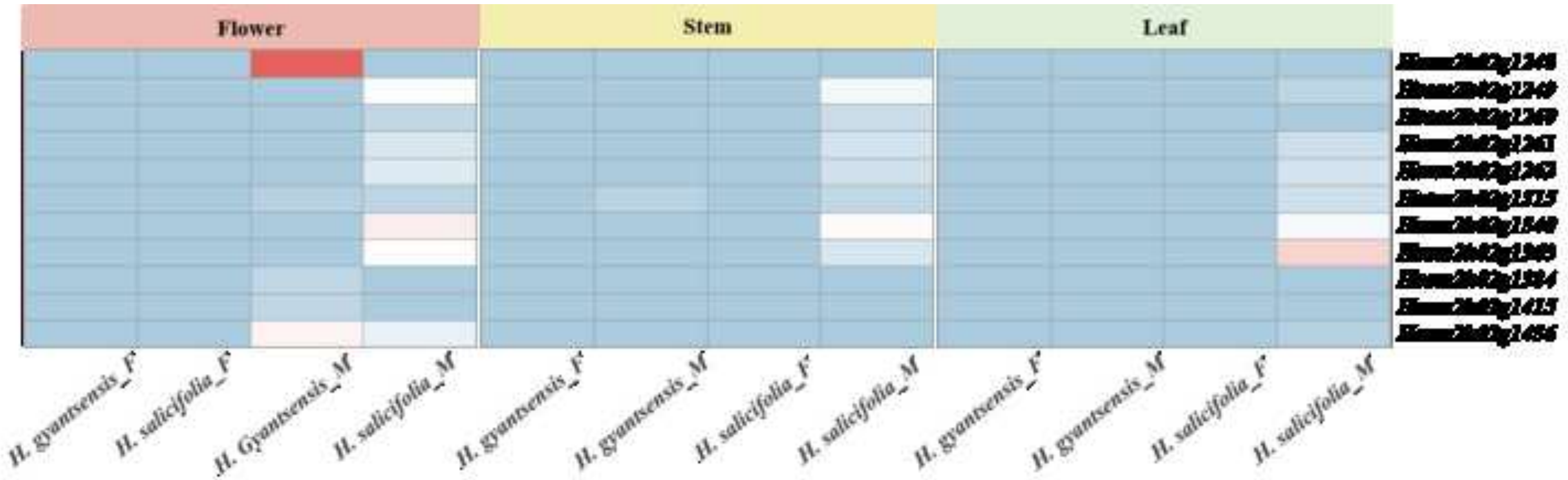

Table

Click here to access/download
Supplementary Material
Table.xlsx

Figure

Click here to access/download
Supplementary Material
Fig.docx
