# Supplementary material for: The genome of Hippophae salicifolia provides new insights into the sexual differentiation of sea buckthorn
Source: Gigascience. 2025 Jul 2;14:giaf046. doi: 10.1093/gigascience/giaf046 (PMC12218201; doi:10.1093/gigascience/giaf046)
Supplement: giaf046_GIGA-D-24-00421_Revision_1 [file giaf046_giga-d-24-00421_revision_1.pdf]

GigaScience

The genome of Hippophae salicifolia provides new insights into the sexual differentiation of seabuckthorn

--Manuscript Draft--

**Manuscript Number:**	GIGA-D-24-00421R1
**Full Title:**	The genome of Hippophae salicifolia provides new insights into the sexual differentiation of seabuckthorn
**Article Type:**	Research
**Funding Information:**	the Second Tibetan Plateau Scientific Expedition and Research (STEP) program (2019QZKK0502) — Dr Yongping Yang<br>Science and Technology Plan Projects of Tibet Autonomous Region (XZ202401ZY0006) — Dr Yongping Yang<br>Science and Technology Program Projects of Shigatse City (RKZ2023ZY-03) — Dr Shihai Yang<br>Yunling Scholar Project to Yang Yongping — Dr Yongping Yang

Funding	Recipient
the Second Tibetan Plateau Scientific Expedition and Research (STEP) program (2019QZKK0502)	Dr Yongping Yang
Science and Technology Plan Projects of Tibet Autonomous Region (XZ202401ZY0006)	Dr Yongping Yang
Science and Technology Program Projects of Shigatse City (RKZ2023ZY-03)	Dr Shihai Yang
Yunling Scholar Project to Yang Yongping	Dr Yongping Yang

Abstract:

Background: Dioecy, a common reproductive strategy in angiosperms, has evolved independently in various plant lineages, and this has resulted in the evolution of diverse sex chromosome systems and sex determination mechanisms. Hippophae is a genus of dioecious plants with an XY sex determination system, but the molecular underpinnings of this process have not yet been clarified. Most previously published sea buckthorn genome data have been derived from females, yet genomic data on males are critically important for clarifying our understanding of sex determination in this genus. Comparative genomic analyses of male and female seabuckthorn plants can shed light on the origins and evolution of sex. These studies can also enhance our understanding of the molecular mechanisms underlying sexual differentiation and provide novel insights and data for future research on sexual reproduction in plants.
Results: We conducted an in-depth analysis of the genomes of two seabuckthorn species, including a male Hippophaegyantsensis, a female Hippophaesalicifolia, and two haplotypes of male H. salicifolia. The genome size of H. gyantsensis was 704.35 Mb, and that of the female H. salicifolia was 788.28 Mb. The sizes of the two haplotype genomes were 1,139.98 Mb and 1,097.34 Mb. The sex-determining regions (SDRs) of the two male sea buckthorn genomes were mapped. The SDR of H. salicifolia was 29.71 Mb and contained 249 genes. The SDR of H. gyantsensis was 12.31 Mb and contained 235 genes. A comparative analysis of the haplotypes of Chr02 of H. salicifolia revealed that the Y chromosome was shorter than the X chromosome. Chromosomal evolution analysis indicated that Hippophae has experienced significant chromosomal rearrangements following two whole-genome duplication events, and the fusion of two chromosomes has potentially led to the early formation of sex chromosomes in seabuckthorn. Multiple structural variations between Y and X sex-linked regions might have facilitated the rapid evolution of sex chromosomes in H. salicifolia. Comparison of the transcriptome data of male and female flower buds from H. gyantsensis and H. salicifolia revealed 11 genes specifically expressed in males. Three of these were identified as candidate genes involved in the sex determination of seabuckthorn. These findings will aid future studies of the sex determination mechanisms in seabuckthorn.
Conclusion: A comparative genomic analysis was performed to identify the sex-determination regions (SDRs) in H. gyantsensis and H. salicifolia. The origins and evolutionary trajectories of sex chromosomes within Hippophae were also determined. Three potential candidate genes associated with seabuckthorn sex determination were identified. Overall, our findings will aid future studies aimed at clarifying the mechanisms of sex determination.

**Corresponding Author:**	mingyue chen<br>XTBG: Xishuangbanna Tropical Botanical Garden<br>yunnan, CHINA
**Corresponding Author Secondary**	

Powered by Editorial Manager® and ProduXion Manager® from Aries Systems Corporation

**Information:**	
**Corresponding Author's Institution:**	XTBG: Xishuangbanna Tropical Botanical Garden
**Corresponding Author's Secondary Institution:**	
**First Author:**	Mingyue chen
**First Author Secondary Information:**	
**Order of Authors:**	Mingyue chen
	Xingyu Yang
	Lan Xun
	Zhenlin Qu
	Shihai Yang
	Yunqiang Yang
	Yongping Yang
**Order of Authors Secondary Information:**	
**Response to Reviewers:**	We would like to express our gratitude to the editor for granting us the opportunity to resubmit our paper. We are also deeply appreciative of the constructive suggestions provided by the reviewers, which have been instrumental in enhancing both the language and the depth of our manuscript. Hereby, we submit a revised version of our manuscript titled “The genome of Hippophae salicifolia provides new insights into the sexual differentiation of sea buckthorn” incorporating the modifications as per the recommendations from the editor and reviewers. All changes have been highlighted in red within the revised manuscript.<br><br>Reviewer #1: I have read the manuscript titled "The genome of Hippophae salicifolia provides new insights into the sexual differentiation of seabuckthorn" from Chen and colleagues. This work assembled and annotated the genomes of two Hippophae species, with an emphasis on mapping sex-determining regions (SDRs) and comparing sex chromosomes. The research uncovered differences in structural variations between sex chromosomes, and identified candidate genes related to SDR. These findings provide important insights for understanding the evolution of sex determination and will be valuable resources in seabuckthorn. The manuscript is well organized and the most important analyses and information were included, but the authors need to address some issues that are described below.<br>1. In Figure S2, some blanks were exhibited in HiC cluster, but these areas were not all blank in different assemblies. For example, the blanks in Chr05 and Chr08 were different among female, hap1 and hap2 genomes, why?<br>Reply:<br>Thank you very much for raising the question regarding the differences in the HiC clustering gaps observed in Figure S2. The disparities in these gaps between Chr05 and Chr08 are likely attributable to genomic structural variations and haplotype diversity. Similar phenomena have been documented in other species [1,2]. Additionally, the differences observed between the female genome and hap1/hap2 may primarily stem from variations in sequencing and assembly strategies. We are grateful to the reviewer for highlighting this important issue and hope that the above explanation addresses your concerns.<br>[1] Cheng, S., Zhang, Q., Geng, X. et al. Haplotype-resolved chromosome-level genome assembly of Ehretia macrophylla. Sci Data 11, 589 (2024). https://doi.org/10.1038/s41597-024-03431-9<br>[2] Oriowo T. O., Chrysostomakis I., Martin S. et al. A chromosome-level, haplotype-resolved genome assembly and annotation for the Eurasian minnow (Leuciscidae: Phoxinus phoxinus) provide evidence of haplotype diversity. Gigascience. Jan 6;14:giae116. (2025). https://doi.org/10.1093/gigascience/giae116.<br><br>2. Page 8, Hap1 and Hap2 of male H. salicifolia genomes were 309.06-351.7 Mb larger

than those of females by about 40%. How to explain these significant differences?

Reply:
We appreciate your inquiry. The disparity in genome size between those assembled using Oxford Nanopore Technologies (ONT) sequencing and those assembled using HiFi sequencing is primarily attributable to the distinct sequencing and assembly strategies employed. For instance, the recently published genomes of Hippophae tibetane exhibit significant size differences [1, 2]. Should further analysis or clarification be required, please do not hesitate to contact us.
[1] Wang R, Wu B, Jian J, et al. How to survive in the world's third poplar: Insights from the genome of the highest altitude woody plant, Hippophae tibetana (Elaeagnaceae). Front Plant Sci. 2022;13:1051587. doi:10.3389/fpls.2022.1051587.
[2] Zhang G, Song Y, Chen N, et al. Chromosome-level genome assembly of Hippophae tibetana provides insights into high-altitude adaptation and flavonoid biosynthesis. BMC Biol. 2024; 22: 82. https://doi.org/10.1186/s12915-024-01875-4

3. Page 9, why is the BUSCO completeness rates scores of genomes (97.4%, 97.6%, 97.6%, 97.7%) less than those of gene set (98.9%, 98.8%, 98.9%, 98.7%)?
Reply:
Thank you for your question. To address this issue, we have reviewed relevant literature and searched for the criteria used in BUSCO scoring. Based on our findings, we believe that the slightly higher BUSCO score for the gene set compared to the genome assembly is attributable to several factors. First, the objects and methods of evaluation differ between the two, making discrepancies quite normal. Additionally, similar observations, where the gene set BUSCO score exceeds that of the genome assembly, have been reported in other genomic studies[1,2].
[1] Yang T, Cai Y, Huang T, Yang D, Yang X, Yin X, Zhang C, Yang Y, Yang Y. A telomere-to-telomere gap-free reference genome assembly of avocado provides useful resources for identifying genes related to fatty acid biosynthesis and disease resistance. Hortic Res. 2024 Apr 22;11(7):uhae119. doi: 10.1093/hr/uhae119.
[2] Wang Y, Zhang RG, Hörandl E, Zhang ZX, Charlesworth D, He L. Evolution of Sex-linked Genes and the Role of Pericentromeric Regions in Sex Chromosomes: Insights from Diploid Willows. Mol Biol Evol. 2024 Nov 1;41(11):msae235. doi: 10.1093/molbev/msae235.

4. Page 10, "Synteny analysis revealed one-to-one homology relationships … a higher frequency of chromosomal rearrangements."
This part of the results were not presented in any graphical or tabular form and need to supplemented.
Reply:
We are grateful to the reviewer for the meticulous review and valuable comments. The additional content has been incorporated into the revised manuscript, specifically on line 1 of page 11 and in Supplementary Figure 6.

5. Page 11, "Applying the same methodology to H. gyantsensis, … 33.86 Mb to 44.78 Mb and contained 235 genes."
The final SLR regions in H. gyantsensis was 12.31 Mb from 14.59 - 15.97 Mb and 33.86 - 44.78 Mb, but the depths in 14.59 - 15.97 Mb and 39.47 - 44.78 Mb do not differ between male and female as those in the H. salicifolia genome. In the presence of species difference, why refine the SLR with collinearity when you have precise results for this analysis?
Reply:
We appreciate the insightful comments from the reviewer. Since the male genome data of Hippophae gyantsensis was not phased, its Chr02 contains sequences from both the X and Y chromosomes. For regions with significant differences between the X and Y chromosomes, we can accurately pinpoint these areas using the coverage depth differences in resequencing data. However, for sequences that are unique to the X and Y chromosomes but show subtle differences, there may be instances of merged assembly. Therefore, we employed the two aforementioned methods to localize the Y-SLR in H. gyantsensis. Nevertheless, we did not account for the potential impact of interspecies differences. To avoid any potential misinterpretation, we have decided not to specifically localize the Y-SLR interval in H.gyantsensis, but rather to use its genomic data as a supplementary tool for subsequent analyses.

6. Page 13, "These observations indicate a lineage-specific chromosomal fusion event in seabuckthorn, and Chr02 was possibly derived from the merging of two ancestral chromosomes."
Even if previous studies suggest that dioecy may be a newer evolutionary state, we can't identify directly from the phylogenetic tree either Hippophae or Elaeagnus was closer to the ancestor. Without a covariance comparison between the common ancestor and these two clades, I think the current results may only indicate a break or fusion occurred in Chr02. Other species that diverged earlier in the Hippophae or Elaeagnus should be added for collinearity comparison and illustration if retaining the existing conclusions.
Reply：
We sincerely thank the reviewer for their valuable suggestion. In response to your advice, we selected species from the Rhamnaceae family, a group closely related to Elaeagnaceae, as outgroups for the collinearity analysis. Specifically, we used Ziziphus jujuba and Rhamnella rubrinervis to investigate whether a fusion event occurred on Chr02. The results of this analysis are presented in Fig. S10, and a detailed description has been added on page 13 of the revised manuscript. This approach has provided further insights into the evolutionary dynamics of Chr02, and we believe it significantly strengthens our findings. Please let us know if additional clarifications or analyses are needed. We greatly appreciate your constructive feedback.

7. Page 13, "The collinear regions were categorized into nine distinct blocks through comparison of chromosomal segment synteny (Supplementary Fig. S10 and Table S9)."
There are 10 blocks from A to H in the Supplementary Table S9, why was H not used together in the analysis?
Reply:
Thank you very much for pointing out this issue; it was an oversight on my part. The SLR only includes 9 blocks because Block H is not within the SLR range we previously determined. I have removed the content of Block H from Supplementary Table 9 and have provided an explanation for Block H in the Fig S11.

8. Page 13, "Block D had the highest Ks value (Ks=0.1266), … and block G (Ks=0.0037)."
It seems that the mean Ks value of the gene pairs was used as the result, but it was not mentioned here or in the method. It should be clarified.
Reply：
We sincerely thank the reviewer for their valuable comment and apologize for the oversight. In response to your suggestion, we have added a detailed description of the method used to calculate the average KS values for different genomic blocks in the Methods section. This addition can be found on line 18-20 of page 28 of the revised manuscript.

9. Page 14, "Stratum 1 (including blocks C and D) was located …. Stratum 2-1 (including blocks A and B) was located …. Stratum 2-2 (including blocks E, F, and G) was located … with an average Ks value of 0.0418 (Fig 4c)."
From the Supplementary Table S10, we can see that the mean Ks value of block C was significant different from that of block D, and they were also relatively far apart from each other in chromosome, but why was it divided into the same group?
Reply：
Thank you for your feedback. I apologize for the lack of clarity in my original description regarding the stratification of genomic layers. I have revised the content, which can be found in the red text on pages 15 and 16. Our stratification is based on the Ks values and the topological structure of the homologous gene trees. In the revised version, we have refined our description to better articulate this approach. Furthermore, I provide additional rationale for this stratification below:
Combining the results of Supplementary Figure 11 and the previous collinearity analysis, we speculate that regions C and D were likely distributed on two separate chromosomes in an early ancestral form, and a lineage-specific chromosomal fusion event brought them into proximity. Numerous studies have shown that during chromosomal evolution, sex-determining regions tend to accumulate a large number of

repetitive sequences. The interval between regions C and D has similarly accumulated a substantial amount of repetitive sequences, which likely accounts for the greater physical distance between C and D due to transposable element (TE) insertions.
Secondly, more than half of the genes in these two regions exhibit the topological structure characteristic of ancient strata (as shown in Structure 1 in Figure 4d), suggesting that both regions are inclined to belong to the older strata. Additionally, region D shows structural variations between the X and Y chromosomes. Structural variations can exacerbate the differentiation of homologous genes on the X and Y chromosomes, leading to higher Ks values. Therefore, the higher Ks value of region D compared to region C can be attributed to these structural variations.
Based on these considerations, we are inclined to classify regions C and D as Stratum 1.
If you require further clarification or additional details, please do not hesitate to contact us.

10. Page 14, "Distinct phylogenetic patterns were observed … tended to cluster together." This point needs to be supported by references.
Reply:
We thank the reviewer for pointing out the need for supporting references. We have revised the sentence to include relevant citations that support the observed phylogenetic patterns and clustering tendencies.

11. Page 14, "In Stratum 1, an ancient origin was observed for 16 out of 34 single-copy genes;"
There were 17 Structure1 trees in Stratum 1 which was showed in Fig 4d, please check this result.
Reply:
We sincerely appreciate the reviewer's careful attention to this detail. Upon re-examining the phylogenetic tree results, we have confirmed that the correct number is indeed 17. We have carefully revised the text to reflect this correction and ensure accuracy.

12. Page 15, "We utilized transcriptome data, compared these data with the Y-SLR of H. salicifolia, and calculated the transcripts per million (TPM) values of the transcripts (Supplementary Table S15)."
"Supplementary Table S15" should be amended to "Supplementary Table S12". Additionally, there was no stem sampling and biological replicates of three tissues mentioned in METHOD.
Reply:
We sincerely apologize for the oversight and thank the reviewer for pointing out this error. Due to the addition of a new figure, we have changed "Supplementary Table S15" to "Supplementary Table S13" in the revised manuscript. We have also added the description of stem sampling and the use of biological replicates for three tissues in the METHOD section on pages 24 and 32.

13. Page 19, "however, the results of our study indicate that X-linked structural variations might be selected and fixed to suppress recombination between XY chromosomes."
It seems that I don't find relevant results, where was this conclusion being drawn from?
Reply:
Based on the comprehensive analysis of Fig. 4b, we have formulated a scientifically grounded hypothesis regarding the observed genomic variations. Our investigation reveals that the X-SLR region exhibits a significantly higher frequency of structural variations compared to its Y-SLR counterpart. These structural alterations demonstrate a potential correlation with our proposed stratigraphic formation patterns.
The integration of recent groundbreaking research on Silene latifolia provides compelling evidence that structural variations within the X-SLR region may facilitate recombination suppression between sex chromosomes. This empirical evidence strongly supports our hypothesis that X-linked structural variations might undergo selective pressure and subsequent fixation to establish recombination suppression mechanisms between XY chromosomes. To more clearly articulate our rationale, we

Powered by Editorial Manager® and ProduXion Manager® from Aries Systems Corporation

have added content to lines 4–8 on page 14 and lines 14–20 on page 16.

Reviewer #2: This manuscript does an excellent job describing the sex-linked region of seabuckthorn. Overall, the introduction provides good context for why this analysis is needed, and the results are logically ordered with most of their hypotheses supported directly with their data. The identification of candidate genes with orthologs in Arabidopsis is a particularly exciting result. Even though no molecular characterization is performed, that is certainly beyond the scope of this manuscript. It is a complete genome assembly and analysis manuscript. I do have a number of minor comments where I believe further clarification is required, but overall kudos to the authors.

1. - "increase number of acquired genes in X SLR"
- page 12, how can you separate that from the hypothesis of an increased loss in Y instead of insertions in X?
- a better explanation of what "ancestral" and "acquired" genes are is required
Reply:
Thank you very much for your suggestions. We apologize for the lack of clarity in the description of the methodology in the original manuscript. In the revised version, we have provided a more detailed explanation in the Methods section on page 30. Additionally, I would like to elaborate further here:
Given that Hippophae and Elaeagnus both belong to the Elaeagnaceae family and diverged after sharing two whole-genome duplication (WGD) events, we defined genes within the SLR that have homologs in the corresponding homologous segments of both Hippophae and Elaeagnus as “ancestral” genes. For instance, based on the results shown in Supplementary Figure 8, if a gene within the SLR has a homolog located in the designated blocks, it is highly likely to be an “ancestral” gene, as these genes have undergone the WGD events prior to the divergence of the Hippophae and Elaeagnus genera.
In contrast, genes within the SLR that have homologs in other chromosomal regions are classified as “acquired” genes. These genes are likely derived from duplications originating from other chromosomes. While there are certainly factors that could influence the results, the significant difference in the number of “acquired” genes between the X- and Y-SLRs supports the notion that gene acquisition occurred in the X-SLR rather than gene degeneration in the Y-SLR.

2. - what was the sampling of Male / Female? Could differences be variation within the species instead of just between male / female?
Reply:
We thank the reviewer for raising this important question. To elucidate this issue, we conducted second-generation sequencing on pooled samples of fourteen female and fourteen male Hippophae salicifolia. The sequencing data were aligned to chromosome 2, and the results revealed that the differences observed between the X- and Y-SLRs are indeed due to divergence between sex chromosomes rather than species variation, with no changes occurring in the respective regions. The data have been updated accordingly. For details, please refer to Figure 3, lines 4–7 on page 11, and the Materials and Methods section.

3. - Throughout, some genus and species names are not italicized
Reply :
Thank you for pointing out this formatting issue. We have made the necessary revisions and have double-checked to ensure consistency in formatting throughout the manuscript.

4. last paragraph of the introduction:
"The genetic basis of sexual differentiation in seabuckthorn has been a major focus of studies of plant reproductive biology and sexual evolution"
- Please support this statement with citations
Reply:
We apologize for the oversight. Upon reviewing the text, we identified an inappropriate description and have since revised it accordingly.

5. You should replace all references of "Gypsy" elements to "Ty3"
- https://doi.org/10.31219/osf.io/fma57
Reply:
Thank you very much for your suggestions. I have revised the relevant content accordingly.

6. Figure 4A:
- The units for the x-axis need labeled at least in the figure legend
Reply:
Thank you very much for pointing out this oversight. I sincerely apologize for this omission and have now included the x-axis unit information in the revised manuscript.

7. Figure 5:
- A legend for the heatmap is required, it is ambiguous which colors are high or low expression
Reply:
Thank you for your valuable suggestions. I fully concur with your perspective that the absence of a heatmap legend can indeed impede the interpretation of the data. Following your advice, I have supplemented the revised manuscript with a complete heatmap legend, clearly indicating the correspondence between colors and gene expression levels (red representing high expression and blue representing low expression).

8. Page 15:
". Sex-determining genes often share phylogenetic similarities with ancient strata genes, and homologous genes from the same gametophyte tended to cluster together"
- this statement needs a citation or further justification as to why certain gene tree topologies are expected
Reply:
Thank you very much for raising the issue. I have now cited the relevant literature accordingly.

9. Page 16:
"Additionally, the pseudogenization of Hsam2h02g1248 was observed in the female counterparts of both H. gyantsensis and H. salicifolia, which resulted in the loss of its original function"
- Amino acid alignments are shown in Figure S15, but an additional check of genome sequences is required to ensure true psuedogenization instead of a misannotation of these loci in females
Reply:
We sincerely appreciate your valuable suggestions. Upon further analysis of the genomic sequence, we identified a large fragment insertion that resulted in the disruption of the homologous gene of Hsam2h02g1248 within the X-SLR region. The specific modifications and detailed findings can be found in revised manuscript version 19, as well as in Supplementary Tables 17 and 18.

10. Discussion:
- mentioning the WGD history of these lineages should be mentioned in the introduction. It makes figure 4A make a lot more sense so mentioning it when describing figure 4A may also contextualize the multiple observed syntenic hits
- this makes Figure 4A even more convincing, there are 4 copies of L and R regions, and only a single pair exist on the same chromosome!
Reply :
We extend our gratitude for your insightful suggestions. In response, we have incorporated the recommended content into both the introduction and the description of Figure 4A. The specific revisions can be found in the red-font section on page 7 and within lines 7–15 on page 14 of the revised manuscript.

11. - "The SLRs of papaya and kiwifruit have evolved from the pericentromeric regions [38-40]."
- this sentence seems out of place, maybe:
'sex chromosomes are known to have independently evolved through similar

	mechanisms in other lineages such as...'<br>Reply:<br>Thank you for your valuable suggestions. We have revised the inappropriate descriptions accordingly, and the changes are highlighted in red on page 20 of the revised manuscript.<br><br>12. Figure S2:<br>- The hi-c maps provided are not high enough resolution to evaluate the accuracy of the SLR. It would be helpful to provide an enlarged contact matrix just for chromosome 2 to increase confidence in the order and orientation of the SLR. Alternatively, please provide a statement in the part discussing Figure 4B that the observed lack of synteny may be observed due to misassembly in either the SLR in this manuscript, or in the genomes compared in this manuscript.<br>- HiFi genome assemblies are incredibly high quality and I do not doubt the author's expertise in carefully evaluating the accuracy of their genome assembly. I'm just a stern believer that genome assemblies are hypotheses and not ground truth; a point readers, especially non-specialists, often miss.<br>Reply:<br>Thank you very much for your suggestion. The Hi-C map of chromosome 2 has been added to Supplementary Figure 7. If you require further explanation or additional details, please do not hesitate to contact us, and we will provide more information.<br><br>13. Figure S12A:<br>- the number of tips on the cladogram and labels do not match.<br>- additionally, I'm not sure how this supports the author's hypotheses as they do not explain sufficiently what an expected topology is, or why it is expected. Bootstrap values and details on phylogenetic reconstruction are not easy to find.<br>- It also needs mentioned why a gene tree might deviate from expectation. When compared a set of orthologues, minute differences can change gene tree topology relative to the species tree. Support values for each node should be provided as well.<br>- I really enjoy most of the analyses set forth by the authors, this is not one of them<br>- Maybe I just didn't take enough time to try and understand it<br>Reply:<br>We apologize for the oversight in omitting a label during the figure adjustments. This issue has now been corrected. Additionally, we have provided further clarification regarding the expected topological structure in the revised manuscript, which can be found on lines 10–15 of page 17.<br>We also regret not paying sufficient attention to the support values in the original version. Bootstrap values have now been included in the updated phylogenetic trees, which can be found in Supplementary Figures 13–15.
**Additional Information:**	
**Question**	**Response**
Are you submitting this manuscript to a special series or article collection?	No
**Experimental design and statistics**<br><br>Full details of the experimental design and statistical methods used should be given in the Methods section, as detailed in our Minimum Standards Reporting Checklist. Information essential to interpreting the data presented should be made available in the figure legends.	Yes

Have you included all the information requested in your manuscript?	
**Resources**<br><br>A description of all resources used, including antibodies, cell lines, animals and software tools, with enough information to allow them to be uniquely identified, should be included in the Methods section. Authors are strongly encouraged to cite Research Resource Identifiers (RRIDs) for antibodies, model organisms and tools, where possible.<br><br>Have you included the information requested as detailed in our Minimum Standards Reporting Checklist?	Yes
**Availability of data and materials**<br><br>All datasets and code on which the conclusions of the paper rely must be either included in your submission or deposited in publicly available repositories (where available and ethically appropriate), referencing such data using a unique identifier in the references and in the "Availability of Data and Materials" section of your manuscript.<br><br>Have you have met the above requirement as detailed in our Minimum Standards Reporting Checklist?	Yes
GigaScience has policies and guidelines in place for the use of generative AI-writing tools such as ChatGPT. If you have used such writing tools to assist with writing the manuscript this must be declared and cited in the text. Authors should not list AI-writing tools and other AI-assisted technologies as an author or co-author and should acknowledge that they are fully responsible for text generated or refined by AI-writing tools.<br><br><br>A summary of use (particularly in the introduction or among methods) needs to be included at the end of the paper, and	No

Powered by Editorial Manager® and ProduXion Manager® from Aries Systems Corporation

the outputs should also be included as a supplementary file hosted in GigaDB or other open repositories. Please read our guidelines for more information. <br><br><br>By submitting to GigaScience, you are aware of the journal's AI-writing tools policy, and if you have declared use of such tools below, you have acknowledged this where appropriate in your manuscript and have made a summary of use and outputs available.<br><br><br><br>**AI-assisted writing tools have been used in the preparation of this manuscript?<br>** 	

Manuscript Click here to access/download;Manuscript;Manuscript_Mar 24.docx

The genome of *Hippophae salicifolia* provides new insights into the sexual differentiation of sea buckthorn

Mingyue Chen [a,b,c], Xingyu Yang [a,b], Lan Xun [a,f], Zhenlin Qu [d], Shihai Yang[e,*],Yunqiang Yang[a,b*], Yongping Yang[a,b*]

[a] Xishuangbanna Tropical Botanical Garden, Chinese Academy of Sciences, Mengla, Yunnan 666303, China.

[b]Yunnan International Joint Laboratory for the Conservation and Utilization of Tropical Timber Tree Species, Xishuangbanna Tropical Botanical Garden, Chinese Academy of Sciences, Mengla, Yunnan 666303, China.

[c] School of Ecology and Environment, Yunnan University, Kunming 650091, China

[d] Beijing Jiu Tian International Education, Beijing 100080, China.

[e] Xizang Ecological Harmony Seed Industry Co, Ltd, Shigatse, Xizang 857000, China.

[f] College of Agriculture and Biotechnology, Yunnan agricultural university, Kunming 650500, China.

* corresponding author(s): Yunqiang Yang (yangyunqiang@xtbg.ac.cn) or Yongping Yang (yangyp@xtbg.ac.cn)

Mingyue Chen [0009-0006-0907-3830]; Xingyu Yang [0009-0005-2719-0646]; Yunqiang Yang [0000-0002-8109-8315]; Yongping Yang [0009-0001-2101-9320]

Abstract

Background: Dioecy, a common reproductive strategy in angiosperms, has evolved independently in various plant lineages, and this has resulted in the evolution of diverse sex chromosome systems and sex determination mechanisms. *Hippophae* is a genus of dioecious plants with an XY sex determination system, but the molecular underpinnings of this process have not yet been clarified. Most previously published sea buckthorn genome data have been derived from females, yet genomic data on males are critically important for clarifying our understanding of sex determination in this genus. Comparative genomic analyses of male and female sea buckthorn plants can shed light on the origins and evolution of sex. These studies can also enhance our understanding of the molecular mechanisms underlying sexual differentiation and provide novel insights and data for future research on sexual reproduction in plants.

Results: We conducted an in-depth analysis of the genomes of two sea buckthorn species, including a male *Hippophae gyantsensis*, a female *Hippophae salicifolia*, and two haplotypes of male *H. salicifolia*. The genome size of *H. gyantsensis* was 704.35 Mb, and that of the female *H. salicifolia* was 788.28 Mb. The sizes of the two haplotype genomes were 1,139.98 Mb and 1,097.34 Mb. The sex-determining regions (SDR) of *H. salicifolia* was 29.71 Mb and contained 249 genes. A comparative analysis of the haplotypes of Chr02 of *H. salicifolia* revealed that the Y chromosome was shorter than the X chromosome. Chromosomal evolution analysis indicated

that *Hippophae* has experienced significant chromosomal rearrangements following two whole-genome duplication events, and the fusion of two chromosomes has potentially led to the early formation of sex chromosomes in sea buckthorn. Multiple structural variations between Y and X sex-linked regions might have facilitated the rapid evolution of sex chromosomes in *H. salicifolia*. Comparison of the transcriptome data of male and female flower buds from *H. gyantsensis* and *H. salicifolia* revealed 11 genes specifically expressed in males. Three of these were identified as candidate genes involved in the sex determination of sea buckthorn. These findings will aid future studies of the sex determination mechanisms in sea buckthorn.

Conclusion: A comparative genomic analysis was performed to identify the SDR in *H. salicifolia*. The origins and evolutionary trajectories of sex chromosomes within *Hippophae* were also determined. Three potential candidate genes associated with sea buckthorn sex determination were identified. Overall, our findings will aid future studies aimed at clarifying the mechanisms of sex determination.

Keywords: *Hippophae*, chromosomal assembly, sex chromosomes, sex determination

Introduction

Clarifying the genetic mechanisms underlying sexual differentiation, as well as the origins and evolution of sex chromosomes, is a major goal of evolutionary biology. Conventional theories suggest that sex chromosomes evolve from autosomes through a series of genetic and evolutionary changes, which ultimately become determinants of sex. This evolutionary trajectory is often initiated with two closely linked mutations on an autosome: a dominant mutation that inhibits female development and a recessive mutation inducing male sterility [1,2].

Due to the genetic conflict associated with sex differences in reproductive strategies, sexually antagonistic genes accumulate around these mutant genes, which suppresses recombination [3]. As time progresses, the inhibition of recombination can extend along the chromosome, leading to the establishment of sex-linked regions, and eventually may lead to the emergence of heteromorphic sex chromosomes [4,5]. However, previous studies have shown that the evolution of sex chromosomes does not always result in heteromorphic pairs. For example, the sex chromosomes in scallops have remained highly undifferentiated for over 350 million years. In these species, homomorphic sex chromosomes are notably enriched with numerous bidirectionally reversible sex-biased genes, which might be critically important for maintaining their undifferentiated state [6]. These findings indicate that sex chromosome evolution can follow various patterns. Many aspects of sex chromosomes have not yet been clarified, including the origin of sex and recombination suppression [7]. In contrast to the pronounced differentiation in human and animal sex chromosomes, plant sex chromosomes have arisen and evolved independently across diverse dioecious plant lineages, and they exhibit high diversity and complexity in their evolutionary pathways [8]. Investigation of the sex determination mechanisms in these plants can provide novel insights and data crucial for elucidating the genetic basis of sexual differentiation.

Dioecious plants account for 5% to 6% of angiosperms; sexual differentiation in dioecious plants provides them with unique reproductive advantages [9]. Dioecious plants can optimize the allocation of reproductive resources and enhance breeding efficiency through sexual differentiation [10]. Furthermore, sexual differentiation can affect their ecological niche [11,12]. Some dioecious plants may reduce self-fertilization and increase gene flow through sexual

differentiation, which enhances the genetic diversity of the population. The differentiation of sex chromosomes plays a key role in the molecular mechanism underlying sexual differentiation in dioecious plants. Sex chromosomes, such as the XY and ZW systems, are typically derived from autosomes and evolve into sex-determining chromosomes via a series of genetic and evolutionary processes [2]. The expression patterns of genes on the sex chromosomes, the formation of recombination suppression regions, and the morphological and functional differentiation of sex chromosomes are all key factors affecting these processes [13,14]. Sex-specific gene expression patterns play a key role in sex determination. In dioecious plants, certain genes may be expressed in only one sex, and these differences in gene expression directly affect sex-specific traits [15]. The formation of regions with suppressed recombination is another critical factor affecting sex chromosome differentiation. The recombination frequency in certain regions significantly decreases during sex chromosome evolution, and this phenomenon is known as recombination suppression [14]. Recombination suppression aids the accumulation of sex-specific genes and the stabilization of sex-determining mechanisms. In these regions, gene flow is restricted, which promotes the differentiation of sex-specific genes and the morphological differentiation of sex chromosomes [16].

The identification and functional analysis of sex-determining genes are critically important in studies of sexual differentiation in dioecious plants [8]. Dioecious plants have evolved independently in different lineages and possess distinct sex-determining genes. However, these genes are products of convergent evolution and have similar characteristics that can facilitate their identification. However, the identification of sex-determining genes is extremely challenging because of various factors, such as heterochromatinization and structural variation;

the sex-determining genes of only a few species have been successfully characterized to date. Diospyros lotus is the first plant from which sex-determining genes were successfully isolated and identified [17]. Illumina sequencing data from the F1 generation of both female and male plants have been used for the assembly of the male-specific region (MSY) of the Y chromosome. They were then integrated into bud transcriptome data, and 22 differentially expressed genes were identified within the MSY. Phylogenetic analysis revealed that *OGI* is the only gene that predates the speciation event within Diospyros. Evolutionary analyses indicated that *OGI* is a best candidate for a sex-determining gene, and this hypothesis was confirmed in subsequent experiments. Similarly, the MSY region was assembled in kiwifruit using Illumina sequencing data from the F1 generation, and differential expression analysis was performed using transcriptome data from flower buds at various developmental stages. This approach led to the identification of two male-specific genes, *SyGI* and *FrBy*, which are conserved across all kiwifruit and lack homologous counterparts in females. Transgenic studies have shown that *SyGI* is a dominant inhibitor of carpel development, and *FrBy* knockout results in pollen inactivation and self-incompatibility [18,19]. Despite advances in sequencing technology, which has enabled the identification of some plant sex-determining genes, only a few such genes have been isolated and characterized.

Sea buckthorn (*Hippophae*), which belongs to the family Elaeagnaceae, is a shrub or small tree with distinct dioecious characteristics and simple and delicate flowers. The berries of sea buckthorn are renowned for their medicinal properties and nutritional richness; they thus play a key role in the pharmaceutical, cosmetic, and food industries [20,21]. This species has been used for ecological restoration because of its high stress tolerance and vigorous growth; it also

can reproduce prolifically and shows significant nitrogen-fixation activity, which makes it ideal for wind protection, sand stabilization, and reforestation in arid regions [22]. Sea buckthorn has an XY sex chromosome system and shows clear sexual dimorphism; males typically bear cone-like inflorescences that bloom before the females, whereas females have raceme inflorescences that are comparatively smaller [23]. In contrast, the *Elaeagnus* genus, which belongs to the same family, has undergone two whole-genome duplication events but remains monoecious. However, sea buckthorn transitioned from monoecy to dioecy after these events [24,25].The genomic sequences of *Hippophae tibetana* [26,27], *Hippophae rhamnoides* subsp. sinensis [28], *Hippophae rhamnoides* subsp. mongolica [24], *H. gyantsensis* [29] have all been published. The sex chromosome of *H. tibetana* was identified as the second chromosome through simplified genome-wide association studies [26]; however, detailed research on its sex chromosome has not yet been conducted. Moreover, no studies have compared the genomic differences between sea buckthorn and *Elaeagnus*. Therefore, studies of the sex chromosomes of male sea buckthorn at the genomic level can provide important insights into sexual differentiation in dioecious plants and genetic resources that could aid the breeding of sea buckthorn. These findings will aid future molecular studies of the sex determination mechanisms of sea buckthorn, as well as ongoing efforts to improve varieties and their applications in medicine, ecology, and other fields.

Here, we assembled the genomes of a male *H. gyantsensis* (NCBI:txid193515), a female *H. salicifolia* (NCBI:txid48234), and two male *H. salicifolia* with haplotype resolution. Specifically, we conducted a comparative genomic analysis to identify the SDRs in both species and examined their genomic distribution and characteristics, along with the origin and evolution

of the sex chromosomes of sea buckthorn. Analysis of transcriptomic data from male and female flowers revealed three candidate genes. Our findings provide new insights into sexual differentiation in sea buckthorn and will aid ongoing efforts to enhance sex-related traits and molecular breeding in the future.

Results

Genome estimation, sequencing, and assembly

To evaluate the genome size and heterozygosity of the three sea buckthorn species, we conducted genomic studies using clean reads obtained from the Illumina next-generation sequencing (NGS) platform: male *H. salicifolia* (57.60Gb), male *H. gyantsensis* (51.95Gb), and female *H. salicifolia* (62.31Gb) (Supplementary Table S1). K-mer (k=21) analysis (Supplementary Fig. S1) indicated that the genome size of male *H. gyantsensis* was approximately 1,031.76 Mb, with a heterozygosity rate of approximately 1.39%; the genome size of male *H. salicifolia* was approximately 1,018.51 Mb, with a heterozygosity rate of approximately 0.744%; and the genome size of female *H. salicifolia* was approximately 1,134.93 Mb, with a heterozygosity rate of approximately 0.728%.

We obtained clean reads from Oxford Nanopore (ONT) sequencing for female *H. salicifolia* (112.31 Gb) and male *H. gyantsensis* (99.03 Gb) to assemble the genomes of the three sea buckthorn species; HiFi reads were obtained for male *H. salicifolia* (142.55 Gb) (Supplementary Table S1). Hi-C sequencing generated 79.47 Gb of clean reads for male *H. salicifolia*, 82.52 Gb for female *H. salicifolia*, and 136.13 Gb for male *H. gyantsensis*, which

were used for chromosome anchoring (Supplementary Table S2). The contigs of the three genomes were anchored to 12 pseudochromosomes (Supplementary Fig. S2). The genome size of male *H. gyantsensis* was 704.35 Mb; the contig N50 was 18.11 Mb(Supplementary Fig. S3); the genome size of female *H. salicifolia* was 788.28 Mb, with a contig N50 of 30.83 Mb (Supplementary Fig. S4). Male *H. salicifolia* was assembled into two haplotypes, Hap1 and Hap2. The genome size of Hap1 was 1,139.98 Mb, with a contig N50 of 53.61 Mb; the genome size of Hap2 was 1,097.34 Mb, with a contig N50 of 64.70 Mb (Supplementary Table S3) (Fig. 1). The genomic BUSCO completeness rates scores were 97.4% for male *H. gyantsensis*, 97.6% for female *H. salicifolia*, 97.6% for male *H. salicifolia* Hap1, and 97.7% for male *H. salicifolia* Hap2 (Supplementary Table S4).

Genome annotation

The repetitive sequence content of the male *H. gyantsensis* genome was 56.42%, and long terminal repeat sequences (LTRs) accounted for 35.06% of the repetitive sequences. The repetitive sequence content of the female *H. salicifolia* genome was 60.41%, and LTRs comprised 37.68% of the repetitive sequences. For Hap1, the repetitive sequence content was 70.88%, of which LTRs comprised 43.41% of the repetitive sequences; for Hap2, the repetitive sequence content was 70.90%, and LTRs comprised 36.14% of the repetitive sequences (Supplementary Table S5).

In the male *H. gyantsensis* genome, 36,482 genes were annotated, and 33,238 were present in at least one database: Swissprot, KEGG, TrEMBL, nr, InterPro, and GO. In the female *H. salicifolia* genome, 39,501 genes were annotated, and 34,811 were present in at least one of the

aforementioned databases. In the Hap1 genome, 45,937 genes were annotated, including 38,287 in at least one database. In the Hap2 genome, 39,854 genes were annotated, including 34,460 in at least one database (Supplementary Table S6). The BUSCO completeness scores for gene sets in the male *H. gyantsensis*, female *H. salicifolia*, male *H. salicifolia* Hap1, and male *H. salicifolia* Hap2 genomes were 98.9%, 98.8%, 98.9%, and 98.7%, respectively (Supplementary Table S7). These findings confirm the high quality of the four sea buckthorn genomes sequenced.

Comparative genomics analysis

To investigate the phylogenetic relationships of sea buckthorn, we conducted a gene family analysis of 13 species, including sea buckthorn and other plants from the order Rosales (Supplementary Fig. S5). A phylogenetic tree was constructed using 1,272 single-copy genes from these genomes. Our results(Fig. 2a) indicate that the genus *Hippophae* and *Elaeagnus* diverged approximately 19.89 million years ago (mya), and there was a notable expansion of gene families preceding their divergence. Within *Hippopha*e, *H. tibetana*, *H. rhamnoides*, and Fructus Hippophae were grouped in Clade 1, whereas *H. salicifolia* and *H. gyantsensis* comprised Clade 2. *H. salicifolia* has recently experienced two whole-genome duplication (WGD) events(Fig. 2b), which is consistent with the results of previous studies of sea buckthorn [24,26-29]. Synteny analysis (Fig. 2c) revealed one-to-one homology relationships between chromosomal segments of *Hippophae* and *Elaeagnus* species[24,25], which indicates that they have a shared history of two WGD events. However, the synteny blocks within *Hippophae* were

shorter than those in *Elaeagnus* (Supplementary Fig. S6), indicating that the sea buckthorn genome has undergone a higher frequency of chromosomal rearrangements.

SDR characteristics of *H. gyantsensis* and *H. salicifolia*

To identify sex-linked regions (SLRs) in *H. salicifolia*, we conducted whole-genome resequencing on two mixed pools: one consisting of 14 female individuals and the other 14 male individuals. This generated 138.68 Gb and 129.15 Gb of sequencing data for the female and male pools, respectively. Through a comparative analysis of genome coverage depth, we identified a distinct SLR on Chr02 in Hap2 (Fig. 3a & 3b). Males exhibited a coverage depth of approximately 50% of the genome-wide average, whereas females had negligible coverage in this region; this region was designated as Y-SLR. Using a similar approach for Hap1, we identified an X-SLR (Fig. 3c&3d), wherein females displayed average coverage depths, and males showed reduced coverage depths. The Y-SLR in *H. salicifolia* spanned 29.71 Mb and was positioned between 25.85 Mb and 55.55 Mb on Chr02, which comprised 32.88% of the chromosome's length and encompassed 249 genes. The chromosome was bookended by undifferentiated pseudoautosomal regions (PARs), totaling 60.64 Mb. Conversely, the X-SLR extended 68.02 Mb from 25.83 Mb to 93.86 Mb on Chr02, comprising 52.96% of the chromosome's length; this region included 334 genes and contained a PAR measuring 60.41 Mb, which was similar to the length of the Y-PAR (Supplementary Fig. S7).

During sex chromosome evolution, genes within SLRs often become degraded and show reduced recombination, which affects the expression and functions of genes [31]. The results of this study provide an in-depth classification and analysis of the genes within the X- and Y-

SLRs in the genus *Hippophae* to clarify the evolutionary mechanisms of the sex chromosomes. Genes in the SLRs were classified into four categories based on homology and origin: "ancestral" genes, "acquired" genes, duplicated genes, and unique genes. The Y-SLR contained 249 genes, which comprised 99 ancestral, 73 duplicated, 53 acquired, and 24 unique genes; the X-SLR contained 100 ancestral, 82 duplicated, 120 acquired, and 31 unique genes (Supplementary Table S8). The similarity in ancestral gene numbers between the X- and Y-SLRs indicates that there was no significant difference in the degree of degradation of these regions. However, the greater number of genes in X-SLR was attributed to a greater number of insertions of acquired genes. Further analysis indicated that the SLRs of *H. salicifolia* were close to the centromere, which is an area enriched with repetitive sequences. In the Y-SLR, these repetitive sequences comprised 90.58% of the 26.91 Mb region; in the PAR, they comprised 55.32% of this region. The content of repetitive sequences was higher in the X-SLR (94.74%) than in the Y-SLR within the 64.44 Mb region, including 54.86% in the PAR (Supplementary Fig. S8). Although the repetitive sequence content was similar in the PAR regions of both sex chromosomes, the content of repetitive sequences was significantly higher in the X-SLR than in the Y-SLR. These findings indicate that the higher number of acquired genes and repetitive sequences in the X-SLR is likely the main cause of the pronounced morphological divergence between the X and Y chromosomes.

Evolution of the Sex Determination Region in *H. salicifolia*

To elucidate the origin of the sex chromosomes in the genus *Hippophae*, we compared Chr02 in *H. salicifolia* with the genomes of *H. gyantsensis*, *H. rhamnoides*, *Elaeagnus*

moorcroftii, and *Elaeagnus mollis* (Fig. 4a) (Supplementary Fig. S9). The synteny analysis identified two major collinear blocks adjacent to the SLRs on Chr02 of *H. salicifolia*, which were designated as L and R. Four copies of each of these two blocks were present within *Hippophae* and *Elaeagnus*, likely resulting from two WGD events shared by both genera. This suggests that these blocks predate the divergence of the two genera. On Chr02 of sea buckthorn, the L and R blocks were merged (L-R), a configuration that was not observed on other chromosomes or within the closely related *Elaeagnus*. To determine whether a breakage or fusion event occurred, we used *Ziziphus jujuba* and *Rhamnella rubrinervis* as outgroups for comparison. In both *Z. jujube* and *R. rubrinervis*, the L and R blocks remain separate (Supplementary Fig. S10), indicating that these blocks were likely independent in the ancestral chromosomes of the both genera. Integrating these findings with the results of prior synteny analyses (in the comparative genomics section), we infer that following the WGD events, the *Hippophae* genus underwent more frequent chromosomal breakage and fusion events during diploidization. Specifically, a lineage-specific chromosomal fusion event occurred, resulting in the merging of two ancestral chromosomes to form Chr02. This fusion might have had a profound effect on the sex determination and reproductive strategies of *Hippophae*.

We performed a microsynteny analysis in which the Y-SLR of *H. salicifolia* was compared with homologous regions in the Y-SLR of other *Hippophae* species and *Elaeagnus* to clarify the evolution of these regions following the formation of sex chromosomes. The collinear regions were categorized into nine distinct blocks(A-G) through the comparison of chromosomal segment synteny (Supplementary Fig. S11 and Table S9). Discrepancies in the positions of blocks A and B between *Elaeagnus* and *Hippophae* were noted (Fig. 4b). However,

the ancestral configuration of these blocks remains uncertain because of a lack of systenic information from outgroup comparisons; we hypothesize that *Hippophae* and *Elaeagnus* have structurally diverged. A comparative analysis of the block arrangement and orientation across closely related species has revealed that the Y-SLR in *H. salicifolia* is more structurally conserved than the X-SLR. Specifically, in the X-SLR, blocks A2, G, F, and E1 have been translocated, and block A1 has been inverted. In contrast, only a single translocation involving block E2 was identified in the Y-SLR, while block D is reversed in X-SLR or Y-SLR, or both. We computed the Ks values for homologous gene pairs within these blocks to infer the sequences for the different structural variations (Supplementary Table S10). Block D had the highest Ks value (Ks=0.1266), followed by block F (Ks=0.082), block E1 (Ks=0.0442), block E2 (Ks=0.0409), block A2 (Ks=0.03854), block A1 (Ks=0.01429), and block G (Ks=0.0037). The elevated Ks value of block D indicates that its structural changes occurred earlier than those of other blocks, and the lower Ks values for A1 and G suggest that their structural changes occurred recently.

Chromosomal rearrangement events are the main drivers of recombination suppression and sex chromosome differentiation [14]. Recombination suppression can promote increased sequence divergence in different regions of the sex chromosomes, forming so-called "evolutionary strata", which reflect the history and chronological order of structural variations in the evolution of sex chromosomes [32]. The synonymous substitution rate (Ks) is employed as a metric to quantify the degree of sequence divergence between homologous gene pairs.

A higher Ks value generally indicates an earlier divergence event. We analyzed the evolutionary rates of genes within the Y-SLR of *H. salicifolia* by calculating the Ks values of

XY homologous gene pairs and mapping these values onto their corresponding evolutionary strata. The distribution of Ks values (Supplementary Table S10) did not exhibit clear clustering of high or low Ks values across specific genomic blocks, a phenomenon potentially attributable to the influence of structural variations on Ks values. Structural variations, such as inversions, may lead to localized increases in Ks values, necessitating a comprehensive consideration of their impact in our analysis. Accordingly, we adjusted the Ks values for certain blocks affected by structural variations. Nevertheless, it is highly probable that Block D belongs to an ancient stratum. Furthermore, considering that the sex chromosomes of *Hippophae* originated from the fusion of two chromosomes, collinearity alignment results identified the region between Blocks C and D as the fusion point. Chromosomal fusion, by juxtaposing previously independent gene regions, promotes the formation of linkage groups associated with sex differentiation, thereby potentially driving the evolution of sex determination.

Thus, we propose that both Blocks D and C likely belong to an ancient stratum. The abundance of repetitive sequences between Blocks C and D may result from recombination suppression during sex chromosome evolution, and their accumulation likely contributes to the stability of the sex-determining region, a hallmark of sex chromosome evolution. Based on the distribution of Ks values, the influence of structural variations, chromosomal fusion events, and the distribution of repetitive sequences, we divided the Y-SLR into two putative evolutionary strata (Supplementary Fig. S12). Stratum 1 (including blocks C and D) was located from 33.13 Mb to 49.72 Mb, and the average Ks value was 0.1041 for the 43 homologous gene pairs in this region. Stratum 2-1 (including blocks A and B) was located from 25.88 Mb to 33.08 Mb and contained 19 homologous gene pairs with an average Ks value of 0.0574. Stratum 2-2

(including blocks E, F, and G) was located from 50.19 Mb to 55.61 Mb and contained 38 homologous gene pairs with an average Ks value of 0.0418 (Fig. 4c).

Genes from different evolutionary strata exhibit distinct phylogenetic patterns. In dioecious genera, where all species are dioecious, sexual differentiation likely predates species divergence. In such cases, genes within the ancient strata of the SDR would have diverged prior to species differentiation. Consequently, homologous genes originating from the same gametophyte tend to cluster together. In contrast, genes from more recent strata may have diverged after species differentiation, leading to a tendency for homologous genes from the same species to cluster together [7,17]. To further validate the proposed strata, we constructed a phylogenetic tree for single-copy genes within the SLRs and analyzed its topology. In Stratum 1, 17 out of 34 single-copy genes exhibited an ancient origin, while in Strata 2-1 and 2-2, only 1 and 3 genes, respectively, exhibited an ancient origin (Fig. 4d). These results indicate that Stratum 1 represents a more ancient partition of the sex chromosomes in *H. salicifolia*, and Strata 2-1 and 2-2 are more recent. Integrating the Ks analysis of structural variations, we hypothesize that the inversion event in Block D may have facilitated the differentiation of Stratum 1 by suppressing recombination, thereby accelerating the functional specialization of the sex chromosomes. Conversely, the inversions in Blocks F, E, and A2 are closely associated with the formation of Stratum 2. These findings suggest that chromosomal structural variations between X- and Y-SLRs might have played a key role in promoting the spread of recombination suppression and the evolution of sex chromosomes.

Sea buckthorn sex determination candidate genes

To identify candidate genes involved in sex determination in sea buckthorn, we analyzed the transcriptional profiles of male and female mixed flower buds at various developmental stages of *H. gyantsensis* and *H. salicifolia* (Supplementary Table S11). We utilized transcriptome data, compared these data with the Y-SLR of *H. salicifolia*, and calculated the transcripts per million (TPM) values of the transcripts (Supplementary Table S12). This analysis identified 11 genes that are specifically expressed in males (Fig. 5). We conducted further analysis on the 11 male-specific expressed genes and found that five of them lacked homologous counterparts in male *H. gyantsensis*, leading to their preliminary exclusion.

As previously mentioned, if all species within a genus are dioecious, sexual differentiation likely predates species divergence. Consequently, sex-determining genes typically exhibit phylogenetic similarities with genes from ancient evolutionary strata, meaning that homologous genes originating from the same gametophyte tend to cluster together. This is because the divergence of sex-determining genes between males and females occurred before species differentiation [7,17]. Therefore, we analyzed the phylogenetic tree topology of the remaining six genes. The results revealed that two of these genes displayed a topology inconsistent with our expectations(Supplementary Fig. S13), showing a clustering pattern where homologous genes from males and females of the same species grouped together. Three genes (*Hsam2h02g1262*, *Hsam2h02g1315*, and *Hsam2h02g1436*) did not have corresponding homologous genes identified within the X-SLR of *H. salicifolia* and *H. gyantsensis* (Supplementary Fig. S14). However, homologous genes were found on autosomes, suggesting

that these genes were likely duplicated from autosomal regions and inserted into the Y-SLR, where they may function as male-specific genes potentially involved in sea buckthorn sex determination. The gene tree structure of *Hsam2h02g1248* largely aligned with our expectations (Supplementary Fig. S15), with homologous genes from the Y-SLR of different sea buckthorn species clustering together and those from the X-SLR forming a separate cluster. Although the ideal phylogenetic tree structure would have shown *Hsam2h02g1248* and *Hgyam02g1367* grouping together rather than exhibiting a sequential divergence pattern, minor variations can easily influence gene tree topology. Thus, *Hsam2h02g1248* remains a strong candidate for a sex-determining gene. Among the four selected genes, three genes (*Hsam2h02g1248*, *Hsam2h02g1262*, and *Hsam2h02g1314*) are located within the ancient evolutionary stratum we previously identified (Supplementary Fig. S16). These genes are considered the most promising candidates for sea buckthorn sex determination.

The gene *Hsam2h02g1248*, a homolog of *FAR*, was highly and specifically expressed (TPM > 100) during the male flower bud development phase in *H. gyantsensis*, and it was minimally expressed in stems or leaves. This expression profile resembles that of *AtFAR2* in *Arabidopsis thaliana*, which is thought to be involved in male sterility [33]. Furthermore, the homologous gene of *Hsam2h02g1248* within the X-SLRs has undergone fragmentation (Supplementary Fig. 17). By comparing resequencing data from male and female *H. salicifolia* (Supplementary Fig. 18), we identified an insertion in the X-SLR homologous gene, which likely caused its disruption. Additionally, compared to *Hsam2h02g1248*, the X-SLR homologous gene lacks several sequences, potentially leading to the loss of its original function. The role of this gene and its pattern of expression are intimately linked to the masculinization

of sea buckthorn, which makes it a male-stimulating factor (M). *Hsam2h02g1315* and *Hsam2h02g1262* are male-specific and lack X-SLR homologs in sea buckthorn (Supplementary Fig. S19&S20). *Hsam2h02g1315* is a homologous gene of FD, and the homologous gene of *Hsam2h02g1262* in *A. thaliana* is *AtRRP44A*. Both of them are closely related to flower development [34,35]. One of these two genes might be a female suppressor factor in sea buckthorn, yet additional experiments are needed to verify this possibility.

Discussion

Sexual differentiation is a critically important component of the reproductive strategy of members of the genus *Hippophae*, and studies of sexual differentiation can provide insights into the physiological and molecular mechanisms by which sea buckthorn adapts to different environments, which have implications for ongoing efforts to genetically improve sea buckthorn and regulate the sex determination process. However, the lack of complete genomic information for male sea buckthorn species limits studies of sexual differentiation. The absence of male genomic data has also impeded studies of the molecular mechanisms of sex determination in sea buckthorn, the gene expression regulatory networks during sexual differentiation, and the genetic basis of sex-specific traits. A recent study of *H. tibetana* has indicated that its sex chromosomes are located on Chr02; however, the SDR interval was not determined in this study [26]. We sequenced the genomes of male *H. gyantsensis* and both male and female *H. salicifolia*; we obtained four sets of genomic data (including two haplotype datasets for male *H. salicifolia*).

H. salicifolia and *H. gyantsensis* have recently undergone two WGD events, and this finding is consistent with that observed in other species within the family Elaeagnaceae [24,25]. The recent WGD event is specific to Elaeagnaceae. Chromosomal breakage and fusion events following WGD events have been more common in *Hippophae* than in *Elaeagnus*, which has also experienced two WGD events [24,25]. Previous studies have indicated that chromosomal breakage and fusion play a major role in the formation of sex chromosomes [36]. These events can lead to substantial changes in genome structure, which affects gene expression and function and can sometimes even result in the formation of new species [37]. In our study, the sex chromosomes of both *H. salicifolia* and *H. gyantsensis* were located on chromosome Chr02. Chr02 in *Hippophae* species was derived from the fusion of two distinct ancestral chromosomes (A and B). One end of the fusion fragment of chromosome A was located near the centromeric region of the chromosome. When it fused with chromosome B, it introduced a large number of repetitive sequences, which facilitated the subsequent cessation of recombination in this region to form the SLR [38]. Sex chromosomes are known to have independently evolved through similar mechanisms in other lineages, such as the SLRs of papaya and kiwifruit, which have evolved from the pericentromeric regions [39–41]. Throughout the evolutionary process, WGD events, chromosomal rearrangements, and the lack of recombination around the centromeric regions have facilitated the origin of sex chromosomes in *Hippophae* species.

Plant sex chromosomes are derived from a pair of autosomes, and recombination suppression is essential for the evolution of sex chromosomes. Chromosomal rearrangements, heterochromatinization, the accumulation of repetitive sequences, and DNA methylation can all lead to recombination suppression [42,43], chromosomal rearrangements are the main cause

of recombination suppression [2,44]. In papaya, two large inversions on the Y chromosome have promoted the differentiation of XY, which is the main cause of recombination suppression in the XY chromosomes [40]. In our study, the evolution of the sex chromosomes in *H. salicifolia* appears to be related to the frequent chromosomal rearrangements in the X- and Y-SLRs. Analysis of the arrangement and orientation of homologous blocks within the SLRs revealed that the Y-SLR structure of *H. salicifolia* was more conserved, with multiple blocks in the X-SLR undergoing chromosomal inversions or translocations and one block in the Y-SLR undergoing translocation. These structural variations are closely related to the spread of recombination suppression.

Based on the structural variations and the Ks values of the evolutionary strata, we simulated the evolution of the sex chromosomes in *H. salicifolia*: the oldest structural variations occurred over a narrow range near the centromere, which promoted the spread of recombination suppression and the formation of ancient strata. Subsequently, large-scale structural variations occurred around the ancient strata, which promoted the spread of recombination suppression and the formation of new strata. X-linked structural variations do not frequently drive the evolution of sex chromosomes; rearrangements in the Y-SLR have been identified in most sex chromosomes studied to date, such as inversions in papaya and willows in the Y chromosome [45,46]. However, recent studies of Silene latifolia have shown that recombination suppression may stem from an inversion in the X-SLR [47]. Many current theories of sex chromosome evolution are based on the premise of Y-linked inversions [48]; however, the results of our study indicate that X-linked structural variations might be selected and fixed to suppress recombination between XY chromosomes.

Sex-determining genes are the main drivers of reproductive organ differentiation in dioecious plants. However, these genes have been isolated and identified in only a handful of species, including kiwifruit, persimmon, asparagus, and poplar [18,19,48,49]. We were unable to identify homologs of these known sex-determining genes in the SDRs of sea buckthorn, indicating that a unique mechanism promotes maleness and inhibits femaleness in this genus. Typically, sex-determining genes are specifically expressed in male flowers and exhibit a phylogenetic pattern in which homologs from various species cluster together. Within the sea buckthorn's MSY region, we identified three genes that appear consistent with these criteria. Notably, *Hsam2h02g1248* is a candidate male-promoting factor, and its Arabidopsis homolog, *FAR2*, is a known male sterility factor [33]. This gene also shares significant evolutionary similarity with the kiwifruit sex-determining gene [19], *FrBy*, which is present during the andromonoecious phase and is differentially maintained in males after sexual differentiation. Our findings are consistent with this pattern, with *Hsam2h02g1248* persisting in males and losing functionality in females after sexual differentiation. Under normal circumstances, the sex determination region has two genes that jointly determine sexual differentiation. Based on the characteristics of SLRs, we speculate that sea buckthorn should belong to a species with dual-gene sex determination, that is, in addition to a male-promoting factor, there is also a female-suppressing factor. *Hsam2h02g1315* and *Hsam2h02g1262* are our inferred female-suppressing candidate genes. These two genes lack homologs in the X-SLR but have homologs on the autosomes, suggesting they may have originated from autosomal gene duplication. Gene duplication plays a key role in the formation of female-inhibiting factors; for example, the female-inhibiting factor *SyGl* in kiwifruit is derived from an autosomal gene duplication that

changes its expression pattern to inhibit carpel development [18]. The sex-determining genes *FERR-R* and *ARR17* in poplar species also arise from duplication [48,50]. *Hsam2h02g1315* and *Hsam2h02g1262* are both related to flower development. Additional experiments are needed to determine whether they have acquired new functions after duplication.

Methods

Sample collection

All samples required for this study of *H. gyantsensis* were obtained from Jiangzi County, Shigatse City, Tibet Autonomous Region, China. All samples of *H. salicifolia* were collected from Gyirong County, Tibet Autonomous Region, China. For genomic sequencing, we collected leaf samples from one male *H. gyantsensis*, one female *H. gyantsensis*, and one male *H. salicifolia*. Additionally, to assist with genome annotation, we collected leaf and stem samples from three male and three female individuals of *H. salicifolia* and two male and two female individuals of *H. gyantsensis* for transcriptome sequencing, with two replicates for each sample. To identify the SLRs in *H. gyantsensis* and *H. salicifolia*, we performed whole-genome resequencing on two mixed pools: one of 14 female H. salicifolia individuals and one of 14 male H. salicifolia individuals. Additionally, to elucidate the differential gene expression patterns between male and female individuals, we collected mixed floral bud samples from both *H. gyantsensis* and *H. salicifolia* for transcriptome sequencing analysis.

Genome sequencing

Genomic DNA samples for short-read sequencing were extracted from the leaves using the CTAB method [51]. Libraries were constructed using the MGIEasy Universal DNA Library Prep Kit V1.0 (CAT#1000005250, MGI) per the standard protocol. The qualified libraries were sequenced on the DNBSEQ-T7RS platform. Total RNA was extracted by grinding tissue using the CTAB-LiCl method (Plant) on dry ice and processed per the manufacturer's protocol. After meeting the quality control standards, an appropriate amount of total RNA was used to construct a library for sequencing using the DNBSEQ-T7RS platform (RRID:SCR_017981).

ONT regular DNA was extracted using the Grandomics Genomic DNA Kit per the manufacturer's protocol. DNA samples were accurately quantified using a Qubit® 3.0 Fluorometer (Invitrogen, USA). Long, size-selected DNA fragments were then extracted using the PippinHT system (Sage Science, USA). DNA was repaired and adapters were attached to the ends using an SQK-LSK110 kit. The concentrations of library fragments were quantified using a Qubit® 3.0 Fluorometer. The DNA library was loaded into the primed Nanopore PromethION sequencer (Oxford Nanopore Technologies, UK) flow cell. After DNA extraction, the SMRTbell target size library was constructed using the 15 kb preparation solution according to PacBio's standard protocol (Pacific Biosciences, CA, USA) for HiFi sequencing. Sequencing was conducted on the Grandomics PacBio Sequel II instrument. Genomic DNA was extracted for the Hi-C library from leaves. Next, we constructed the Hi-C library and obtained sequencing data using the DNBSEQ-T7RS platform.

Genome Size Assessment and Genome Assembly

The K-mer (k=21) frequency table of clean NGS reads was generated using the Jellyfish (RRID:SCR_005491) v2.3.0 [52] program, and the genome size and heterozygosity of the three sea buckthorn species were assessed using GenomeScope (RRID:SCR_017014) v2.0 [53]. NextDenovo (RRID:SCR_025033) v2.4.0 was used to generate the preliminary assembly from the ONT clean sequencing data for male *H. gyantsensis* and female *H. salicifolia*. NextPolish (RRID:SCR_025232) v1.3.1 [54] was used to perform three rounds of ONT and NGS iterative error correction on the initially assembled genomes. For male *H. salicifolia* sequenced using HiFi sequencing, raw BAM files obtained from sequencing were converted into gz format using SAMTOOLS (RRID:SCR_002105) v1.18 [55], and Hifiasm (RRID:SCR_021069) v0.19.9-r616 [56] was used to assemble contigs by integrating HiFi and HiC reads. After obtaining the preliminary assemblies of the three sea buckthorn genomes, Minimap2 (RRID:SCR_018550) v2.28-r1209 [57] was used to compare the third-generation sequencing data with the genome, and the read coverage depth and coverage breadth for each contig were calculated. Redundant sequences and small genome sequences were identified and removed based on read coverage using Purge_Dups (RRID:SCR_021173) v1.2.5 [58] software. Cleaned Hi-C reads were input into Juicer (RRID:SCR_017226) v1.6 software [59] for ALLHiC (RRID:SCR_022750) v210623 [60] chromosome construction. Manual adjustments for inversions and shifts in the assembly were made using Juicebox (RRID:SCR_021172) [61] to obtain three chromosome-level sea buckthorn genomes. The final Hi-C contact map was visualized using HiCExplorer (RRID:SCR_022111) v3.7.3 [62]. To improve the quality of the chromosome assembly, the

male *H. salicifolia* genome was corrected using NextPolish2 [63], using clean HiFi and NGS data as input. The completeness of the genome assembly was evaluated using BUSCO (RRID:SCR_015008) v5.7.0 [64] and the embryophyta-odb10 dataset.

Genome annotation

Repetitive sequences of the three sea buckthorn genomes were annotated using the Extensive de novo TE Annotator (EDTA (RRID:SCR_022063) v2.1.2 [65]). After obtaining the EDTA-annotated transposable element (TE) library, TEsorter v1.4.6 [66] was used to reclassify the "LTR-unknown", followed by deepTE [67] classification for the "TEsorter-unknown". Finally, the three obtained TE databases were merged, and the repetitive sequences were masked using RepeatMasker (RRID:SCR_012954) v4.1.2-p1 [68].

Homology-based protein alignment, *de novo* prediction, and transcriptome prediction were used for gene structure annotation. HISAT2 (RRID:SCR_015530) v2.2.1 [69] was used to align clean reads from the transcriptome to the genome, and the resulting alignment files were used as input for Braker3 [70,71]. GeneMark-ET [72] was used for gene structure identification, and Augustus was used for model training to obtain high-confidence gene structures based on the transcript and *de novo* prediction results. *H. rhamnoides* protein sequences were input into Braker3, and GeneMark-EP [73] combined with ProtHint was used for sequence alignment. Augustus was used for model training to obtain high-confidence gene structures based on the known proteins and *de novo* prediction results. The results obtained via Braker3 were then integrated using TSEBRA [74] (default parameters); this was followed by quality filtering and normalization using scripts from the MAKER (RRID:SCR_005309) package v3.01.04 [75],

which ultimately generated a gff annotation file that describes genetic structural information. TBtools (RRID:SCR_023018) [76] was used to extract the longest transcript, gffread (RRID:SCR_018965) v0.12.8 [77] was used to extract the corresponding cds sequence, and then SeqKit (RRID:SCR_018926) [78] was used to translate it into a protein sequence. BUSCO (RRID:SCR_015008) v5.7.0 [64] was used to assess the integrated annotation proteins.

InterProScan (RRID:SCR_005829) v5 [79] was used to conduct a search of the annotated genes with the parameters set to '-appl Pfam, CDD'. The predicted genes were functionally annotated by scanning the NCBI non-redundant protein (nr), TrEMBL, and Swiss-Prot [80] databases using DIAMOND (RRID:SCR_009457) v2.0.15 [81] with default parameters. eggNOG-mapper (RRID:SCR_021165) [82] with default parameters was used to obtain Kyoto Encyclopedia of Genes and Genomes (KEGG (RRID:SCR_012773) [83]) and Gene Ontology (GO (RRID:SCR_017505) [84]) annotations.

Comparative genomics analysis

Protein sequences of *H. rhamnoides*, *H. salicifolia*, *H. gyantsensis*, Fructus Hippophae, *H. tibetana*, *E. moorcroftii*, *E. mollis*, *Ziziphus jujuba*, *Fragaria daltoniana*, *Vitis vinifera*, *Morus notabilis*, *Cannabis sativa*, and *Rhamnella rubrinervis* were selected for gene family analysis. OrthoFinder (RRID:SCR_017118) v2.5.5 [85] was used to identify orthologous groups with the parameters set as '-M msa', and single-copy genes were used to construct a phylogenetic tree. The SeqKit (RRID:SCR_018926) [78] tool was used to extract the coding sequences of single-copy orthologous genes, and they were aligned using MUSCLE (RRID:SCR_011812) v3.8.31 [86]. The SeqKit (RRID:SCR_018926) [78] tool was used to concatenate the sequences

into supergenes, which were then trimmed using trimAl (RRID:SCR_017334) v1.4 [87] (-gt 0.6 -cons 60). The trimmed sequences were used to construct a phylogenetic tree using the maximum likelihood method in RAxML (RRID:SCR_006086) v1.1.0 software [88]. Three fossil calibration time points were obtained from the TimeTree (RRID:SCR_021162) database [89]: *V. vinifera* and *Z. jujuba* [109.8 to 122.4 million years ago (mya)], *Z. jujuba* and *C. sativa* (68.5 to 85.2 mya), and *C. sativa* and *M. notabilis* (48.9 to 70.9 mya). The MCMCtree program in the PAML (RRID:SCR_014932) v.4.10.0 software package [90] was used to estimate the divergence times of each node in the phylogenetic tree obtained in the previous step. The phylogenetic tree with divergence times and the sorted gene family results (i.e., gene families with significant differences in copy number were removed) were used to construct a phylogenetic tree containing information on gene family expansions and contractions using the CAFE (RRID:SCR_005983) V5 [91] program.

We used WGDI v0.6.5 [92] to detect whole-genome duplication events. DIAMOND (RRID:SCR_009457) v2.0.15 [81] was used to identify homologous genes with an e-value that did not exceed 1e-5. WGDI was used to identify collinear genes with the parameter '-icl'. Next, the 'ks' parameter in WGDI was modified to calculate Ks values, and the 'bi' and 'c' parameters were modified to filter the collinear results. Finally, the 'kp' parameter was modified to calculate the Ks peak, and the 'kf' parameter was used to fit and visualize the results. JCVI (RRID:SCR_021641) [93] (MCscan Python version) was used for whole-genome collinearity analysis between *Hippophae* and *Elaeagnus* plants.

Sex determination interval and characteristics

The *H. salicifolia* resequencing data were compared with the Hap1 and Hap2 reference genomes using BWA-MEM2 (RRID:SCR_022192) v2.2.1 [94] software. Based on the comparison results, a window was established within an interval of 50 kb, with a sliding window of 5 kb for stepwise progression. BEDTools (RRID:SCR_006646) v2.30.0 [95] was used to calculate the coverage depth of each window, and the R package ggplot2 (RRID:SCR_014601) [96] was used to visualize the results. The chromosomes and intervals of the SDR were located assuming that male-specific regions have no female read coverage and male read coverage was half that of other regions. After obtaining the initial SLRs, Integrative Genomics Viewer(IGV) (RRID:SCR_011793) software [97] was used to view the precise SLR range by comparing different regions.

We mainly used JCVI (RRID:SCR_021641) [93] and BLASTP (RRID:SCR_001010) v2.14.0+ [98] to analyze the origin of genes within the X- and Y-SLRs of *H. salicifolia*. To begin, we conducted a collinearity analysis by comparing the X-SLR and Y-SLR regions with each other and with two species of the genus *Elaeagnus*. Given that both *Hippophae* and *Elaeagnus* belong to the family Elaeagnaceae and diverged after experiencing two WGD events, we designated the copies of Chr02 in *Elaeagnus* and *Hippophae* as the putative ancestral chromosome. Genes within the SLRs that exhibited homology with those in the putative ancestral chromosomes of *Hippophae* and *Elaeagnus* were classified as "ancestral" genes. For the remaining genes, we performed a BLASTP (RRID:SCR_001010) v2.14.0+ [98] search to identify homologous sequences. Genes with homologous sequences on autosomes (non-

putative ancestral chromosomes) were categorized as "acquired" genes, which may have been gained through duplication and insertion events of autosomal genes. Genes without homologous sequences were classified as "specific" genes, with those exclusively present in the X-SLR termed X-SLR-specific genes and those exclusively present in the Y-SLR termed Y-SLR-specific genes. Finally, genes with duplicated copies within the SLRs were categorized as "duplicated" genes. Specifically, if two duplicated copies were identified among the "ancestral" genes, one was assigned to the "duplicated" gene category, and the same classification method was applied to other gene categories.

Sex chromosome evolution of *H. salicifolia* and *H. gyantsensis*

For evolutionary analysis, we first used JCVI (RRID:SCR_021641) [93] to analyze the collinearity Chr02 of *H. salicifolia* with *H. gyantsensis*, *H. rhamnoides*, *E. moorcroftii*, *E. mollis*, *R. rubrinervis*, and *Z. jujuba*. JCVI (RRID:SCR_021641) [93] was also used to analyze the microsynteny of the SLR regions in *H. salicifolia* with *H. gyantsensis*, *H. rhamnoides*, Fructus Hippophae, *E. moorcroftii*, and *E. mollis*. Based on the identified homologous blocks, we analyzed block rearrangements in *Hippophae* and *Elaeagnus* plants. To analyze the substitution rates of X- and Y-linked genes, we created pairwise alignments including X-linked and Y-linked homologous genes, and then used ParaAT.pl v2.0 [99] and KaKs_Caculater v3.0 [100] to calculate synonymous and non-synonymous substitution rates. Then, based on the block coordinate, we calculated the average Ks value of homologous genes within each block. Different phylogenetic patterns were observed for genes in the "ancient" and "recent" regions of the sex chromosomes. Therefore, OrthoFinder (RRID:SCR_017118) v2.5.5 [85] was used to

identify single-copy orthologous genes within the seabuckthorn SLRs, and MAFFT (RRID:SCR_011811) v7.520 [101] was used to align the sequences in each set of single-copy orthologs. We also conducted searches of the corresponding chromosomes of *E. moorcroftii* and *E. mollis* using BLASTP [98] to identify corresponding homologous genes. Finally, we used IQ-TREE2 v2.2.0 [102] to generate the gene trees with 1,000 bootstrap replicates.

Screening of sex-determining candidate genes

To identify candidate genes involved in sex determination, we first conducted an analysis of differentially expressed genes between males and females. Fastp (RRID:SCR_016962) [103] software was used to filter the transcriptome data of male and female flower buds of *H. gyantsensis* and *H. salicifolia*, and STAR (RRID:SCR_004463) [104] was used to align them with the male *H. salicifolia* Hap2 genome; RSEM (RRID:SCR_000262) [105] software was used to calculate expression levels. Finally, we utilized the R package ggplot2 [96] to generate a heatmap by plotting the logarithm-transformed TPM values. Using the same method, we also calculated the expression levels of the stem and leaf transcriptome data from both female and male individuals of *H. gyantsensis* and *H. salicifolia* (the transcriptome data used were the same as those used for assisting genome annotation, including the published data for the stem and leaf transcriptome of the female *H. gyantsensis* [PRJNA997223]) [28].

After identifying genes specifically expressed in males, we further screened them based on their phylogenetic positions relative to those of homologous genes. First, we extracted the protein sequences of genes specifically expressed in males in the SDR region of *H. salicifolia* as queries and used data from *H. rhamnoides*, *H. tibetana*, male *H. gyantsensis*, female *H.*

gyantsensis, male *H. salicifolia*, female *H. salicifolia*, and Fructus Hippophae as protein databases; *E. moorcroftii* and *E. mollis* were used as outgroups in homologous gene searches. Based on the obtained BLASTP (RRID:SCR_001010) v2.14.0+ [98] alignment results, we retained sequences with identity greater than 70 and performed sequence alignment using MAFFT (RRID:SCR_011811) v7.520 [101] software. Gene trees were constructed using IQ-TREE2 v2.2.0 [102], and Figtree was used to visualize the trees.

Acknowledgements

This research was supported by the Second Tibetan Plateau Scientific Expedition and Research (STEP) program (2019QZKK0502), Key Research and Development Program for Bureau of Science and Technology of Xizang Autonomous Region (XZ202401ZY0006), Key Research, Development and Transformation Program for Shigatse City Bureau of Science and Technology (RKZ2023ZY-03), Yunling Scholar Project to Yang Yongping.

Additional Files

Supplementary Fig. S1. Distribution of K-mer (K = 21) frequency in sequencing reads of the three seabuckthorn plants. (a) Distribution of K-mer frequency for male *H. salicifolia* from Illumina sequencing. (b) Distribution of K-mer frequency for female *H. salicifolia* from Illumina sequencing. (c) Distribution of K-mer frequency for male *H. gyantsensis* from Illumina sequencing.

Supplementary Fig. S2. Hi-C heatmap of chromosomes for male *H. gyantsensis* (a), female

H. salicifolia (b), Hap1 (c), and Hap2 (d) of male *H. salicifolia*.

Supplementary Fig. S3. Circos plot of the genomic landscape of male *H. gyantsensis*. (a) Pseudochromosome. (b) Gene density. (c) Repeat sequences density. (d) Ty3 density. (e) Copia density. (f) GC content.

Supplementary Fig. S4. Circos plot of the genomic landscape of female *H. salifolia*. (a) Pseudochromosome. (b) Gene density. (c) Repeat sequences density. (d) Ty3 density. (e) Copia density. (f) GC content.

Supplementary Fig. S5. Statistics of orthogroups in different plants defined by OrthoFinder.

Supplementary Fig. S6. The synteny dot plot within the genome of *E. mollis* and *H. salicifolia*. (a) *E. mollis* (b) *H. salicifolia*

Supplementary Fig. S7. Hi-C Schematic Diagram of the Sex Chromosomes in *H. salicifolia*. (a) Hap1 Chr02. (b) Hap2 Chr02. (c) the schematic of Chr02 in Hap1, with the yellow region highlighting the X-SLR. (d) the schematic of the sex chromosome in Hap2, where the yellow region denotes the Y-SLR.

Supplementary Fig. S8. Density plot of repetitive sequences on Chr02 for Hap1 (a) and Hap2(b). Pink dots represent the density of pseudoautosomal repeats, and blue dots represent the density of repeats in sex-linked regions.

Supplementary Fig. S9. Synteny relationship between Chr02 of Hap2 and its closely related species: (a) Syntenic point map of Hap2 Chr02 and whole genome of *H. gyantsensis* (b) Syntenic point map of Hap2 Chr02 and whole genome of *E. moorcroftii*.

Supplementary Fig. S10. Synteny relationship between chromosome 2 of Hap2 and its closely related species: (a) Synteny relationship between Chr02 of Hap2 and *Z. jujuba*. (b)

Synteny relationship between Chr02 of Hap2 and *R. rubrinervis*.

Supplementary Fig. S11. Microsynteny of homologous blocks between the Y-SLR of Hap2 and its closely related species. (a) *E. mollis* (b) *E. moorcroftii* (c) *H. rhamnoides* (d) Fructus Hippophae (e) female *H. gyantsensis* (f) male *H. gyantsensis* (g) Hap1. The yellow collinearity blocks correspond to the Pseudoautosomal Regions (PARs) of the chromosomes.

Supplementary Fig. S12. The Ks values of XY homologous gene pairs within the Y-SLR are mapped to the positional coordinates of the Y chromosome. Blue represents gene pairs within stratum 1, red represents gene pairs within stratum 2-1, and yellow represents gene pairs within stratum 2-2.

Supplementary Fig. S13. Among the 11 male-specifically expressed genes, the phylogenetic trees of two genes are not consistent with the expected phylogenetic structure. 2Y-SLR represents the genes within the Y-SLR, and 2X-SLR represents the genes within the X-SLR. Furthermore, the genes within the Y-SLR are marked in red. (a) *Hsam2h02g1340* (b) *Hsam2h02g1384*.

Supplementary Fig. S14. Among the 11 male-specifically expressed genes, three are male-specific genes that lack corresponding homologous genes within the X-SLR. The genes within the Y-SLR are marked in red, the genes within the X-SLR are marked in blue. (a) *Hsam2h02g1262* (b) *Hsam2h02g1315* (c) *Hsam2h02g1436*.

Supplementary Fig. S15. Gene tree constructed by *Hsam2h02g1248* and its homologous genes. The genes within the Y-SLR are marked in red, the genes within the X-SLR are marked in blue.

Supplementary Fig. S16. The distribution of the four candidate genes within the SLR; three

genes are located in the presumed ancient strata, one gene (*Hsam02g1436*) is close to the PAR region.

Supplementary Fig. S17. The sequence alignment of *Hsam2h02g1248* with its homologous genes in the female- and male-specific linkage regions of *H. salicifolia* and *H. gyantsensis* reveals that gene fragmentation has occurred in the homologous genes of females.

Supplementary Fig. S18. Alignments of the resequencing reads from male and female individuals to the genes. (a) *Hsam2h02g1248*. (b) The homologous genes of *Hsam2h02g1248* in X-SLR were *Hsam1h02g1337*, *Hsam1h02g1338* and *Hsam1h02g1339*.

Supplementary Fig. S19. Alignments of the resequencing reads from male and female individuals to the gene *Hsam2h02g1262*.

Supplementary Fig. S20. Alignments of the resequencing reads from male and female individuals to the gene *Hsam2h02g1315*.

Supplementary Table S1. Sequencing statistics of male *H. gyantsensis,* female *H. salicifolia* and male *H. salicifolia.*

Supplementary Table S2. Statistics of Hi-C reads mapping for male *H. gyantsensis,* female *H. salicifolia* and male *H. salicifolia.*

Supplementary Table S3. Summary of male and female seabuckthorn genome assemblies.

Supplementary Table S4. Quality assessment of the completeness of female and male genome assemblies using the BUSCO tool.

Supplementary Table S5. Repeat sequence annotation statistics.

Supplementary Table S6. Functional annotation of predicted protein-coding genes of the male and female sea buckthorn genomes.

Supplementary Table S7. Quality assessment of the completeness of female and male gene sets with the BUSCO tool.

Supplementary Table S8. The classification of gene origins within the X- and Y-SLR.

Supplementary Table S9. The collinearity relationship between the X-SLR of *H. salicifolia* and the homologous regions of closely related species.

Supplementary Table S10. The Ks values of homologous genes in different blocks within the SLRs of *H. salicifolia*.

Supplementary Table S11. Statistics of transcriptome sequencing of female and male mixed flower buds in *H. gyantsensis* and *H. salicifolia*.

Supplementary Table S12. TPM values of genes in the SLR of *H. salicifolia* across different tissues in males and females.

Abbreviations

bp: base pairs; BUSCO: Benchmarking Universal Single-Copy Orthologs; CTAB: cetyl trimethyl ammonium bromide; Gb: gigabase pairs; HiC: high-throughput/resolution chromosome conformation capture; Mb: megabase pairs; Mya: million years ago; ONT: Oxford Nanopore Technologies; Pacbio: Pacific Biosciences; SLR: sex-link region; SDR: sex-determining region; MSY: male-specific region; Ks: synonymous substitutions per synonymous site; TPM: transcripts per million; NGS: next-generation sequencing; PAR: pseudoautosomal region;

Author Contributions

YPY, YQY and SHY designed the research. MYC and XYY performed the research. MYC, XYY, XL and ZLQ analyzed the data. MYC and YQY wrote the paper.

Competing Interests

The authors declare that they have no competing interests.

Ethics Statement

All plant molecular materials and specimens were collected with permission.

Data Availability

The sequencing data that support the findings of this study are openly available in the NCBI Sequence Read Archive (SRA) under BioProject accession number PRJNA1166656. The resequencing data for female and male *Hippophae salicifolia* can be found in the National Genomics Data Center (NGDC) under Project accession number PRJCA036349. The genome assembly and annotation data of the three seabuckthorns have been deposited in FigShare [106]. All supporting data and materials are available in the *GigaScience* database, GigaDB [107], with separate datasets for male *H. gyantsensis* [108] and female *H. salicifolia* [109].

References

1. Charlesworth D, Charlesworth B, Marais G. Steps in the evolution of heteromorphic sex chromosomes. *Heredity*. 2005;95(2):118-128. doi:10.1038/sj.hdy.6800697.

2. Charlesworth B. The evolution of sex chromosomes. *Science*. 1991;251(4997):1030-1033. doi:10.1126/science.1998119.

3. Charlesworth D. Plant sex determination and sex chromosomes. *Heredity*. 2002;88(2):94-101. doi:10.1038/sj.hdy.6800016.

4. Aryal R, Ming R. Sex determination in flowering plants: papaya as a model system. *Plant Sci.* 2014;217-218:56-62. doi:10.1016/j.plantsci.2013.10.018.

5. Ming R, Bendahmane A, Renner SS. Sex chromosomes in land plants. *Annu Rev Plant Biol.* 2011;62:485-514. doi:10.1146/annurev-arplant-042110-103914.

6. Han W, Liu L, Wang J, et al. Ancient homomorphy of molluscan sex chromosomes sustained by reversible sex-biased genes and sex determiner translocation. *Nat Ecol Evol.* 2022;6(12):1891-1906. doi:10.1038/s41559-022-01898-6.

7. Wright AE, Dean R, Zimmer F, Mank JE. How to make a sex chromosome. *Nat Commun.* 2016;7:12087. doi:10.1038/ncomms12087.

8. Westergaard M. The mechanism of sex determination in dioecious flowering plants. *Adv Genet.* 1958;9:217-281. doi:10.1016/s0065-2660(08)60163-7.

9. Renner SS. The relative and absolute frequencies of angiosperm sexual systems: dioecy, monoecy, gynodioecy, and an updated online database. *Am J Bot*. 2014;101(10):1588-1596. doi:10.3732/ajb.1400196.

10. Yu Q, Barrett SCH, Wang X-J, et al. Sexual dimorphism, temporal niche differentiation, and evidence for the Jack Sprat effect in an annual dioecious plant. *Journal of Systematics*

and Evolution. 2022;60:1078-1091. doi: 10.1111/jse.12753.

11. Onyekwelu SS, Harper JL. Sex ratio and niche differentiation in spinach (*Spinacia oleracea* L.). *Nature*. 1979;282:609-611. doi: 10.1038/282609a0.
12. Sánchez-Vilas J, Pannell JR. Differential niche modification by males and females of a dioecious herb: extending the Jack Sprat effect. *J Evol Biol.* 2010;23(10):2262-2266. doi:10.1111/j.1420-9101.2010.02089.x.
13. Ohta K, Shibata T, Nicolas A. Changes in chromatin structure at recombination initiation sites during yeast meiosis. *EMBO J.* 1994;13(23):5754-5763. doi:10.1002/j.1460-2075.1994.tb06913.x.
14. Rifkin JL, Beaudry FEG, Humphries Z, et al. Widespread Recombination Suppression Facilitates Plant Sex Chromosome Evolution. *Mol Biol Evol.* 2021;38(3):1018-1030. doi:10.1093/molbev/msaa271
15. Ellegren H, Parsch J. The evolution of sex-biased genes and sex-biased gene expression. *Nat Rev Genet*. 2007;8(9):689-698. doi:10.1038/nrg2167.
16. Berner D, Roesti M. Genomics of adaptive divergence with chromosome-scale heterogeneity in crossover rate. *Mol Ecol*. 2017;26(22):6351-6369. doi:10.1111/mec.14373.
17. Akagi T, Henry IM, Tao R, Comai L. Plant genetics. A Y-chromosome-encoded small RNA acts as a sex determinant in persimmons. *Science.* 2014;346(6209):646-650. doi:10.1126/science.1257225.
18. Akagi T, Henry IM, Ohtani H, et al. A Y-Encoded Suppressor of Feminization Arose via Lineage-Specific Duplication of a Cytokinin Response Regulator in Kiwifruit. *Plant Cell.*

2018;30(4):780-795. doi:10.1105/tpc.17.00787.

19. Akagi T, Pilkington SM, Varkonyi-Gasic E, et al. Two Y-chromosome-encoded genes determine sex in kiwifruit. *Nat Plants.* 2019;5(8):801-809. doi:10.1038/s41477-019-0489-6.

20. Yu W, Du Y, Li S, et al.. Sea buckthorn-nutritional composition, bioactivity, safety, and applications: A review. *Journal of Food Composition and Analysis*. 2024; doi: 10.1016/j.jfca.2024.106371.

21. Ling N, Tian H, Wang Q, et al. Advance in *Hippophae rhamnoides* polysaccharides: Extraction, structural characteristics, pharmacological activity, structure-activity relationship and application. *Int J Biol Macromol.* 2024;270(Pt 2):132420. doi:10.1016/j.ijbiomac.2024.132420.

22. Dolkar P, Dolkar D, Kant A, Chaurasia OP, Stobdan T. Gender differences in phenotypic plasticity and adaptive response of Seabuckthorn (*Hippophae rhamnoides* L.) along an altitudinal gradient in trans-Himalaya. *JBR*. 2018, 9 (1): 1-10. doi: 10.3233/JBR-180294.

23. Puterova J, Razumova O, Martinek T, et al. Satellite DNA and Transposable Elements in Seabuckthorn *(Hippophae rhamnoides*), a Dioecious Plant with Small Y and Large X Chromosomes. *Genome Biol Evol*. 2017;9(1):197-212. doi:10.1093/gbe/evw303.

24. Yu L, Diao S, Zhang G, et al. Genome sequence and population genomics provide insights into chromosomal evolution and phytochemical innovation of *Hippophae rhamnoides.* Plant Biotechnol J. 2022;20(7):1257-1273. doi:10.1111/pbi.13802.

25. Fu X, Wu J, Ma X, Li K, Zhang H, Wu S, et al. The Chromosome-Level Genome of *Elaeagnus moorcroftii* Wall., an Economically and Ecologically Important Tree Species

in Drylands. Diversity. 2022, 14 (6): 468-468. doi: 10.3390/d14060468.

26. Wang R, Wu B, Jian J, et al. How to survive in the world's third poplar: Insights from the genome of the highest altitude woody plant, *Hippophae tibetana* (Elaeagnaceae). *Front Plant Sci.* 2022;13:1051587. doi:10.3389/fpls.2022.1051587.

27. Zhang G, Song Y, Chen N, et al. Chromosome-level genome assembly of *Hippophae tibetana* provides insights into high-altitude adaptation and flavonoid biosynthesis. *BMC Biol.* 2024; 22: 82. https://doi.org/10.1186/s12915-024-01875-4

28. Wu Z, Chen H, Pan Y, et al. Genome of *Hippophae rhamnoides* provides insights into a conserved molecular mechanism in actinorhizal and rhizobial symbioses. *New Phytol.* 2022;235(1):276-291. doi:10.1111/nph.18017.

29. Chen M, Yang D, Yang S, et al. Chromosome-level genome assembly of *Hippophae gyantsensis*. Sci Data. 2024;11(1):126. doi:10.1038/s41597-024-02909-w.

30. Wu J, Zhang L, Ma X, et al. The evolutionary significance of whole genome duplications in oil biosynthesis of oil crops. *Hortic Res*. 2024;11(7):uhae156. doi:10.1093/hr/uhae156.

31. ojas MG, Pozzi E, Ramasamy R. Unlocking the mystery of the human Y chromosome. *Nat Rev Urol*. 2024;21(2):65-66. doi:10.1038/s41585-023-00826-y.

32. Bergero R, Charlesworth D. The evolution of restricted recombination in sex chromosomes. *Trends Ecol Evol.* 2009;24(2):94-102. doi:10.1016/j.tree.2008.09.010.

33. Doan TT, Carlsson AS, Stymne S, Hofvander P. Biochemical characteristics of *AtFAR2*, a fatty acid reductase from *Arabidopsis thaliana* that reduces fatty acyl-CoA and -ACP substrates into fatty alcohols. Acta Biochim Pol. 2016;63(3):565-570. doi:10.18388/abp.2016_1245.

34. Abe M, Kobayashi Y, Yamamoto S, et al. *FD*, a bZIP protein mediating signals from the floral pathway integrator *FT* at the shoot apex. *Science.* 2005;309(5737):1052-1056. doi:10.1126/science.1115983.

35. Kitagawa M, Wu P, Balkunde R, Cunniff P, Jackson D. An RNA exosome subunit mediates cell-to-cell trafficking of a homeobox mRNA via plasmodesmata. *Science*. 2022;375(6577):177-182. doi:10.1126/science.abm0840.

36. Huang Z, De O Furo I, Liu J, et al. Recurrent chromosome reshuffling and the evolution of neo-sex chromosomes in parrots. *Nat Commun.* 2022;13(1):944. doi:10.1038/s41467-022-28585-1.

37. Damas J, Corbo M, Lewin HA. Vertebrate Chromosome Evolution. *Annu Rev Anim Biosci*. 2021;9:1-27. doi:10.1146/annurev-animal-020518-114924.

38. Haenel Q, Laurentino TG, Roesti M, Berner D. Meta-analysis of chromosome-scale crossover rate variation in eukaryotes and its significance to evolutionary genomics. *Mol Ecol.* 2018;27(11):2477-2497. doi:10.1111/mec.14699.

39. Zhou R, Macaya-Sanz D, Rodgers-Melnick E, et al. Characterization of a large sex determination region in *Salix purpurea* L. (Salicaceae). *Mol Genet Genomics*. 2018;293(6):1437-1452. doi:10.1007/s00438-018-1473-y.

40. Wu M, Moore RC. The Evolutionary Tempo of Sex Chromosome Degradation in *Carica papaya*. *J Mol Evol*. 2015;80(5-6):265-277. doi:10.1007/s00239-015-9680-1.

41. Pilkington SM, Tahir J, Hilario E, et al. Genetic and cytological analyses reveal the recombination landscape of a partially differentiated plant sex chromosome in kiwifruit. *BMC Plant Biol*. 2019;19(1):172. doi:10.1186/s12870-019-1766-2.

42. Mariotti B, Manzano S, Kejnovský E, Vyskot B, Jamilena M. Accumulation of Y-specific satellite DNAs during the evolution of *Rumex acetosa* sex chromosomes. *Mol Genet Genomics*. 2009;281(3):249-259. doi:10.1007/s00438-008-0405-7.

43. Lahn BT, Page DC. Four evolutionary strata on the human X chromosome. *Science*. 1999;286(5441):964-967. doi:10.1126/science.286.5441.964.

44. Natri HM, Merilä J, Shikano T. The evolution of sex determination associated with a chromosomal inversion. *Nat Commun*. 2019;10(1):145. doi:10.1038/s41467-018-08014-y.

45. Yin T, Difazio SP, Gunter LE, et al. Genome structure and emerging evidence of an incipient sex chromosome in Populus. *Genome Res.* 2008;18(3):422-430. doi:10.1101/gr.7076308.

46. Wang J, Na JK, Yu Q, et al. Sequencing papaya X and Yh chromosomes reveals molecular basis of incipient sex chromosome evolution. *Proc Natl Acad Sci*. 2012;109(34):13710-13715. doi:10.1073/pnas.1207833109.

47. Yue J, Krasovec M, Kazama Y, et al. The origin and evolution of sex chromosomes, revealed by sequencing of the *Silene latifolia* female genome. *Curr Biol*. 2023;33(12):2504-2514.e3. doi:10.1016/j.cub.2023.05.046.

48. Xue L, Wu H, Chen Y, et al. Evidences for a role of two Y-specific genes in sex determination in *Populus deltoides*. *Nat Commun*. 2020;11(1):5893. doi:10.1038/s41467-020-19559-2.

49. Harkess A, Huang K, van der Hulst R, et al. Sex Determination by Two Y-Linked Genes in Garden Asparagus. *Plant Cell.* 2020;32(6):1790-1796. doi:10.1105/tpc.19.00859.

50. Müller NA, Kersten B, Leite Montalvão AP, et al. A single gene underlies the dynamic evolution of poplar sex determination. *Nat Plants.* 2020;6(6):630-637. doi:10.1038/s41477-020-0672-9.

51. Pahlich E, Gerlitz C. A rapid DNA isolation procedure for small quantities of fresh leaf tissue. *Phytochemistry*. 1980;19:11-13 doi: 10.1016/0031-9422(80)85004-7.

52. Marçais G, Kingsford C. A fast, lock-free approach for efficient parallel counting of occurrences of k-mers. *Bioinformatics*. 2011;27(6):764-770. doi:10.1093/bioinformatics/btr011.

53. Vurture GW, Sedlazeck FJ, Nattestad M, et al. GenomeScope: fast reference-free genome profiling from short reads. *Bioinformatics.* 2017;33(14):2202-2204. doi:10.1093/bioinformatics/btx153.

54. Hu J, Fan J, Sun Z, Liu S. NextPolish: a fast and efficient genome polishing tool for long-read assembly. *Bioinformatics*. 2020;36(7):2253-2255. doi:10.1093/bioinformatics/btz891.

55. Danecek P, Bonfield JK, Liddle J, et al. Twelve years of SAMtools and BCFtools. *Gigascience*. 2021;10(2):giab008. doi:10.1093/gigascience/giab008.

56. Cheng H, Jarvis ED, Fedrigo O, et al. Haplotype-resolved assembly of diploid genomes without parental data. *Nat Biotechnol*. 2022;40(9):1332-1335. doi:10.1038/s41587-022-01261-x.

57. Li H. Minimap2: pairwise alignment for nucleotide sequences. *Bioinformatics*. 2018;34(18):3094-3100. doi:10.1093/bioinformatics/bty191

58. Guan D, McCarthy SA, Wood J, et al. Identifying and removing haplotypic duplication in

primary genome assemblies. *Bioinformatics*. 2020;36(9):2896-2898. doi:10.1093/bioinformatics/btaa025.

59. Durand NC, Shamim MS, Machol I, et al. Juicer Provides a One-Click System for Analyzing Loop-Resolution Hi-C Experiments. *Cell Syst*. 2016;3(1):95-98. doi:10.1016/j.cels.2016.07.002

60. Zhang X, Zhang S, Zhao Q, Ming R, Tang H. Assembly of allele-aware, chromosomal-scale autopolyploid genomes based on Hi-C data. *Nat Plants*. 2019;5(8):833-845. doi:10.1038/s41477-019-0487-8.

61. Durand NC, Robinson JT, Shamim MS, et al. Juicebox Provides a Visualization System for Hi-C Contact Maps with Unlimited Zoom. *Cell Syst*. 2016;3(1):99-101. doi:10.1016/j.cels.2015.07.012.

62. Ramírez F, Bhardwaj V, Arrigoni L, et al. High-resolution TADs reveal DNA sequences underlying genome organization in flies. *Nat Commun.* 2018;9(1):189. doi:10.1038/s41467-017-02525-w.

63. Hu J, Wang Z, Liang F, et al. NextPolish2: A Repeat-aware Polishing Tool for Genomes Assembled Using HiFi Long Reads. *Genomics Proteomics Bioinformatics*. 2024;22(1):qzad009. doi:10.1093/gpbjnl/qzad009.

64. Manni M, Berkeley MR, Seppey M, Simão FA, Zdobnov EM. BUSCO Update: Novel and Streamlined Workflows along with Broader and Deeper Phylogenetic Coverage for Scoring of Eukaryotic, Prokaryotic, and Viral Genomes. *Mol Biol Evol*. 2021;38(10):4647-4654. doi:10.1093/molbev/msab199.

65. Ou S, Su W, Liao Y, et al. Benchmarking transposable element annotation methods for

creation of a streamlined, comprehensive pipeline. *Genome Biol*. 2019;20(1):275. doi:10.1186/s13059-019-1905-y.

66. Zhang RG, Li GY, Wang XL, et al. TEsorter: an accurate and fast method to classify LTR-retrotransposons in plant genomes. *Hortic Res*. 2022; doi:10.1093/hr/uhac017.

67. Yan H, Bombarely A, Li S. DeepTE: a computational method for de novo classification of transposons with convolutional neural network. *Bioinformatics*. 2020;36(15):4269-4275. doi:10.1093/bioinformatics/btaa519.

68. Chen N. Using RepeatMasker to identify repetitive elements in genomic sequences. *Curr Protoc Bioinformatics*. 2004. doi:10.1002/0471250953.bi0410s05.

69. Kim D, Langmead B, Salzberg SL. HISAT: a fast spliced aligner with low memory requirements. *Nat Methods*. 2015;12(4):357-360. doi:10.1038/nmeth.3317.

70. Stanke M, Diekhans M, Baertsch R, Haussler D. Using native and syntenically mapped cDNA alignments to improve de novo gene finding. *Bioinformatics*. 2008;24(5):637-644. doi:10.1093/bioinformatics/btn013.

71. Stanke M, Schöffmann O, Morgenstern B, Waack S. Gene prediction in eukaryotes with a generalized hidden Markov model that uses hints from external sources. *BMC Bioinformatics*. 2006;7:62. doi:10.1186/1471-2105-7-62.

72. Brůna T, Lomsadze A, Borodovsky M. GeneMark-EP+: eukaryotic gene prediction with self-training in the space of genes and proteins. *NAR Genom Bioinform*. 2020;2(2):lqaa026. doi:10.1093/nargab/lqaa026.

73. Gabriel L, Brůna T, Hoff KJ, et al. BRAKER3: Fully automated genome annotation using RNA-seq and protein evidence with GeneMark-ETP, AUGUSTUS and TSEBRA.

Preprint. bioRxiv. 2024;2023.06.10.544449. doi:10.1101/2023.06.10.544449.

74. Gabriel L, Hoff KJ, Brůna T, Borodovsky M, Stanke M. TSEBRA: transcript selector for BRAKER. *BMC Bioinformatics*. 2021;22(1):566. doi:10.1186/s12859-021-04482-0.

75. Campbell MS, Holt C, Moore B, Yandell M. Genome Annotation and Curation Using MAKER and MAKER-P. *Curr Protoc Bioinformatics*. 2014;48:4.11.1-4.11.39. doi:10.1002/0471250953.bi0411s48.

76. Chen C, Chen H, Zhang Y, et al. TBtools: An Integrative Toolkit Developed for Interactive Analyses of Big Biological Data. *Mol Plant*. 2020;13(8):1194-1202. doi:10.1016/j.molp.2020.06.009.

77. Pertea G, Pertea M. GFF Utilities: GffRead and GffCompare. F1000Res. 2020;9:ISCB Comm J-304. doi:10.12688/f1000research.23297.2.

78. Shen W, Le S, Li Y, Hu F. SeqKit: A Cross-Platform and Ultrafast Toolkit for FASTA/Q File Manipulation. *PLoS One*. 2016;11(10):e0163962. doi:10.1371/journal.pone.0163962.

79. Jones P, Binns D, Chang HY, et al. InterProScan 5: genome-scale protein function classification. *Bioinformatics*. 2014;30(9):1236-1240. doi:10.1093/bioinformatics/btu031.

80. Boeckmann B, Bairoch A, Apweiler R, et al. The SWISS-PROT protein knowledgebase and its supplement TrEMBL in 2003. *Nucleic Acids Res*. 2003;31(1):365-370. doi:10.1093/nar/gkg095.

81. Buchfink B, Reuter K, Drost HG. Sensitive protein alignments at tree-of-life scale using DIAMOND. *Nat Methods*. 2021;18(4):366-368. doi:10.1038/s41592-021-01101-x.

82. Cantalapiedra CP, Hernández-Plaza A, Letunic I, et al. eggNOG-mapper v2: Functional Annotation, Orthology Assignments, and Domain Prediction at the Metagenomic Scale. *Mol Biol Evol*. 2021;38(12):5825-5829. doi:10.1093/molbev/msab293.

83. Ogata H, Goto S, Sato K, Fujibuchi W, Bono H, Kanehisa M. KEGG: Kyoto Encyclopedia of Genes and Genomes. *Nucleic Acids Res*. 1999;27(1):29-34. doi:10.1093/nar/27.1.29.

84. Gene Ontology Consortium, Aleksander SA, Balhoff J, et al. The Gene Ontology knowledgebase in 2023. *Genetics*. 2023;224(1):iyad031. doi:10.1093/genetics/iyad031.

85. Emms DM, Kelly S. OrthoFinder: solving fundamental biases in whole genome comparisons dramatically improves orthogroup inference accuracy. *Genome Biol*. 2015;16(1):157. doi:10.1186/s13059-015-0721-2.

86. Edgar RC. MUSCLE: multiple sequence alignment with high accuracy and high throughput. *Nucleic Acids Res*. 2004;32(5):1792-1797. doi:10.1093/nar/gkh340.

87. Capella-Gutiérrez S, Silla-Martínez JM, Gabaldón T. trimAl: a tool for automated alignment trimming in large-scale phylogenetic analyses. *Bioinformatics.* 2009;25(15):1972-1973. doi:10.1093/bioinformatics/btp348.

88. Stamatakis A, Hoover P, Rougemont J. A rapid bootstrap algorithm for the RAxML Web servers. *Syst Biol*. 2008;57(5):758-771. doi:10.1080/10635150802429642.

89. Kumar S, Stecher G, Suleski M, Hedges SB. TimeTree: A Resource for Timelines, Timetrees, and Divergence Times. *Mol Biol Evol*. 2017;34(7):1812-1819. doi:10.1093/molbev/msx116.

90. Yang Z. PAML 4: phylogenetic analysis by maximum likelihood. *Mol Biol Evol*. 2007;24(8):1586-1591. doi:10.1093/molbev/msm088.

91. Mendes FK, Vanderpool D, Fulton B, Hahn MW. CAFE 5 models variation in evolutionary rates among gene families. *Bioinformatics*. 2021;36(22-23):5516-5518. doi:10.1093/bioinformatics/btaa1022.

92. Sun P, Jiao B, Yang Y, et al. WGDI: A user-friendly toolkit for evolutionary analyses of whole-genome duplications and ancestral karyotypes. *Mol Plant*. 2022;15(12):1841-1851. doi:10.1016/j.molp.2022.10.018.

93. Tang H, Krishnakumar V, Zeng X, et al. JCVI: A versatile toolkit for comparative genomics analysis. *Imeta*. 2024;3(4):e211. doi:10.1002/imt2.211.

94. Li H, Durbin R. Fast and accurate long-read alignment with Burrows-Wheeler transform. *Bioinformatics*. 2010;26(5):589–95. 10.1093/bioinformatics/btp698.

95. Quinlan AR, Hall IM. BEDTools: a flexible suite of utilities for comparing genomic features. *Bioinformatics*. 2010;26(6):841-842. doi:10.1093/bioinformatics/btq033.

96. Ginestet C. ggplot2: Elegant Graphics for Data Analysis. *Journal of the Royal Statistical Society Series A: Statistics in Society*. 2011; doi: 10.1111/j.1467-985X.2010.00676_9.x.

97. Robinson JT, Thorvaldsdottir H, Turner D, Mesirov JP. igv.js: an embeddable JavaScript implementation of the Integrative Genomics Viewer (IGV). *Bioinformatics*. 2023;39(1):btac830. doi:10.1093/bioinformatics/btac830.

98. Camacho C, Coulouris G, Avagyan V, et al. BLAST+: architecture and applications. *BMC Bioinformatics*. 2009;10:421. doi:10.1186/1471-2105-10-421.

99. Zhang Z, Xiao J, Wu J, et al. ParaAT: a parallel tool for constructing multiple protein-coding DNA alignments. *Biochem Biophys Res Commun*. 2012;419(4):779-781. doi:10.1016/j.bbrc.2012.02.101.

100. Wang D, Zhang Y, Zhang Z, Zhu J, Yu J. KaKs_Calculator 2.0: a toolkit incorporating gamma-series methods and sliding window strategies. *Genomics Proteomics Bioinformatics*. 2010;8(1):77-80. doi:10.1016/S1672-0229(10)60008-3.

101. Nakamura T, Yamada KD, Tomii K, Katoh K. Parallelization of MAFFT for large-scale multiple sequence alignments. *Bioinformatics*. 2018;34(14):2490-2492. doi:10.1093/bioinformatics/bty121.

102. Minh BQ, Schmidt HA, Chernomor O, et al. IQ-TREE 2: New Models and Efficient Methods for Phylogenetic Inference in the Genomic Era. *Mol Biol Evol*. 2020;37(5):1530-1534. doi:10.1093/molbev/msaa015.

103. Chen S, Zhou Y, Chen Y, Gu J. fastp: an ultra-fast all-in-one FASTQ preprocessor. *Bioinformatics*. 2018;34(17):i884-i890. doi:10.1093/bioinformatics/bty560.

104. Dobin A, Davis CA, Schlesinger F, et al. STAR: ultrafast universal RNA-seq aligner. *Bioinformatics*. 2013;29(1):15-21. doi:10.1093/bioinformatics/bts635.

105. Li B, Dewey CN. RSEM: accurate transcript quantification from RNA-Seq data with or without a reference genome. *BMC Bioinformatics*. 2011;12:323. doi:10.1186/1471-2105-12-323.

106. Chen M. The latest genomic data of three sea buckthorn species. Figshare Database. 2025. https://doi.org/10.6084/m9.figshare.27093856.v1

107. Chen M, Yang X, Xun L, et al. Supporting data for "The genome of Hippophae salicifolia provides new insights into the sexual differentiation of seabuckthorn" GigaScience Database. 2025. https://doi.org/10.5524/102682

108. Chen M, Yang X, Xun L, et al. The genomic data of Hippophae gyantsensis (male) GigaScience Database. 2025. https://doi.org/10.5524/102684
109. Chen M, Yang X, Xun L, et al. Genomic data of Hippophae salicifolia (one female and two male haplotypes) GigaScience Database. 2025. https://doi.org/10.5524/102683

Tables and Figures

Figure 1. Genomic features of male *H. salicifolia*. (a) Pseudochromosome. (b) Gene density. (c) Repeat sequences density. (d) Ty3 density. (e) Copia density. (f) GC content. (g) Interspecies collinearity. Feature density and GC percentage were calculated with a 5Mb window size. *H. salicifolia* is diploid, so the haploid genome was used as the reference genome.

Figure 2. Comparative genomics analysis. (a) The divergence time tree of 13 species. The number of expanded gene families (red) and the number of contracted gene families (blue) are indicated to the right of each species branch. (b) Synonymous substitution rate per site (Ks) distribution for *H. salicifolia*, *H. gyantsensis*, *H. rhamnoides*, *H. tibetana*, *E. moorcroftii* and *Z.*

jujuba. Two recent whole genome duplication(WGD) events occurred in both *Hippophae* and *Elaeagnus*. (c) Macro-synteny plot of *H. salicifolia*, *E. mollis* and *Z. jujuba*. Syntenic comparison between *H. salicifolia* and *Z. jujuba* or between *E. mollis* and *Z. jujuba* revealed a 4:1 ratio that suggests two lineage-specific WGDs in Elaeagnaceae.

Figure 3. Identification of the *H. salicifolia* 2X- and 2Y-SLRs. (a) The resequencing data of male and female *H. salicifolia* were visualized in the coverage depth of Chr02 of Hap2. The red line represents the depth of female coverage. The blue line represents the depth of male coverage. Y indicates the position of the Y-SLR. (b) Genes distribution in 2Y-SLR. (c) The Resequencing data of male and female *H. salicifolia* were visualized in the coverage depth of Chr02 of Hap1. The red line represents the depth of female coverage. The blue line represents the depth of male coverage. X indicates the position of the X-SLR. (d) Genes distribution in 2X-SLR.

Figure 4. Sex chromosome evolution of *H. salicifolia.* (A) The local syntenic blocks identified between the Hap2 Chr02 and the genomes of *H. rhamnoides* and *E. mollis*. (B) The order and direction of Y-SLR in homologous blocks in the orthologous region of *Hippophae* and *Elaeagnus*. '+' indicates that the internal gene order is consistent, '-' indicates that the internal gene order is opposite, and the absence of a mark suggests that the direction of the internal genes is variable. (C) The distribution of Ks values across different strata, with * indicating the mean value. (D) Phylogenetic structure statistics of single-copy genes within different strata. Structure 1 represents the "ancestral" pattern, Structure 2 represents the "recent"

pattern, and Structure 3 represents the "chaotic" pattern.

Figure 5. Within the Y-SLR of *H. salicifolia*, there are male-specific expression genes in both *H. salicifolia* and *H. gyantsensis*. 'F' represents female, and 'M' represents male.

 Click here to access/download;Table;Table2.xlsx

Table S1 Sequencing statistics of male *H.gyantsensis*, female *H.salicifolia* and male *H*

Species	Source	Platform
H.gyantsensis_M	Genomic DNA	DNBSEQ-T7RS
		OXFORD_NANOPORE
	Transcriptome	DNBSEQ-T7RS
H.salicifolia_F	Genomic DNA	DNBSEQ-T7RS
		OXFORD_NANOPORE
	Transcriptome	DNBSEQ-T7RS
H.salicifolia_M	Genomic DNA	DNBSEQ-T7RS
		PacBio-HiFi
	Transcriptome	DNBSEQ-T7RS

.salicifolia.

Total Clean Data (Gb)
51.95
99.03
67.46
62.31
112.31
94.41
57.60
142.55
101.37

Table S2 Statistics of Hi-C reads mapping for male *H. gyantsensi*

	H. gyantsensis_M		*H. salicifolia* _F	
	Reads 1	Reads 2	Reads 1	Reads 2
Total Reads	453,837,544	453,837,544	276,021,303	276,021,303
Mapped Reads	358,818,554	338,266,081	244,500,698	243,967,083
Mapping Ratio (%)	89.03%	83.93%	88.58%	88.39%
Valid Pairs	124,518,314		89,237,887	
Percentage	27.44%		32.33%	
Total bases (Gb)	136.13		82.52	

Note: The statistical data are extracted from Hi-C pro result files: *.mpairstat, *.mRSstat, and * allValid

s , female *H. salicifolia* and male *H. salicifolia* .

H. salicifolia _M Hap1		*H. salicifolia* _M Hap2	
Reads 1	Reads 2	Reads 1	Reads 2
265,897,415	265,897,415	265,897,415	265,897,415
249,081,740	248,728,007	249,192,292	248,888,959
93.68%	93.54%	93.72%	93.60%
87,384,820		85,855,404	
32.86%		32.29%	
79.47		79.47	

Pairs.mergestat. The term “valid interaction rmdup” denotes non-redundant and valid Hi-C read pairs, which were emplo

[illegible]yed by EndHiC for the scaffolding process.

Table S3 Summary of male and female seabuckthorn genome assemblies

	*H. gyantsensis*_ M	*H. salicifolia* _F	*H. salicifolia* _M Hap1
Length of genome (bp)	704,347,864	788,279,297	1,139,980,221
Number of contigs	116	192	282
Contigs N50 (bp)	18,114,469	30,832,038	53,609,585
Number of scaffold	27	84	269
Scaffold N50 (bp)	63,851,542	72,668,590	98,061,035
GC content is (%)	29.70%	30.10%	30.07%

H. salicifolia _M Hap2
1,097,339,724
94
64,697,811
75
98,628,608
29.82%

Table S4 Quality assessment of the completeness of female and male geno

Statistical level	*H. gyantsensis* _M	*H. salicifolia* _F
Complete BUSCOs (C)	1,571	1,576
Complete Single-Copy BUSCOs (S)	1,426	1,440
Complete Duplicated BUSCOs (D)	145	136
Fragmented BUSCOs (F)	30	25
Missing BUSCOs (M)	13	13
Total BUSCO groups searched	1,614	1,614
Total	97.4%[S:88.4%,D:9.0%]	97.6%[S:89.2%,D:8.4%]

me assemblies using the BUSCO tool

H. salicifolia _M Hap1	*H. salicifolia* _M Hap2
1576	1576
1,437	1,439
139	137
25	23
13	15
1,614	1,614
97.6%[S:89.0%,D:8.6%]	97.7%[S:89.2%,D:8.5%]

Supplementary Table S5 Repeat sequence annotat

		H. gyatsensis _M	*H. salicifolia* _F
DNA_transposon		1.45%	1.37%
LTR	Copia	14.68%	15.88%
	Ty3	13.13%	16.55%
	unknown	7.25%	5.25%
TIR		12.80%	15.57%
low_complexity		0.06%	0.07%
nonLTR		2.78%	3.14%
helitron		2.70%	2.24%
repeat_region		1.51%	1.33%
retrotransposon		0.08%	0.01%
total		56.42%	60.41%

ion statistics

H. salicifolia _M Hap1	*H. salicifolia* _M Hap2
3.53%	1.05%
13.74%	11.94%
18.01%	13.75%
5.21%	10.45%
25.63%	29.56%
0.01%	0.01%
1.50%	1.88%
2.37%	1.24%
0.91%	1.01%
0.00%	0.00%
70.88%	70.90%

Table S6 Functional annotation of predicted protein-coding genes of the male and female seal

Values	*Male H. gyantsensis*	*Female H. salicifolia*	*H. salicifolia* Hap1
Total	36482	39501	45937
Swissprot-Annotated	25317	26624	28236
KEGG-Annotated	15832	16188	17683
TrEMBL-Annotated	33070	34634	37987
nr	32312	33741	37157
Interpro-Annotated	26345	27016	29029
GO-Annotated	18095	18742	20210
Overall	33238	34811	38287

buckthorn genomes

H. salicifolia Hap2
39854
26443
16189
34147
33412
26465
18864
34460

Table S7 Quality assessment of the completeness of female and male gene sets with the BUS

	H. gyantsenisi _M	*H. salicifolia_F*	Hap1
Complete BUSCOs (C)	1596	1595	1596
Complete Single-Copy BUSCOs (S)	1,441	1450	1447
Complete Duplicated BUSCOs (D)	155	145	149
Fragmented BUSCOs (F)	7	9	9
Missing BUSCOs (M)	11	10	9
Total BUSCO groups searched	1614	1614	1614
	98.9%[S:89.3%,D:9.6%]	98.8%[S:89.8%,D:9.0%]	98.9%[S:89.7%,D:9.2%]

CO tool

Hap2
1592
1447
145
11
11
1614
98.7%[S:89.7%,D:9.0%]

Table S9 The classification of gene origins within the X- and Y-SLR

	Gene of X-SLR	Gene of Y-SLR
Total gene number	334	250
ancestral genes	101	100
acquired genes	120	53
duplicated genes	82	73
unique genes	31	24

Table S10 The collinearity relationship between the X-SLR of *H. sali*

block	Hap2	*E. moorcroftii*	*E. mollis*	Hap1
B	Hsam2h02g1200.1	.	.	Hsam1h02g1239.1
B	Hsam2h02g1201.1	Emoo08g1484.1	Emol03g1015.1	Hsam1h02g1240.1
B	Hsam2h02g1202.1	Emoo08g1483.1	Emol03g1016.1	Hsam1h02g1245.1
B	Hsam2h02g1203.1	.	Emol03g1017.1	Hsam1h02g1246.1
B	Hsam2h02g1204.2	Emoo08g1488.1	Emol03g1011.1	Hsam1h02g1247.3
B	Hsam2h02g1205.1	.	.	.
B	Hsam2h02g1206.2	Emoo08g1490.1	Emol03g1009.1	Hsam1h02g1249.3
B	Hsam2h02g1207.1	.	.	.
B	Hsam2h02g1208.1	.	.	.
B	Hsam2h02g1209.2	Emoo08g1491.1	Emol03g1007.1	.
B	Hsam2h02g1210.1	.	.	Hsam1h02g1258.1
B	Hsam2h02g1211.1	.	.	Hsam1h02g1268.1
B	Hsam2h02g1212.2	Emoo08g1492.1	Emol03g1006.1	Hsam1h02g1276.2
A1	Hsam2h02g1213.1	.	.	.
A1	Hsam2h02g1214.1	.	.	.
A1	Hsam2h02g1215.1	Emoo08g1010.1	Emol03g1436.1	Hsam1h02g1296.1
A1	Hsam2h02g1216.1	Emoo08g1014.1	Emol03g1432.1	Hsam1h02g1295.1
A1	Hsam2h02g1217.2	Emoo08g1015.1	Emol03g1430.1	Hsam1h02g1294.1
A1	Hsam2h02g1218.1	.	.	.
A1	Hsam2h02g1219.1	Emoo08g1016.1	Emol03g1429.1	Hsam1h02g1286.1
A1	Hsam2h02g1220.1	.	.	.
A1	Hsam2h02g1221.1	Emoo08g1017.1	Emol03g1428.1	Hsam1h02g1285.2
A1	Hsam2h02g1222.1	Emoo08g1023.1	Emol03g1420.1	Hsam1h02g1284.1
A1	Hsam2h02g1223.1	Emoo08g1024.1	Emol03g1419.1	Hsam1h02g1280.1
A2	Hsam2h02g1224.1	Emoo08g1030.1	Emol03g1413.1	Hsam1h02g1567.1
A2	Hsam2h02g1225.1	Emoo08g1040.1	Emol03g1402.1	.
A2	Hsam2h02g1226.1	Emoo08g1040.1	Emol03g1402.1	Hsam1h02g1562.1
A2	Hsam2h02g1227.2	Emoo08g1040.1	Emol03g1402.1	Hsam1h02g1562.1
A2	Hsam2h02g1228.1	Emoo08g1028.1	Emol03g1414.1	Hsam1h02g1558.2
A2	Hsam2h02g1229.1	.	.	.
A2	Hsam2h02g1230.1	.	.	.
A2	Hsam2h02g1231.1	.	.	.
A2	Hsam2h02g1232.1	Emoo08g1025.1	Emol03g1417.1	Hsam1h02g1555.1
A2	Hsam2h02g1233.1	.	.	.
A2	Hsam2h02g1234.1	Emoo08g1039.1	Emol03g1404.1	.
A2	Hsam2h02g1235.1	Emoo08g1039.1	Emol03g1404.1	.
A2	Hsam2h02g1236.1	Emoo08g1039.1	Emol03g1404.1	.
A2	Hsam2h02g1237.1	.	.	Hsam1h02g1569.1
C	Hsam2h02g1238.1	Emoo08g1859.1	Emol03g0658.1	Hsam1h02g1320.1
C	Hsam2h02g1239.1	Emoo08g1859.1	Emol03g0658.1	Hsam1h02g1321.1
C	Hsam2h02g1240.1	.	.	.
C	Hsam2h02g1241.1	.	.	Hsam1h02g1333.1
C	Hsam2h02g1242.1	.	.	.
C	Hsam2h02g1243.1	.	.	Hsam1h02g1331.1
C	Hsam2h02g1244.1	.	.	Hsam1h02g1331.1

C	Hsam2h02g1245.1	Emoo08g1860.1	Emol03g0657.1	Hsam1h02g1334.2
C	Hsam2h02g1246.1	.	.	Hsam1h02g1335.1
C	Hsam2h02g1247.2	Emoo08g1863.1	Emol03g0654.1	Hsam1h02g1336.1
C	Hsam2h02g1248.1	Emoo08g1864.1	Emol03g0653.1	Hsam1h02g1339.1
C	Hsam2h02g1249.1	.	.	Hsam1h02g1341.1
C	Hsam2h02g1250.2	Emoo08g1865.1	Emol03g0652.1	Hsam1h02g1342.2
C	Hsam2h02g1251.1	Emoo08g1866.1	Emol03g0651.1	Hsam1h02g1343.1
C	Hsam2h02g1252.1	Emoo08g1866.1	Emol03g0651.1	Hsam1h02g1343.1
C	Hsam2h02g1253.1	Emoo08g1866.1	Emol03g0651.1	Hsam1h02g1343.1
C	Hsam2h02g1254.1	Emoo08g1867.1	Emol03g0650.1	Hsam1h02g1345.2
C	Hsam2h02g1255.2	Emoo08g1869.1	Emol03g0648.1	Hsam1h02g1346.2
C	Hsam2h02g1256.1	.	.	Hsam1h02g1351.1
C	Hsam2h02g1257.1	.	.	.
C	Hsam2h02g1258.1	.	.	Hsam1h02g1355.1
C	Hsam2h02g1259.1	.	.	.
C	Hsam2h02g1260.1	.	.	.
C	Hsam2h02g1261.2	.	.	.
C	Hsam2h02g1262.1	.	.	.
C	Hsam2h02g1263.1	.	.	Hsam1h02g1359.1
C	Hsam2h02g1264.1	.	.	.
C	Hsam2h02g1265.1	.	.	.
C	Hsam2h02g1266.1	.	.	Hsam1h02g1360.1
C	Hsam2h02g1267.1	.	.	Hsam1h02g1362.1
C	Hsam2h02g1268.1	.	.	.
C	Hsam2h02g1269.2	.	.	Hsam1h02g1371.2
D	Hsam2h02g1298.1	.	.	Hsam1h02g1473.1
D	Hsam2h02g1299.1	.	.	.
D	Hsam2h02g1300.1	.	.	.
D	Hsam2h02g1301.1	.	.	.
D	Hsam2h02g1302.1	.	.	.
D	Hsam2h02g1303.1	.	.	.
D	Hsam2h02g1304.1	.	.	.
D	Hsam2h02g1305.2	Emoo12g1657.1	Emol07g1597.1	.
D	Hsam2h02g1306.1	Emoo12g1656.1	Emol07g1596.1	.
D	Hsam2h02g1307.1	.	.	.
D	Hsam2h02g1308.2	.	.	.
D	Hsam2h02g1309.1	.	.	.
D	Hsam2h02g1310.1	.	.	.
D	Hsam2h02g1311.1	.	.	Hsam1h02g1482.1
D	Hsam2h02g1312.1	.	.	Hsam1h02g1482.1
D	Hsam2h02g1313.1	.	.	.
D	Hsam2h02g1314.1	.	.	.
D	Hsam2h02g1315.1	.	.	.
D	Hsam2h02g1316.1	Emoo12g1646.1	Emol07g1586.1	Hsam1h02g1481.2
D	Hsam2h02g1317.2	Emoo12g1647.1	Emol07g1587.1	Hsam1h02g1484.1
D	Hsam2h02g1318.1	Emoo12g1647.1	Emol07g1587.1	Hsam1h02g1484.1
D	Hsam2h02g1319.1	.	.	Hsam1h02g1436.1

D	Hsam2h02g1320.1	.	.	.
D	Hsam2h02g1321.1	.	.	.
D	Hsam2h02g1322.1	.	.	.
D	Hsam2h02g1323.1	Emoo12g1655.1	Emol07g1594.1	Hsam1h02g1437.1
D	Hsam2h02g1324.1	.	.	Hsam1h02g1441.1
D	Hsam2h02g1325.1	.	.	.
D	Hsam2h02g1326.1	.	.	Hsam1h02g1449.1
D	Hsam2h02g1327.2	.	.	Hsam1h02g1450.2
D	Hsam2h02g1328.1	Emoo12g1651.1	Emol07g1590.1	Hsam1h02g1451.1
D	Hsam2h02g1329.1	Emoo12g1651.1	Emol07g1590.1	Hsam1h02g1451.1
D	Hsam2h02g1330.1	Emoo12g1651.1	Emol07g1590.1	Hsam1h02g1451.1
D	Hsam2h02g1331.2	.	.	Hsam1h02g1453.1
D	Hsam2h02g1332.1	Emoo12g1642.1	Emol07g1582.1	Hsam1h02g1478.1
D	Hsam2h02g1333.1	.	.	Hsam1h02g1476.1
D	Hsam2h02g1334.1	.	.	Hsam1h02g1475.1
D	Hsam2h02g1335.1	.	.	Hsam1h02g1474.1
D	Hsam2h02g1336.1	.	.	Hsam1h02g1474.1
D	Hsam2h02g1337.1	Emoo12g1634.1	Emol07g1574.1	Hsam1h02g1472.1
D	Hsam2h02g1338.1	.	.	.
D	Hsam2h02g1339.1	Emoo12g1633.1	Emol07g1572.1	.
D	Hsam2h02g1340.2	.	.	.
D	Hsam2h02g1341.1	Emoo12g1632.1	Emol07g1571.1	Hsam1h02g1470.1
D	Hsam2h02g1342.1	.	.	Hsam1h02g1468.1
D	Hsam2h02g1343.1	.	.	Hsam1h02g1468.1
D	Hsam2h02g1344.1	.	.	Hsam1h02g1467.1
D	Hsam2h02g1345.1	Emoo12g1629.1	Emol07g1566.1	Hsam1h02g1465.1
D	Hsam2h02g1346.1	.	.	.
D	Hsam2h02g1347.1	Emoo12g1628.1	Emol07g1565.1	Hsam1h02g1464.1
D	Hsam2h02g1348.1	.	.	.
D	Hsam2h02g1349.2	.	.	.
D	Hsam2h02g1350.2	.	.	Hsam1h02g1458.1
D	Hsam2h02g1351.1	Emoo12g1644.1	Emol07g1584.1	Hsam1h02g1459.1
D	Hsam2h02g1352.1	.	.	Hsam1h02g1462.1
D	Hsam2h02g1353.3	Emoo12g1645.1	Emol07g1585.1	Hsam1h02g1460.3
D	Hsam2h02g1354.1	.	.	Hsam1h02g1462.1
D	Hsam2h02g1355.3	Emoo12g1645.1	Emol07g1585.1	Hsam1h02g1460.3
D	Hsam2h02g1356.1	.	.	Hsam1h02g1462.1
D	Hsam2h02g1357.1	Emoo12g1627.1	Emol07g1564.1	Hsam1h02g1463.1
D	Hsam2h02g1358.1	.	.	.
D	Hsam2h02g1359.1	.	.	.
D	Hsam2h02g1360.1	.	.	Hsam1h02g1456.1
D	Hsam2h02g1361.1	.	.	Hsam1h02g1455.1
D	Hsam2h02g1362.1	.	Emol07g1549.1	Hsam1h02g1485.1
D	Hsam2h02g1363.1	.	Emol07g1545.1	.
E1	Hsam2h02g1364.1	Emoo12g1523.1	Emol07g1471.1	Hsam1h02g1383.1
E1	Hsam2h02g1365.1	.	.	.
E1	Hsam2h02g1366.1	Emoo12g1524.1	Emol07g1472.1	Hsam1h02g1385.1

E1	Hsam2h02g1367.1	Emoo12g1525.1	Emol07g1473.1	Hsam1h02g1386.1
E1	Hsam2h02g1368.1	.	.	Hsam1h02g1387.1
E1	Hsam2h02g1369.1	.	.	Hsam1h02g1388.1
E1	Hsam2h02g1370.1	.	.	Hsam1h02g1391.1
E1	Hsam2h02g1371.1	Emoo12g1528.1	Emol07g1476.1	Hsam1h02g1392.1
E1	Hsam2h02g1372.1	Emoo12g1529.1	Emol07g1477.1	Hsam1h02g1393.1
E1	Hsam2h02g1373.1	Emoo12g1532.1	Emol07g1480.1	Hsam1h02g1394.1
E1	Hsam2h02g1374.1	Emoo12g1532.1	Emol07g1480.1	Hsam1h02g1395.1
E1	Hsam2h02g1375.1	.	.	Hsam1h02g1396.1
E1	Hsam2h02g1376.1	.	.	Hsam1h02g1397.1
E1	Hsam2h02g1377.1	.	.	.
E1	Hsam2h02g1378.1	Emoo12g1533.1	Emol07g1481.1	Hsam1h02g1398.1
E1	Hsam2h02g1379.1	Emoo12g1552.1	Emol07g1496.1	Hsam1h02g1400.1
E1	Hsam2h02g1380.1	.	.	.
E1	Hsam2h02g1381.1	.	.	.
E1	Hsam2h02g1382.1	.	.	.
E1	Hsam2h02g1383.1	.	.	.
E1	Hsam2h02g1384.1	Emoo12g1545.1	Emol07g1490.1	Hsam1h02g1402.1
E1	Hsam2h02g1385.1	.	.	Hsam1h02g1407.1
E1	Hsam2h02g1386.1	.	.	Hsam1h02g1408.1
E1	Hsam2h02g1387.1	Emoo12g1550.1	Emol07g1494.1	Hsam1h02g1409.1
E1	Hsam2h02g1388.1	Emoo12g1549.1	Emol07g1493.1	Hsam1h02g1410.1
E1	Hsam2h02g1389.1	.	Emol07g1504.1	Hsam1h02g1411.1
E1	Hsam2h02g1390.1	.	.	.
E1	Hsam2h02g1391.3	Emoo12g1556.1	Emol07g1500.1	Hsam1h02g1412.1
E1	Hsam2h02g1392.1	Emoo12g1557.1	.	Hsam1h02g1415.1
E1	Hsam2h02g1393.1	.	.	Hsam1h02g1416.1
E1	Hsam2h02g1394.1	.	.	.
E1	Hsam2h02g1395.2	.	.	Hsam1h02g1417.1
E1	Hsam2h02g1396.1	Emoo12g1554.1	Emol07g1498.1	Hsam1h02g1418.1
E1	Hsam2h02g1397.1	Emoo12g1553.1	Emol07g1497.1	Hsam1h02g1423.1
E1	Hsam2h02g1398.1	Emoo12g1578.1	Emol07g1524.1	Hsam1h02g1425.1
E1	Hsam2h02g1399.3	.	.	Hsam1h02g1427.1
E1	Hsam2h02g1400.1	.	.	.
E1	Hsam2h02g1401.1	.	.	.
E1	Hsam2h02g1402.1	.	.	Hsam1h02g1435.1
E1	Hsam2h02g1403.1	Emoo12g1572.1	.	Hsam1h02g1432.1
E1	Hsam2h02g1404.1	Emoo12g1572.1	Emol07g1518.1	.
E1	Hsam2h02g1405.1	.	.	.
E1	Hsam2h02g1406.1	.	.	.
E1	Hsam2h02g1407.1	.	.	.
E1	Hsam2h02g1408.1	.	.	.
E1	Hsam2h02g1409.1	.	.	.
E1	Hsam2h02g1410.1	.	.	.
E2	Hsam2h02g1411.1	Emoo12g1603.1	.	Hsam1h02g1487.1
E2	Hsam2h02g1412.1	Emoo12g1588.1	.	Hsam1h02g1504.1
E2	Hsam2h02g1413.1	Emoo12g1588.1	.	Hsam1h02g1504.1

E2	Hsam2h02g1414.2	Emoo12g1563.1	Emol07g1510.1	.
E2	Hsam2h02g1415.1	.	.	.
E2	Hsam2h02g1416.1	Emoo12g1565.1	Emol07g1511.1	Hsam1h02g1503.1
E2	Hsam2h02g1417.1	Emoo12g1567.1	Emol07g1513.1	Hsam1h02g1502.1
E2	Hsam2h02g1418.1	Emoo12g1568.1	Emol07g1514.1	Hsam1h02g1501.1
E2	Hsam2h02g1419.1	Emoo12g1568.1	Emol07g1514.1	Hsam1h02g1501.1
E2	Hsam2h02g1420.1	Emoo12g1568.1	Emol07g1514.1	Hsam1h02g1500.1
E2	Hsam2h02g1421.1	Emoo12g1568.1	Emol07g1514.1	Hsam1h02g1501.1
E2	Hsam2h02g1422.1	Emoo12g1568.1	Emol07g1514.1	Hsam1h02g1501.1
E2	Hsam2h02g1423.1	Emoo12g1568.1	Emol07g1514.1	Hsam1h02g1500.1
E2	Hsam2h02g1424.1	Emoo12g1568.1	Emol07g1514.1	Hsam1h02g1500.1
E2	Hsam2h02g1425.1	Emoo12g1568.1	Emol07g1514.1	Hsam1h02g1501.1
E2	Hsam2h02g1426.1	Emoo12g1568.1	Emol07g1514.1	Hsam1h02g1501.1
E2	Hsam2h02g1427.1	Emoo12g1568.1	Emol07g1514.1	Hsam1h02g1500.1
E2	Hsam2h02g1428.1	.	.	.
E2	Hsam2h02g1429.1	.	.	.
E2	Hsam2h02g1430.1	.	.	.
F	Hsam2h02g1431.1	Emoo12g1522.1	Emol07g1469.1	Hsam1h02g1382.1
F	Hsam2h02g1432.1	.	.	Hsam1h02g1381.1
F	Hsam2h02g1433.1	Emoo12g1521.1	Emol07g1468.1	Hsam1h02g1380.1
F	Hsam2h02g1434.1	Emoo12g1520.1	Emol07g1467.1	Hsam1h02g1379.1
F	Hsam2h02g1435.1	.	.	Hsam1h02g1378.1
F	Hsam2h02g1436.1	Emoo12g1518.1	.	.
F	Hsam2h02g1437.1	Emoo12g1519.1	Emol07g1466.1	Hsam1h02g1377.1
G	Hsam2h02g1438.1	Emoo12g1514.1	Emol07g1462.1	Hsam1h02g1303.1
G	Hsam2h02g1439.1	Emoo12g1513.1	Emol07g1461.1	Hsam1h02g1307.1
G	Hsam2h02g1440.1	Emoo12g1511.1	Emol07g1460.1	Hsam1h02g1308.1
G	Hsam2h02g1441.1	.	.	.
G	Hsam2h02g1442.1	Emoo12g1508.1	Emol07g1457.1	Hsam1h02g1309.1
G	Hsam2h02g1443.1	.	.	Hsam1h02g1310.1
G	Hsam2h02g1444.1	.	.	Hsam1h02g1311.1
G	Hsam2h02g1445.2	Emoo12g1507.1	Emol07g1456.1	Hsam1h02g1312.1
G	Hsam2h02g1446.1	Emoo12g1505.1	Emol07g1454.1	Hsam1h02g1313.1
G	Hsam2h02g1447.1	Emoo12g1504.1	.	Hsam1h02g1314.1
G	Hsam2h02g1448.1	Emoo12g1504.1	.	Hsam1h02g1315.1

cifolia **and the homologous regions of closely related species**

**male *H. gyantsensis***	**female *H. gyantsensis***	***Fructus Hippophae***	***H.rhamnoides***
.	Hgyaf02g1135.1	Hipf02g1155.1	.
Hgyam02g1177.1	Hgyaf02g1136.1	Hipf02g1156.1	Hrha02g0997.1
Hgyam02g1179.1	Hgyaf02g1138.1	Hipf02g1158.1	Hrha02g0998.1
.	Hgyaf02g1139.2	.	.
Hgyam02g1180.1	Hgyaf02g1140.1	Hipf02g1159.2	Hrha02g1000.1
.	.	.	.
Hgyam02g1181.1	Hgyaf02g1141.1	Hipf02g1160.1	Hrha02g1002.1
.	.	.	.
.	.	.	.
.	Hgyaf02g1143.1	Hipf02g1161.3	Hrha02g1005.1
Hgyam02g1185.1	Hgyaf02g1148.1	Hipf02g1162.1	Hrha02g1006.1
Hgyam02g1185.1	Hgyaf02g1145.1	Hipf02g1162.1	Hrha02g1006.1
Hgyam02g1190.2	Hgyaf02g1149.2	Hipf02g1163.1	Hrha02g1007.1
Hgyam02g1191.1	.	Hipf02g1164.1	.
Hgyam02g1194.1	Hgyaf02g1153.1	Hipf02g1167.1	Hrha02g1011.1
Hgyam02g1195.2	Hgyaf02g1154.1	Hipf02g1166.1	Hrha02g1012.1
Hgyam02g1196.1	Hgyaf02g1155.1	Hipf02g1165.1	.
Hgyam02g1199.1	Hgyaf02g1158.1	Hipf02g1192.2	Hrha02g1013.1
.	.	.	.
Hgyam02g1201.1	Hgyaf02g1160.1	Hipf02g1193.1	Hrha02g1015.1
.	.	.	.
Hgyam02g1206.1	Hgyaf02g1161.1	Hipf02g1194.1	Hrha02g1016.1
Hgyam02g1207.3	Hgyaf02g1162.2	Hipf02g1195.2	Hrha02g1017.1
Hgyam02g1210.1	Hgyaf02g1163.1	.	.
Hgyam02g1260.1	Hgyaf02g1255.1	Hipf02g1208.1	Hrha02g1024.1
.	.	.	.
Hgyam02g1268.1	Hgyaf02g1247.1	.	.
Hgyam02g1268.1	Hgyaf02g1247.1	.	.
Hgyam02g1271.1	Hgyaf02g1244.2	Hipf02g1181.1	Hrha02g1062.1
.	.	.	.
.	.	.	.
.	.	.	.
Hgyam02g1275.1	Hgyaf02g1241.1	Hipf02g1177.1	Hrha02g1058.1
.	.	Hipf02g1178.1	.
.	.	.	Hrha02g1057.1
.	.	.	Hrha02g1057.1
.	.	.	Hrha02g1057.1
Hgyam02g1255.1	Hgyaf02g1259.1	Hipf02g1169.1	Hrha02g1056.1
Hgyam02g1247.1	Hgyaf02g1264.1	Hipf02g1222.1	Hrha02g1054.1
Hgyam02g1247.1	Hgyaf02g1264.1	Hipf02g1222.1	Hrha02g1054.1
.	.	.	.
.	.	.	.
.	.	.	.
.	.	.	.
.	.	.	.

Hgyam02g1242.2	Hgyaf02g1267.3	Hipf02g1224.2	Hrha02g1053.1
Hgyam02g1239.1	Hgyaf02g1269.1	Hipf02g1226.1	.
Hgyam02g1366.2	Hgyaf02g1270.1	Hipf02g1227.1	Hrha02g1048.1
Hgyam02g1367.1	Hgyaf02g1272.1	Hipf02g1228.1	Hrha02g1049.1
.	Hgyaf02g1273.1	.	.
Hgyam02g1368.1	Hgyaf02g1274.1	Hipf02g1229.1	.
Hgyam02g1369.1	Hgyaf02g1275.1	Hipf02g1230.1	Hrha02g1047.1
Hgyam02g1369.1	Hgyaf02g1275.1	Hipf02g1230.1	Hrha02g1047.1
Hgyam02g1369.1	Hgyaf02g1275.1	Hipf02g1230.1	Hrha02g1047.1
Hgyam02g1371.1	Hgyaf02g1277.1	Hipf02g1232.1	Hrha02g1046.1
Hgyam02g1381.1	Hgyaf02g1278.2	Hipf02g1234.2	Hrha02g1045.1
Hgyam02g1380.1	Hgyaf02g1279.1	Hipf02g1235.2	Hrha02g1044.1
Hgyam02g1378.1	.	Hipf02g1236.1	Hrha02g1042.1
Hgyam02g1376.1	Hgyaf02g1282.1	Hipf02g1238.2	Hrha02g1039.1
Hgyam02g1373.1	.	.	.
.	.	.	.
Hgyam02g1372.1	.	.	.
Hgyam02g1372.1	.	.	.
Hgyam02g1385.2	Hgyaf02g1286.1	Hipf02g1240.1	Hrha02g1038.1
Hgyam02g1387.1	Hgyaf02g1288.1	Hipf02g1241.1	.
.	.	.	.
Hgyam02g1391.1	Hgyaf02g1289.1	Hipf02g1245.1	Hrha02g1032.1
Hgyam02g1392.2	Hgyaf02g1290.1	Hipf02g1246.1	.
.	.	.	.
Hgyam02g1393.2	Hgyaf02g1292.1	Hipf02g1251.1	Hrha02g1029.1
.	Hgyaf02g1209.1	.	.
.	.	.	.
.	.	.	.
.	.	.	.
.	.	.	.
Hgyam02g1407.1	.	.	.
.	.	.	.
.	.	.	.
Hgyam02g1397.1	.	.	.
.	.	.	.
.	.	.	.
.	.	.	.
.	.	.	.
.	.	.	.
.	.	.	.
Hgyam02g1426.1	.	.	.
Hgyam02g1428.1	.	.	.
Hgyam02g1429.1	.	.	.
Hgyam02g1412.1	Hgyaf02g1218.2	Hipf02g1265.1	Hrha02g1078.1
Hgyam02g1414.1	Hgyaf02g1219.1	Hipf02g1264.1	Hrha02g1077.1
Hgyam02g1414.1	Hgyaf02g1219.1	Hipf02g1264.1	Hrha02g1077.1
.	.	.	.

.	.	.	.
.	.	.	.
.	.	.	.
Hgyam02g1441.1	Hgyaf02g1179.1	.	Hrha02g1073.1
Hgyam02g1439.1	Hgyaf02g1182.1	.	.
.	.	.	.
.	Hgyaf02g1188.1	Hipf02g1258.1	.
.	Hgyaf02g1189.2	Hipf02g1258.1	.
.	Hgyaf02g1191.1	Hipf02g1260.1	.
.	Hgyaf02g1191.1	Hipf02g1260.1	.
.	Hgyaf02g1191.1	Hipf02g1261.1	.
.	Hgyaf02g1193.1	Hipf02g1263.1	Hrha02g1076.1
Hgyam02g1411.2	Hgyaf02g1214.1	Hipf02g1273.1	Hrha02g1082.1
Hgyam02g1410.1	Hgyaf02g1213.1	Hipf02g1269.1	.
Hgyam02g1410.1	Hgyaf02g1212.1	Hipf02g1269.1	.
.	.	Hipf02g1271.1	.
.	.	Hipf02g1271.1	.
Hgyam02g1405.1	Hgyaf02g1208.1	Hipf02g1277.1	Hrha02g1083.1
.	.	.	.
Hgyam02g1404.1	.	Hipf02g1280.1	Hrha02g1085.1
Hgyam02g1403.1	.	.	.
Hgyam02g1402.1	Hgyaf02g1206.1	Hipf02g1281.1	Hrha02g1087.1
.	.	.	.
.	.	.	.
Hgyam02g1400.1	.	.	.
.	Hgyaf02g1205.1	Hipf02g1282.1	Hrha02g1091.1
.	.	.	.
Hgyam02g1460.1	Hgyaf02g1204.1	Hipf02g1283.1	Hrha02g1092.1
.	.	.	.
Hgyam02g1450.1	.	Hipf02g1364.1	Hrha02g1096.1
.	.	.	.
Hgyam02g1451.1	Hgyaf02g1198.1	Hipf02g1288.2	Hrha02g1095.1
Hgyam02g1456.1	Hgyaf02g1201.1	.	.
Hgyam02g1452.3	Hgyaf02g1199.3	.	.
Hgyam02g1456.1	Hgyaf02g1201.1	.	.
Hgyam02g1452.3	Hgyaf02g1199.3	.	.
Hgyam02g1456.1	Hgyaf02g1201.1	.	.
Hgyam02g1457.1	Hgyaf02g1202.1	Hipf02g1285.1	Hrha02g1094.1
.	.	.	.
.	.	.	.
Hgyam02g1445.1	Hgyaf02g1195.1	.	.
Hgyam02g1445.1	Hgyaf02g1195.1	.	.
Hgyam02g1444.1	Hgyaf02g1220.1	.	.
.	.	.	.
Hgyam02g1523.1	Hgyaf02g1338.1	Hipf02g1379.1	Hrha02g1148.1
.	Hgyaf02g1337.1	Hipf02g1380.1	Hrha02g1149.1
Hgyam02g1522.1	Hgyaf02g1336.1	Hipf02g1369.1	Hrha02g1144.1

Hgyam02g1521.1	Hgyaf02g1335.1	Hipf02g1368.1	Hrha02g1143.1
.	.	.	.
.	.	.	.
Hgyam02g1519.1	.	.	.
Hgyam02g1515.1	Hgyaf02g1333.2	Hipf02g1367.2	Hrha02g1142.1
Hgyam02g1514.1	Hgyaf02g1332.1	Hipf02g1366.1	Hrha02g1141.1
.	.	Hipf02g1359.1	Hrha02g1140.1
.	.	Hipf02g1359.1	Hrha02g1140.1
.	.	Hipf02g1360.2	Hrha02g1134.1
.	.	Hipf02g1360.2	.
.	.	.	.
Hgyam02g1511.1	Hgyaf02g1331.1	Hipf02g1361.1	Hrha02g1139.1
Hgyam02g1510.1	Hgyaf02g1330.1	Hipf02g1362.1	Hrha02g1138.1
Hgyam02g1509.1	Hgyaf02g1329.1	.	.
Hgyam02g1506.1	Hgyaf02g1328.1	.	.
Hgyam02g1505.1	Hgyaf02g1327.1	.	.
Hgyam02g1504.1	Hgyaf02g1327.1	.	.
Hgyam02g1502.1	Hgyaf02g1325.1	Hipf02g1352.1	.
Hgyam02g1498.1	Hgyaf02g1324.1	.	.
Hgyam02g1497.1	.	.	.
Hgyam02g1496.1	Hgyaf02g1323.1	Hipf02g1351.1	Hrha02g1136.1
Hgyam02g1495.1	Hgyaf02g1322.1	Hipf02g1350.1	Hrha02g1135.1
Hgyam02g1494.1	Hgyaf02g1321.1	.	Hrha02g1132.1
Hgyam02g1493.1	Hgyaf02g1320.1	.	.
Hgyam02g1492.2	Hgyaf02g1319.4	Hipf02g1365.2	.
.	.	Hipf02g1356.1	Hrha02g1126.1
Hgyam02g1490.1	Hgyaf02g1316.1	.	.
Hgyam02g1489.1	.	.	.
.	.	.	.
Hgyam02g1488.1	Hgyaf02g1315.1	Hipf02g1313.1	Hrha02g1124.1
Hgyam02g1487.1	Hgyaf02g1314.1	Hipf02g1312.1	Hrha02g1123.1
Hgyam02g1484.1	Hgyaf02g1311.1	Hipf02g1309.1	Hrha02g1118.1
Hgyam02g1481.2	Hgyaf02g1308.1	Hipf02g1307.1	.
.	.	.	.
.	.	.	.
Hgyam02g1468.1	Hgyaf02g1300.1	Hipf02g1303.1	Hrha02g1115.1
Hgyam02g1474.1	Hgyaf02g1303.1	.	.
Hgyam02g1475.1	Hgyaf02g1304.1	Hipf02g1304.1	Hrha02g1117.1
.	.	.	.
.	.	.	.
.	.	.	.
.	.	.	.
.	.	.	.
.	.	.	.
.	Hgyaf02g1222.1	Hipf02g1291.1	Hrha02g1101.1
.	Hgyaf02g1232.1	Hipf02g1293.2	Hrha02g1104.1
.	Hgyaf02g1232.1	Hipf02g1293.2	Hrha02g1104.1

.	.	.	Hrha02g1105.1
.	.	.	.
.	Hgyaf02g1231.1	Hipf02g1294.1	Hrha02g1106.1
.	Hgyaf02g1230.1	Hipf02g1295.1	Hrha02g1109.1
.	Hgyaf02g1229.1	Hipf02g1296.2	Hrha02g1110.1
.	Hgyaf02g1229.1	Hipf02g1296.2	Hrha02g1110.1
.	Hgyaf02g1229.1	Hipf02g1296.2	Hrha02g1110.1
.	Hgyaf02g1229.1	Hipf02g1296.2	Hrha02g1110.1
.	Hgyaf02g1229.1	Hipf02g1296.2	Hrha02g1110.1
.	Hgyaf02g1229.1	Hipf02g1296.2	Hrha02g1110.1
.	Hgyaf02g1229.1	Hipf02g1296.2	Hrha02g1110.1
.	Hgyaf02g1229.1	Hipf02g1296.2	Hrha02g1110.1
.	Hgyaf02g1229.1	Hipf02g1296.2	Hrha02g1110.1
.	Hgyaf02g1229.1	Hipf02g1296.2	Hrha02g1110.1
.	.	.	.
.	.	.	.
.	.	.	.
Hgyam02g1524.1	Hgyaf02g1340.1	.	Hrha02g1147.1
.	.	.	.
Hgyam02g1525.1	Hgyaf02g1341.1	.	Hrha02g1146.1
Hgyam02g1530.1	Hgyaf02g1343.1	.	.
Hgyam02g1531.1	Hgyaf02g1344.1	.	.
Hgyam02g1532.1	.	.	Hrha02g1150.1
Hgyam02g1533.1	Hgyaf02g1345.1	.	Hrha02g1151.1
Hgyam02g1540.1	Hgyaf02g1349.1	.	.
Hgyam02g1548.1	Hgyaf02g1356.1	.	Hrha02g1156.1
Hgyam02g1549.1	Hgyaf02g1357.1	.	Hrha02g1157.1
.	.	.	.
Hgyam02g1550.1	Hgyaf02g1358.1	.	Hrha02g1160.1
.	Hgyaf02g1359.1	.	.
.	.	.	.
Hgyam02g1551.1	Hgyaf02g1360.1	.	Hrha02g1161.1
Hgyam02g1554.1	Hgyaf02g1362.1	.	.
Hgyam02g1555.1	Hgyaf02g1363.1	.	Hrha02g1163.1
Hgyam02g1555.1	Hgyaf02g1363.1	.	Hrha02g1163.1

Table S11 The Ks values of homologous genes in different blocks within th

block	Hap1 gene	Hap2 gene	Ks
B	Hsam2h02g1200	Hsam1h02g1239	0.5886
B	Hsam2h02g1201	Hsam1h02g1240	0.0085
B	Hsam2h02g1202	Hsam1h02g1245	0.0179
B	Hsam2h02g1204	Hsam1h02g1247	0.0093
B	Hsam2h02g1206	Hsam1h02g1249	0.0961
B	Hsam2h02g1210	Hsam1h02g1258	0.0542
B	Hsam2h02g1211	Hsam1h02g1268	0.0232
B	Hsam2h02g1212	Hsam1h02g1276	0.0141
A1	Hsam2h02g1215	Hsam1h02g1296	0.0142
A1	Hsam2h02g1216	Hsam1h02g1295	0.0131
A1	Hsam2h02g1217	Hsam1h02g1294	0.0191
A1	Hsam2h02g1219	Hsam1h02g1286	0.0174
A1	Hsam2h02g1221	Hsam1h02g1285	0.0150
A1	Hsam2h02g1222	Hsam1h02g1284	0.0070
A2	Hsam2h02g1224	Hsam1h02g1567	0.0303
A2	Hsam2h02g1226	Hsam1h02g1562	0.1008
A2	Hsam2h02g1228	Hsam1h02g1558	0.0100
A2	Hsam2h02g1232	Hsam1h02g1555	0.0164
A2	Hsam2h02g1237	Hsam1h02g1569	0.0352
C	Hsam2h02g1238	Hsam1h02g1320	0.0259
C	Hsam2h02g1239	Hsam1h02g1321	0.0162
C	Hsam2h02g1241	Hsam1h02g1333	0.2361
C	Hsam2h02g1243	Hsam1h02g1331	0.2432
C	Hsam2h02g1245	Hsam1h02g1334	0.0135
C	Hsam2h02g1247	Hsam1h02g1336	0.0211
C	Hsam2h02g1248	Hsam1h02g1339	0.0394
C	Hsam2h02g1250	Hsam1h02g1342	0.0270
C	Hsam2h02g1252	Hsam1h02g1343	0.0119
C	Hsam2h02g1254	Hsam1h02g1345	0.0370
C	Hsam2h02g1255	Hsam1h02g1346	0.0032
C	Hsam2h02g1256	Hsam1h02g1351	0.0172
C	Hsam2h02g1258	Hsam1h02g1355	0.0177
C	Hsam2h02g1263	Hsam1h02g1359	0.0055
C	Hsam2h02g1266	Hsam1h02g1360	0.0403
C	Hsam2h02g1267	Hsam1h02g1362	0.4119
C	Hsam2h02g1269	Hsam1h02g1371	0.0175
D	Hsam2h02g1312	Hsam1h02g1482	0.4759
D	Hsam2h02g1316	Hsam1h02g1481	0.0271
D	Hsam2h02g1317	Hsam1h02g1484	0.0226
D	Hsam2h02g1319	Hsam1h02g1436	1.0098
D	Hsam2h02g1323	Hsam1h02g1437	0.0209
D	Hsam2h02g1324	Hsam1h02g1441	0.0000
D	Hsam2h02g1326	Hsam1h02g1449	0.0100
D	Hsam2h02g1329	Hsam1h02g1451	0.0118

D	Hsam2h02g1331	Hsam1h02g1453	0.0218
D	Hsam2h02g1332	Hsam1h02g1478	0.0101
D	Hsam2h02g1333	Hsam1h02g1476	0.2173
D	Hsam2h02g1335	Hsam1h02g1474	0.2244
D	Hsam2h02g1337	Hsam1h02g1472	0.0197
D	Hsam2h02g1341	Hsam1h02g1470	0.0177
D	Hsam2h02g1344	Hsam1h02g1467	0.0190
D	Hsam2h02g1345	Hsam1h02g1465	0.0665
D	Hsam2h02g1347	Hsam1h02g1464	0.0113
D	Hsam2h02g1350	Hsam1h02g1458	0.0498
D	Hsam2h02g1351	Hsam1h02g1459	0.0173
D	Hsam2h02g1352	Hsam1h02g1462	0.0156
D	Hsam2h02g1355	Hsam1h02g1460	0.0992
D	Hsam2h02g1357	Hsam1h02g1463	0.0100
D	Hsam2h02g1360	Hsam1h02g1456	0.0399
D	Hsam2h02g1361	Hsam1h02g1455	0.5597
D	Hsam2h02g1362	Hsam1h02g1485	0.3131
D	Hsam2h02g1364	Hsam1h02g1383	0.0000
E1	Hsam2h02g1366	Hsam1h02g1385	0.0036
E1	Hsam2h02g1367	Hsam1h02g1386	0.0000
E1	Hsam2h02g1368	Hsam1h02g1387	0.0000
E1	Hsam2h02g1369	Hsam1h02g1388	0.0092
E1	Hsam2h02g1370	Hsam1h02g1391	0.0000
E1	Hsam2h02g1371	Hsam1h02g1392	0.0000
E1	Hsam2h02g1372	Hsam1h02g1393	0.0000
E1	Hsam2h02g1373	Hsam1h02g1394	0.0000
E1	Hsam2h02g1374	Hsam1h02g1395	0.0000
E1	Hsam2h02g1375	Hsam1h02g1396	0.0000
E1	Hsam2h02g1376	Hsam1h02g1397	0.0857
E1	Hsam2h02g1378	Hsam1h02g1398	0.0035
E1	Hsam2h02g1379	Hsam1h02g1400	0.5276
E1	Hsam2h02g1384	Hsam1h02g1402	0.0418
E1	Hsam2h02g1385	Hsam1h02g1407	0.0000
E1	Hsam2h02g1387	Hsam1h02g1409	0.0000
E1	Hsam2h02g1388	Hsam1h02g1410	0.0000
E1	Hsam2h02g1389	Hsam1h02g1411	0.0000
E1	Hsam2h02g1391	Hsam1h02g1412	0.0027
E1	Hsam2h02g1392	Hsam1h02g1415	0.1141
E1	Hsam2h02g1393	Hsam1h02g1416	0.0000
E1	Hsam2h02g1395	Hsam1h02g1417	0.1564
E1	Hsam2h02g1396	Hsam1h02g1418	0.0197
E1	Hsam2h02g1397	Hsam1h02g1423	0.0179
E1	Hsam2h02g1398	Hsam1h02g1425	0.0226
E1	Hsam2h02g1399	Hsam1h02g1427	0.2154
E1	Hsam2h02g1402	Hsam1h02g1435	0.0000
E1	Hsam2h02g1403	Hsam1h02g1432	0.0177
E2	Hsam2h02g1411	Hsam1h02g1487	0.0261

E2	Hsam2h02g1413	Hsam1h02g1504	0.0158
E2	Hsam2h02g1416	Hsam1h02g1503	0.0215
E2	Hsam2h02g1417	Hsam1h02g1502	0.0116
E2	Hsam2h02g1419	Hsam1h02g1501	0.0879
E2	Hsam2h02g1420	Hsam1h02g1500	0.0828
F	Hsam2h02g1431	Hsam1h02g1382	0.0000
F	Hsam2h02g1432	Hsam1h02g1381	0.2513
F	Hsam2h02g1433	Hsam1h02g1380	0.0000
F	Hsam2h02g1434	Hsam1h02g1379	0.0092
F	Hsam2h02g1435	Hsam1h02g1378	0.2246
F	Hsam2h02g1437	Hsam1h02g1377	0.0068
G	Hsam2h02g1438	Hsam1h02g1303	0.0000
G	Hsam2h02g1439	Hsam1h02g1307	0.0000
G	Hsam2h02g1440	Hsam1h02g1308	0.0000
G	Hsam2h02g1442	Hsam1h02g1309	0.0022
G	Hsam2h02g1444	Hsam1h02g1311	0.0218
G	Hsam2h02g1445	Hsam1h02g1312	0.0031
G	Hsam2h02g1446	Hsam1h02g1313	0.0022
G	Hsam2h02g1447	Hsam1h02g1314	0.0000

e SLRs of *H. salicifolia*

average Ks	Method
	YN
	YN
	YN
0.1015	YN
	YN
	YN
	YN
	YN
	YN
	YN
0.0143	YN
	YN
	YN
	YN
	YN
	YN
0.0385	YN
	YN
	YN
	YN
	YN
	YN
	YN
	YN
	YN
	YN
	YN
0.0697	YN
	YN
	YN
	YN
	YN
	YN
	YN
	YN
	YN
	YN
	YN
	YN
	YN
	YN
	YN
	YN
	YN

	YN
	YN
	YN
	YN
0.1266	YN
	YN
	YN
	YN
	YN
	YN
	YN
	YN
	YN
	YN
	YN
	YN
	YN
	YN
	YN
	YN
	YN
	YN
	YN
	YN
	YN
	YN
	YN
	YN
	YN
	YN
	YN
0.0442	YN
	YN
	YN
	YN
	YN
	YN
	YN
	YN
	YN
	YN
	YN
	YN
	YN
	YN
	YN
	YN

	YN
0.0409	YN
	YN
	YN
	YN
	YN
	YN
0.0802	YN
	YN
	YN
	YN
	YN
	YN
	YN
0.0037	YN
	YN
	YN
	YN
	YN

Supplementary Table S12. Statistics of transcriptome sequencing of female and ma

sample	tissue	totalReads	mappedReads
H. gyantsensis _F	*flower buds*	331592369	292555896
H. salicifolia _F	*flower buds*	329413194	310051880
H. gyantsensis _M	*flower buds*	311504982	277398830
H. salicifolia _M	*flower buds*	304753736	271656603

[illegible]le mixed flower buds in *H. gyantsensis* and *H. salicifolia*

mapRate	uniq	uniqrate
88.23%	273324308	82.50%
94.12%	293597638	89.12%
89.05%	259575563	83.51%
89.14%	255861671	83.83%

Table S13 TPM values of genes in the SLR of

	flower				
gene_id	*H. gyantsensis* _F	*H. salicifolia* _F	*H. gyantsensis* _M	*H. salicifolia* _M	*H. gyantsensis* _F
Hsam2h02g1200	0.00	0.00	0.00	0.33	0.00
Hsam2h02g1201	2.00	2.58	2.17	3.33	8.00
Hsam2h02g1202	9.25	9.25	13.06	13.56	25.50
Hsam2h02g1203	0.00	0.00	0.00	0.00	0.00
Hsam2h02g1204	6.97	7.50	11.58	10.39	27.00
Hsam2h02g1205	0.00	0.00	0.00	0.00	0.00
Hsam2h02g1206	46.36	28.33	33.47	32.89	57.00
Hsam2h02g1207	0.00	0.00	0.00	0.00	0.00
Hsam2h02g1208	0.00	0.00	0.00	0.00	0.00
Hsam2h02g1209	2.94	0.00	1.72	0.00	0.00
Hsam2h02g1210	8.75	1.58	9.86	1.89	42.50
Hsam2h02g1211	6.22	1.83	7.06	2.33	39.50
Hsam2h02g1212	1074.50	495.42	366.19	346.50	199.00
Hsam2h02g1213	0.00	0.00	0.00	0.00	0.00
Hsam2h02g1214	0.00	0.00	0.00	0.00	0.00
Hsam2h02g1215	0.00	0.00	0.00	0.00	0.00
Hsam2h02g1216	0.00	0.00	0.00	0.00	0.00
Hsam2h02g1217	1.06	1.33	1.92	1.50	0.00
Hsam2h02g1218	0.00	0.00	0.00	0.56	0.00
Hsam2h02g1219	0.17	0.67	0.06	1.22	0.00
Hsam2h02g1220	0.00	0.17	0.00	0.11	0.00
Hsam2h02g1221	7.61	7.83	8.53	10.78	25.00
Hsam2h02g1222	23.75	23.08	12.19	21.89	51.50
Hsam2h02g1223	0.00	0.00	0.00	0.00	0.00
Hsam2h02g1224	25.81	21.33	23.17	15.50	21.50
Hsam2h02g1225	0.00	0.00	0.00	0.00	0.00
Hsam2h02g1226	0.00	0.00	0.00	0.00	0.00
Hsam2h02g1227	0.00	0.00	0.00	0.00	0.00
Hsam2h02g1228	4.92	4.83	8.22	7.56	14.00
Hsam2h02g1229	0.00	0.00	0.00	0.00	0.00
Hsam2h02g1230	0.00	0.00	0.00	0.00	0.00
Hsam2h02g1231	0.00	0.00	0.00	0.00	0.00
Hsam2h02g1232	51.11	45.92	41.69	30.17	0.00
Hsam2h02g1233	0.00	0.00	0.00	0.00	0.00
Hsam2h02g1234	0.92	0.00	0.17	0.72	4.00
Hsam2h02g1235	0.00	0.00	0.00	0.11	0.00
Hsam2h02g1236	0.00	0.00	0.00	0.06	0.00
Hsam2h02g1237	28.64	17.42	18.42	13.39	28.50
Hsam2h02g1238	3.64	1.08	6.00	6.22	4.50
Hsam2h02g1239	0.50	0.00	1.72	2.39	3.00
Hsam2h02g1240	0.00	0.00	0.00	0.00	0.00
Hsam2h02g1241	0.00	0.00	0.00	0.00	0.00

Hsam2h02g1242	0.00	0.00	0.00	0.00	0.00
Hsam2h02g1243	0.00	0.00	0.00	0.00	0.00
Hsam2h02g1244	0.00	0.00	0.00	0.00	0.50
Hsam2h02g1245	6.47	4.17	5.25	9.11	16.00
Hsam2h02g1246	0.00	0.00	0.00	0.00	0.00
Hsam2h02g1247	10.92	8.25	10.64	14.67	18.00
Hsam2h02g1248	0.00	0.00	93.19	0.00	0.00
Hsam2h02g1249	0.00	0.00	0.00	8.56	0.00
Hsam2h02g1250	6.89	7.42	12.39	11.83	31.50
Hsam2h02g1251	0.50	0.42	0.36	0.56	3.50
Hsam2h02g1252	0.50	0.67	0.47	0.94	2.50
Hsam2h02g1253	1.06	1.25	1.08	1.11	3.50
Hsam2h02g1254	40.61	42.83	61.28	51.67	83.00
Hsam2h02g1255	21.11	20.83	23.25	23.78	24.50
Hsam2h02g1256	4.83	6.50	5.31	7.39	7.50
Hsam2h02g1257	0.00	0.00	0.00	0.00	0.00
Hsam2h02g1258	7.86	14.58	5.58	14.28	37.50
Hsam2h02g1259	0.00	0.00	0.00	0.22	0.00
Hsam2h02g1260	0.00	0.00	0.00	0.83	0.00
Hsam2h02g1261	0.00	0.00	0.00	2.56	0.00
Hsam2h02g1262	0.00	0.00	0.00	3.22	0.00
Hsam2h02g1263	12.08	12.17	6.61	13.67	23.00
Hsam2h02g1264	0.00	0.00	0.00	0.00	0.00
Hsam2h02g1265	0.00	0.00	0.00	0.00	0.00
Hsam2h02g1266	173.03	170.08	147.50	127.50	182.00
Hsam2h02g1267	0.00	0.00	0.00	0.00	0.00
Hsam2h02g1268	0.00	0.00	0.00	0.00	0.00
Hsam2h02g1269	6.31	7.42	14.22	12.39	31.50
Hsam2h02g1270	0.00	0.00	0.00	0.00	0.00
Hsam2h02g1271	0.00	0.00	0.00	0.00	0.00
Hsam2h02g1272	0.00	0.00	0.00	0.00	0.00
Hsam2h02g1273	0.00	0.00	0.00	0.00	0.00
Hsam2h02g1274	0.00	0.00	0.00	0.00	0.00
Hsam2h02g1275	0.00	0.00	0.00	0.00	0.00
Hsam2h02g1276	0.00	0.00	0.00	0.00	0.00
Hsam2h02g1277	0.00	0.00	0.00	0.00	0.00
Hsam2h02g1278	0.00	0.00	0.00	0.00	0.00
Hsam2h02g1279	0.00	0.00	0.00	0.00	0.00
Hsam2h02g1280	0.00	0.00	0.00	0.00	0.00
Hsam2h02g1281	0.00	0.00	0.00	0.00	0.00
Hsam2h02g1282	0.00	0.00	0.00	0.00	0.00
Hsam2h02g1283	0.00	0.00	0.00	0.00	0.00
Hsam2h02g1284	0.00	0.00	0.00	0.00	0.00
Hsam2h02g1285	0.00	0.00	0.00	0.00	0.00
Hsam2h02g1286	0.00	0.00	0.00	0.00	0.00
Hsam2h02g1287	0.00	0.00	0.00	0.00	0.00
Hsam2h02g1288	0.00	0.00	0.00	0.00	0.00

Hsam2h02g1289	0.00	0.00	0.00	0.00	0.00
Hsam2h02g1290	0.00	0.00	0.00	0.00	0.00
Hsam2h02g1291	0.00	0.00	0.00	0.00	0.00
Hsam2h02g1292	0.00	0.00	0.00	0.00	0.00
Hsam2h02g1293	0.00	0.00	0.00	0.00	0.00
Hsam2h02g1294	0.00	0.00	0.00	0.00	0.00
Hsam2h02g1295	0.00	0.00	0.00	0.00	0.00
Hsam2h02g1296	0.00	0.00	0.00	0.00	0.00
Hsam2h02g1297	0.00	0.00	0.00	0.00	0.00
Hsam2h02g1298	0.00	0.00	0.00	0.00	0.00
Hsam2h02g1299	0.00	0.00	0.00	0.00	0.00
Hsam2h02g1300	0.00	0.00	0.00	0.00	0.00
Hsam2h02g1301	0.00	0.00	0.00	0.00	0.00
Hsam2h02g1302	0.00	0.00	0.00	0.00	0.00
Hsam2h02g1303	0.00	0.00	0.00	0.00	0.00
Hsam2h02g1304	0.00	0.00	0.00	0.00	0.00
Hsam2h02g1305	4.03	3.83	12.67	2.28	2.50
Hsam2h02g1306	44.25	85.92	66.89	76.00	44.50
Hsam2h02g1307	0.00	0.00	0.00	0.00	0.00
Hsam2h02g1308	0.00	0.00	0.00	0.00	0.00
Hsam2h02g1309	0.00	0.00	0.00	0.00	0.00
Hsam2h02g1310	0.00	0.00	0.00	0.00	0.00
Hsam2h02g1311	0.00	0.00	0.00	0.00	0.00
Hsam2h02g1312	0.00	0.00	0.00	0.00	0.00
Hsam2h02g1313	0.00	0.00	0.00	0.00	0.00
Hsam2h02g1314	0.00	0.00	0.39	0.00	0.00
Hsam2h02g1315	0.00	0.00	0.33	0.67	0.00
Hsam2h02g1316	20.97	28.25	28.06	31.17	32.50
Hsam2h02g1317	5.58	2.83	6.03	4.33	11.00
Hsam2h02g1318	0.08	0.08	0.06	0.11	1.00
Hsam2h02g1319	0.00	0.00	0.00	0.00	0.00
Hsam2h02g1320	0.00	0.00	0.00	0.00	0.00
Hsam2h02g1321	0.00	0.00	0.00	0.00	0.00
Hsam2h02g1322	0.00	0.00	0.00	0.00	0.00
Hsam2h02g1323	19.00	17.67	35.22	49.17	42.50
Hsam2h02g1324	15.83	22.83	22.14	35.28	33.50
Hsam2h02g1325	0.00	0.00	0.00	0.00	0.00
Hsam2h02g1326	0.83	1.50	0.67	3.44	6.00
Hsam2h02g1327	0.00	0.00	0.00	0.00	0.00
Hsam2h02g1328	0.00	0.17	0.00	1.28	0.00
Hsam2h02g1329	0.50	1.00	0.42	2.83	16.50
Hsam2h02g1330	0.17	0.25	0.25	1.78	14.00
Hsam2h02g1331	17.06	15.58	19.69	26.61	11.50
Hsam2h02g1332	11.67	14.75	14.00	18.17	24.00
Hsam2h02g1333	0.00	0.00	0.00	0.00	0.00
Hsam2h02g1334	0.00	0.00	0.00	0.00	0.00
Hsam2h02g1335	0.00	0.00	0.00	0.00	0.00

Hsam2h02g1336	0.00	0.00	0.00	0.00	0.00
Hsam2h02g1337	0.50	1.58	11.50	3.72	3.50
Hsam2h02g1338	0.00	0.00	0.00	0.00	0.00
Hsam2h02g1339	3.28	0.00	2.61	5.06	0.00
Hsam2h02g1340	0.00	0.00	0.00	11.50	0.00
Hsam2h02g1341	2.08	2.75	7.11	6.67	18.00
Hsam2h02g1342	0.00	0.00	0.00	0.00	0.00
Hsam2h02g1343	0.00	0.00	0.00	0.00	0.00
Hsam2h02g1344	0.00	0.00	0.00	0.00	0.00
Hsam2h02g1345	229.89	0.17	197.22	11.83	246.50
Hsam2h02g1346	0.00	0.00	0.00	0.00	0.00
Hsam2h02g1347	2.06	2.33	0.69	4.67	6.00
Hsam2h02g1348	0.00	0.00	0.00	0.00	0.00
Hsam2h02g1349	0.00	0.00	0.00	0.00	0.00
Hsam2h02g1350	0.00	0.00	0.00	0.00	0.00
Hsam2h02g1351	1.61	2.00	2.72	2.72	6.50
Hsam2h02g1352	0.00	0.00	0.00	0.00	0.00
Hsam2h02g1353	11.14	9.42	15.17	7.67	12.50
Hsam2h02g1354	0.00	0.00	0.00	0.00	0.00
Hsam2h02g1355	11.14	9.42	15.17	7.67	12.50
Hsam2h02g1356	0.00	0.00	0.00	0.00	0.00
Hsam2h02g1357	3.22	0.00	6.86	2.72	3.50
Hsam2h02g1358	0.00	0.00	0.00	0.00	0.00
Hsam2h02g1359	0.00	0.00	0.00	0.00	0.00
Hsam2h02g1360	0.00	0.00	0.00	0.00	0.00
Hsam2h02g1361	0.00	0.00	0.00	0.00	0.00
Hsam2h02g1362	0.92	1.42	0.67	4.39	3.50
Hsam2h02g1363	0.00	0.00	0.00	8.33	0.00
Hsam2h02g1364	28.00	39.58	22.42	27.06	24.00
Hsam2h02g1365	0.00	1.92	0.75	1.72	5.50
Hsam2h02g1366	11.33	5.25	11.19	6.78	6.50
Hsam2h02g1367	137.44	105.92	117.19	96.94	83.50
Hsam2h02g1368	0.00	0.00	0.00	0.00	0.00
Hsam2h02g1369	0.00	0.00	0.00	0.00	0.00
Hsam2h02g1370	0.00	0.00	0.00	0.00	0.00
Hsam2h02g1371	0.00	0.00	0.33	0.00	0.00
Hsam2h02g1372	1.61	1.92	3.36	2.72	11.00
Hsam2h02g1373	1.33	0.00	3.06	0.00	0.00
Hsam2h02g1374	0.11	0.00	0.61	0.00	0.00
Hsam2h02g1375	0.00	0.00	0.00	0.00	0.00
Hsam2h02g1376	0.00	0.00	0.00	0.00	0.00
Hsam2h02g1377	0.00	0.00	0.00	0.00	0.00
Hsam2h02g1378	30.89	43.17	34.31	45.06	35.00
Hsam2h02g1379	11.56	0.00	6.75	0.00	5.00
Hsam2h02g1380	0.00	0.00	0.00	0.00	0.00
Hsam2h02g1381	0.00	0.00	0.11	0.00	0.00
Hsam2h02g1382	0.00	0.00	0.00	0.00	0.00

Hsam2h02g1383	0.00	0.00	0.00	0.00	0.00
Hsam2h02g1384	0.00	0.00	0.75	0.00	0.00
Hsam2h02g1385	0.00	0.00	0.00	0.00	0.00
Hsam2h02g1386	0.00	0.00	0.00	0.00	0.00
Hsam2h02g1387	90.58	93.92	122.42	108.83	100.50
Hsam2h02g1388	19.42	13.17	19.86	8.72	11.50
Hsam2h02g1389	6.81	7.67	13.78	11.39	26.00
Hsam2h02g1390	0.00	0.00	0.00	0.00	0.00
Hsam2h02g1391	10.58	11.42	15.83	23.78	63.50
Hsam2h02g1392	0.00	0.00	0.00	0.00	0.00
Hsam2h02g1393	0.00	0.00	0.00	0.00	0.00
Hsam2h02g1394	0.00	0.00	0.00	0.00	0.00
Hsam2h02g1395	0.00	0.00	0.00	0.00	0.00
Hsam2h02g1396	16.97	23.08	19.00	26.17	20.50
Hsam2h02g1397	5.33	6.25	15.22	6.44	21.50
Hsam2h02g1398	0.42	1.08	1.00	1.17	1.50
Hsam2h02g1399	0.36	0.00	0.53	1.39	0.00
Hsam2h02g1400	0.00	0.00	0.00	0.00	0.00
Hsam2h02g1401	0.00	0.00	0.00	0.00	0.00
Hsam2h02g1402	22.50	22.33	26.58	16.33	56.00
Hsam2h02g1403	0.08	0.08	0.00	0.44	0.00
Hsam2h02g1404	13.83	0.00	19.25	17.28	38.00
Hsam2h02g1405	0.00	0.00	0.00	0.00	0.00
Hsam2h02g1406	0.00	0.00	0.00	0.00	0.00
Hsam2h02g1407	0.00	0.00	0.00	0.00	0.00
Hsam2h02g1408	0.00	0.00	0.00	0.00	0.00
Hsam2h02g1409	0.00	0.00	0.00	0.00	0.00
Hsam2h02g1410	0.00	0.00	0.00	0.00	0.00
Hsam2h02g1411	10.14	6.50	12.47	14.06	38.50
Hsam2h02g1412	4.58	4.50	2.28	4.78	7.00
Hsam2h02g1413	1.42	1.75	0.75	2.17	5.50
Hsam2h02g1414	0.78	0.00	2.64	0.11	0.00
Hsam2h02g1415	0.00	0.00	0.75	0.00	0.00
Hsam2h02g1416	0.00	0.00	0.00	0.00	0.00
Hsam2h02g1417	13.97	12.92	14.42	16.28	10.00
Hsam2h02g1418	0.00	0.00	0.00	0.00	0.50
Hsam2h02g1419	0.08	0.00	0.08	0.11	0.00
Hsam2h02g1420	0.00	0.00	0.00	0.00	0.00
Hsam2h02g1421	0.00	0.00	0.00	0.00	0.00
Hsam2h02g1422	0.00	0.00	0.00	0.00	0.00
Hsam2h02g1423	0.00	0.00	0.00	0.00	0.00
Hsam2h02g1424	0.00	0.00	0.00	0.00	0.00
Hsam2h02g1425	0.00	0.00	0.00	0.00	0.00
Hsam2h02g1426	0.00	0.00	0.00	0.00	0.00
Hsam2h02g1427	0.08	0.00	0.08	0.22	0.50
Hsam2h02g1428	0.33	0.00	1.28	0.00	1.00
Hsam2h02g1429	0.00	0.00	0.00	0.00	0.00

Hsam2h02g1430	0.00	0.00	0.00	0.00	0.00
Hsam2h02g1431	8.86	8.92	8.58	10.00	23.00
Hsam2h02g1432	0.00	0.00	0.00	0.00	0.00
Hsam2h02g1433	0.00	0.42	0.00	0.39	2.00
Hsam2h02g1434	16.92	21.67	8.61	11.50	12.50
Hsam2h02g1435	0.00	0.00	0.00	0.00	0.00
Hsam2h02g1436	0.00	0.00	10.22	4.56	0.00
Hsam2h02g1437	0.00	0.17	100.33	2.39	0.00
Hsam2h02g1438	0.00	0.00	0.00	0.00	0.00
Hsam2h02g1439	3.44	4.50	6.00	7.22	15.00
Hsam2h02g1440	91.00	54.00	44.69	57.11	47.50
Hsam2h02g1441	0.00	0.00	0.00	0.00	0.00
Hsam2h02g1442	2.94	1.25	3.42	2.39	1.00
Hsam2h02g1443	0.00	0.00	0.00	0.00	0.00
Hsam2h02g1444	0.00	0.00	0.00	0.00	0.00
Hsam2h02g1445	0.72	1.25	0.67	1.67	6.50
Hsam2h02g1446	0.83	2.83	0.33	3.50	2.50
Hsam2h02g1447	0.00	0.00	0.00	0.00	0.00
Hsam2h02g1448	0.00	0.00	0.00	0.00	0.00

H. salicifolia across different tissues in males and females

stem				leaves	
H. gyantsensis _M	*H. salicifolia* _F	*H. salicifolia* _M	*H. gyantsensis* _F	*H. gyantsensis* _M	*H. salicifolia* _F
0.00	0.00	0.00	0.00	6.00	0.00
5.50	6.17	5.50	5.00	5.00	3.00
22.50	19.17	17.67	15.00	13.00	8.17
0.00	0.00	0.00	0.00	0.00	0.00
25.50	21.67	19.83	21.00	16.00	15.33
0.00	0.00	0.00	0.00	0.00	0.00
23.50	12.17	15.50	109.50	61.00	26.00
0.00	0.00	0.00	0.00	0.00	0.00
0.00	0.00	0.00	0.00	0.00	0.00
0.00	0.00	0.00	0.00	0.00	0.00
14.00	11.50	5.83	22.00	7.00	2.50
10.00	13.50	8.33	20.00	3.00	3.17
188.00	122.17	209.50	121.00	108.00	92.67
0.00	0.00	0.00	0.00	0.00	0.00
0.00	0.00	0.00	0.00	0.00	0.00
0.00	33.33	3.67	0.00	0.00	0.00
0.00	0.00	0.00	0.00	0.00	0.00
2.00	1.17	1.17	0.00	2.00	2.17
0.00	0.00	0.17	0.00	0.00	0.00
0.00	3.00	5.17	0.00	0.00	0.00
0.00	0.00	0.00	0.00	0.00	0.00
19.00	17.33	13.00	21.50	20.00	15.67
17.00	28.33	18.50	39.50	14.00	18.67
0.00	0.00	0.17	0.00	0.00	0.00
19.50	18.00	17.00	15.50	14.00	15.00
0.00	0.00	0.33	0.00	0.00	0.00
0.00	0.00	0.00	0.00	0.00	0.00
0.00	0.00	0.00	0.00	0.00	0.00
15.00	18.50	15.00	15.00	12.00	22.33
0.00	0.00	0.00	0.00	0.00	0.00
0.00	0.00	0.00	0.00	0.00	0.00
0.00	0.00	0.00	0.00	0.00	0.00
0.00	0.00	0.00	0.00	0.00	0.00
0.00	0.00	0.00	0.00	0.00	0.00
3.00	0.00	1.00	3.50	4.00	0.00
0.00	0.00	0.00	0.00	0.00	0.00
0.00	0.00	0.00	0.00	0.00	0.00
29.50	15.17	13.17	71.00	57.00	20.67
0.00	7.00	4.00	2.00	1.00	2.00
0.00	4.00	2.00	1.00	0.00	1.50
0.00	0.00	0.00	0.00	0.00	0.00
0.00	0.00	0.00	0.00	0.00	0.00

0.00	0.00	0.00	0.00	0.00	0.00
0.00	0.00	0.00	0.00	0.00	0.00
0.00	0.00	0.00	0.00	0.00	0.00
12.00	4.50	9.17	13.00	6.00	3.00
0.00	0.00	0.00	0.00	0.00	0.00
18.50	12.83	16.00	14.00	10.00	10.17
0.00	0.00	0.00	0.00	0.00	0.00
0.00	0.00	6.33	0.00	0.00	0.00
25.50	25.00	23.50	19.00	17.00	15.83
1.50	3.00	2.17	2.00	2.00	2.17
2.00	2.17	1.50	2.50	1.00	1.50
2.00	3.00	1.67	2.50	2.00	3.50
79.50	76.00	77.17	54.50	45.00	45.67
30.50	19.17	34.67	11.00	8.00	11.00
4.00	15.00	11.67	3.00	1.00	6.33
0.00	0.00	0.00	0.00	0.00	0.00
40.50	15.67	27.83	2.50	1.00	0.50
0.00	0.00	0.17	0.00	0.00	0.00
0.00	0.00	1.33	0.00	0.00	0.00
0.00	0.00	2.00	0.00	0.00	0.00
0.00	0.00	1.67	0.00	0.00	0.00
51.50	18.33	38.83	6.50	7.00	2.00
0.00	0.00	0.00	0.00	0.00	0.00
0.00	0.00	0.00	0.00	0.00	0.00
272.00	149.17	206.33	141.50	104.00	64.33
0.00	0.00	0.00	0.00	0.00	0.00
0.00	0.00	0.00	0.00	0.00	0.00
24.00	30.83	25.50	31.00	23.00	31.33
0.00	0.00	0.00	0.00	0.00	0.00
0.00	0.00	0.00	0.00	0.00	0.00
0.00	0.00	0.00	0.00	0.00	0.00
0.00	0.00	0.00	0.00	0.00	0.00
0.00	0.00	0.00	0.00	0.00	0.00
0.00	0.00	0.00	0.00	0.00	0.00
0.00	0.00	0.00	0.00	0.00	0.00
0.00	0.00	0.00	0.00	0.00	0.00
0.00	0.00	0.00	0.00	0.00	0.00
0.00	0.00	0.00	0.00	0.00	0.00
0.00	0.00	0.00	0.00	0.00	0.00
0.00	0.00	0.00	0.00	0.00	0.00
0.00	0.00	0.00	0.00	0.00	0.00
0.00	0.00	0.00	0.00	0.00	0.00
0.00	0.00	0.00	0.00	0.00	0.00
0.00	0.00	0.00	0.00	0.00	0.00
0.00	0.00	0.00	0.00	0.00	0.00
0.00	0.00	0.00	0.00	0.00	0.00
0.00	0.00	0.00	0.00	0.00	0.00

0.00	0.00	0.00	0.00	0.00	0.00
0.00	0.00	0.00	0.00	0.00	0.00
0.00	0.00	0.00	0.00	0.00	0.00
0.00	0.00	0.00	0.00	0.00	0.00
0.00	0.00	0.00	0.00	0.00	0.00
0.00	0.00	0.00	0.00	0.00	0.00
0.00	0.00	0.00	0.00	0.00	0.00
0.00	0.00	0.00	0.00	0.00	0.00
0.00	0.00	0.00	0.00	0.00	0.00
0.00	0.00	0.00	0.00	0.00	0.00
0.00	0.00	0.00	0.00	0.00	0.00
0.00	0.00	0.00	0.00	0.00	0.00
0.00	0.00	0.00	0.00	0.00	0.00
0.00	0.00	0.00	0.00	0.00	0.00
0.00	0.00	0.00	0.00	0.00	0.00
0.00	0.00	0.00	0.00	0.00	0.00
1.00	0.00	0.00	2.00	6.00	1.50
55.50	116.33	93.00	32.50	36.00	57.50
0.00	0.00	0.00	0.00	0.00	0.00
0.00	0.00	0.00	0.00	0.00	0.00
0.00	0.00	0.00	0.00	0.00	0.00
0.00	0.00	0.00	0.00	0.00	0.00
0.00	0.00	0.00	0.00	0.00	0.00
0.00	0.00	0.00	0.00	0.00	0.00
0.00	0.00	0.00	0.00	0.00	0.00
0.00	0.00	0.00	0.00	0.00	0.00
0.50	0.00	0.83	0.00	0.00	0.00
46.50	24.83	24.33	56.00	97.00	45.33
5.00	11.67	7.50	12.50	8.00	12.00
0.00	2.83	0.83	0.50	0.00	2.00
0.00	0.00	0.00	0.00	0.00	0.00
0.00	0.00	0.00	0.00	0.00	0.00
0.00	0.00	0.00	0.00	0.00	0.00
0.00	0.00	0.00	0.00	0.00	0.00
50.50	54.17	62.17	3.00	3.00	10.67
14.00	22.83	32.17	13.50	0.00	21.33
0.00	0.00	0.17	0.00	0.00	0.00
2.00	2.33	3.17	3.00	2.00	1.50
0.00	0.00	0.00	0.00	0.00	0.00
0.00	1.67	3.00	0.00	0.00	0.83
5.50	7.33	6.50	14.00	5.00	6.50
5.00	5.83	5.00	13.00	5.00	5.00
5.50	61.00	47.33	12.50	4.00	59.67
26.50	22.67	17.83	17.00	14.00	12.50
0.00	0.00	0.00	0.00	0.00	0.00
0.00	0.00	0.00	0.00	0.00	0.00
0.00	0.00	0.00	0.00	0.00	0.00

0.00	0.00	0.00	0.00	0.00	0.00
0.50	19.83	6.00	5.50	1.00	7.50
0.00	0.00	0.00	0.00	0.00	0.00
5.50	0.00	3.17	0.00	12.00	0.00
0.00	0.00	9.17	0.00	0.00	0.00
21.00	13.00	12.00	15.00	19.00	11.00
0.00	0.00	0.00	0.00	0.00	0.00
0.00	0.00	0.00	0.00	0.00	0.00
0.00	0.00	0.00	0.00	0.00	0.00
202.50	1.33	23.83	475.50	320.00	0.00
0.00	0.00	0.00	0.00	0.00	0.00
2.50	10.00	7.83	5.00	3.00	2.67
0.00	0.00	0.00	0.00	0.00	0.00
0.00	0.00	0.00	0.00	0.00	0.00
0.00	0.00	0.00	0.00	0.00	0.00
6.00	6.33	6.00	4.00	4.00	3.00
0.00	0.00	0.00	0.00	0.00	0.00
15.50	7.00	7.17	9.00	5.00	3.67
0.00	0.00	0.00	0.00	0.00	0.00
15.50	7.00	7.17	9.00	5.00	3.67
0.00	0.00	0.00	0.00	0.00	0.00
11.00	1.67	12.17	0.00	0.00	0.00
0.00	0.00	0.00	0.00	0.00	0.00
0.00	0.00	0.00	0.00	0.00	0.00
0.00	0.00	0.00	0.00	0.00	0.00
0.00	0.00	0.00	0.00	0.00	0.00
1.50	4.17	4.00	6.50	6.00	5.67
0.00	0.00	2.33	0.00	0.00	0.00
19.00	34.67	13.33	16.00	16.00	21.50
0.00	5.83	3.33	0.00	0.00	1.33
6.50	6.17	3.83	5.00	5.00	3.33
61.50	60.17	64.00	44.00	30.00	34.50
0.00	0.00	0.00	0.00	0.00	0.00
0.00	0.00	0.00	0.00	0.00	0.00
0.00	0.00	0.00	0.00	0.00	0.00
0.00	0.00	0.00	0.00	0.00	0.00
8.50	7.33	6.33	6.50	5.00	3.83
0.00	0.00	0.00	0.00	0.00	0.00
0.00	0.00	0.00	0.00	0.00	0.00
0.00	0.00	0.00	0.00	0.00	0.00
0.00	0.00	0.00	0.00	0.00	0.00
0.00	0.00	0.00	0.00	0.00	0.00
34.00	21.67	11.83	549.00	419.00	266.83
10.00	0.00	0.00	2.50	4.00	0.00
0.00	0.00	0.00	0.00	0.00	0.00
0.00	0.00	0.00	0.00	0.00	0.00
0.00	0.00	0.00	0.00	0.00	0.00

0.00	0.00	0.00	0.00	0.00	0.00
0.00	0.00	0.00	0.00	0.00	0.00
0.00	0.00	0.00	0.00	0.00	0.00
0.00	0.00	0.00	0.00	0.00	0.00
54.00	277.17	118.33	189.00	169.00	71.67
10.50	7.67	6.17	20.00	16.00	5.83
17.50	12.17	19.00	33.00	19.00	8.00
0.00	0.00	0.00	0.00	0.00	0.00
54.50	58.17	51.67	61.00	37.00	38.00
0.00	0.00	0.00	0.00	0.00	0.00
0.00	0.00	0.00	0.00	0.00	0.00
0.00	0.00	0.00	0.00	0.00	0.00
0.00	0.00	0.00	0.00	0.00	0.00
20.50	22.33	20.00	13.50	15.00	16.50
19.50	13.67	11.00	11.50	10.00	6.17
1.50	6.67	6.50	0.00	0.00	0.00
0.00	0.00	0.67	0.00	0.00	0.00
0.00	0.00	0.00	0.00	0.00	0.00
0.00	0.00	0.00	0.00	0.00	0.00
51.50	45.83	21.17	40.50	31.00	32.67
0.00	0.00	0.00	0.00	0.00	0.00
30.00	0.00	17.67	19.50	22.00	0.00
0.00	0.00	0.00	0.00	0.00	0.00
0.00	0.00	0.00	0.00	0.00	0.00
0.00	0.00	0.00	0.00	0.00	0.00
0.00	0.00	0.00	0.00	0.00	0.00
0.00	0.00	0.00	0.00	0.00	0.00
0.00	0.00	0.00	0.00	0.00	0.00
22.50	12.83	13.50	81.00	53.00	33.00
4.00	4.33	2.67	2.50	4.00	1.00
2.50	3.00	2.50	2.00	2.00	0.33
3.50	0.00	0.33	0.00	1.00	0.00
0.00	0.00	0.00	0.00	0.00	0.00
0.00	0.00	0.00	0.00	0.00	0.00
10.50	10.17	14.00	7.50	9.00	6.67
0.00	0.17	0.33	0.50	0.00	0.00
0.50	0.17	0.33	0.00	0.00	0.00
0.00	0.00	0.00	0.00	0.00	0.00
0.00	0.00	0.00	0.00	0.00	0.00
0.00	0.00	0.00	0.00	0.00	0.00
0.00	0.00	0.00	0.00	0.00	0.00
0.00	0.00	0.00	0.00	0.00	0.00
0.00	0.00	0.00	0.00	0.00	0.00
0.00	0.00	0.00	0.00	0.00	0.00
1.50	0.83	0.17	0.00	0.00	0.17
0.00	0.00	0.00	2.50	2.00	0.00
0.00	0.00	0.00	0.00	0.00	0.00

0.00	0.00	0.00	0.00	0.00	0.00
30.50	9.17	9.00	7.00	5.00	4.50
0.00	0.00	0.00	0.00	0.00	0.00
1.00	1.00	0.83	0.00	0.00	0.00
58.50	12.83	24.83	16.00	11.00	0.83
0.00	0.00	0.00	0.00	0.00	0.00
0.00	0.00	0.00	0.00	0.00	0.00
0.00	0.00	0.00	0.00	0.00	0.00
0.00	0.00	0.00	0.00	0.00	0.00
15.50	11.00	15.67	6.00	8.00	4.83
44.00	40.17	39.67	23.50	19.00	24.00
0.00	0.00	0.00	0.00	0.00	0.00
1.00	1.00	2.00	0.50	1.00	0.00
0.00	0.00	0.50	0.00	0.00	0.33
0.00	0.00	0.00	0.00	0.00	0.00
4.00	5.00	2.33	2.00	0.00	0.33
1.50	8.17	5.83	5.50	5.00	0.00
0.00	0.33	0.00	0.00	0.00	0.00
0.00	1.33	0.00	0.00	0.00	0.00

H. salicifolia _M
0.00
4.17
8.67
0.00
13.33
0.00
25.83
0.00
0.00
0.00
0.33
1.00
140.00
0.00
0.00
0.00
0.00
1.33
1.00
0.83
0.00
15.67
12.50
0.00
10.50
0.00
0.00
0.00
15.67
0.00
0.00
0.00
0.00
0.00
0.00
0.00
0.00
16.50
3.00
2.50
0.00
0.00

0.00
0.00
0.00
6.00
0.00
10.83
0.00
0.67
18.00
1.67
1.33
2.33
38.50
13.83
4.67
0.00
4.50
0.00
0.00
1.50
2.17
4.17
0.00
0.00
43.33
0.00
0.00
27.33
0.00
0.00
0.00
0.00
0.00
0.00
0.00
0.00
0.00
0.00
0.00
0.00
0.00
0.00
0.00
0.00
0.00
0.00
0.00

0.00
0.00
0.00
0.00
0.00
0.00
0.00
0.00
0.00
0.00
0.00
0.00
0.00
0.00
0.00
0.00
1.83
51.33
0.00
0.00
0.00
0.00
0.00
0.00
0.00
0.00
1.67
37.83
8.83
0.33
0.00
0.00
0.00
0.00
18.83
17.67
0.33
2.67
0.00
2.50
5.83
5.17
35.33
13.17
0.00
0.00
0.00

0.00
2.17
0.00
22.33
6.67
11.50
0.00
0.00
0.00
65.83
0.00
4.50
0.00
0.00
0.00
3.33
0.00
3.33
0.00
3.33
0.00
0.17
0.00
0.00
0.00
0.00
13.33
16.67
12.17
0.00
4.50
28.00
0.00
0.00
0.00
0.00
3.83
0.00
0.00
0.00
0.00
0.00
115.33
0.00
0.00
0.00
0.00

0.00
0.00
0.00
0.00
128.83
6.00
13.33
0.00
30.83
0.00
0.00
0.00
0.00
16.33
8.83
0.67
0.00
0.00
0.00
14.83
0.00
17.50
0.00
0.00
0.00
0.00
0.00
0.00
34.50
2.83
2.67
0.00
0.00
0.00
9.33
0.00
0.00
0.00
0.00
0.00
0.00
0.00
0.00
0.00
0.17
0.00
0.00

0.00
4.50
0.00
0.00
0.50
0.00
0.33
0.00
0.00
6.17
28.83
0.00
0.83
0.00
0.00
0.00
0.00
0.00
0.00

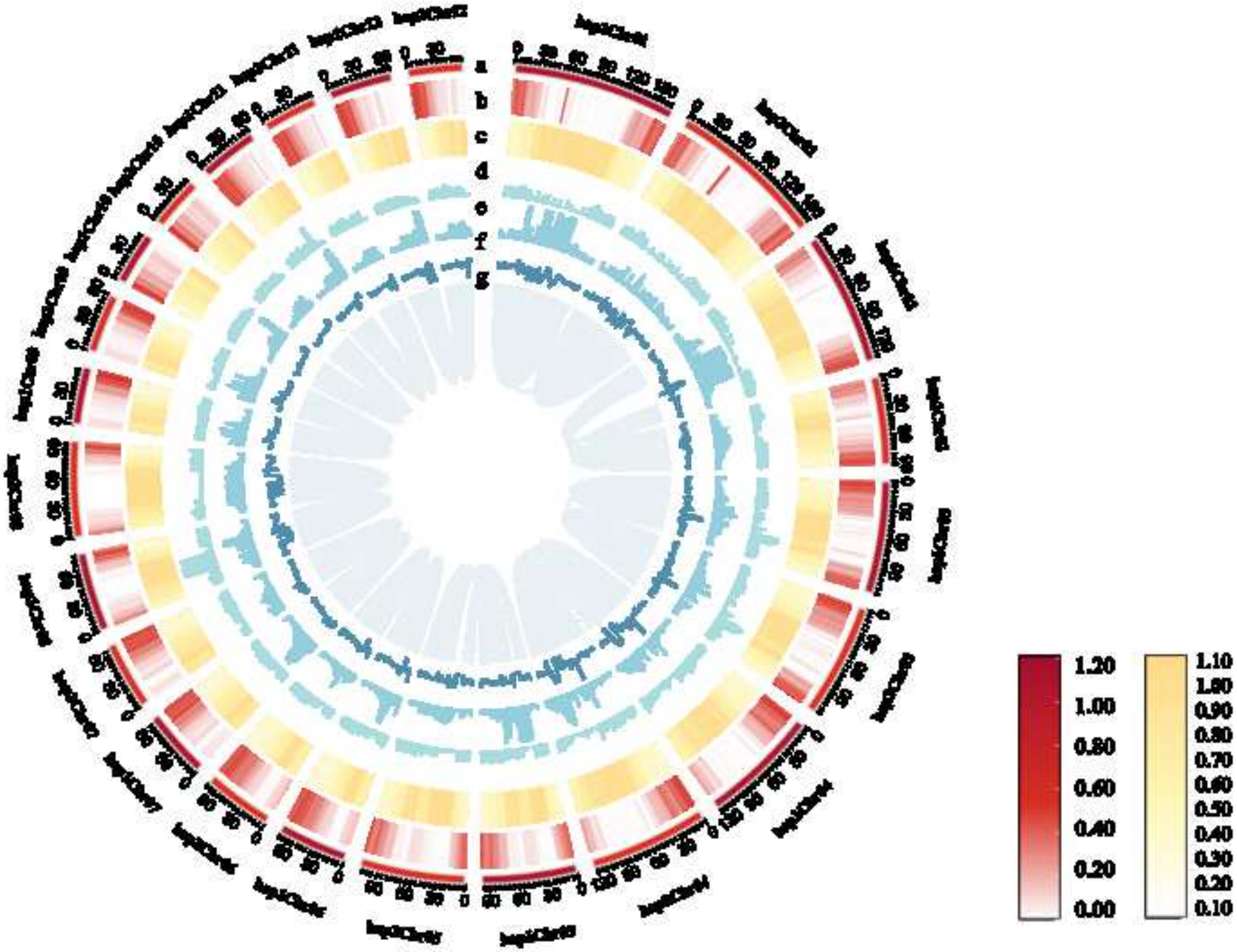
a
b
c
d
e
f
g
1.20
1.00
0.80
0.60
0.40
0.20
0.00
1.10
1.00
0.90
0.80
0.70
0.60
0.50
0.40
0.30
0.20
0.10

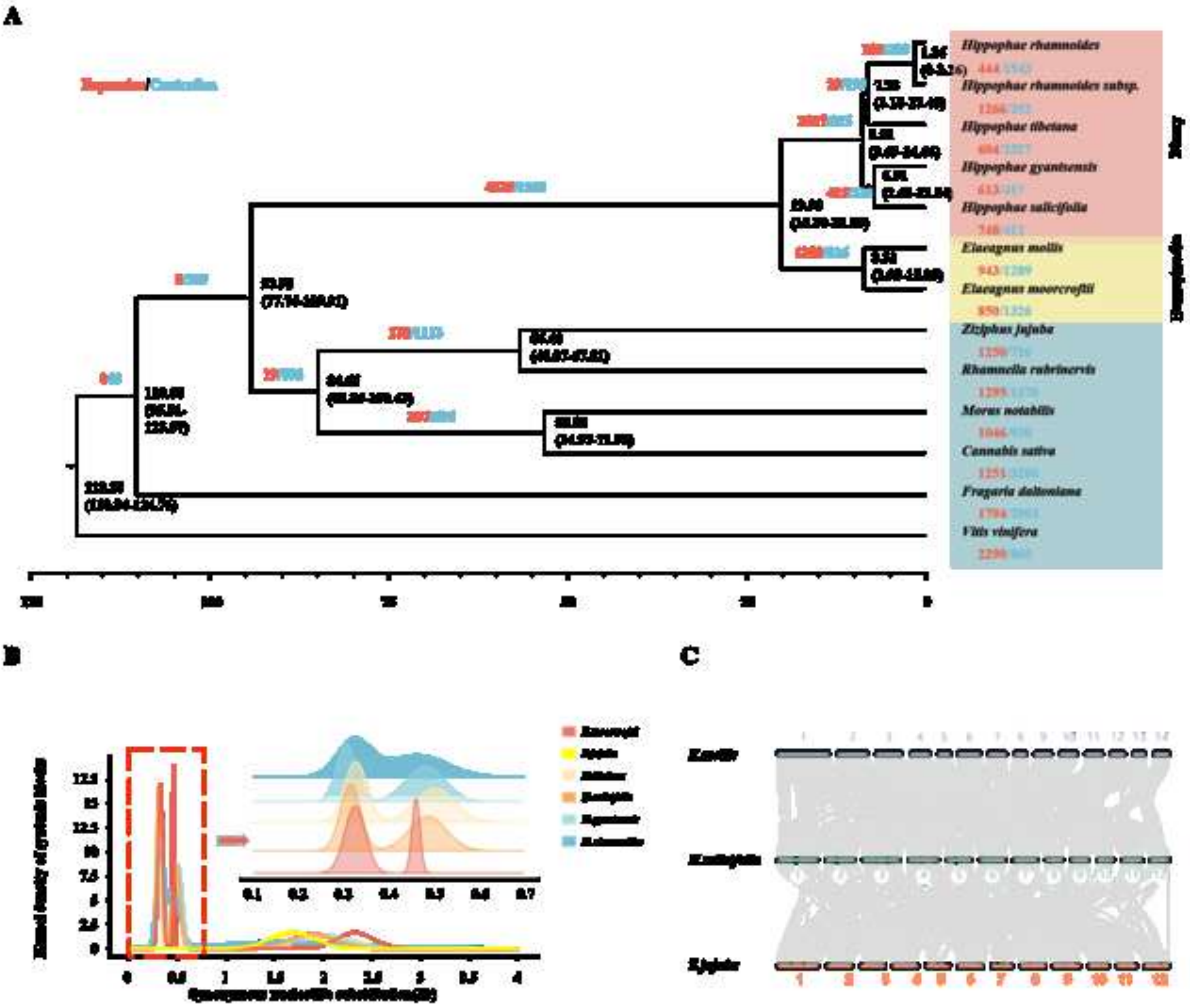
A
Hippophae rhamnoides
Hippophae rhamnoides subsp.
Hippophae tibetana
Hippophae gyantsensis
Hippophae salicifolia
Elaeagnus mollis
Elaeagnus moorcroftii
Ziziphus jujuba
Rhamnella rubrinervis
Morus notabilis
Cannabis sativa
Fragaria daltoniana
Vitis vinifera
B
C

Figure4

Click here to access/download;Figure;Fig4.tif

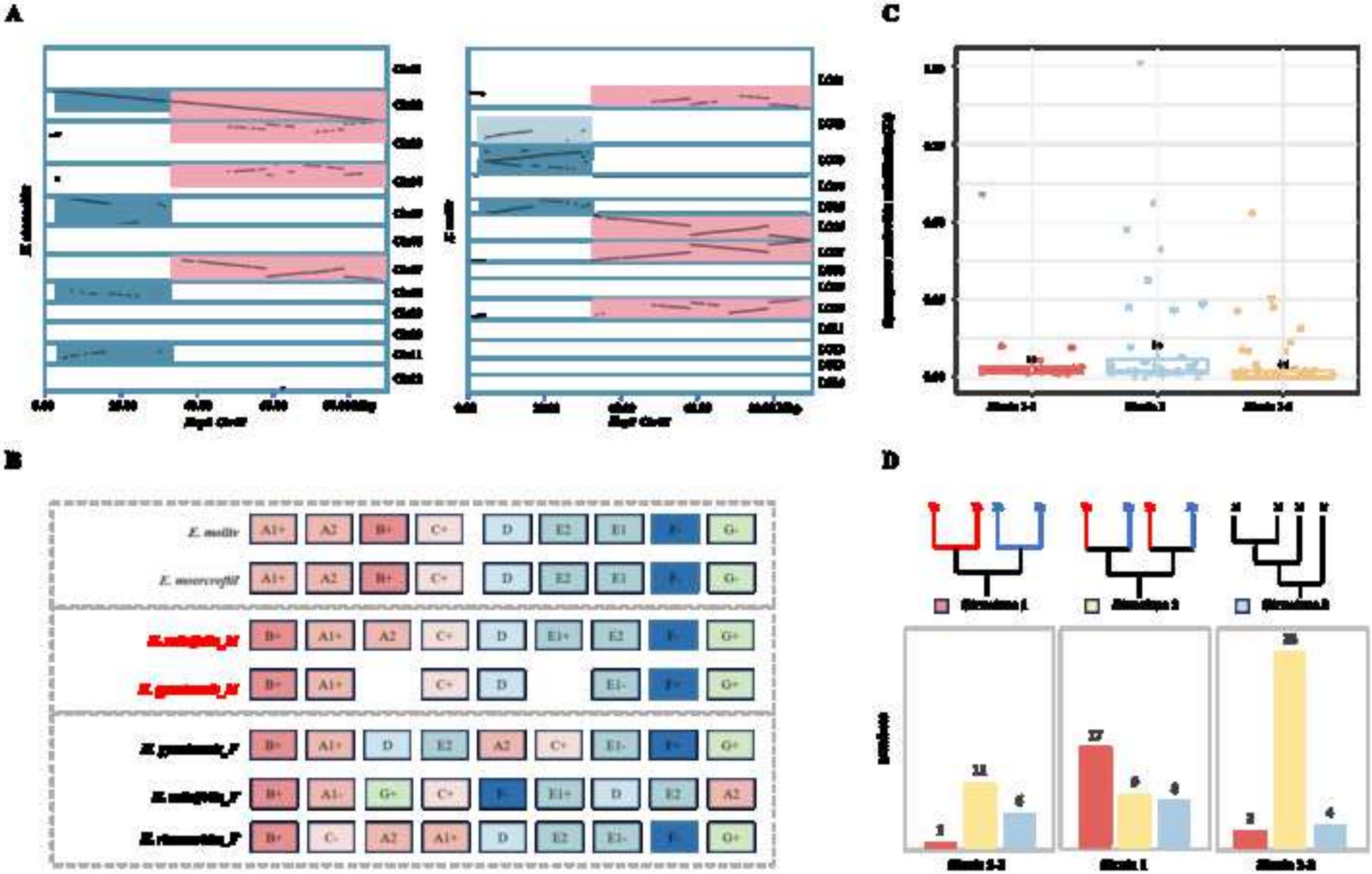

Figure5 Click here to access/download;Figure;Fig5.tif

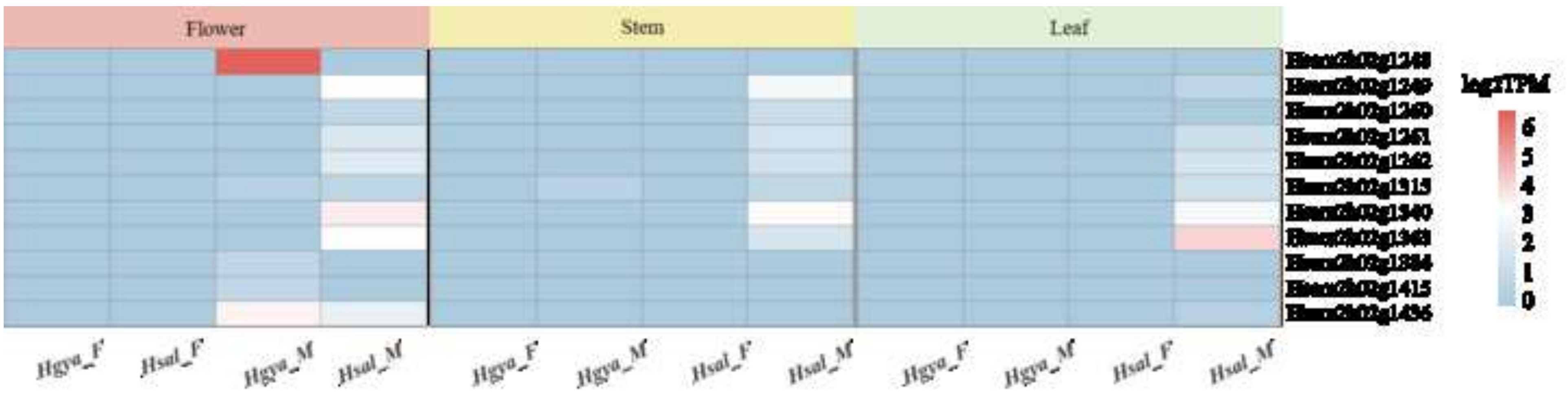

Click here to access/download;Figure;Fig3.tif

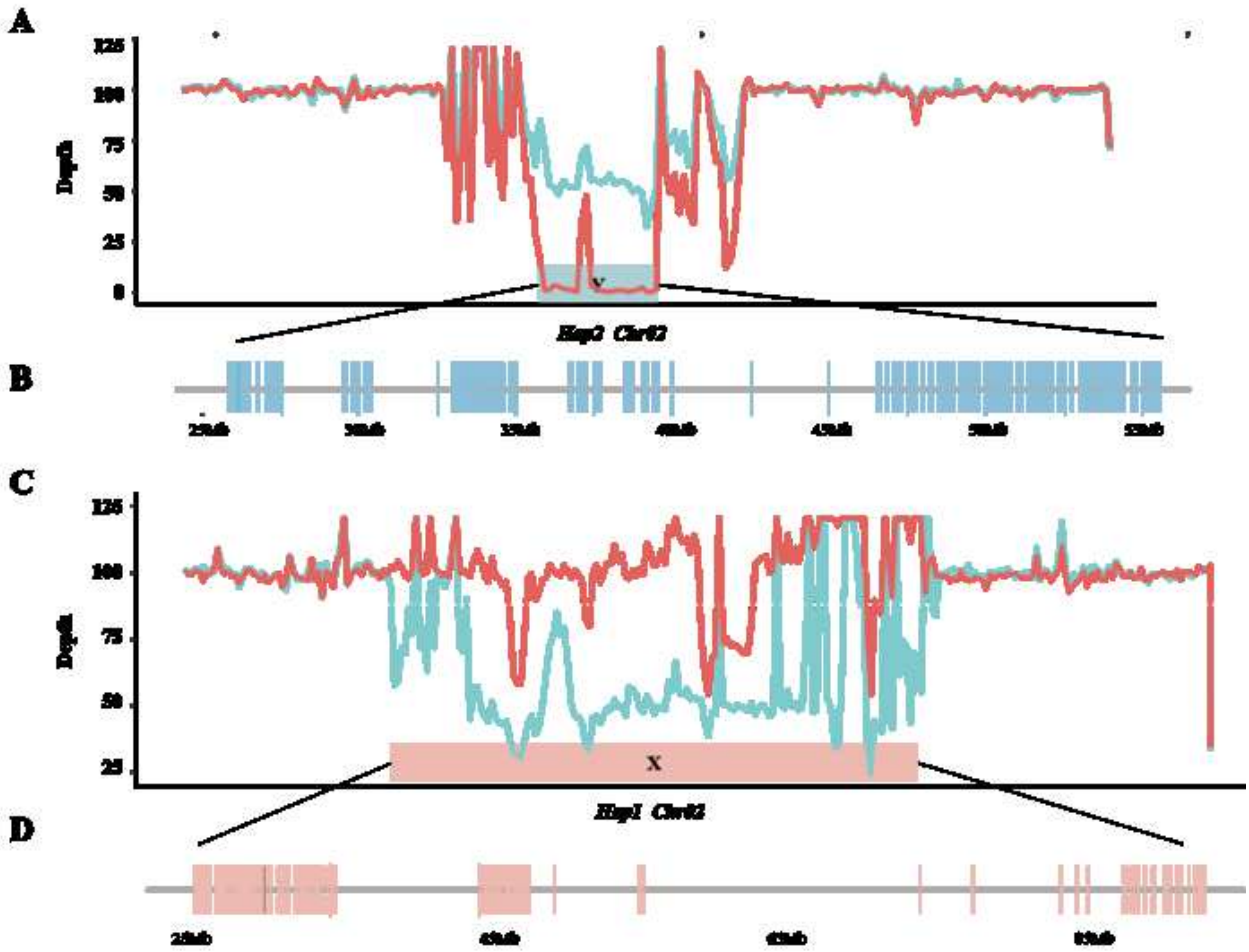

Click here to access/download;Figure;Fig2.docx

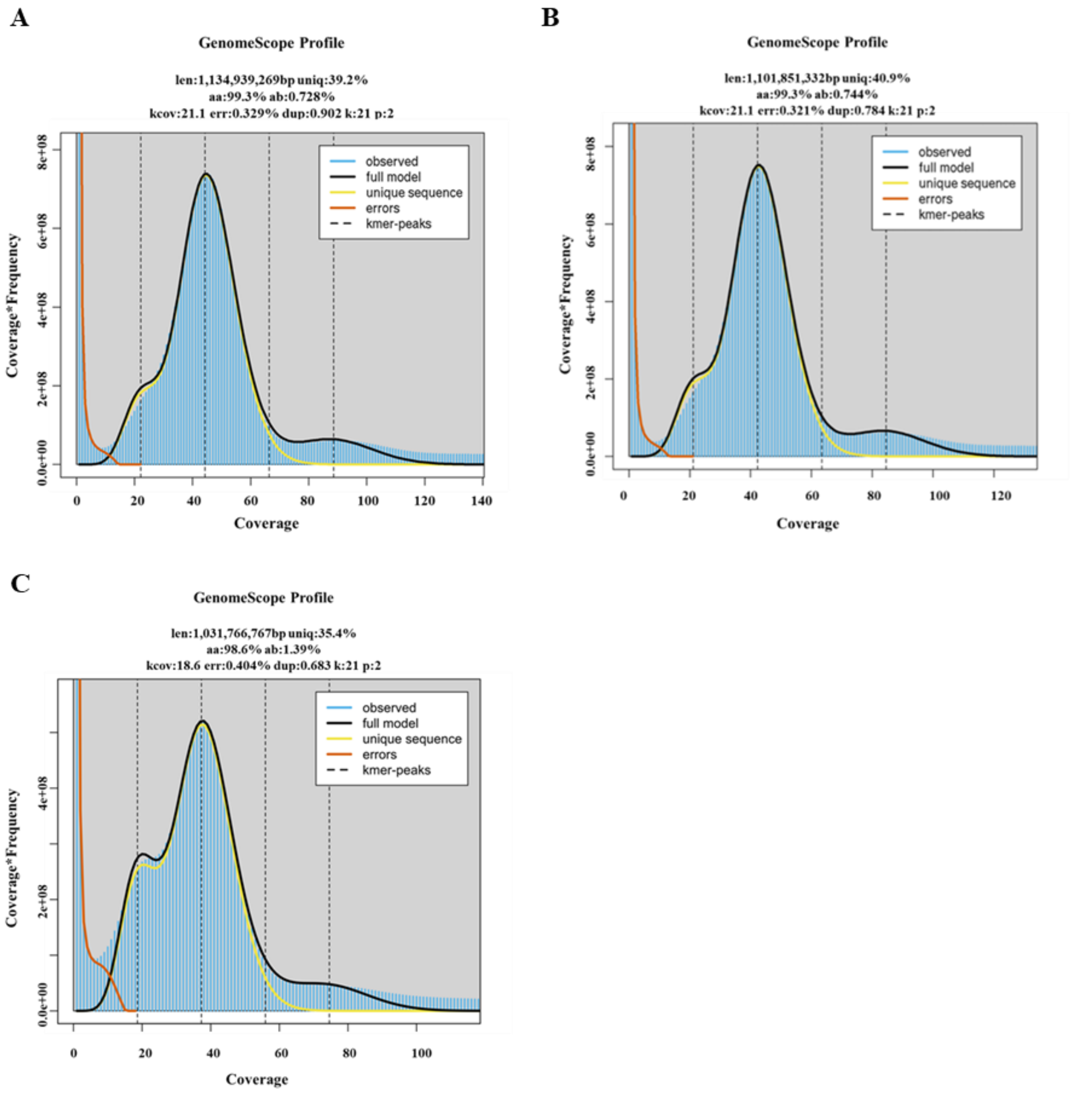

Fig. S1 Distribution of K-mer (K = 21) frequency in sequencing reads of the three seabuckthorn plants. (a) Distribution of K-mer frequency for female *H. salicifolia* from Illumina sequencing. (b) Distribution of K-mer frequency for male *H. salicifolia* from Illumina sequencing. (c) Distribution of K-mer frequency for male *H. gyantsensis* from Illumina sequencing.

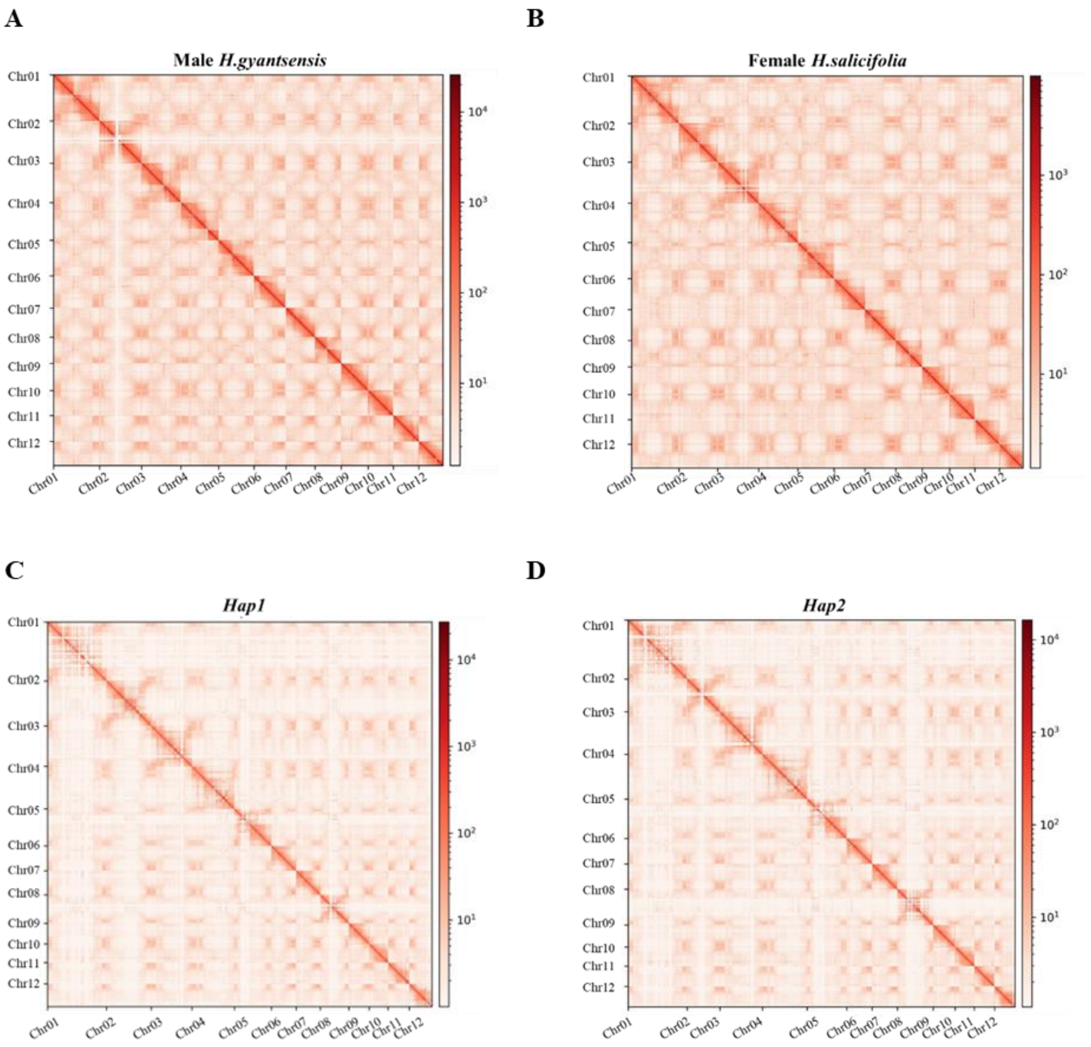

Fig. S2 Hi-C heatmap of chromosomes for male *H. gyantsensis* (a), female *H. salicifolia* (b), Hap1 (c), and Hap2 (d) of male *H. salicifolia*.

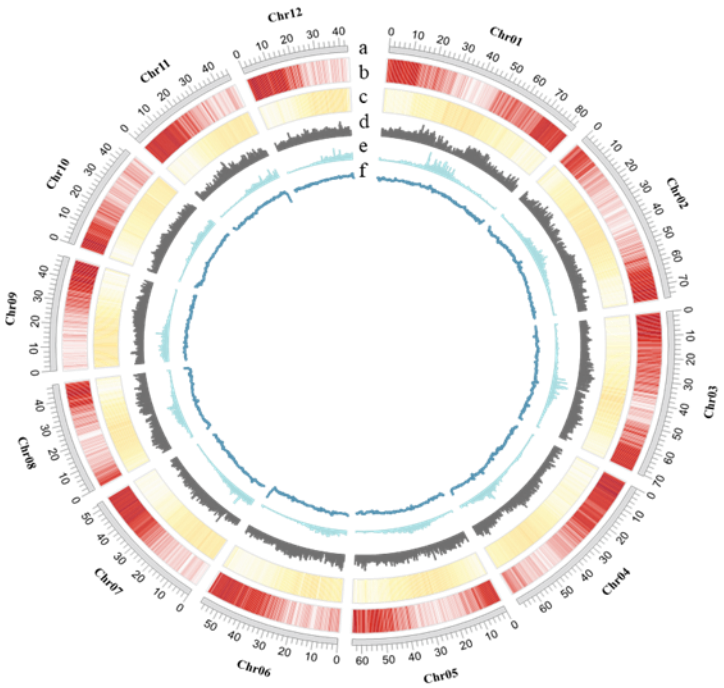

Fig. S3 Circos plot of the genomic landscape of male *H. gyantsensis*. (a) Pseudochromosome. (b) Gene density. (c) Repeat sequences density. (d) Gypsy density. (e) Copia density. (f) GC content.

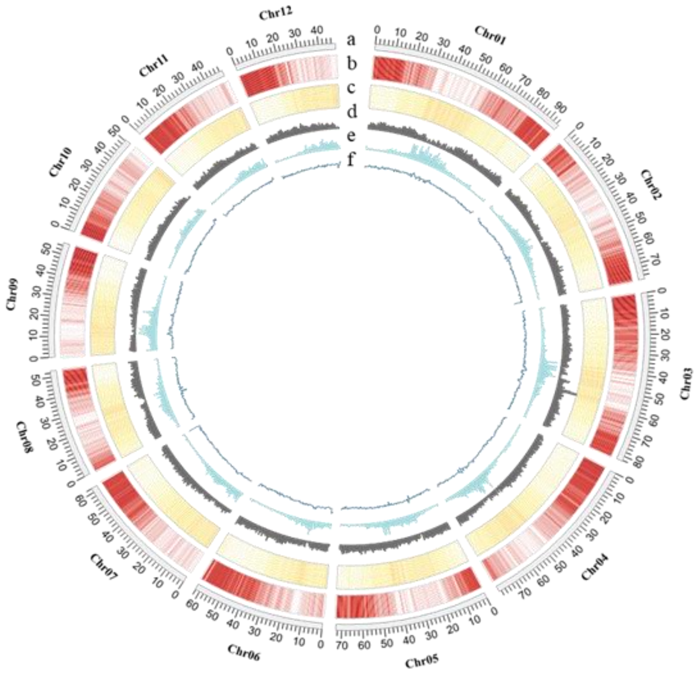

Fig. S4 Circos plot of the genomic landscape of female *H. salifolia*. (a) Pseudochromosome. (b) Gene density. (c) Repeat sequences density. (d) Gypsy density. (e) Copia density. (f) GC content.

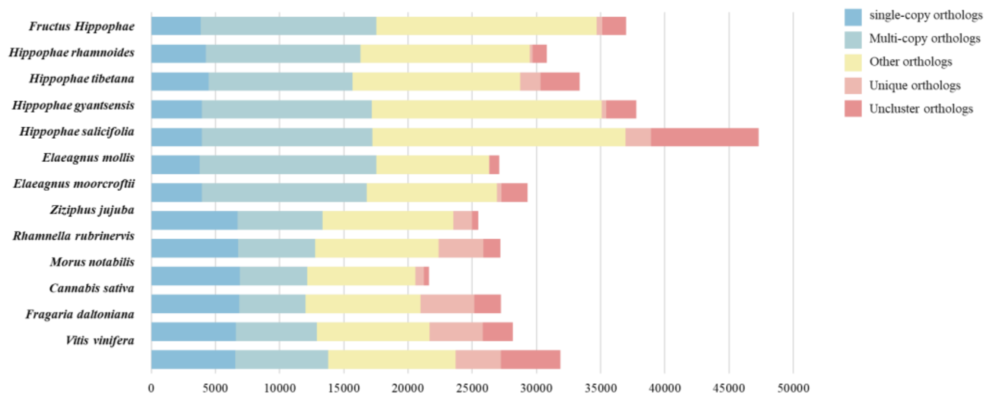

Fig. S5 Statistics of orthogroups in different plants defined by OrthoFinder.

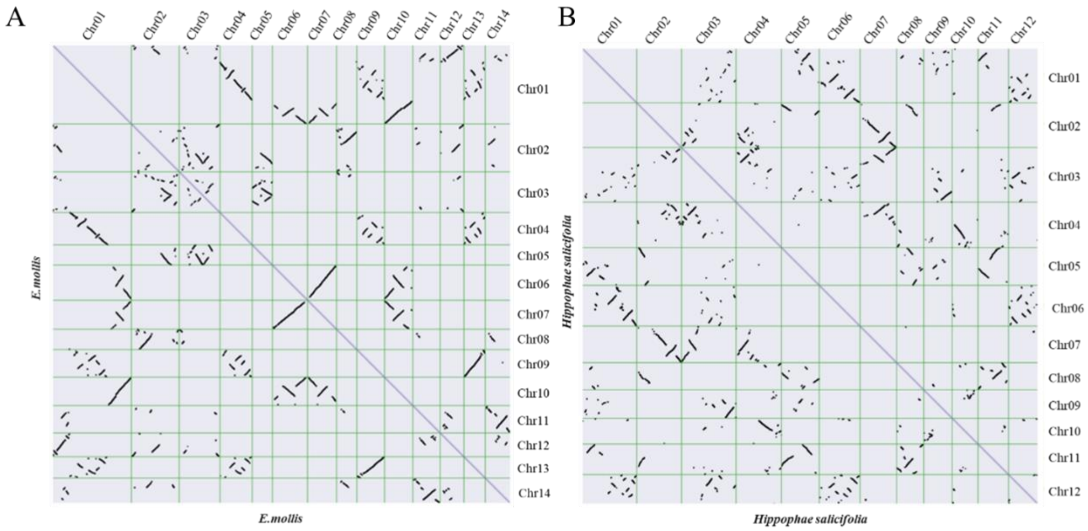

Fig. S6 The collinearity relationship dot plot within the *Elaeagnus mollis* genome and within the *H. salicifolia* genome. (a) *E. mollis* (b) *H. salicifolia*

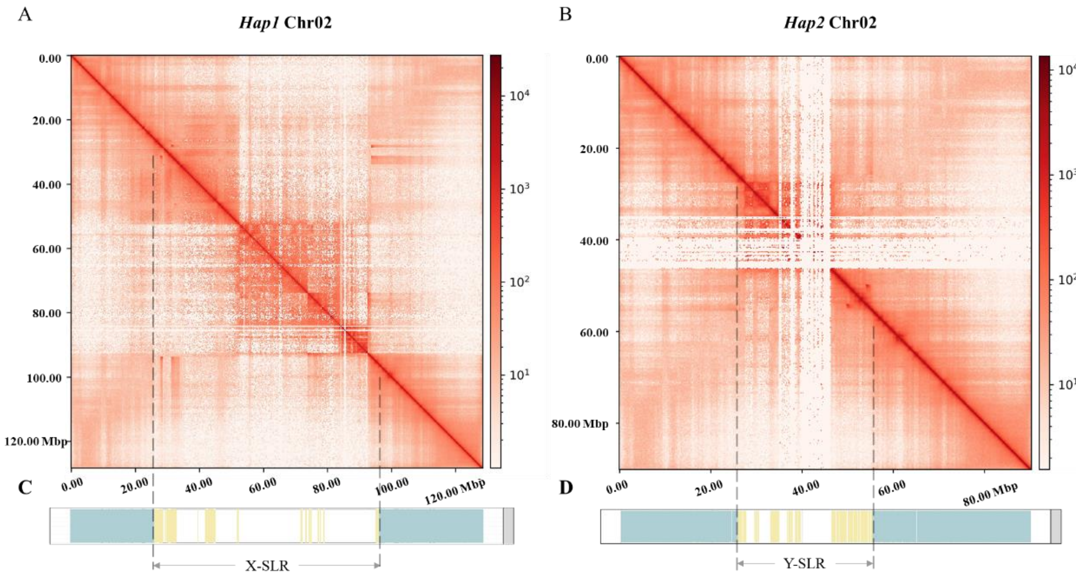

Fig. S7 Hi-C Schematic Diagram of the Sex Chromosomes in *H. salicifolia*. (a) Hap1 Chr02. (b) Hap2 Chr02. (c) the schematic of Chr02 in Hap1, with the yellow region highlighting the X-SLR. (d) the schematic of the sex chromosome in Hap2, where the yellow region denotes the Y-SLR.

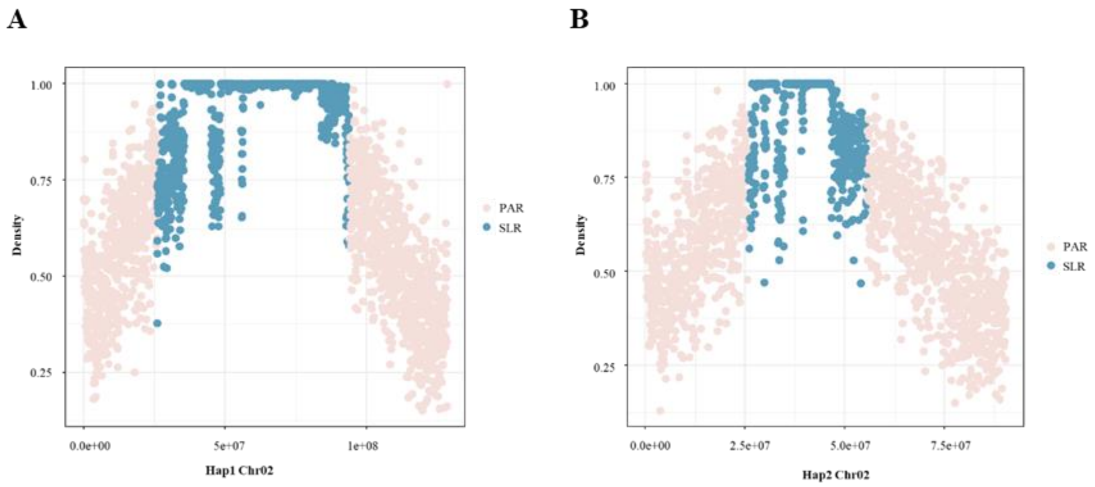

Fig. S8 Density plot of repetitive sequences on Chr02 for Hap1 (a) and Hap2(b). Pink dots represent the density of pseudoautosomal repeats, and blue dots represent the density of repeats in sex-linked regions.

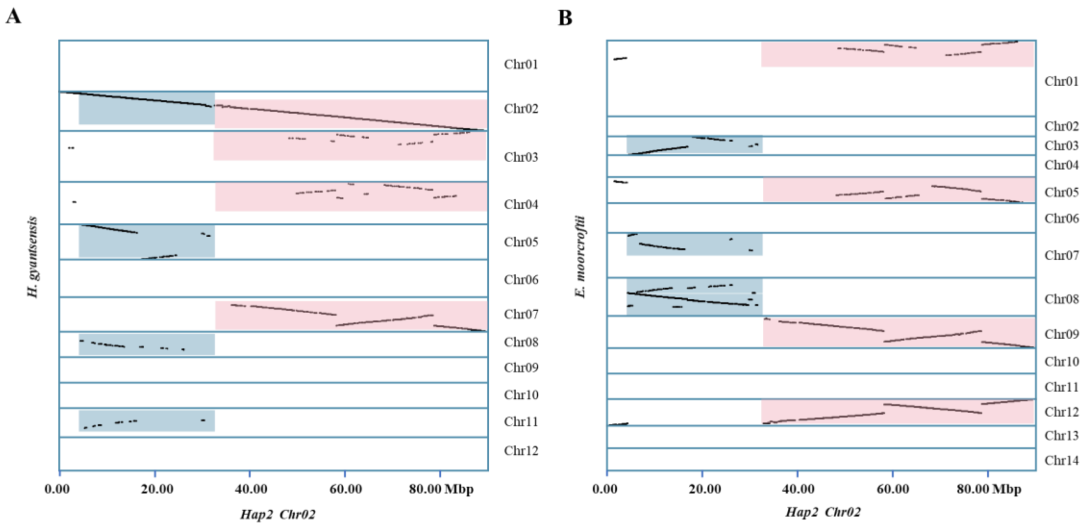

Fig. S9 Synteny relationship between Chr02 of Hap2 and its closely related species: (a) Syntenic point map of Hap2 Chr02 and whole genome of *H. gyantsensis* (b) Syntenic point map of Hap2 Chr02 and whole genome of *E. moorcroftii*.

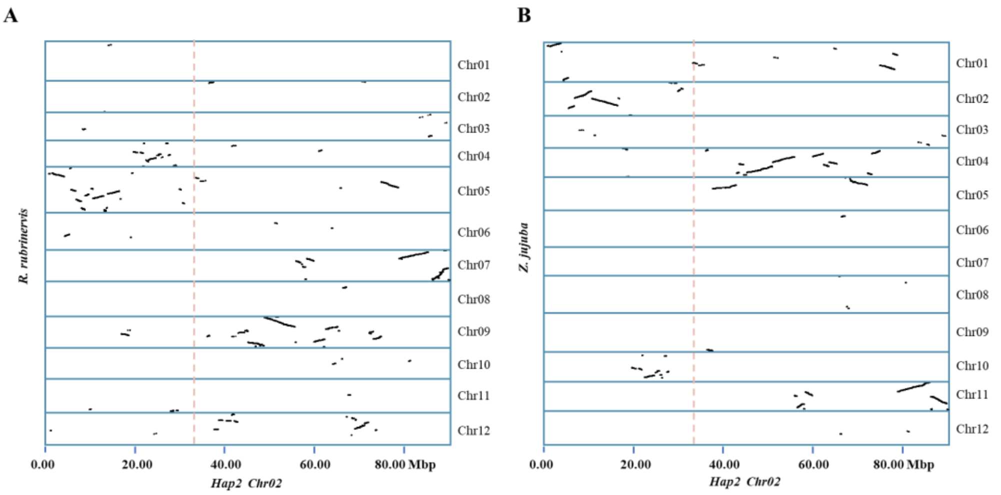

Fig. S10 Synteny relationship between chromosome 2 of Hap2 and its closely related species: (a) Synteny relationship between Chr02 of Hap2 and *Z. jujuba*. (b) Synteny relationship between Chr02 of Hap2 and *R. rubrinervis.*

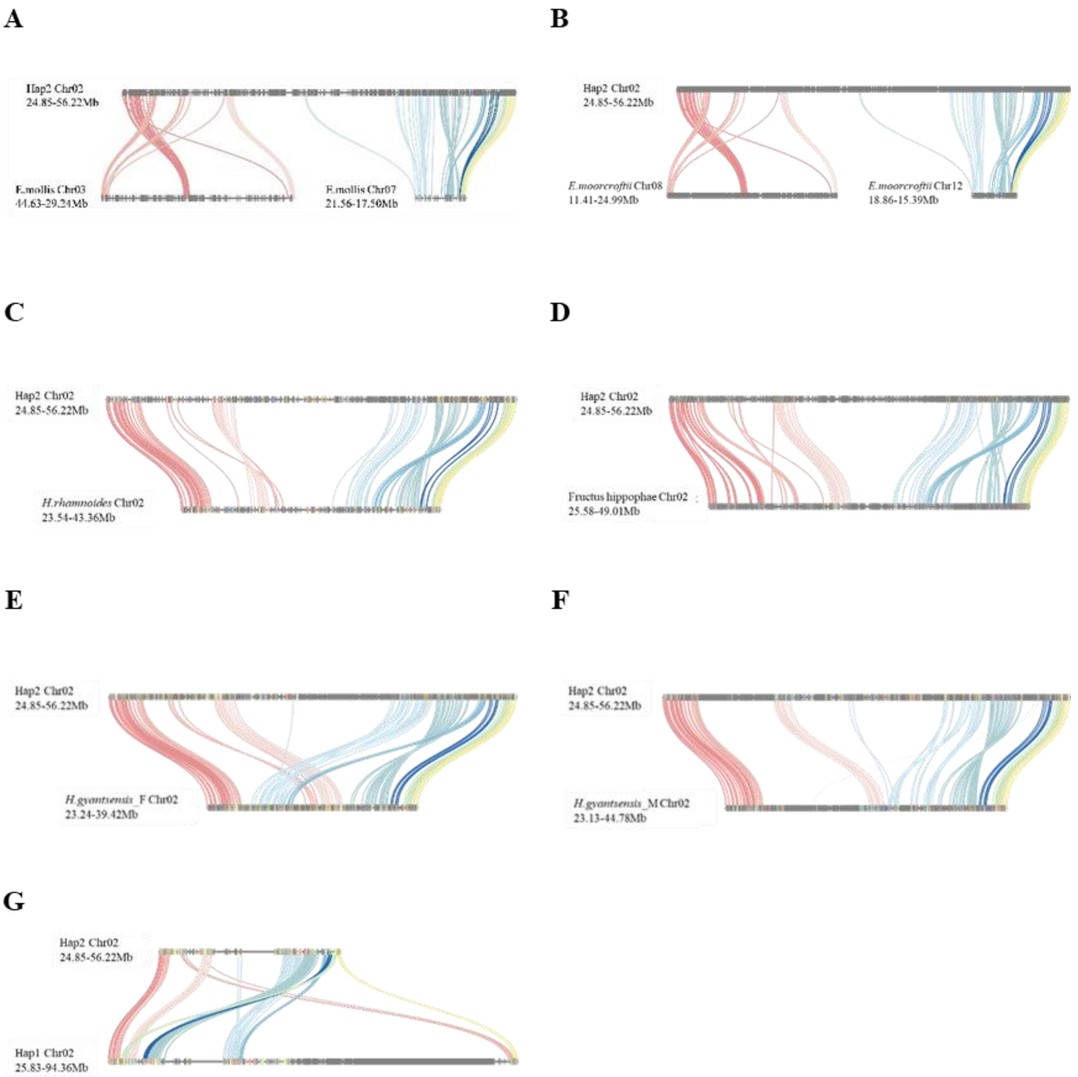

Fig. S11 M Microsynteny of homologous blocks between the Y-SLR of Hap2 and its closely related species. (a) *E. mollis* (b) *E. moorcroftii* (c) *H. rhamnoides* (d) Fructus Hippophae (e) female *H. gyantsensis* (f) male *H. gyantsensis* (g) Hap1.

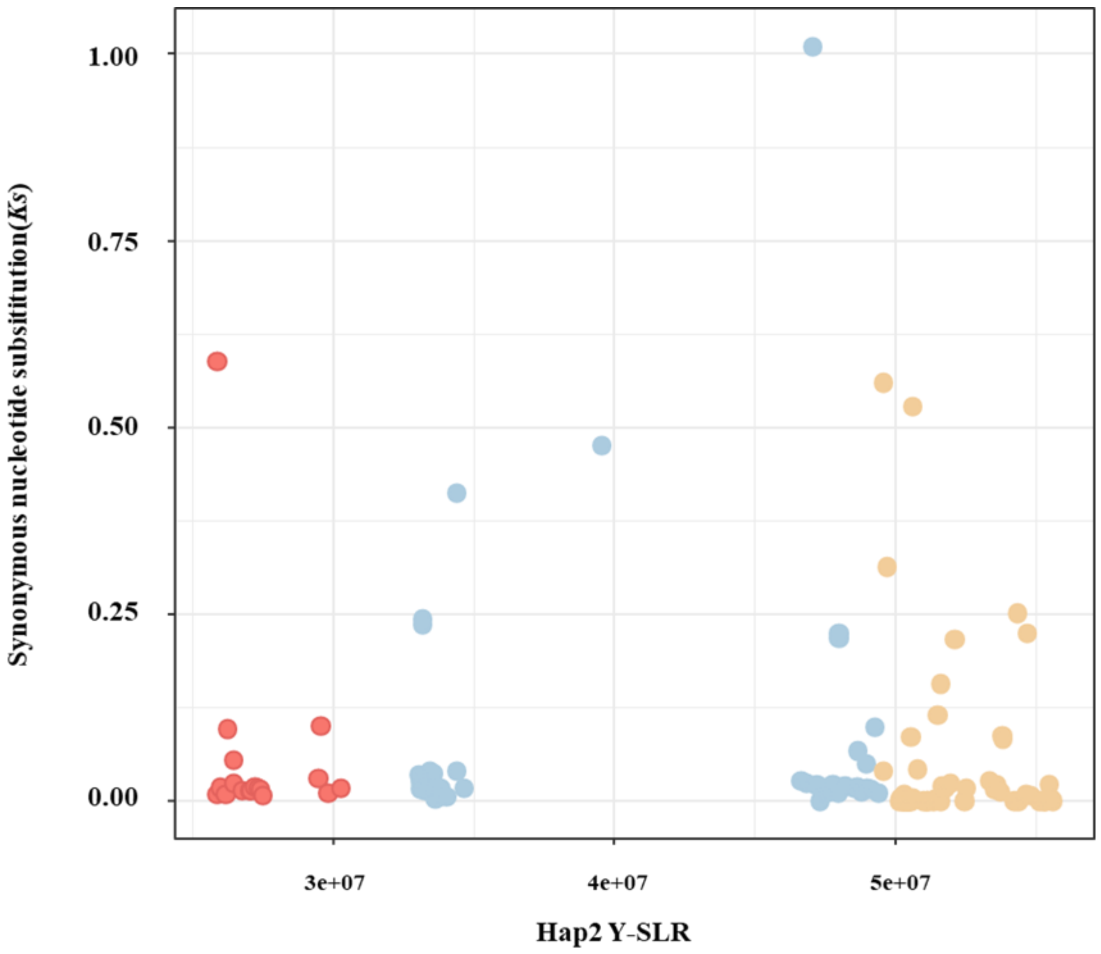

Fig. S12 The Ks values of XY homologous gene pairs within the Y-SLR are mapped to the positional coordinates of the Y chromosome. Blue represents gene pairs within stratum 1, red represents gene pairs within stratum 2-1, and yellow represents gene pairs within stratum 2-2.

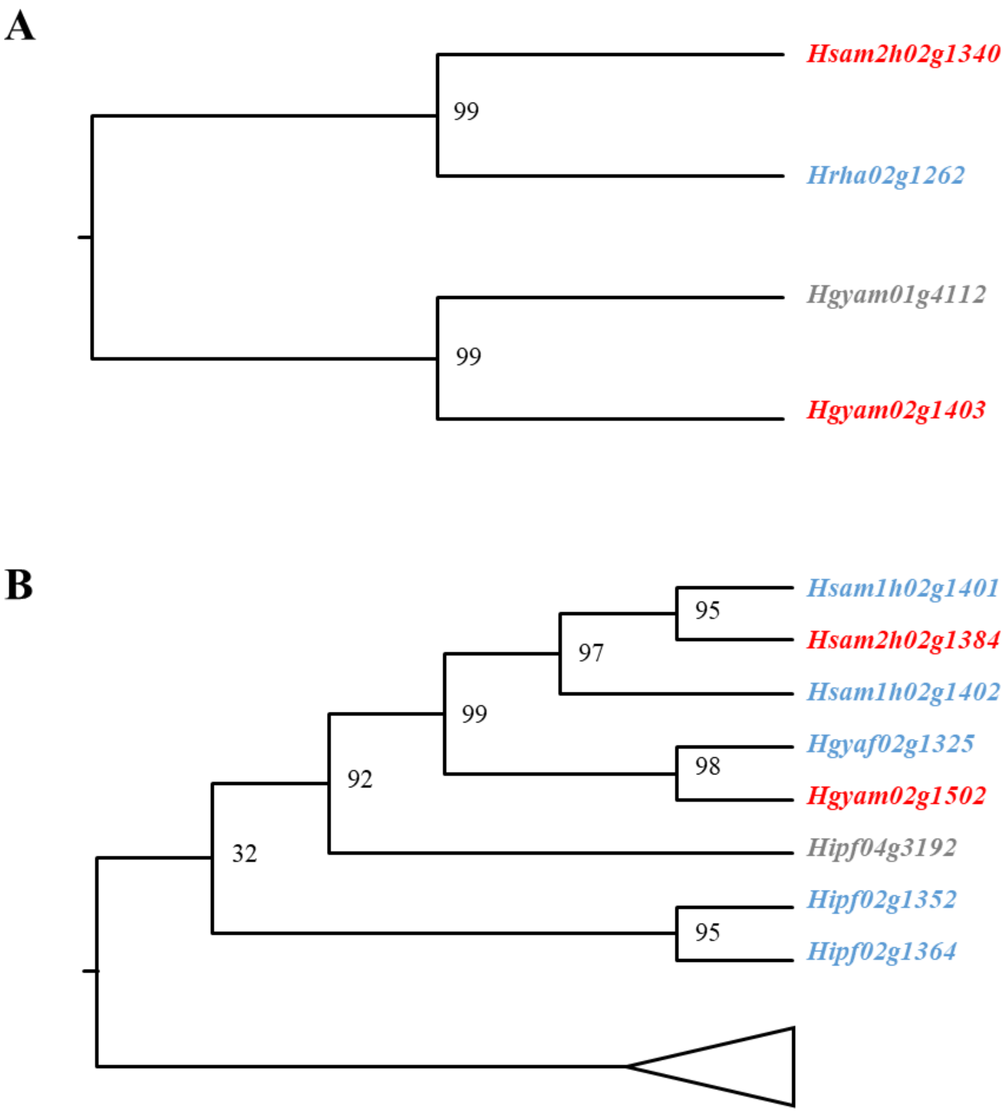

Fig. S13 Among the 11 male-specifically expressed genes, the phylogenetic trees of two genes are not consistent with the expected phylogenetic structure. The genes within the Y-SLR are marked in red, the genes within the X-SLR are marked in blue (a) *Hsam2h02g1340* (b) *Hsam2h02g1384*.

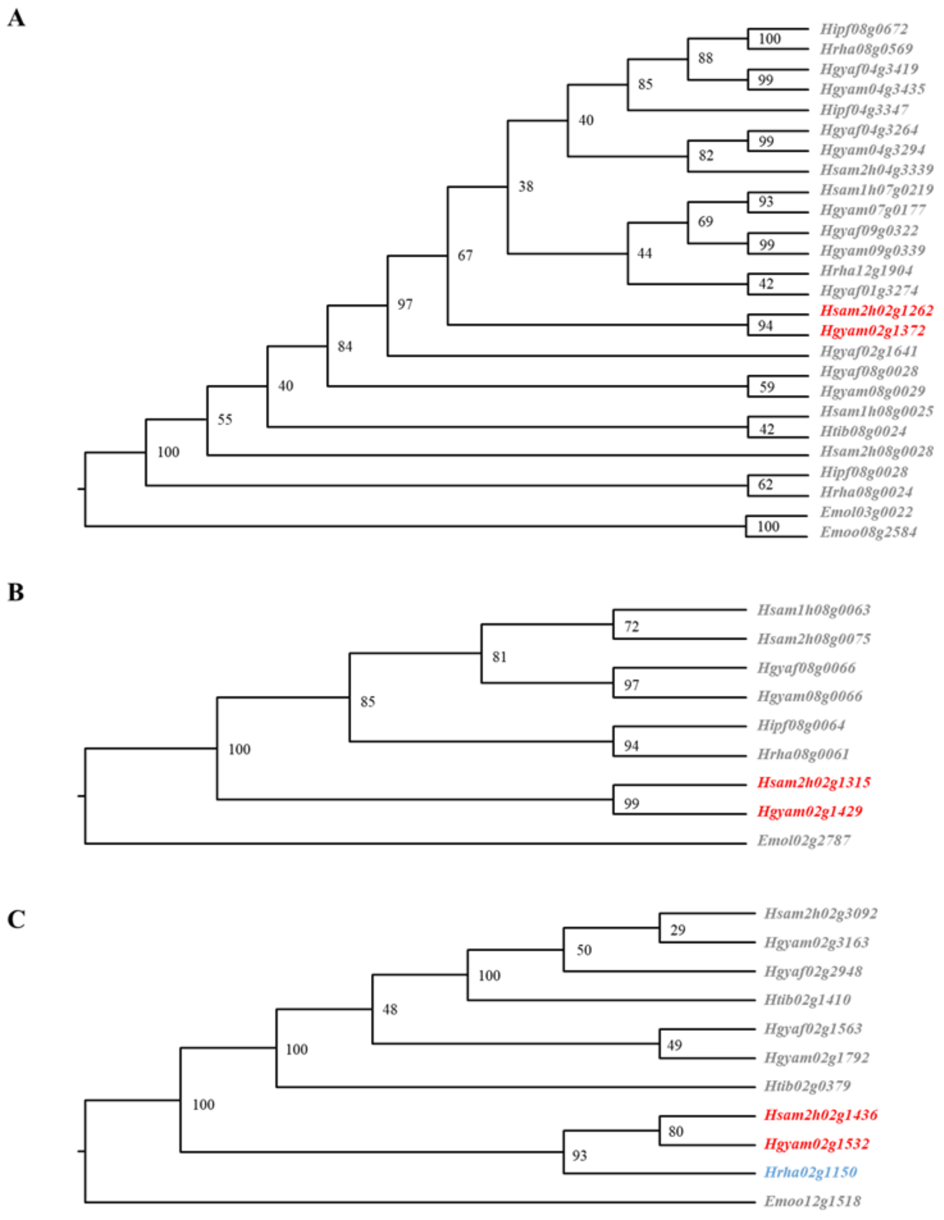

Fig. S14 Among the 11 male-specifically expressed genes, three are male-specific genes that lack corresponding homologous genes within the X-SLR. The genes within the Y-SLR are marked in red, the genes within the X-SLR are marked in blue. (a) *Hsam2h02g1262* (b) *Hsam2h02g1315* (c) *Hsam2h02g1436*.

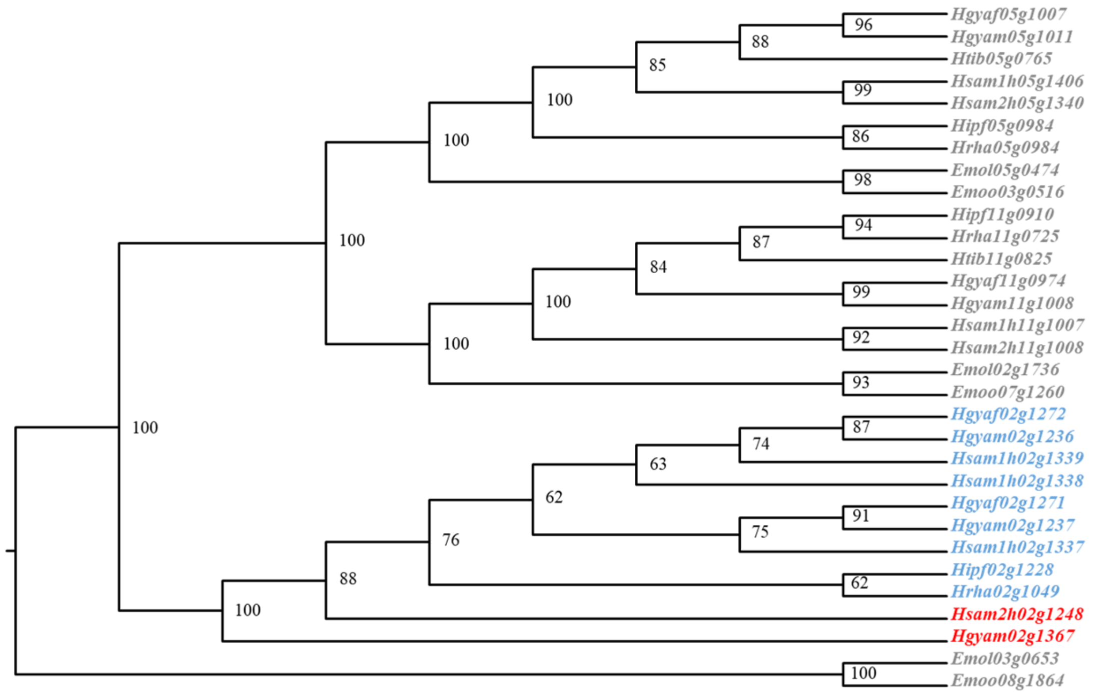

Fig. S15 Gene tree constructed by *Hsam2h02g1248* and its homologous genes. The genes within the Y-SLR are marked in red, the genes within the X-SLR are marked in blue.

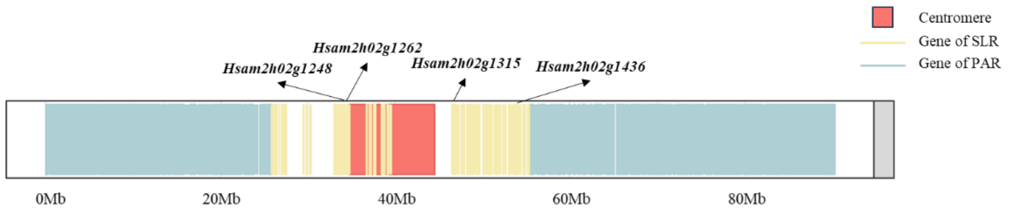

Fig. S16 The distribution of the four candidate genes within the SLR; three genes are located in the presumed ancient strata, one gene (*Hsam02g1436*) is close to the PAR region.

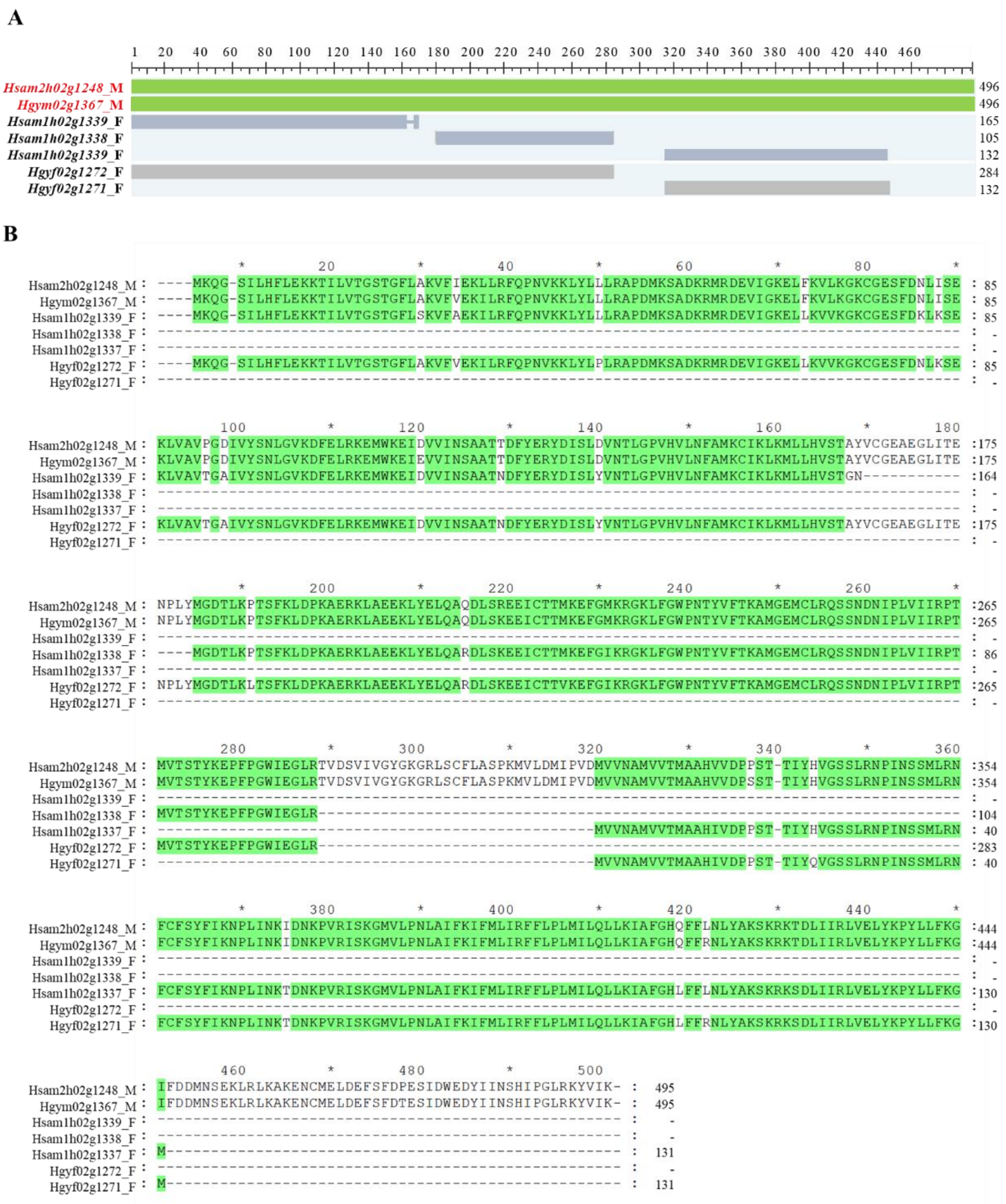

Fig. S17 The sequence alignment of *Hsam2h02g1248* with its homologous genes in the female- and male-specific linkage regions of *H. salicifolia* and *H. gyantsensis* reveals that gene fragmentation has occurred in the homologous genes of females.

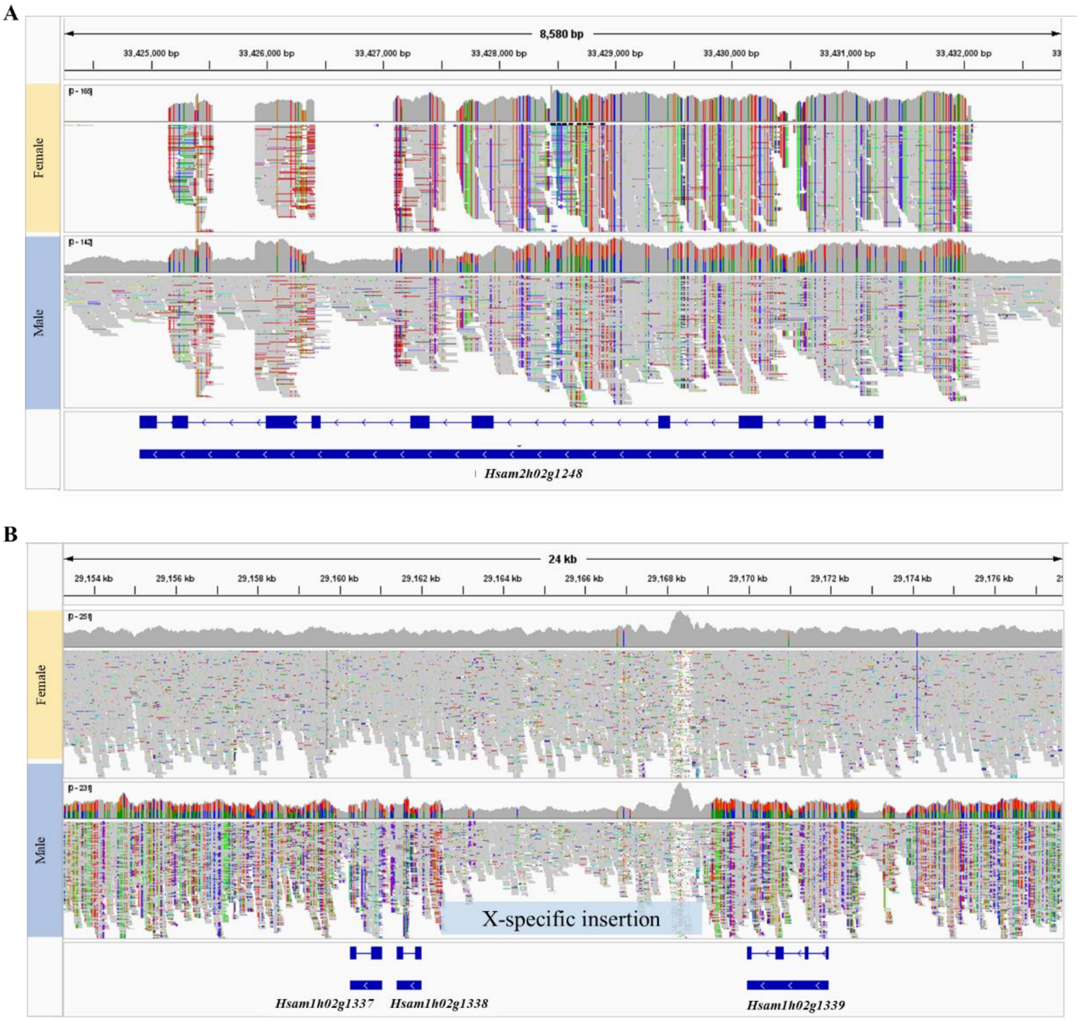

Fig. S18 Alignments of the resequencing reads from male and female individuals to the genes. (a) *Hsam2h02g1248*. (b) The homologous genes of *Hsam2h02g1248* in X-SLR were *Hsam1h02g1337*, *Hsam1h02g1338* and *Hsam1h02g1339*.

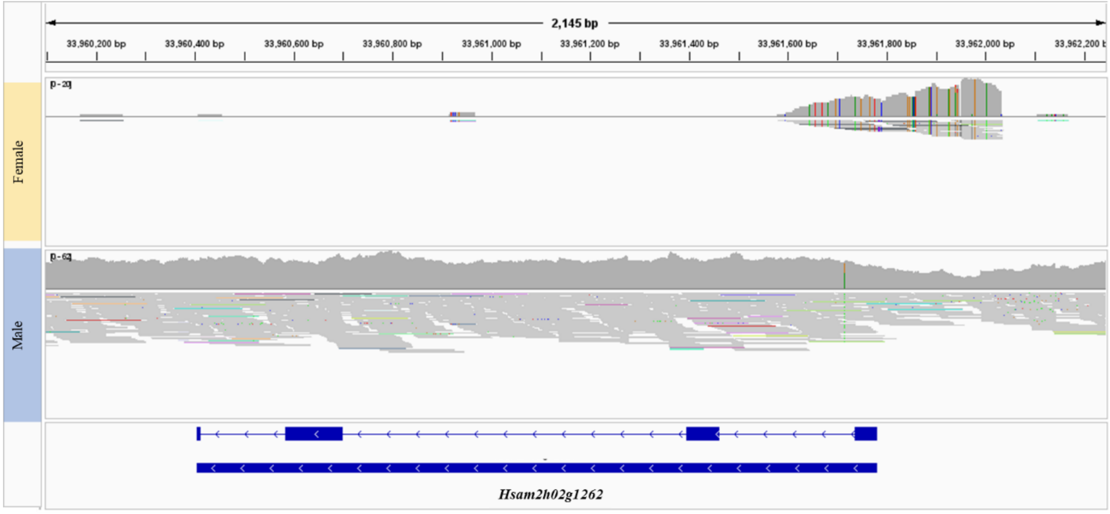

Fig. S19 Alignments of the resequencing reads from male and female individuals to the gene *Hsam2h02g1262*.

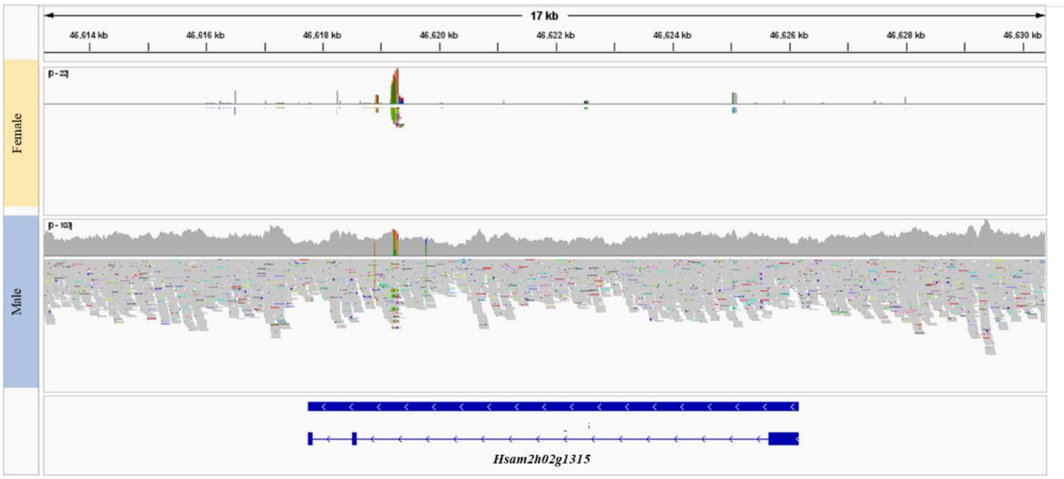

Fig. S20 Alignments of the resequencing reads from male and female individuals to the gene *Hsam2h02g1315*.
