# Supplementary material for: The genome of Hippophae salicifolia provides new insights into the sexual differentiation of sea buckthorn
Source: Gigascience. 2025 Jul 2;14:giaf046. doi: 10.1093/gigascience/giaf046 (PMC12218201; doi:10.1093/gigascience/giaf046)
Supplement: giaf046_Authors_Response_To_Reviewer_Comments_original_submission [file giaf046_authors_response_to_reviewer_comments_original_submission.pdf]

Author's Response To Reviewer Comments

We would like to express our gratitude to the editor for granting us the opportunity to resubmit our paper. We are also deeply appreciative of the constructive suggestions provided by the reviewers, which have been instrumental in enhancing both the language and the depth of our manuscript. Hereby, we submit a revised version of our manuscript titled "The genome of Hippophae salicifolia provides new insights into the sexual differentiation of sea buckthorn" incorporating the modifications as per the recommendations from the editor and reviewers. All changes have been highlighted in red within the revised manuscript.

Reviewer #1: I have read the manuscript titled "The genome of Hippophae salicifolia provides new insights into the sexual differentiation of seabuckthorn" from Chen and colleagues. This work assembled and annotated the genomes of two Hippophae species, with an emphasis on mapping sex-determining regions (SDRs) and comparing sex chromosomes. The research uncovered differences in structural variations between sex chromosomes, and identified candidate genes related to SDR. These findings provide important insights for understanding the evolution of sex determination and will be valuable resources in seabuckthorn. The manuscript is well organized and the most important analyses and information were included, but the authors need to address some issues that are described below.
1. In Figure S2, some blanks were exhibited in HiC cluster, but these areas were not all blank in different assemblies. For example, the blanks in Chr05 and Chr08 were different among female, hap1 and hap2 genomes, why?
Reply:
Thank you very much for raising the question regarding the differences in the HiC clustering gaps observed in Figure S2. The disparities in these gaps between Chr05 and Chr08 are likely attributable to genomic structural variations and haplotype diversity. Similar phenomena have been documented in other species [1,2]. Additionally, the differences observed between the female genome and hap1/hap2 may primarily stem from variations in sequencing and assembly strategies. We are grateful to the reviewer for highlighting this important issue and hope that the above explanation addresses your concerns.
[1] Cheng, S., Zhang, Q., Geng, X. et al. Haplotype-resolved chromosome-level genome assembly of Ehretia macrophylla. Sci Data 11, 589 (2024). https://doi.org/10.1038/s41597-024-03431-9
[2] Oriowo T. O., Chrysostomakis I., Martin S. et al. A chromosome-level, haplotype-resolved genome assembly and annotation for the Eurasian minnow (Leuciscidae: Phoxinus phoxinus) provide evidence of haplotype diversity. Gigascience. Jan 6;14:giae116. (2025). https://doi.org/10.1093/gigascience/giae116.

2. Page 8, Hap1 and Hap2 of male H. salicifolia genomes were 309.06-351.7 Mb larger than those of females by about 40%. How to explain these significant differences?

Reply:
We appreciate your inquiry. The disparity in genome size between those assembled using Oxford Nanopore Technologies (ONT) sequencing and those assembled using HiFi sequencing is primarily attributable to the distinct sequencing and assembly strategies employed. For instance, the recently published genomes of Hippophae tibetane exhibit significant size differences [1, 2]. Should further analysis or clarification be required, please do not hesitate to contact us.
[1] Wang R, Wu B, Jian J, et al. How to survive in the world's third poplar: Insights from the genome of the highest altitude woody plant, Hippophae tibetana (Elaeagnaceae). Front Plant Sci. 2022;13:1051587. doi:10.3389/fpls.2022.1051587.
[2] Zhang G, Song Y, Chen N, et al. Chromosome-level genome assembly of Hippophae tibetana provides insights into high-altitude adaptation and flavonoid biosynthesis. BMC Biol. 2024; 22: 82. https://doi.org/10.1186/s12915-024-01875-4

3. Page 9, why is the BUSCO completeness rates scores of genomes (97.4%, 97.6%, 97.6%, 97.7%) less than those of gene set (98.9%, 98.8%, 98.9%, 98.7%)?
Reply:
Thank you for your question. To address this issue, we have reviewed relevant literature and searched for

the criteria used in BUSCO scoring. Based on our findings, we believe that the slightly higher BUSCO score for the gene set compared to the genome assembly is attributable to several factors. First, the objects and methods of evaluation differ between the two, making discrepancies quite normal. Additionally, similar observations, where the gene set BUSCO score exceeds that of the genome assembly, have been reported in other genomic studies[1,2].

[1] Yang T, Cai Y, Huang T, Yang D, Yang X, Yin X, Zhang C, Yang Y, Yang Y. A telomere-to-telomere gap-free reference genome assembly of avocado provides useful resources for identifying genes related to fatty acid biosynthesis and disease resistance. Hortic Res. 2024 Apr 22;11(7):uhae119. doi: 10.1093/hr/uhae119.

[2] Wang Y, Zhang RG, Hörandl E, Zhang ZX, Charlesworth D, He L. Evolution of Sex-linked Genes and the Role of Pericentromeric Regions in Sex Chromosomes: Insights from Diploid Willows. Mol Biol Evol. 2024 Nov 1;41(11):msae235. doi: 10.1093/molbev/msae235.

4. Page 10, "Synteny analysis revealed one-to-one homology relationships … a higher frequency of chromosomal rearrangements."
This part of the results were not presented in any graphical or tabular form and need to supplemented.
Reply:
We are grateful to the reviewer for the meticulous review and valuable comments. The additional content has been incorporated into the revised manuscript, specifically on line 1 of page 11 and in Supplementary Figure 6.

5. Page 11, "Applying the same methodology to H. gyantsensis, … 33.86 Mb to 44.78 Mb and contained 235 genes."
The final SLR regions in H. gyantsensis was 12.31 Mb from 14.59 - 15.97 Mb and 33.86 - 44.78 Mb, but the depths in 14.59 - 15.97 Mb and 39.47 - 44.78 Mb do not differ between male and female as those in the H. salicifolia genome. In the presence of species difference, why refine the SLR with collinearity when you have precise results for this analysis?
Reply:
We appreciate the insightful comments from the reviewer. Since the male genome data of Hippophae gyantsensis was not phased, its Chr02 contains sequences from both the X and Y chromosomes. For regions with significant differences between the X and Y chromosomes, we can accurately pinpoint these areas using the coverage depth differences in resequencing data. However, for sequences that are unique to the X and Y chromosomes but show subtle differences, there may be instances of merged assembly. Therefore, we employed the two aforementioned methods to localize the Y-SLR in H. gyantsensis. Nevertheless, we did not account for the potential impact of interspecies differences. To avoid any potential misinterpretation, we have decided not to specifically localize the Y-SLR interval in H.gyantsensis, but rather to use its genomic data as a supplementary tool for subsequent analyses.

6. Page 13, "These observations indicate a lineage-specific chromosomal fusion event in seabuckthorn, and Chr02 was possibly derived from the merging of two ancestral chromosomes."
Even if previous studies suggest that dioecy may be a newer evolutionary state, we can't identify directly from the phylogenetic tree either Hippophae or Elaeagnus was closer to the ancestor. Without a covariance comparison between the common ancestor and these two clades, I think the current results may only indicate a break or fusion occurred in Chr02. Other species that diverged earlier in the Hippophae or Elaeagnus should be added for collinearity comparison and illustration if retaining the existing conclusions.
Reply：
We sincerely thank the reviewer for their valuable suggestion. In response to your advice, we selected species from the Rhamnaceae family, a group closely related to Elaeagnaceae, as outgroups for the collinearity analysis. Specifically, we used Ziziphus jujuba and Rhamnella rubrinervis to investigate whether a fusion event occurred on Chr02. The results of this analysis are presented in Fig. S10, and a detailed description has been added on page 13 of the revised manuscript. This approach has provided further insights into the evolutionary dynamics of Chr02, and we believe it significantly strengthens our findings. Please let us know if additional clarifications or analyses are needed. We greatly appreciate your constructive feedback.

7. Page 13, "The collinear regions were categorized into nine distinct blocks through comparison of

chromosomal segment synteny (Supplementary Fig. S10 and Table S9)."
There are 10 blocks from A to H in the Supplementary Table S9, why was H not used together in the analysis?
Reply:
Thank you very much for pointing out this issue; it was an oversight on my part. The SLR only includes 9 blocks because Block H is not within the SLR range we previously determined. I have removed the content of Block H from Supplementary Table 9 and have provided an explanation for Block H in the Fig S11.

8. Page 13, "Block D had the highest Ks value (Ks=0.1266), … and block G (Ks=0.0037)."
It seems that the mean Ks value of the gene pairs was used as the result, but it was not mentioned here or in the method. It should be clarified.
Reply：
We sincerely thank the reviewer for their valuable comment and apologize for the oversight. In response to your suggestion, we have added a detailed description of the method used to calculate the average KS values for different genomic blocks in the Methods section. This addition can be found on line 18-20 of page 28 of the revised manuscript.

9. Page 14, "Stratum 1 (including blocks C and D) was located …. Stratum 2-1 (including blocks A and B) was located …. Stratum 2-2 (including blocks E, F, and G) was located … with an average Ks value of 0.0418 (Fig 4c)."
From the Supplementary Table S10, we can see that the mean Ks value of block C was significant different from that of block D, and they were also relatively far apart from each other in chromosome, but why was it divided into the same group?
Reply：
Thank you for your feedback. I apologize for the lack of clarity in my original description regarding the stratification of genomic layers. I have revised the content, which can be found in the red text on pages 15 and 16. Our stratification is based on the Ks values and the topological structure of the homologous gene trees. In the revised version, we have refined our description to better articulate this approach.
Furthermore, I provide additional rationale for this stratification below:
Combining the results of Supplementary Figure 11 and the previous collinearity analysis, we speculate that regions C and D were likely distributed on two separate chromosomes in an early ancestral form, and a lineage-specific chromosomal fusion event brought them into proximity. Numerous studies have shown that during chromosomal evolution, sex-determining regions tend to accumulate a large number of repetitive sequences. The interval between regions C and D has similarly accumulated a substantial amount of repetitive sequences, which likely accounts for the greater physical distance between C and D due to transposable element (TE) insertions.
Secondly, more than half of the genes in these two regions exhibit the topological structure characteristic of ancient strata (as shown in Structure 1 in Figure 4d), suggesting that both regions are inclined to belong to the older strata. Additionally, region D shows structural variations between the X and Y chromosomes. Structural variations can exacerbate the differentiation of homologous genes on the X and Y chromosomes, leading to higher Ks values. Therefore, the higher Ks value of region D compared to region C can be attributed to these structural variations.
Based on these considerations, we are inclined to classify regions C and D as Stratum 1.
If you require further clarification or additional details, please do not hesitate to contact us.

10. Page 14, "Distinct phylogenetic patterns were observed … tended to cluster together." This point needs to be supported by references.
Reply:
We thank the reviewer for pointing out the need for supporting references. We have revised the sentence to include relevant citations that support the observed phylogenetic patterns and clustering tendencies.

11. Page 14, "In Stratum 1, an ancient origin was observed for 16 out of 34 single-copy genes;"
There were 17 Structure1 trees in Stratum 1 which was showed in Fig 4d, please check this result.
Reply:

We sincerely appreciate the reviewer's careful attention to this detail. Upon re-examining the phylogenetic tree results, we have confirmed that the correct number is indeed 17. We have carefully revised the text to reflect this correction and ensure accuracy.

12. Page 15, "We utilized transcriptome data, compared these data with the Y-SLR of H. salicifolia, and calculated the transcripts per million (TPM) values of the transcripts (Supplementary Table S15)." "Supplementary Table S15" should be amended to "Supplementary Table S12". Additionally, there was no stem sampling and biological replicates of three tissues mentioned in METHOD.
Reply:
We sincerely apologize for the oversight and thank the reviewer for pointing out this error. Due to the addition of a new figure, we have changed "Supplementary Table S15" to "Supplementary Table S13" in the revised manuscript. We have also added the description of stem sampling and the use of biological replicates for three tissues in the METHOD section on pages 24 and 32.

13. Page 19, "however, the results of our study indicate that X-linked structural variations might be selected and fixed to suppress recombination between XY chromosomes."
It seems that I don't find relevant results, where was this conclusion being drawn from?
Reply:
Based on the comprehensive analysis of Fig. 4b, we have formulated a scientifically grounded hypothesis regarding the observed genomic variations. Our investigation reveals that the X-SLR region exhibits a significantly higher frequency of structural variations compared to its Y-SLR counterpart. These structural alterations demonstrate a potential correlation with our proposed stratigraphic formation patterns.
The integration of recent groundbreaking research on Silene latifolia provides compelling evidence that structural variations within the X-SLR region may facilitate recombination suppression between sex chromosomes. This empirical evidence strongly supports our hypothesis that X-linked structural variations might undergo selective pressure and subsequent fixation to establish recombination suppression mechanisms between XY chromosomes. To more clearly articulate our rationale, we have added content to lines 4–8 on page 14 and lines 14–20 on page 16.

Reviewer #2: This manuscript does an excellent job describing the sex-linked region of seabuckthorn. Overall, the introduction provides good context for why this analysis is needed, and the results are logically ordered with most of their hypotheses supported directly with their data. The identification of candidate genes with orthologs in Arabidopsis is a particularly exciting result. Even though no molecular characterization is performed, that is certainly beyond the scope of this manuscript. It is a complete genome assembly and analysis manuscript. I do have a number of minor comments where I believe further clarification is required, but overall kudos to the authors.

1. - "increase number of acquired genes in X SLR"
- page 12, how can you separate that from the hypothesis of an increased loss in Y instead of insertions in X?
- a better explanation of what "ancestral" and "acquired" genes are is required
Reply:
Thank you very much for your suggestions. We apologize for the lack of clarity in the description of the methodology in the original manuscript. In the revised version, we have provided a more detailed explanation in the Methods section on page 30. Additionally, I would like to elaborate further here:
Given that Hippophae and Elaeagnus both belong to the Elaeagnaceae family and diverged after sharing two whole-genome duplication (WGD) events, we defined genes within the SLR that have homologs in the corresponding homologous segments of both Hippophae and Elaeagnus as “ancestral” genes. For instance, based on the results shown in Supplementary Figure 8, if a gene within the SLR has a homolog located in the designated blocks, it is highly likely to be an “ancestral” gene, as these genes have undergone the WGD events prior to the divergence of the Hippophae and Elaeagnus genera.
In contrast, genes within the SLR that have homologs in other chromosomal regions are classified as “acquired” genes. These genes are likely derived from duplications originating from other chromosomes. While there are certainly factors that could influence the results, the significant difference in the number of

“acquired” genes between the X- and Y-SLRs supports the notion that gene acquisition occurred in the X-SLR rather than gene degeneration in the Y-SLR.

2. - what was the sampling of Male / Female? Could differences be variation within the species instead of just between male / female?
Reply:
We thank the reviewer for raising this important question. To elucidate this issue, we conducted second-generation sequencing on pooled samples of fourteen female and fourteen male Hippophae salicifolia. The sequencing data were aligned to chromosome 2, and the results revealed that the differences observed between the X- and Y-SLRs are indeed due to divergence between sex chromosomes rather than species variation, with no changes occurring in the respective regions. The data have been updated accordingly. For details, please refer to Figure 3, lines 4–7 on page 11, and the Materials and Methods section.

3. - Throughout, some genus and species names are not italicized
Reply：
Thank you for pointing out this formatting issue. We have made the necessary revisions and have double-checked to ensure consistency in formatting throughout the manuscript.

4. last paragraph of the introduction:
"The genetic basis of sexual differentiation in seabuckthorn has been a major focus of
studies of plant reproductive biology and sexual evolution"
- Please support this statement with citations
Reply:
We apologize for the oversight. Upon reviewing the text, we identified an inappropriate description and have since revised it accordingly.

5. You should replace all references of "Gypsy" elements to "Ty3"
- https://doi.org/10.31219/osf.io/fma57
Reply:
Thank you very much for your suggestions. I have revised the relevant content accordingly.

6. Figure 4A:
- The units for the x-axis need labeled at least in the figure legend
Reply:
Thank you very much for pointing out this oversight. I sincerely apologize for this omission and have now included the x-axis unit information in the revised manuscript.

7. Figure 5:
- A legend for the heatmap is required, it is ambiguous which colors are high or low expression
Reply:
Thank you for your valuable suggestions. I fully concur with your perspective that the absence of a heatmap legend can indeed impede the interpretation of the data. Following your advice, I have supplemented the revised manuscript with a complete heatmap legend, clearly indicating the correspondence between colors and gene expression levels (red representing high expression and blue representing low expression).

8. Page 15:
". Sex-determining genes often share phylogenetic similarities with ancient strata genes, and homologous genes from the same gametophyte tended to cluster together"
- this statement needs a citation or further justification as to why certain gene tree topologies are expected
Reply:
Thank you very much for raising the issue. I have now cited the relevant literature accordingly.

9. Page 16:
"Additionally, the pseudogenization of Hsam2h02g1248 was observed in the female counterparts of both H.

gyantsensis and H. salicifolia, which resulted in the loss of its original function"
- Amino acid alignments are shown in Figure S15, but an additional check of genome sequences is required to ensure true psuedogenization instead of a misannotation of these loci in females
Reply:
We sincerely appreciate your valuable suggestions. Upon further analysis of the genomic sequence, we identified a large fragment insertion that resulted in the disruption of the homologous gene of Hsam2h02g1248 within the X-SLR region. The specific modifications and detailed findings can be found in revised manuscript version 19, as well as in Supplementary Tables 17 and 18.

10. Discussion:
- mentioning the WGD history of these lineages should be mentioned in the introduction. It makes figure 4A make a lot more sense so mentioning it when describing figure 4A may also contextualize the multiple observed syntenic hits
- this makes Figure 4A even more convincing, there are 4 copies of L and R regions, and only a single pair exist on the same chromosome!
Reply：
We extend our gratitude for your insightful suggestions. In response, we have incorporated the recommended content into both the introduction and the description of Figure 4A. The specific revisions can be found in the red-font section on page 7 and within lines 7–15 on page 14 of the revised manuscript.

11. - "The SLRs of papaya and kiwifruit have evolved from the pericentromeric regions [38-40]."
- this sentence seems out of place, maybe:
'sex chromosomes are known to have independently evolved through similar mechanisms in other lineages such as...'
Reply:
Thank you for your valuable suggestions. We have revised the inappropriate descriptions accordingly, and the changes are highlighted in red on page 20 of the revised manuscript.

12. Figure S2:
- The hi-c maps provided are not high enough resolution to evaluate the accuracy of the SLR. It would be helpful to provide an enlarged contact matrix just for chromosome 2 to increase confidence in the order and orientation of the SLR. Alternatively, please provide a statement in the part discussing Figure 4B that the observed lack of synteny may be observed due to misassembly in either the SLR in this manuscript, or in the genomes compared in this manuscript.
- HiFi genome assemblies are incredibly high quality and I do not doubt the author's expertise in carefully evaluating the accuracy of their genome assembly. I'm just a stern believer that genome assemblies are hypotheses and not ground truth; a point readers, especially non-specialists, often miss.
Reply:
Thank you very much for your suggestion. The Hi-C map of chromosome 2 has been added to Supplementary Figure 7. If you require further explanation or additional details, please do not hesitate to contact us, and we will provide more information.

13. Figure S12A:
- the number of tips on the cladogram and labels do not match.
- additionally, I'm not sure how this supports the author's hypotheses as they do not explain sufficiently what an expected topology is, or why it is expected. Bootstrap values and details on phylogenetic reconstruction are not easy to find.
- It also needs mentioned why a gene tree might deviate from expectation. When compared a set of orthologues, minute differences can change gene tree topology relative to the species tree. Support values for each node should be provided as well.
- I really enjoy most of the analyses set forth by the authors, this is not one of them
- Maybe I just didn't take enough time to try and understand it
Reply:

We apologize for the oversight in omitting a label during the figure adjustments. This issue has now been corrected. Additionally, we have provided further clarification regarding the expected topological structure in the revised manuscript, which can be found on lines 10–15 of page 17.
We also regret not paying sufficient attention to the support values in the original version. Bootstrap values have now been included in the updated phylogenetic trees, which can be found in Supplementary Figures 13–15.

Editorial Manager®

Cookie Preference Center

We use cookies which are necessary to make our site work. We may also use additional cookies to analyze, improve and personalize our content and your digital experience. For more information, see our Cookie Policy.

You may choose not to allow some types of cookies. However, blocking some types may impact your experience of our site and the services we are able to offer. See the different category headings below to find out more or change your settings.

Allow all

Manage Consent Preferences

Strictly Necessary Cookies

Always active

These cookies are necessary for the website to function and cannot be switched off in our systems. They are usually only set in response to actions made by you which amount to a request for services, such as setting your privacy preferences, logging in or filling in forms. You can set your browser to block or alert you about these cookies, but some parts of the site will not then work. These cookies do not store any personally identifiable information.

Functional Cookies

These cookies enable the website to provide enhanced functionality and personalisation. They may be set by us or by third party providers whose services we have added to our pages. If you do not allow these cookies then some or all of these services may not function properly.

Performance Cookies

These cookies allow us to count visits and traffic sources so we can measure and improve the performance of our site. They help us to know which pages are the most and least popular and see how visitors move around the site.

Targeting Cookies

These cookies may be set through our site by our advertising partners. They may be used by those companies to build a profile of your interests and show you relevant adverts on other sites. If you do not allow these cookies, you will experience less targeted advertising.

Cookie List

Clear

Apply Cancel

Consent Leg.Interest

Confirm my choices

Powered by onetrus
