# Supplementary material for: The genome of Hippophae salicifolia provides new insights into the sexual differentiation of sea buckthorn
Source: Gigascience. 2025 Jul 2;14:giaf046. doi: 10.1093/gigascience/giaf046 (PMC12218201; doi:10.1093/gigascience/giaf046)
Supplement: giaf046_Reviewer_1_Report_original_submission — Zhiqiang Wu -- 10/28/2024 [file giaf046_reviewer_1_report_original_submission.pdf]

Reviewer Report

Title: The genome of Hippophae salicifolia provides new insights into the sexual differentiation of seabuckthorn

Version: Original Submission **Date:** 10/28/2024

Reviewer name: Zhiqiang Wu

Reviewer Comments to Author:

I have read the manuscript titled "The genome of Hippophae salicifolia provides new insights into the sexual differentiation of seabuckthorn" from Chen and colleagues. This work assembled and annotated the genomes of two Hippophae species, with an emphasis on mapping sex-determining regions (SDRs) and comparing sex chromosomes. The research uncovered differences in structural variations between sex chromosomes, and identified candidate genes related to SDR. These findings provide important insights for understanding the evolution of sex determination and will be valuable resources in seabuckthorn. The manuscript is well organized and the most important analyses and information were included, but the authors need to address some issues that are described below.

1. In Figure S2, some blanks were exhibited in HiC cluster, but these areas were not all blank in different assemblies. For example, the blanks in Chr05 and Chr08 were different among female, hap1 and hap2 genomes, why?
2. Page 8, Hap1 and Hap2 of male H. salicifolia genomes were 309.06-351.7 Mb larger than those of females by about 40%. How to explain these significant differences?
3. Page 9, why is the BUSCO completeness rates scores of genomes (97.4%, 97.6%, 97.6%, 97.7%) less than those of gene set (98.9%, 98.8%, 98.9%, 98.7%)?
4. Page 10, "Synteny analysis revealed one-to-one homology relationships … a higher frequency of chromosomal rearrangements."

This part of the results were not presented in any graphical or tabular form and need to supplemented.

5. Page 11, "Applying the same methodology to H. gyantsensis, … 33.86 Mb to 44.78 Mb and contained 235 genes."

The final SLR regions in H. gyantsensis was 12.31 Mb from 14.59 - 15.97 Mb and 33.86 - 44.78 Mb, but the depths in 14.59 - 15.97 Mb and 39.47 - 44.78 Mb do not differ between male and female as those in the H. salicifolia genome. In the presence of species difference, why refine the SLR with collinearity when you have precise results for this analysis?

6. Page 13, "These observations indicate a lineage-specific chromosomal fusion event in seabuckthorn, and Chr02 was possibly derived from the merging of two ancestral chromosomes."

Even if previous studies suggest that dioecy may be a newer evolutionary state, we can't identify directly from the phylogenetic tree either Hippophae or Elaeagnus was closer to the ancestor. Without a covariance comparison between the common ancestor and these two clades, I think the current results may only indicate a break or fusion occurred in Chr02. Other species that diverged earlier in the Hippophae or Elaeagnus should be added for collinearity comparison and illustration if retaining the existing conclusions.

7. Page 13, "The collinear regions were categorized into nine distinct blocks through comparison of chromosomal segment synteny (Supplementary Fig. S10 and Table S9)."
There are 10 blocks from A to H in the Supplementary Table S9, why was H not used together in the analysis?
8. Page 13, "Block D had the highest Ks value (Ks=0.1266), … and block G (Ks=0.0037)."
It seems that the mean Ks value of the gene pairs was used as the result, but it was not mentioned here or in the method. It should be clarified.
9. Page 14, "Stratum 1 (including blocks C and D) was located …. Stratum 2-1 (including blocks A and B) was located …. Stratum 2-2 (including blocks E, F, and G) was located … with an average Ks value of 0.0418 (Fig 4c)."
From the Supplementary Table S10, we can see that the mean Ks value of block C was significant different from that of block D, and they were also relatively far apart from each other in chromosome, but why was it divided into the same group?
10. Page 14, "Distinct phylogenetic patterns were observed … tended to cluster together." This point needs to be supported by references.
11. Page 14, "In Stratum 1, an ancient origin was observed for 16 out of 34 single-copy genes;"
There were 17 Structure1 trees in Stratum 1 which was showed in Fig 4d, please check this result.
12. Page 15, "We utilized transcriptome data, compared these data with the Y-SLR of H. salicifolia, and calculated the transcripts per million (TPM) values of the transcripts (Supplementary Table S15)."
"Supplementary Table S15" should be amended to "Supplementary Table S12". Additionally, there was no stem sampling and biological replicates of three tissues mentioned in METHOD.
13. Page 19, "however, the results of our study indicate that X-linked structural variations might be selected and fixed to suppress recombination between XY chromosomes."
It seems that I don't find relevant results, where was this conclusion being drawn from?

Methods

Are the methods appropriate to the aims of the study, are they well described, and are necessary controls included? Choose an item.

Conclusions

Are the conclusions adequately supported by the data shown? Choose an item.

Reporting Standards

Does the manuscript adhere to the journal’s guidelines on minimum standards of reporting? Choose an item.

Choose an item.

Statistics

Are you able to assess all statistics in the manuscript, including the appropriateness of statistical tests used? Choose an item.

Quality of Written English

Please indicate the quality of language in the manuscript: Choose an item.

Declaration of Competing Interests

Please complete a declaration of competing interests, considering the following questions:

- Have you in the past five years received reimbursements, fees, funding, or salary from an organisation that may in any way gain or lose financially from the publication of this manuscript, either now or in the future?
- Do you hold any stocks or shares in an organisation that may in any way gain or lose financially from the publication of this manuscript, either now or in the future?
- Do you hold or are you currently applying for any patents relating to the content of the manuscript?
- Have you received reimbursements, fees, funding, or salary from an organization that holds or has applied for patents relating to the content of the manuscript?
- Do you have any other financial competing interests?
- Do you have any non-financial competing interests in relation to this paper?

If you can answer no to all of the above, write 'I declare that I have no competing interests' below. If your reply is yes to any, please give details below.

no

I agree to the open peer review policy of the journal. I understand that my name will be included on my report to the authors and, if the manuscript is accepted for publication, my named report including any attachments I upload will be posted on the website along with the authors' responses. I agree for my report to be made available under an Open Access Creative Commons CC-BY license (http://creativecommons.org/licenses/by/4.0/). I understand that any comments which I do not wish to be included in my named report can be included as confidential comments to the editors, which will not be published.

Choose an item.

To further support our reviewers, we have joined with Publons, where you can gain additional credit to further highlight your hard work (see: https://publons.com/journal/530/gigascience). On publication of this paper, your review will be automatically added to Publons, you can then choose whether or not to claim your Publons credit. I understand this statement.

Yes Choose an item.
