# Supplementary material for: The genome of Hippophae salicifolia provides new insights into the sexual differentiation of sea buckthorn
Source: Gigascience. 2025 Jul 2;14:giaf046. doi: 10.1093/gigascience/giaf046 (PMC12218201; doi:10.1093/gigascience/giaf046)
Supplement: giaf046_Reviewer_2_Report_original_submission — Alan E. Yocca -- 11/26/2024 [file giaf046_reviewer_2_report_original_submission.pdf]

Reviewer Report

Title: The genome of Hippophae salicifolia provides new insights into the sexual differentiation of seabuckthorn

Version: Original Submission **Date:** 11/26/2024

Reviewer name: Alan E. Yocca

Reviewer Comments to Author:

This manuscript does an excellent job describing the sex-linked region of seabuckthorn. Overall, the introduction provides good context for why this analysis is needed, and the results are logically ordered with most of their hypotheses supported directly with their data. The identification of candidate genes with orthologs in Arabidopsis is a particularly exciting result. Even though no molecular characterization is performed, that is certainly beyond the scope of this manuscript. It is a complete genome assembly and analysis manuscript. I do have a number of minor comments where I believe further clarification is required, but overall kudos to the authors.
- "increase number of acquired genes in X SLR"
- page 12, how can you separate that from the hypothesis of an increased loss in Y instead of insertions in X?
- a better explanation of what "ancestral" and "acquired" genes are is required
- what was the sampling of Male / Female? Could differences be variation within the species instead of just between male / female?
- Throughout, some genus and species names are not italicized

last paragraph of the introduction:
"The genetic basis of sexual differentiation in seabuckthorn has been a major focus of studies of plant reproductive biology and sexual evolution"
- Please support this statement with citations

You should replace all references of "Gypsy" elements to "Ty3"
- https://doi.org/10.31219/osf.io/fma57

Figure 4A:
- The units for the x-axis need labeled at least in the figure legend

Figure 5:
- A legend for the heatmap is required, it is ambiguous which colors are high or low expression

Page 15:
". Sex-determining genes often share phylogenetic similarities with ancient strata genes, and homologous genes from the same gametophyte tended to cluster together"
- this statement needs a citation or further justification as to why certain gene tree topologies are expected

Page 16:
"Additionally, the pseudogenization of Hsam2h02g1248 was observed in the female counterparts of both H. gyantsensis and H. salicifolia, which resulted in the loss of its original function"

- Amino acid alignments are shown in Figure S15, but an additional check of genome sequences is required to ensure true psuedogenization instead of a misannotation of these loci in females
Discussion:
- mentioning the WGD history of these lineages should be mentioned in the introduction. It makes figure 4A make a lot more sense so mentioning it when describing figure 4A may also contextualize the multiple observed syntenic hits
- this makes Figure 4A even more convincing, there are 4 copies of L and R regions, and only a single pair exist on the same chromosome!
- "The SLRs of papaya and kiwifruit have evolved from the pericentromeric
regions [38-40]."
- this sentence seems out of place, maybe:
'sex chromosomes are known to have independently evolved through similar mechanisms in other lineages such as...'
Figure S2:
- The hi-c maps provided are not high enough resolution to evaluate the accuracy of the SLR. It would be helpful to provide an enlarged contact matrix just for chromosome 2 to increase confidence in the order and orientation of the SLR. Alternatively, please provide a statement in the part discussing Figure 4B that the observed lack of synteny may be observed due to misassembly in either the SLR in this manuscript, or in the genomes compared in this manuscript.
- HiFi genome assemblies are incredibly high quality and I do not doubt the author's expertise in carefully evaluating the accuracy of their genome assembly. I'm just a stern believer that genome assemblies are hypotheses and not ground truth; a point readers, especially non-specialists, often miss.
Figure S12A:
- the number of tips on the cladogram and labels do not match.
- additionally, I'm not sure how this supports the author's hypotheses as they do not explain sufficiently what an expected topology is, or why it is expected. Bootstrap values and details on phylogenetic reconstruction are not easy to find.
- It also needs mentioned why a gene tree might deviate from expectation. When compared a set of orthologues, minute differences can change gene tree topology relative to the species tree. Support values for each node should be provided as well.
- I really enjoy most of the analyses set forth by the authors, this is not one of them
- Maybe I just didn't take enough time to try and understand it

Methods

Are the methods appropriate to the aims of the study, are they well described, and are necessary controls included? Choose an item.

Conclusions

Are the conclusions adequately supported by the data shown? Choose an item.

Reporting Standards

Does the manuscript adhere to the journal's guidelines on [minimum standards of reporting?]() Choose an item.

Choose an item.

Statistics

Are you able to assess all statistics in the manuscript, including the appropriateness of statistical tests used? Choose an item.

Quality of Written English

Please indicate the quality of language in the manuscript: Choose an item.

Declaration of Competing Interests

Please complete a declaration of competing interests, considering the following questions:

- Have you in the past five years received reimbursements, fees, funding, or salary from an organisation that may in any way gain or lose financially from the publication of this manuscript, either now or in the future?
- Do you hold any stocks or shares in an organisation that may in any way gain or lose financially from the publication of this manuscript, either now or in the future?
- Do you hold or are you currently applying for any patents relating to the content of the manuscript?
- Have you received reimbursements, fees, funding, or salary from an organization that holds or has applied for patents relating to the content of the manuscript?
- Do you have any other financial competing interests?
- Do you have any non-financial competing interests in relation to this paper?

If you can answer no to all of the above, write 'I declare that I have no competing interests' below. If your reply is yes to any, please give details below.

I declare that I have no competing interests

I agree to the open peer review policy of the journal. I understand that my name will be included on my report to the authors and, if the manuscript is accepted for publication, my named report including any attachments I upload will be posted on the website along with the authors' responses. I agree for my report to be made available under an Open Access Creative Commons CC-BY license (http://creativecommons.org/licenses/by/4.0/). I understand that any comments which I do not wish to be included in my named report can be included as confidential comments to the editors, which will not be published.

Choose an item.

To further support our reviewers, we have joined with Publons, where you can gain additional credit to further highlight your hard work (see: https://publons.com/journal/530/gigascience). On publication of this paper, your review will be automatically added to Publons, you can then choose whether or not to claim your Publons credit. I understand this statement.

Yes Choose an item.
